# Regio- and Enantioselective *N*-Heterocyclic Carbene-Catalyzed Annulation of Aminoindoles Initiated by Friedel–Crafts Alkylation

**DOI:** 10.1021/acs.orglett.4c02434

**Published:** 2024-08-08

**Authors:** Vojtěch Dočekal, Yaroslava Niderer, Adam Kurčina, Ivana Císařová, Jan Veselý

**Affiliations:** † Department of Organic Chemistry, Faculty of Science, Charles University, Hlavova 2030/8, 128 43 Prague 2, Czech Republic; ‡ Faculty of Sciences, Aix-Marseille University, 52 Av. Escadrille Normandie Niemen, 13013 Marseille, France; § Department of Inorganic Chemistry, Faculty of Science, Charles University, Hlavova 2030/8, 128 43 Prague 2, Czech Republic

## Abstract

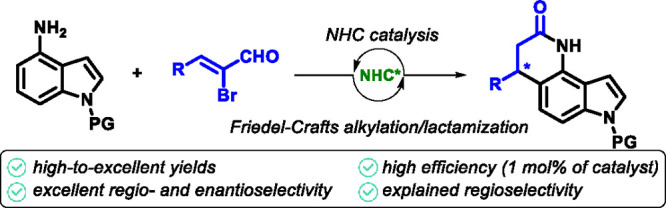

Chiral indoles annulated on the benzene ring are unique and significant in natural and medicinal compounds. However, accessing these enantioenriched molecules has often been overlooked. The present study introduces an organocatalytic protocol to access these compounds efficiently, demonstrated by substrate scope, functional group tolerance, and using only 1 mol % of a chiral conjugated acid catalyst. Additionally, the study explores regioselectivity, gram-scale reactions, and follow-up transformations, underscoring the method’s potential.

The indole structural motif and its derivatives, including indole-annulated (or fused) carbo- and heterocycles, are core motifs in various natural products (Figure 1A),^1^ medicinally relevant compounds,^2^ agrochemicals,^3^ and dyes.^4^ The inherent electron-rich nature of indoles dictates their primary synthetic utility, which is typically represented by electrophilic aromatic substitutions.^5^ In this context, Friedel–Crafts alkylation (FCA), discovered by Charles Friedel and James Crafts in 1877, has been one of the most valuable methods for C–C bond formation via electrophilic aromatic substitution. Almost 150 years after its pioneering works, FCA remains a viable and highly relevant approach, with indoles,^6^ pyrroles,^7^ and many other compounds^8^ serving as common starting materials. Due to the connection between biological activity and stereochemistry, developing novel asymmetric synthetic routes toward that unique structural motif presents a significant challenge. Generally, an asymmetric pathway to chiral indoles (and their annulated derivatives) relies on an enantioselective Friedel–Crafts alkylation.^9^ The most extensively studied organocatalytic FCA involves the activation of an azole ring by chiral Brønsted or Lewis acids,^10^ typically promoting FCA at the C_2_ or C_3_ indole positions (Figure 1B, left). In contrast, asymmetric methods targeting the benzene ring of indole are much less developed. To achieve remote regioselectivity in the substitution reaction, a directing group (usually electron-donating groups like *N*-substituted amines) is employed (Figure 1B, right). Asymmetric induction of FCA is then determined by the regioselective attack on the chiral electrophile.^11^ In the context of asymmetric synthesis of chiral annulated indoles, three major organocatalytic approaches have been identified. One of the most common pathways involves an organocascade reaction of indoles substituted at the C_7_ position with a functional group crucial for the enantiodiscrimination step, followed by *N*-substitution of the indole nitrogen.^12,13^ A second approach has been applied to C_4_-substituted indoles (for example, those with Michael acceptors), where the sequence is initiated by asymmetric FCA at the C_3_ position of the indole, followed by a ring-closing reaction involving the functional group at C_4_.^14,15^ The significantly less explored third pathway involves the use of substituted indoles, where directing groups enable remote regioselective enantioselective FCA followed by a ring-closing reaction. This approach has been primarily limited to hydroxyindoles, which have been used in sequences catalyzed by chiral bifunctional organocatalysts.^16^ These catalysts facilitate FCA followed by an annulative nucleophilic attack of the hydroxy group. Nowadays, organocatalytic activations of various carbonyl compounds are induced by chiral *N*-heterocyclic carbenes (NHCs).^17^ With easily accessible bench-stable chiral precursors, NHC organocatalysis offers a broad area of various activation modes and represents the current flagship in asymmetric synthesis.^18^ For example, unsaturated acyl-azolium intermediate easily formed from α-bromocinnamic aldehyde and NHC in the presence of base, resulting in a versatile chiral a^3^-synthon.^19^ This intermediate allows a broad scope of asymmetric annulation reaction providing various chiral heterocycles.^20,21^ Drawing inspiration from the regioselective FCA of *N*-substituted 4-aminoindoles, we propose an efficient approach for regio- and enantiocontroled NHC-catalyzed Friedel–Crafts alkylation/lactamization sequence for the annulation of readily available aminoindoles. We utilize formation of chiral α,β-unsaturated acyl-azolium intermediate from α-bromocinnamic aldehyde (Figure 1C) without any external oxidant.

**Figure 1:**
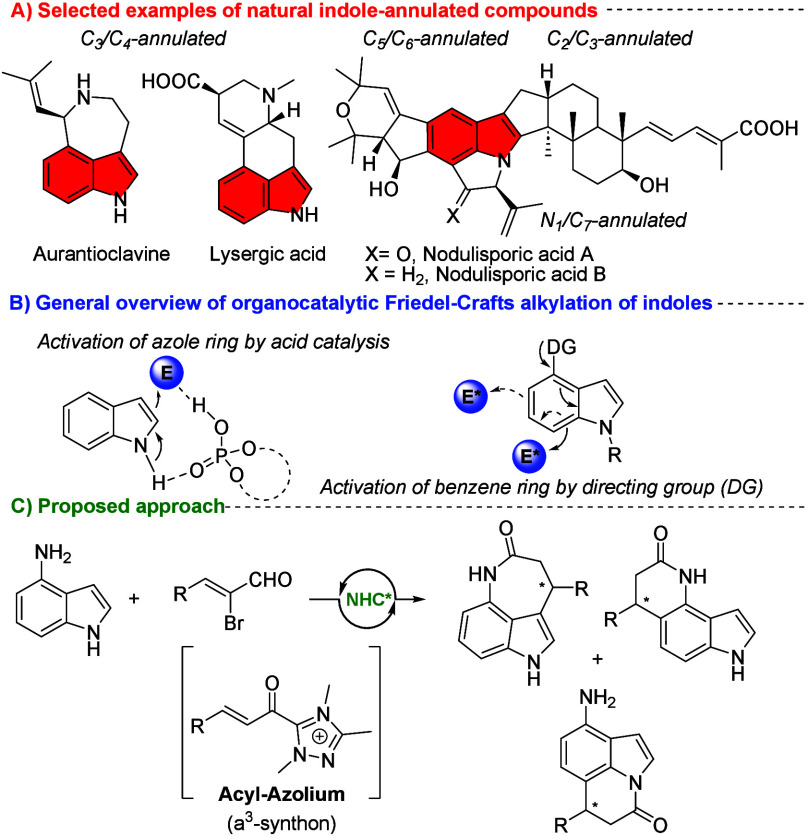
(A) Selected examples of natural indole-annulated compounds. (B) General overview of organocatalytic Friedel–Crafts alkylation of indoles. (C) Proposed approach.

From the outset of our study, we chose unprotected aminoindole (**1a**), considering the possible formation of three regioisomeric products (Figure 1C). To our delight, simply mixing indole **1a** with α-bromocinnamic aldehyde **2a** and an excess of sodium carbonate as a base in the presence of Rovis triazolinium salt (*pre*-**C1**) produced the six-membered lactam **3a**. The compound **3a** was isolated with good stereochemical outcome (74:26 *er*), albeit as a hardly separable mixture with amide **4a** (Table 1, entry 1). This proof-of-concept result motivated us to switch the starting indole substrate to *N*-methyl protected aminoindole **1b**. Moreover, we hypothesized that the presence of the *N*-alkyl group may increase the nucleophilic character of C_5_, and reduce the polarity of the expected product, thereby resolving separation difficulties. As a result of the switch of starting indole, a significantly increased isolated yield of **3b** (61%, entry 2) was observed without forming the parasitic byproduct **4b**. Based on this, we chose substrate **1b** for further reaction condition optimizations. Encouragingly, the model reaction of **1b** with **2a** conducted in the presence of the aminoindanole-based triazolinium salt *pre*-**C2** (entry 3) produced the expected product in excellent yield and enantiomeric excess (86%, 96:4 *er*). Moreover, the model reaction in the presence of the conjugated acid of a bifunctional catalyst (*pre*-**C3**), combining NHC with a hydrogen-bonding tertiary alcohol, showed similar enantioselectivity but a lower yield (58%, 96:4 *er*). Other conjugated NHC acids, including the l-phenylalanine-derived acid (*pre*-**C4**), did not show better efficiency (for a complete optimization survey, please refer to the SI file). Notably, the model reaction exhibited lower tolerance to bases but good tolerance toward solvents. For example, a model reaction conducted in the presence of potassium phosphate (entry 6) resulted in a lower yield of **3b**. Among the tested organic bases, lactam **3b** was isolated only in the presence of 2,6-lutidine (entry 7) Slightly increased enantiocontrol was observed in model reactions conducted in chloroform, benzene, EtOAc, or THF (entries 8–11). Based on the yield of **3b**, we chose chloroform (entry 8) as the suitable solvent for further optimization (72%, 98:2 *er*). Notably, the process demonstrated extraordinary efficiency by reducing the amount of triazolinium salt. Surprisingly, we did not observe any significant negative impact on yield or enantiocontrol. The use of only 1 mol % of the conjugated acid of the catalyst (*pre*-**C2**, entry 12) produced lactam **3b** in excellent yield and enantiomeric excess (84%, 97:3 *er*). Further variations in reaction conditions, such as temperature lowering, did not yield better outcomes (entry 13).

**Table 1:**
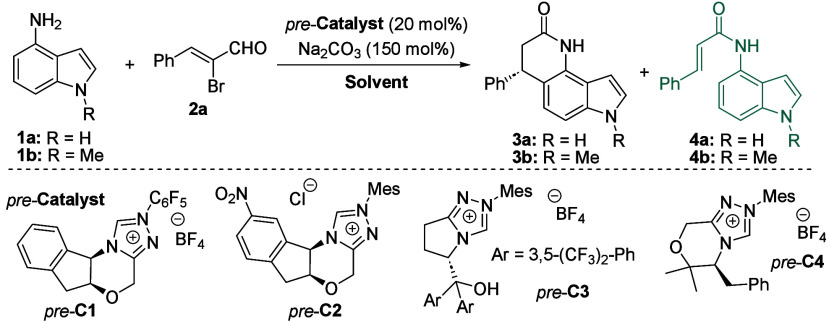
Optimization Studies

Entry^a^	*pre*-**Cat**.	Solvent	Time (h)	Yield^b^ (**3b**, %)	*Er*^c^ (**3b**)
1^d^	*pre*-**C1**	DCM	15	25	74:26
2	*pre*-**C1**	DCM	15	61	68:32
3	*pre*-**C2**	DCM	24	86	96:4
4^e^	*pre*-**C3**	DCM	24	58	96:4
5^e^	*pre*-**C4**	DCM	24	67	18:82
6^f^	*pre*-**C2**	DCM	24	53	96:4
7^g^	*pre*-**C2**	DCM	24	50	95:5
8	*pre*-**C2**	CHCl_3_	15	73	98:2
9^h^	*pre*-**C2**	benzene	72	66	98:2
10^e^	*pre*-**C2**	EtOAc	48	70	97:3
11^e^	*pre*-**C2**	THF	15	61	97:3
12^i^	*pre*-**C2**	CHCl_3_	15	84	97:3
13^i^,^j^,^h^	*pre*-**C2**	CHCl_3_	72	74	98:2

a Reactions were conducted with **1b** (0.2 mmol), enal **2a** (0.3 mmol), Na_2_CO_3_ (0.3 mmol), and *pre*-catalyst (20 mol %) in selected solvent (1.0 mL) at room temperature.

b Isolated yield after column chromatography.

c Determined by chiral HPLC analysis.

d 1*H*-indol-4-amine (**1a**) was used instead of **1b**; product **3a** was isolated.

e Full consumption of **1b** was not observed; aldehyde **2a** disappeared.

f K_3_PO_4_ was used.

g 2,6-lutidine was used.

h Full consumption of **1b** was not observed.

i Na_2_CO_3_ (0.4 mmol) and *pre*-catalyst (1 mol %) were used.

j Reaction was performed at 0 °C.

After optimizing the reaction conditions, we began exploring the scope of the annulation reaction with various *N*-substituted 4-aminoindoles **1** (Scheme 1A). Initially, the reaction of the model substrate **1b** conducted with the opposite enantiomeric form of the conjugated acid of the catalyst (*ent*-*pre*-**C2**) produced the expected opposite enantiomeric product *ent*-**3b** in high yield (90%) and excellent enantiopurity (98:2 *er*). Additionally, the absolute configurations of both product **3b** and *ent*-**3b** were confirmed by X-ray analysis. Similar results in terms of yield and enantiocontrol were observed when the starting indole **1** was *N*-substituted with electron-donating alkyl groups, such as propyl, allyl, or benzyl. Additionally, no deviation in terms of yield or stereocontrol was observed for C_3_-methylated aminoindole (**3f**, 91%, 96:4 *er*). As expected, the reaction efficiency was lower, accompanied by the aforementioned separation problems, when unprotected aminoindole (**1a**) was used. Nonetheless, the stereochemical outcome remained good (94:6 *er*). The results indicated that the developed method was not suitable for *N*-substituted indoles with electron-withdrawing groups, such as tosyl or Boc, due to the decreased nucleophilic character of the benzene ring in the starting indoles, as well as for *N*-substituted indoles with substituted nitrogen at position 4 of indole (R^1^ = Bn, Ts; PG = Me). Next, the scope of the developed method was explored using various α-bromocinnamic aldehydes (Scheme 1B). In general, introducing a variety of enals yielded excellent yields and stereocontrol of the annulation process, with excellent functional group tolerance.

**Scheme 1:**
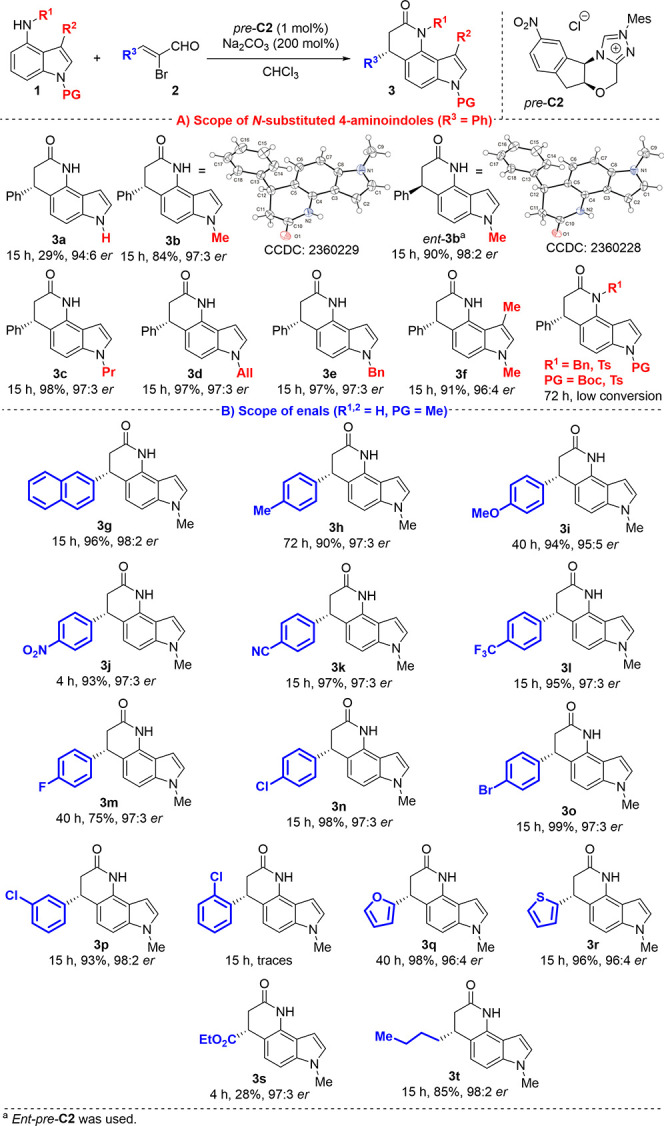
Substrate Scope of Annulation

Specifically, α-bromocinnamic aldehydes substituted with electron-donating groups at the *para* position of the benzene ring showed a slightly lower reactivity, resulting in prolonged reaction times. However, the corresponding products **3h** and **3i** were isolated with excellent yields (90 and 94%) and stereochemical outcomes (97:3 and 95:5 *er*). Reactions with electron-withdrawing groups at the same position provided the corresponding products **3j**-**o** in nearly quantitative yields (typically over 95%) with identical enantiomeric purities (all 97:3 *er*). Then, we assessed the effect of steric hindrance in α-bromocinnamic aldehydes substituted at the *ortho* or *meta* positions of the benzene ring. The annulation reaction of *meta*-substituted cinnamic aldehyde resulted in the formation of product **3p** with excellent efficiency (93%, 98:2 *er*). However, no conversion to the expected product was observed for *ortho*-substituted cinnamic aldehyde, likely due to increased steric hindrance. Subsequently, we explored the scope of this method using heteroaromatic or aliphatic aldehydes. We found that the expected products **3q**-**t** were isolated with excellent enantiomeric purities (96:4–98:2 *er*) and excellent isolated yields (over 85%), except for **3s**, which was unexpectedly obtained in a lower yield. To assess our method, we introduced a series of regioisomeric aminoindoles (Scheme 2). Using 5- and 7-aminoindole derivatives, we successfully obtained the expected annulation products **3u** and **3v**. For example, annulation product **3v** was isolated with a good yield and excellent stereochemical outcome (71%, 96:4 *er*). On the other hand, the introduction of 6-aminoindole predominantly led to the formation of the amidation product **4**. Further exploration with sulfur and oxygen analogs of indoles did not result in any conversion of the starting material under the optimized reaction conditions.

**Scheme 2:**
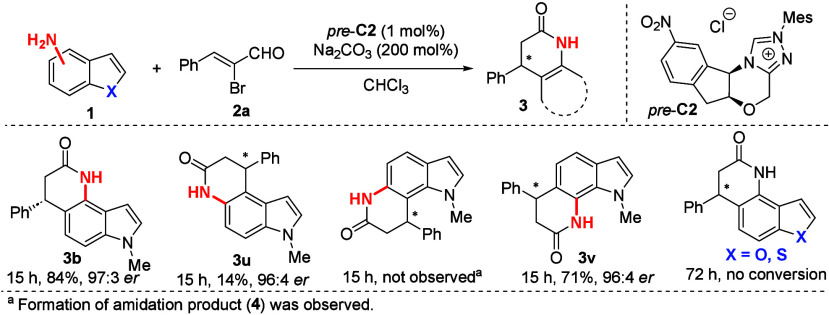
Substrate Scope of Regioisomeric Aminoindoles

To elucidate the regioselectivity of the annulation process, we performed DFT calculations of the energies for three expected regioisomeric products (Figure 1C) during the annulation of unprotected indole (when PG = H, **1a**). Based on the calculated free energies of the products, the most stable product was identified as **3a**. The expected seven-membered C_3_-annulated product followed, with an energy gap of around 3 kcal/mol (3.4 kcal/mol in chloroform, 2.7 kcal/mol in the gas phase). The highest energy gap was found for the six-membered C_7_-annulated product, with a difference of around 6 kcal/mol compared to **3a** (7.1 kcal/mol in chloroform, 5.1 kcal/mol in the gas phase). A similar energy gap was calculated for the proposed seven-membered C_3_-annulated product in the reaction of **1b**. In this case, the energy gap was approximately 3 kcal/mol (3.4 kcal/mol in chloroform, 2.9 kcal/mol in the gas phase), consistent with the findings for **1a**. Additionally, we studied the nucleophilic character of various positions of 4-aminoindoles **1a** and **1b** using the condensed Fukui function.^22^ We revealed that the C_7_ position is the most nucleophilic in both indoles **1a** and **1b**, which aligns with previously reported Friedel–Crafts alkylation at this position (please see the introduction part). For a complete DFT survey, please refer to the SI file. To demonstrate the practicality of the developed method, we performed a gram-scale annulation of **1b** using the optimized conditions (Scheme 3A). This gram-scale transformation yielded product **3b** in a high yield of 91% with 97:3 *er*, with a slightly prolonged reaction time. Additionally, the extraordinary efficiency of the methodology was validated by using only 1 mol % of the conjugated acid of a chiral catalyst. The follow-up transformations of the enantioenriched product **3b** highlight its synthetic utility and increase molecular complexity through modifications of both the amide and indole parts (Scheme 3B). The secondary amide nitrogen of **3b** was methylated using an excess of sodium hydride for deprotonation, followed by the addition of methyl iodide, resulting in the formation of tertiary amide **5** in high yield (81%). Similarly, lithium aluminum hydride reduction of the amide of **3b** provided amine **6** in good yield (56%). We also explored indole modifications, such as the reduction of the azole double bond or enone FCA. To achieve FCA at the C_3_ position of indole **3b**, we tested both Lewis and Brønsted acid catalytic conditions. To our delight, the corresponding product **7** was isolated under both conditions. Notably, under Brønsted acid catalysis, we achieved nearly a quantitative yield of **7**. Finally, we prepared the dihydroindole derivative **8** with a good yield of 66% under reductive conditions using sodium cyanoborohydride. In all cases, there was no observed deviation in optical purity for any of the follow-up transformations.

**Scheme 3:**
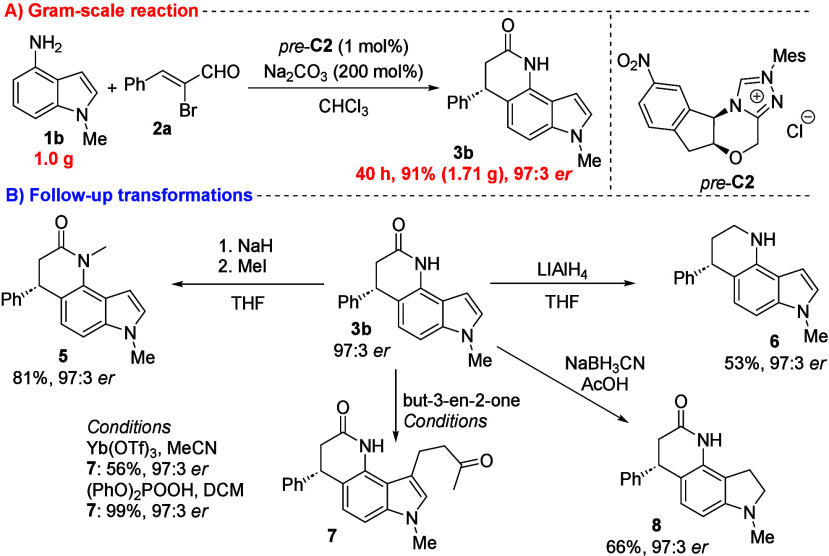
Gram-Scale Reaction and Synthetic Utility Demonstration

In summary, we have developed an efficient organocatalytic methodology for NHC-catalyzed enantioselective annulation of aminoindoles with α-bromocinnamic aldehydes.^23^ This novel approach provides robust access to chiral annulated indoles with a broad substrate scope and excellent functional group tolerance, utilizing only 1 mol % of a chiral conjugated acid catalyst. Additionally, DFT calculations have provided insights into the observed regioselectivity. The methodology has been demonstrated to be effective on a gram scale and allows follow-up transformations that increase the molecular complexity of the obtained chiral annulated indoles. This underscores the feasibility and potential of the novel methodology for future applications. Ongoing work in our laboratory includes applications in medicinal chemistry and further investigation of other enantioselective annulations.

## Data Availability

The data underlying this study are available in the published article and its Supporting Information.

## Supporting Information Available

Reaction conditions optimization, experimental procedures and characterization data for all compounds, crystallographic data, description of computational methods, copies of ^1^H NMR, ^13^C NMR, ^19^F NMR, and copies of chiral HPLC (PDF)
FAIR data, including the primary NMR FID files, for compounds **3a–t** and related compounds (ZIP)
