# Supplementary material for: Regio- and Enantioselective N-Heterocyclic Carbene-Catalyzed Annulation of Aminoindoles Initiated by Friedel–Crafts Alkylation
Source: Org Lett. 2024 Aug 8;26(33):6993–8. doi: 10.1021/acs.orglett.4c02434 (PMC11348421; doi:10.1021/acs.orglett.4c02434)
Supplement: Supplementary file 1 — ol4c02434_si_001.pdf [file ol4c02434_si_001.pdf]

Regio- and Enantioselective *N*-Heterocyclic Carbene-Catalyzed Annulation of Aminoindoles Initiated by Friedel-Crafts Alkylation

Vojtěch Dočekal,[1] Yaroslava Niderer.[1,2] Adam Kurčina, [1] Ivana Císařová[3] and Jan Veselý[1*]*

[1] Department of Organic Chemistry, Faculty of Science, Charles University, Hlavova 2030/8, 128 43 Prague 2, Czech Republic, e-mail: vojtech.docekal@natur.cuni.cz, jan.vesely@natur.cuni.cz

[2] Faculty of Science, Aix-Marseille University, 52 Av. Escadrille Normandie Niemen, 13013 Marseille, France

[3] Department of Inorganic Chemistry, Faculty of Science, Charles University, Hlavova 2030/8, 128 43 Prague 2, Czech Republic

Supporting Information

General **2**

Starting material **2**

General procedures for preparation of amino derivatives *2*

Characterization data of amino derivatives *3*

Annulation reaction **8**

Complete reaction optimization survey *8*

General procedure for annulation reaction *14*

Characterization data of products *14*

Follow-up transformations **22**

Crystallographic data **24**

Computational methods **27**

Regiochemistry study – stability of products *27*

Regiochemistry study – Condensed Fukui function as a measure of electrophilicity *30*

XYZ data *31*

NMR spectra **73**

Chiral HPLC **116**

References **143**

General

Chemicals and solvents were purchased from commercial suppliers and purified using standard techniques. Thin-layer chromatography (TLC) was performed using silica gel plates Merck 60 F_{254}. The compounds were visualized by irradiation with UV light and/or by treatment with a solution of phosphomolybdic acid (AMC) followed by heating. Column chromatography was performed using silica gel Fluka (40-63 μm) or SiliCycle-SiliaFlash P60 (particle size: 40-63 μm, pore diameter: 60 Å. ^{1}H, ^{13}C NMR, and ^{19}F spectra were recorded with Bruker AVANCE III 400. Chemical shifts for protons are given in δ relative to tetramethylsilane (TMS) and referenced to residual protium in the NMR solvent (chloroform-*d*: $\delta_H = 7.26$ ppm). Chemical shifts for carbon are referenced to the carbon of the NMR solvent (chloroform-*d*: δ_C = 77.16 ppm). The coupling constants J are given in hertz. IR DRIFT spectra were recorded on a Nicolet AVATAR 370 FT-IR in cm^{-1}. Chiral HPLC was performed on a LC20AD Shimadzu liquid chromatograph with an SPD-M20A diode array detector with Daicel Chiralpak® IA, Daicel Chiralpak® IB, and Phenomenex Lux® Amylose-1 columns. For chiral HPLC, the samples were prepared by dissolving them in *i*-PrOH. Optical rotations were measured on AU-Tomatica polarimeter, Autopol III, and specific optical rotations are given in concentrations *c* [g/100 ml], the samples were prepared by dissolving them in solvent specified solvent for each compound. All melting points were measured on a Büchi melting point B-545 apparatus, in an open glass capillary, and all values are uncorrected. High-resolution mass spectra were recorded on an LCQ Fleet spectrometer using a Bruker Compact QTOF-MS controlled by the Compass 1.9 Control software to measure the ESI high-resolution mass spectrums. The monoisotopic mass values were calculated using Data analysis software v 4.4. The analysis was conducted in the positive ion mode at a scan range from m/z 50 to 1000, and nitrogen was used as nebulizer gas at a pressure of 4 psi and flow of 3 l/min for the dry gas. The capillary voltage and temperature were set at 4500 V and 220 °C, respectively. For HRMS, the samples were prepared by dissolving them in methanol.

Starting material

General procedures for preparation of amino derivatives

General procedure A: Alkylation/Pd-mediated catalytic hydrogenation

The round-bottom flask was charged with a magnetic stirrer and corresponding nitroindole derivative (1.0 equiv.), followed by anhydrous DMF (0.5 M with respect to nitroindole). Then, mixture was cooled to 0 °C (ice/water bath) and suspension of sodium hydride (60% *w*/*w* in oil, 1.2 equiv.) was added portionwise (gas evolution). The reaction mixture was heated up to room temperature and left to stir for 30 minutes. Then, corresponding alkylating agent (1.1 equiv.) was added dropwise at 0 °C (ice/water). Resulting mixture was left to stir at room temperature. Once the starting nitroindole was no longer detected (by TLC, usually overnight reaction), the reaction was quenched by slow addition of water (100 ml per 10 mmol of starting material). Mixture was diluted with EtOAc (100 ml per 10 mmol of starting material). The organic phase was separated (in some cases separation was accelerated by addition of brine). The water phase was extracted with EtOAc (3 × 100 ml per 10 mmol of starting material). The collected organic phases were washed with brine (2 × 100 ml) and dried under anhydrous $MgSO_4$. After filtration of the solid, the filtrate was concentrated under reduced pressure. The crude product was used directly to a second step without other purification (yield usually above 80%).

Corresponding *N*-alkylated nitro derivative (1.0 equiv.) was dissolved in methanol (0.5M with respect to starting nitro derivative). Resulting mixture was degassed, followed by addition of Pd/C (10% w/w, 0.05 equiv.) at room temperature and left to stir overnight under pressure of

hydrogen (balloon). Once the starting nitroindole was no longer detected (by TLC), the reaction was filtered through short pad of Celite and washed with MeOH. Resulting filtrate was evaporated. The crude product was purified by column chromatography (eluting by hexane/EtOAc).

General procedure B: Alkylation/Fe-mediated nitro reduction
Alkylation step was performed using the same procedure as described in General procedure A. The round-bottom flask was charged by magnetic stirrer, corresponding *N*-alkylated nitro derivative (1.0 equiv.), ammonium chloride (2.0 equiv.), iron (2.0 equiv.) followed by ethanol/water (10/1, *v*/*v*, 0.25 M with respect to nitro derivative). The resulting suspension was heated up to reflux (oil bath), and left to stir at this temperature overnight. Once the starting nitro derivative was no longer detected (by TLC), the reaction was cooled to room temperature, filtered thought short pad of Celite (washed with EtOAc). Resulting organic phase of filtrate was separated and water phase was extracted with EtOAc (3 × 100 ml per 10 mmol of starting material). The collected organic phases were washed with brine (1 × 100 ml) and dried under anhydrous $MgSO_4$. After filtration of the solid, the filtrate was concentrated under reduced pressure. The crude product was purified by column chromatography (eluting by hexane/EtOAc).

General procedure C: Substitution/Smiles rearrangement of heterocyclic hydroxy derivatives
The round-bottom flask was charged by magnetic stirrer, corresponding heterocyclic hydroxy derivative (1.0 equiv.) potassium carbonate (3.0 equiv.), potassium iodide (0.1 equiv.), followed by DMSO (0.13M with respect to hydroxy derivative). Then, 2-bromopropionamide (3.0 equiv.) was added in one portion with stirring to and heated up to 70 °C (oil bath). Once the starting hydroxy derivative was no longer detected (by TLC, usually after 2 hours), potassium hydroxide (4.0 equiv.) was added in one portion and mixture was heated up to 150 °C (oil bath). At this temperature, mixture was left to stir overnight. Once the substitution intermediate was no longer detected (by TLC), the reaction was cooled to room temperature, diluted with water (200 ml per 10 mmol of starting hydroxy derivative). Resulting mixture was extracted with EtOAc (3 × 100 ml per 10 mmol of starting material). The collected organic phases were washed with brine (2 × 50 ml per 10 mmol of starting material), and dried under anhydrous $MgSO_4$. After filtration of the solid, the filtrate was concentrated under reduced pressure. The crude product was purified by column chromatography (eluting by hexane/EtOAc).

Characterization data of amino derivatives

1*H*-Indol-4-amine (1a)

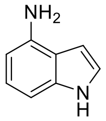

The title compound was synthesized according to the second part (Pd-mediated hydrogenation) of general procedure A, using 4-nitroindole (2.0 g, 12.3 mmol). The product was purified by column chromatography (hexane/EtOAc - 2:1), affording **1a** (1.5 g, 93%) as a grey amorphous solid.

^{1}H NMR (400 MHz, dimethylsulfoxide-d_6): δ 10.70 (s, 1H), 7.07 (q, J = 2.5 Hz, 1H), 6.82 – 6.73 (m, 1H), 6.61 (dd, J = 8.1, 3.1 Hz, 1H), 6.54 – 6.45 (m, 1H), 6.19 – 6.10 (m, 1H), 5.09 (s, 2H) ppm. ^{13}C{^{1}H} NMR (101 MHz, dimethylsulfoxide-d_6): δ 141.2, 136.9, 122.2, 121.8, 116.8, 101.5, 100.0, 99.0 ppm. HRMS (ESI+) m/z: calcd. for $C_8H_9N_2$ $[M + H]^+$: 133.0760, found: 133.0758. Our physical and spectroscopic data matched previously reported data.[1]

1-Methyl-1*H*-indol-4-amine (1b)

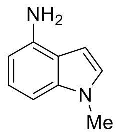

The title compound was synthesized according to the general procedure A, using 4-nitroindole (5.0 g, 30.8 mmol) and iodomethane (2.1 ml, 33.9 mmol) as an alkylating agent. The product was purified by column chromatography (hexane/EtOAc - 2:1 to 1:1), affording **1b** (3.6 g, 79%, over two steps) as a brown amorphous solid.

^{1}H NMR (400 MHz, chloroform-*d*): δ 7.08 (t, *J* = 7.8 Hz, 1H), 6.98 (d, *J* = 3.2 Hz, 1H), 6.83 (dd, *J* = 8.2, 0.9 Hz, 1H), 6.49 – 6.37 (m, 2H), 4.04 (br s, 2H), 3.76 (s, 3H) ppm. ^{13}C{^{1}H} NMR (101 MHz, chloroform-*d*): δ 139.4, 137.9, 127.1, 122.9, 117.9, 104.0, 100.6, 97.3, 33.2 ppm. HRMS (ESI+) *m*/*z*: calcd. for $C_9H_{11}N_2$ $[M + H]^+$: 147.0917, found: 147.0918. Our physical and spectroscopic data matched previously reported data.[2]

1-Propyl-1*H*-indol-4-amine (1c)

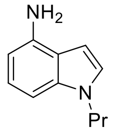

The title compound was synthesized according to the general procedure A, using 4-nitroindole (1000 mg, 6.2 mmol) and 1-iodopropane (0.7 ml, 6.8 mmol) as an alkylating agent. The product was purified by column chromatography (hexane/EtOAc - 3:1), affording **1c** (870 mg, 81%, over two steps) as a dark reddish oil.

^{1}H NMR (400 MHz, chloroform-*d*): δ 7.49 (dd, *J* = 10.2, 5.5 Hz, 1H), 7.28 (t, *J* = 3.0 Hz, 1H), 7.21 (dd, *J* = 8.4, 3.5 Hz, 1H), 6.81 – 6.69 (m, 2H), 4.27 – 4.21 (m, 2H), 4.21 (br s, 2H), 2.11 (hd, *J* = 7.2, 2.0 Hz, 2H), 1.21 (td, *J* = 7.4, 2.4 Hz, 3H) ppm. ^{13}C{^{1}H} NMR (101 MHz, chloroform-*d*): δ 139.5, 136.7, 125.6, 122.2, 117.5, 103.1, 100.1, 96.8, 47.5, 22.9, 11.0 ppm. IR (ATR): ν = 3354 (N-H, amine) cm^{-1}. HRMS (ESI+) *m*/*z*: calcd. for $C_{11}H_{15}N_2$ $[M + H]^+$: 175.1230, found: 175.1229.

1-Allyl-1*H*-indol-4-amine (1d)

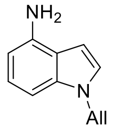

The title compound was synthesized according to the general procedure B, using 4-nitroindole (1000 mg, 6.2 mmol) and allyl bromide (0.6 ml, 6.8 mmol) as an alkylating agent. The product was purified by column chromatography (hexane/EtOAc - 2:1), affording **1d** (231 mg, 22%, over two steps) as a dark brown oil.

^{1}H NMR (400 MHz, chloroform-*d*): δ 7.07 (dd, *J* = 8.2, 7.5 Hz, 1H), 7.03 (d, *J* = 3.2 Hz, 1H), 6.83 (dt, *J* = 8.3, 0.9 Hz, 1H), 6.47 (dd, *J* = 3.2, 0.9 Hz, 1H), 6.44 (dd, *J* = 7.5, 0.8 Hz, 1H), 6.02 (ddt, *J* = 17.1, 10.5, 5.4 Hz, 1H), 5.22 (dq, *J* = 10.2, 1.5 Hz, 1H), 5.12 (dq, *J* = 17.1, 1.7 Hz, 1H), 4.70 (dt, *J* = 5.4, 1.7 Hz, 2H), 3.95 (br s, 2H) ppm. ^{13}C{^{1}H} NMR (101 MHz, chloroform-*d*): δ 139.6, 137.3, 133.7, 126.1, 122.9, 118.1, 117.3, 104.1, 101.0, 97.8, 49.1 ppm. IR (ATR): ν = 3352 (N-H, amine) cm^{-1}. HRMS (ESI+) *m*/*z*: calcd. for $C_{11}H_{13}N_2$ $[M + H]^+$: 173.1073, found: 173.1072.

1-Benzyl-1*H*-indol-4-amine (1e)

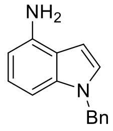

The title compound was synthesized according to the general procedure B, using 4-nitroindole (1000 mg, 6.2 mmol) and benzyl bromide (0.8 ml, 6.8 mmol) as an alkylating agent. The product was purified by column chromatography (hexane/EtOAc - 3:1), affording **1e** (433 mg, 32%, over two steps) as a brown amorphous solid.

^{1}H NMR (400 MHz, chloroform-*d*): δ 7.37 (dddd, *J* = 8.6, 6.8, 4.8, 1.8 Hz, 3H), 7.23 – 7.17 (m, 2H), 7.16 – 7.08 (m, 2H), 6.87 (d, *J* = 8.3 Hz, 1H), 6.57 (dd, *J* = 3.3, 0.9 Hz, 1H), 6.53 – 6.48 (m, 1H), 5.32 (s, 2H), 3.99 (s, 2H) ppm. ^{13}C{^{1}H} NMR (101 MHz, chloroform-*d*): δ 139.6, 137.7, 137.5, 128.7 (2C), 127.5, 126.8 (2C), 126.4, 123.0, 117.9, 104.0, 100.9, 98.0, 50.1 ppm.

HRMS (ESI+) *m/z*: calcd. for $C_{15}H_{15}N_2$ [M + H]$^+$: 223.1230, found: 223.1231. Our physical and spectroscopic data matched previously reported data.[3]

1,3-Dimethyl-1*H*-indol-4-amine (1f)

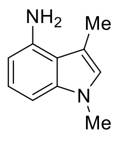

The title compound was synthesized according to the general procedure B, using 3-methyl-4-nitro-1*H*-indole[4] (300 mg, 1.7 mmol) and iodomethane (0.6 ml, 1.9 mmol) as an alkylating agent. The product was purified by column chromatography (hexane/EtOAc - 6:1), affording **1f** (135 mg, 50%, over two steps) as a purple amorphous solid.

^{1}H NMR (400 MHz, chloroform-*d*): δ 7.00 (t, J = 7.8 Hz, 1H), 6.72 (d, J = 8.1 Hz, 1H), 6.66 (q, J = 1.2 Hz, 1H), 6.32 (ddd, J = 7.5, 2.0, 1.0 Hz, 1H), 4.13 (br s, 2H), 3.65 (s, 3H), 2.56 (d, J = 1.1 Hz, 3H) ppm. ^{13}C{^{1}H} NMR (101 MHz, chloroform-*d*): δ 141.3, 139.0, 125.4, 122.9, 117.1, 109.3, 104.2, 100.6, 32.7, 12.7 ppm. IR (ATR): ν = 3371 (N-H, amine) cm^{-1}. HRMS (ESI+) *m/z*: calcd. for $C_{10}H_{13}N_2$ [M + H]$^+$: 161.1073, found: 161.1072.

***N*-Benzyl-1-methyl-1*H*-indol-4-amine (1g)**

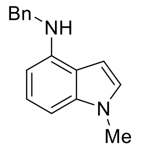

Benzaldehyde (0.17 ml, 1.6 mmol, 1.2 equiv.) was added in one portion to a stirred solution of aminoindole **1b** (200 mg, 1.4 mmol, 1.0 equiv.), and $ZnCl_2$ (224 mg, 1.6 mmol, 1.2 equiv.) in MeOH (20 ml) at room temperature. Then, $NaBH_3CN$ (103 mg, 1.6 mmol, 1.2 equiv.) was added portionwise (gas evolution). Once the starting amino derivative was no longer detected (by TLC, 15 minutes), the reaction was quenched by the addition of an aqueous solution of sodium hydroxide (1M, 50 ml) and diluted with EtOAc (30 ml). The organic phase was separated and the water phase was extracted with EtOAc (3 × 30 ml). Collected organic phases were mixed with an aqueous saturated solution of sodium bisulfite (50 ml) followed by intensive stirring for 30 minutes at room temperature (by this treatment residue of benzaldehyde was removed). The organic phase was separated and washed with water (1 × 30 ml), followed by brine (1 × 30 ml), and dried under $MgSO_4$. Solids were filtered, and solvents were evaporated. The crude product was purified by column chromatography (hexane/EtOAc - 11:1), affording **1g** (278 mg, 86%) as a white amorphous solid.

^{1}H NMR (400 MHz, chloroform-*d*): δ 7.52 – 7.45 (m, 2H), 7.43 – 7.36 (m, 2H), 7.35 – 7.30 (m, 1H), 7.14 (t, J = 7.9 Hz, 1H), 6.98 (d, J = 3.2 Hz, 1H), 6.82 (dd, J = 8.2, 0.9 Hz, 1H), 6.44 (dd, J = 3.1, 0.9 Hz, 1H), 6.36 (d, J = 7.6 Hz, 1H), 4.54 (s + br s, 2 + 1H), 3.78 (s, 3H) ppm. ^{13}C{^{1}H} NMR (101 MHz, chloroform-*d*): δ 141.3, 139.8, 137.5, 128.7 (2C), 127.8 (2C), 127.3, 126.7, 123.1, 117.3, 100.0, 99.7, 97.1, 48.6, 33.1 ppm. HRMS (ESI+) *m/z*: calcd. for $C_{16}H_{17}N_2$ [M + H]$^+$: 237.1386, found: 237.1387. Our physical and spectroscopic data matched previously reported data.[5]

4-Methyl-*N*-(1-methyl-1*H*-indol-4-yl)benzenesulfonamide (1h)

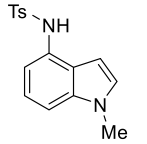

Tosyl chloride (516 mg, 2.7 mmol, 1.2 equiv.) was added in one portion to a stirred solution of aminoindole **1b** (330 mg, 2.3 mmol, 1.0 equiv.) in anhydrous pyridine (8 ml) at room temperature. The reaction mixture was heated up to 65 °C (oil bath). Once the starting amino derivative was no longer detected (by TLC, 2 hours), the residual pyridine was evaporated. The resulting residue was dissolved in DCM (50 ml) and washed with diluted hydrochloric acid (1M, 50 ml). Organic phase was separated and the water phase was extracted with DCM (3 × 30 ml). Collected organic phases were washed with water (1 × 30 ml), brine (1 × 30 ml), and dried under $MgSO_4$. Solids were filtered, and solvents were evaporated. The crude product was purified by column chromatography (hexane/EtOAc - 3:1 to 1:1), affording **1h** (352 mg, 52%) as a white amorphous solid.

^{1}H NMR (400 MHz, chloroform-*d*): δ 7.71 (d, *J* = 8.3 Hz, 2H), 7.17 – 7.13 (m, 2H), 7.11 – 7.04 (m, 3H), 7.03 – 6.97 (m, 1H), 6.95 (d, *J* = 3.0 Hz, 1H), 6.37 (d, *J* = 3.2 Hz, 1H), 3.72 (s, 3H), 2.32 (s, 3H) ppm. ^{13}C{^{1}H} NMR (101 MHz, chloroform-*d*): δ 143.6, 137.7, 136.7, 129.6 (2C), 129.0, 128.7, 127.4 (2C), 122.6, 122.1, 112.4, 107.0, 97.5, 33.2, 21.6 ppm. IR (ATR): ν = 1342, 1153 (S=O, sulfonamide) cm^{-1}. HRMS (ESI+) *m*/*z*: calcd. for $C_{16}H_{17}N_2O_2S$ [M + H]$^+$: 301.1005, found: 301.1009.

tert-Butyl 4-amino-1*H*-indole-1-carboxylate (1i)

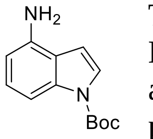

The title compound was synthesized according to the modified general procedure B, using 4-nitroindole (1000 mg, 6.2 mmol), Boc_2O (1.3 g, 12.3 mmol, 2.0 equiv.), and DMAP (113 mg, 0.92 mmol, 0.15 equiv.) instead of sodium hydride. The product was purified by column chromatography (hexane/EtOAc - 4:1), affording **1i** (377 mg, 26%, over two steps) as a brown amorphous solid.

^{1}H NMR (400 MHz, chloroform-*d*): δ 7.71 (d, *J* = 8.4 Hz, 1H), 7.58 (d, *J* = 3.9 Hz, 1H), 7.21 (t, *J* = 8.0 Hz, 1H), 6.61 – 6.49 (m, 2H), 3.97 (s, 2H), 1.73 (s, 9H) ppm. ^{13}C{^{1}H} NMR (101 MHz, chloroform-*d*): δ 150.0, 139.0, 136.4, 125.5, 124.4, 119.3, 108.1, 106.6, 103.5, 83.7, 28.3 (3C) ppm. HRMS (ESI+) *m*/*z*: calcd. for $C_{13}H_{17}N_2O_2$ [M + H]$^+$: 233.1285, found: 233.1289. Our physical and spectroscopic data matched previously reported data.[6]

1-Tosyl-1*H*-indol-4-amine (1j)

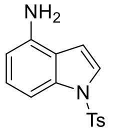

The title compound was synthesized according to the general procedure B, using 4-nitroindole (1000 mg, 6.2 mmol) and tosyl chloride (1.3 g, 6.8 mmol) as an alkylating agent. The product was purified by column chromatography (hexane/EtOAc – 4:1 to 2:1), affording **1j** (516 mg, 29%, over two steps) as a brown amorphous solid.

^{1}H NMR (400 MHz, dimethylsulfoxide-*d*$_6$): δ 7.84 – 7.73 (m, 2H), 7.51 (d, *J* = 3.6 Hz, 1H), 7.35 (d, *J* = 7.9 Hz, 2H), 7.07 (dd, *J* = 8.3, 2.2 Hz, 1H), 6.97 (td, *J* = 7.9, 1.6 Hz, 1H), 6.93 (t, *J* = 2.9 Hz, 1H), 6.35 (dd, *J* = 7.8, 1.7 Hz, 1H), 5.55 (br s, 2H), 2.30 (s, 3H) ppm. ^{13}C{^{1}H} NMR (101 MHz, dimethylsulfoxide-*d*$_6$): δ 145.1, 142.3, 135.4, 134.3, 130.0 (2C), 126.6 (2C), 125.9, 123.5, 117.9, 107.4, 106.4, 100.8, 21.0 ppm. HRMS (ESI+) *m*/*z*: calcd. for $C_{15}H_{15}N_2O_2S$ [M + H]$^+$: 287.0849, found: 287.0852. Our physical and spectroscopic data matched previously reported data.[7]

1-Methyl-1*H*-indol-5-amine (1k)

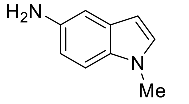

The title compound was synthesized according to the general procedure A, using 5-nitroindole (1000 mg, 6.2 mmol) and iodomethane (0.42 ml, 6.8 mmol) as an alkylating agent. The product was purified by column chromatography (hexane/EtOAc - 2:1 to 1:1), affording **1k** (224 mg, 25%, over two steps) as a brown oil.

^{1}H NMR (400 MHz, chloroform-*d*): δ 7.13 (d, *J* = 8.6 Hz, 1H), 6.97 (d, *J* = 3.0 Hz, 1H), 6.94 (d, *J* = 2.2 Hz, 1H), 6.70 (dd, *J* = 8.5, 2.2 Hz, 1H), 6.30 (dd, *J* = 3.0, 0.9 Hz, 1H), 3.73 (s, 3H), 3.49 (br s, 2H) ppm. ^{13}C{^{1}H} NMR (101 MHz, chloroform-*d*): δ 139.4, 132.0, 129.5, 129.3, 112.6, 109.9, 105.9, 99.7, 33.0 ppm. HRMS (ESI+) *m*/*z*: calcd. for $C_9H_{11}N_2$ [M + H]$^+$: 147.0917, found: 147.0918. Our physical and spectroscopic data matched previously reported data.[8]

1-Methyl-1*H*-indol-6-amine (1l)

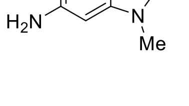

The title compound was synthesized according to the general procedure A, using 6-nitroindole (1000 mg, 6.2 mmol) and iodomethane (0.42 ml, 6.8 mmol) as an alkylating agent. The product was purified by column chromatography

(hexane/EtOAc - 2:1 to 1:1), affording **1l** (205 mg, 23%, over two steps) as a dark brown amorphous solid.
^{1}H NMR (400 MHz, chloroform-*d*): δ 7.44 (dd, *J* = 8.2, 0.8 Hz, 1H), 6.89 (d, *J* = 3.1 Hz, 1H), 6.62 (dt, *J* = 1.8, 0.8 Hz, 1H), 6.60 (dd, *J* = 8.2, 2.0 Hz, 1H), 6.41 (dd, *J* = 3.2, 0.9 Hz, 1H), 3.68 (s, 3H), 3.62 (br s, 2H) ppm. ^{13}C{^{1}H} NMR (101 MHz, chloroform-*d*): δ 141.9, 138.1, 127.0, 122.0, 121.6, 110.4, 100.9, 95.0, 32.8 ppm. HRMS (ESI+) *m/z*: calcd. for $C_9H_{11}N_2$ $[M + H]^+$: 147.0917, found: 147.0918. Our physical and spectroscopic data matched previously reported data.[9]

1-Methyl-1*H*-indol-7-amine (1m)

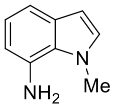

The title compound was synthesized according to the general procedure A, using 7-nitroindole (1000 mg, 6.2 mmol) and iodomethane (0.42 ml, 6.8 mmol) as an alkylating agent. The product was purified by column chromatography (hexane/EtOAc - 2:1 to 1:1), affording **1m** (178 mg, 20%, over two steps) as grey amorphous solid.
^{1}H NMR (400 MHz, chloroform-*d*): δ 7.13 (dd, *J* = 7.9, 1.0 Hz, 1H), 6.92 – 6.85 (m, 2H), 6.49 (dd, *J* = 7.4, 1.1 Hz, 1H), 6.39 (d, *J* = 3.1 Hz, 1H), 4.12 (s, 3H), 3.87 (br s, 2H) ppm. ^{13}C{^{1}H} NMR (101 MHz, chloroform-*d*): δ 132.4, 131.1, 130.4, 127.5, 120.4, 113.5, 110.1, 101.3, 36.5 ppm. HRMS (ESI+) *m/z*: calcd. for $C_9H_{11}N_2$ $[M + H]^+$: 147.0917, found: 147.0918. Our physical and spectroscopic data matched previously reported data.[10]

Benzofuran-4-amine (1n)

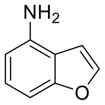

The title compound was synthesized according to the general procedure C, using benzo[*b*]thiophen-4-ol[11] (2.0 g, 14.7 mmol). The product was purified by column chromatography (hexane/EtOAc - 4:1), affording **1n** (730 mg, 37%, over two steps) as a brown oil.
^{1}H NMR (400 MHz, chloroform-*d*): δ 7.54 (d, *J* = 2.3 Hz, 1H), 7.11 (t, *J* = 8.0 Hz, 1H), 6.98 (dt, *J* = 8.4, 0.9 Hz, 1H), 6.70 (dd, *J* = 2.3, 1.0 Hz, 1H), 6.52 (dd, *J* = 7.6, 0.8 Hz, 1H), 3.93 (br s, 2H) ppm. ^{13}C{^{1}H} NMR (101 MHz, chloroform-*d*): δ 156.3, 143.3, 140.1, 125.4, 116.0, 107.6, 103.3, 102.4 ppm. HRMS (ESI+) *m/z*: calcd. for C_8H_8NO $[M + H]^+$: 134.0600, found: 134.0602. Our physical and spectroscopic data matched previously reported data.[11]

Benzo[*b*]thiophen-4-amine (1o)

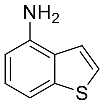

The title compound was synthesized according to the general procedure C, using benzo[*b*]thiophen-4-ol[11] (1.50 g, 10.0 mmol). The product was purified by column chromatography (hexane/EtOAc - 4:1), affording **1o** (760 mg, 51%, over two steps) as a brown amorphous solid.
^{1}H NMR (400 MHz, chloroform-*d*): δ 7.40 – 7.31 (m, 2H), 7.28 (dd, *J* = 5.5, 0.9 Hz, 1H), 7.17 (t, *J* = 7.8 Hz, 1H), 6.64 (dd, *J* = 7.6, 0.8 Hz, 1H), 4.03 (br s, 2H) ppm. ^{13}C{^{1}H} NMR (101 MHz, chloroform-*d*): δ 141.6, 141.2, 128.4, 125.6, 124.5, 119.4, 113.1, 109.0 ppm. HRMS (ESI+) *m/z*: calcd. for C_8H_8NS $[M + H]^+$: 150.0372, found: 150.0374. Our physical and spectroscopic data matched previously reported data.[11]

Annulation reaction

Complete reaction optimization survey

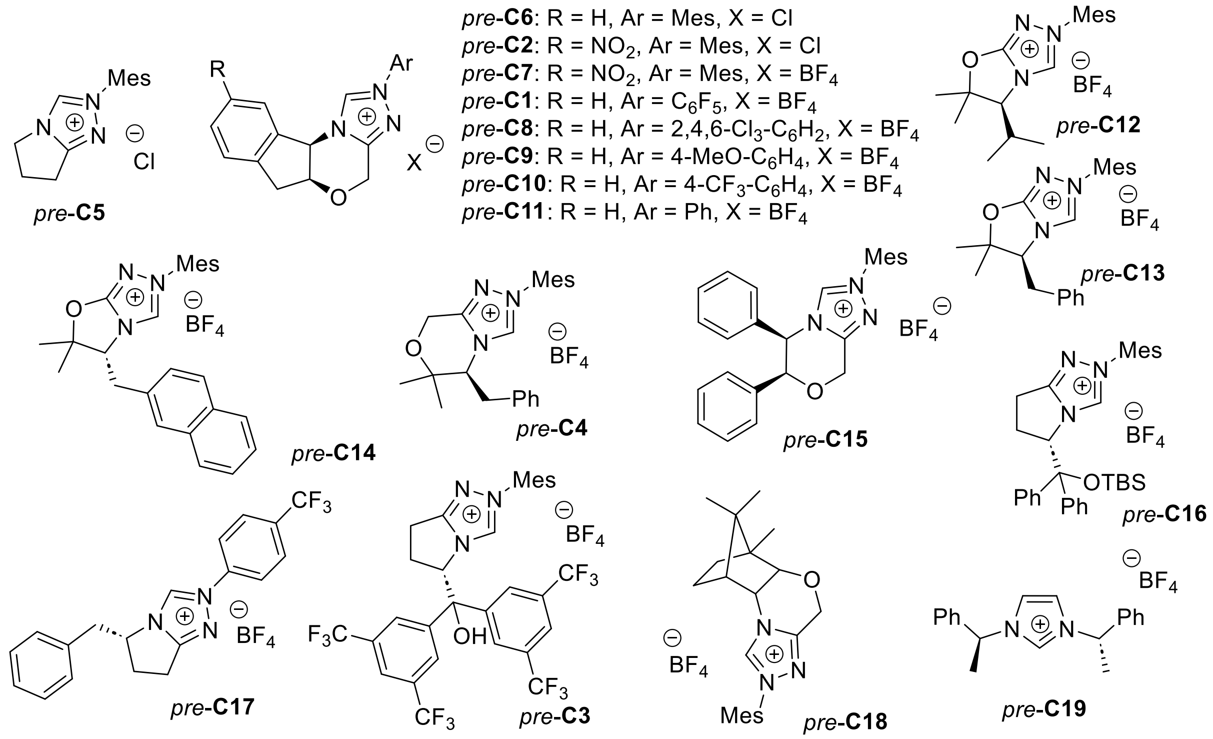

Figure S1. Screened precursors

Table S1. Precursor screening

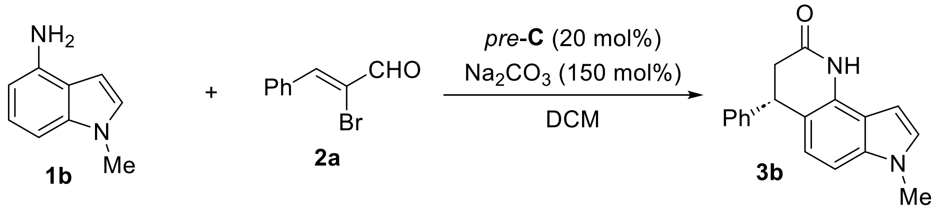

Entry[a]	*pre*-**C**	Time (h)	Conversion[b]	Yield (%)[c]	*er* (%)[d]
1	*pre*-**C5**	2	full	47	50:50
2	*pre*-**C6**	2	full	79	88:12
3	*pre*-**C2**	24	full	86	96:4
4	*pre*-**C7**	24	full	97	94:6
5	*pre*-**C1**	2	full	61	32:68
6	*pre*-**C8**	2	full	85	33:67
7	*pre*-**C9**	24	full	61	72:28
8	*pre*-**C10**	24	full	52	71:29
9	*pre*-**C11**	24	full	72	71:29
10	*pre*-**C12**	24	full	75	31:69
11	*pre*-**C13**	24	full	68	40:60
12	*pre*-**C14**	24	full	71	50:50
13	*pre*-**C4**	24	not full[e]	67	18:82
14	*pre*-**C15**	24	full	75	50:50
15	*pre*-**C16**	72	no	-	-
16	*pre*-**C17**	24	not full[e]	51	44:56
17	*pre*-**C3**	24	not full[e]	58	96:4
18	*pre*-**C18**	24	not full[e]	50	67:33
19	*pre*-**C19**	72	no	-	-

[a] Reactions were conducted with **1b** (0.2 mmol), **2a** (0.3 mmol), Na_2CO_3 (0.15 mmol), and *pre*-**Catalyst** (20 mol%) in DCM (1.0 ml) at room temperature. [b] Determined by TLC of crude mixture, conversion of **1b**. [c] Isolated yield after column chromatography. [d] Determined by chiral HPLC analysis. [e] Aldehyde disappeared. *Er* - enantiomeric ratio.

Table S2. Base screening

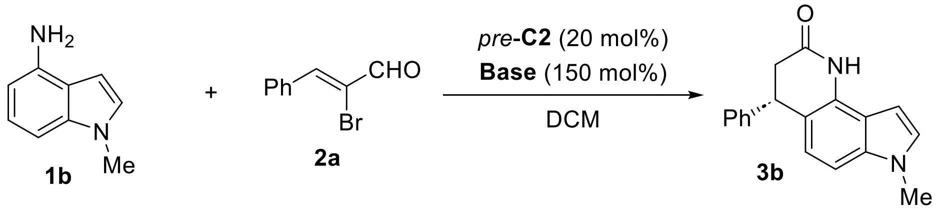

Entry[a]	Base	Time (h)	Conversion[b]	Yield (%)[c]	*er* (%)[d]
1	Na_2CO_3	24	full	86	96:4
2	$NaHCO_3$	24	not full[e]	26	95:5
3	Cs_2CO_3	72	not full	19	95:5
4	K_2CO_3	24	full	39	96:4
5	K_3PO_4	24	full	53	96:4
6	KO*t*Bu	72	no	-	-
7	AcONa	24	not full[e]	n.d.[f]	95:5
8	TEA	24	not full[e]	n.d.[f]	94:6
9	DIPEA	24	not full[e]	n.d.[f]	93:7
10	DABCO	24	not full[e]	n.d.[f]	96:4
11	DBU	72	no	-	-
12	pyridine	72	not full	traces	-
13	2,6-lutidine	24	full	50	95:5

[a] Reactions were conducted with **1b** (0.2 mmol), **2a** (0.3 mmol), selected base (0.15 mmol), and *pre*-**C2** (20 mol%) in DCM (1.0 ml) at room temperature. [b] Determined by TLC of crude mixture, conversion of **1b**. [c] Isolated yield after column chromatography. [d] Determined by chiral HPLC analysis. [e] Aldehyde disappeared. [f] Contamined by cinnamic acid. *Er* - enantiomeric ratio.

Table S3. Solvent screening

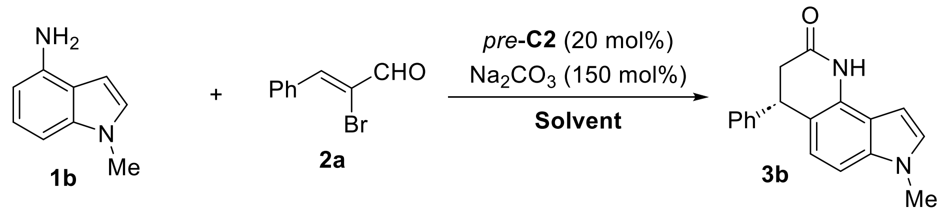

Entry[a]	Solvent	Time (h)	Conversion[b]	Yield (%)[c]	*er* (%)[d]
1	DCM	24	full	86	96:4
2	$CHCl_3$	15	full	73	97:3
3	1,2-DCE	15	full	79	90:10
4	CCl_4	24	full	58	96:4
5	benzene	72	not full[e]	66	98:2
6	toluene	72	not full[e]	62	97:3
7	MeCN	15	full	90	80:20
8	EtOAc	48	not full[e]	70	97:3
9	acetone	15	not full[e]	51	90:10
10	MTBE	48	full	36	95:5
11	THF	15	not full[e]	61	93:3
12	DMF	72	not full	29	83:17
13	heptane	72	not full	complex mixture	-

[a] Reactions were conducted with **1b** (0.2 mmol), **2a** (0.3 mmol), Na_2CO_3 (0.15 mmol), and *pre*-**C2** (20 mol%) in selected solvent (1.0 ml) at room temperature. [b] Determined by TLC of crude mixture, conversion of **1b**. [c] Isolated yield after column chromatography. [d] Determined by chiral HPLC analysis. [e] Aldehyde disappeared. *Er* - enantiomeric ratio.

Table S4. Ratio screening

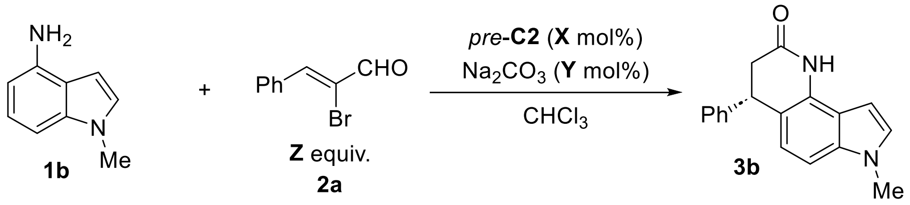

Entry[a]	X	Y	Z	Time (h)	Conversion[b]	Yield (%)[c]	*er* (%)[d]
1	20	150	1.5	15	full	73	97:3
2	15	150	1.5	15	full	78	97:3
3	10	150	1.5	15	full	74	97:3
4	5	150	1.5	15	full	80	97:3
5	2.5	150	1.5	15	full	88	97:3
6	1	150	1.5	15	almost full	87	97:3
7	1	100	1.5	48	full	87	97:3
8	1	200	1.5	15	full	84	97:3
9	1	200	2.0	48	full	85	97:3
10	1	200	1.2	15	not full[e]	87	97:3
11	1	200	1.0	15	not full[e]	76	97:3

[a] Reactions were conducted with **1b** (0.2 mmol), **2a** (**Z** mmol), Na_2CO_3 (**Y** mmol), and *pre*-**C2** (**X** mol%) in chloroform (1.0 ml) at room temperature. [b] Determined by TLC of crude mixture, conversion of **1b**.
[c] Isolated yield after column chromatography. [d] Determined by chiral HPLC analysis. [e] Aldehyde disappeared.
Er - enantiomeric ratio.

Table S5. Additive screening

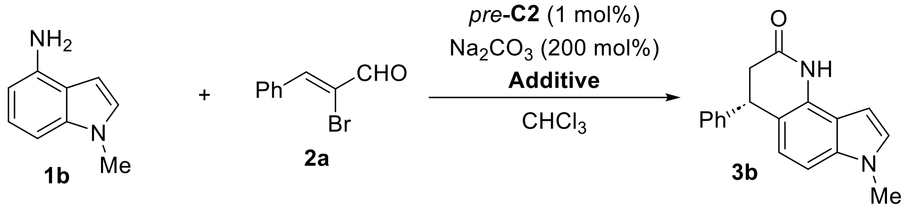

Entry[a]	Additive	Time (h)	Conversion[b]	Yield (%)[c]	*er* (%)[d]
1	none	15	full	84	97:3
2	MS (3Å, 50 mg)	72	full	73	97:3
3	MS (4Å, 50 mg)	72	not full	59	97:3
4	MS (5Å, 50 mg)	72	not full	41	97:3
5	H_2O (50 mol%)	15	full	90	97:3
6	Schreiner THU (20 mol%)	72	not full	40	61:39
7	LiCl (150 mol%)	72	not full[e]	27	97:3
8[f]	$Yb(OTf)_3$ (20 mol%)	15	full	17[g]	96:4

[a] Conducted with **1b** (0.2 mmol), **2a** (0.3 mmol), Na_2CO_3 (0.2 mmol), selected additive, and *pre*-**C2** (1 mol%) in chloroform (1.0 ml) at room temperature. [b] Determined by TLC of crude mixture, conversion of **1b**. [c] Isolated yield after column chromatography. [d] Determined by chiral HPLC analysis.
[e] Aldehyde disappeared. [f] Aminoindole **1a** was used instead of **1b**, yielding product **3a**. [g] The product was contaminated by amidation product **4a** and another undefined by-product (approx. 20%, HPLC).
Er - enantiomeric ratio, Schreiner THU = 1,3-*Bis*[3,5-*bis*(trifluoromethyl)phenyl]thiourea.

Table S6. Temperature and concentration screening

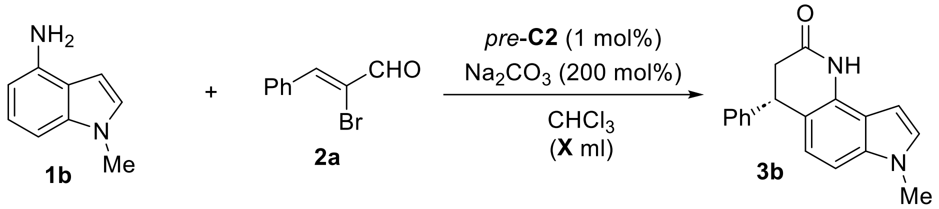

Entry[a]	X	Temperature	Time (h)	Conversion[b]	Yield (%)[c]	*er* (%)[d]
1	1.0	r.t.	15	full	84	97:3
2	2.0	r.t.	24	full	65	98:2
3	0.5	r.t.	15	full	87	97:3
4	1.0	40 °C	9	full	98	96:4
5	1.0	0 °C	72	not full	74	98:2

[a] Reactions were conducted with **1b** (0.2 mmol), **2a** (0.3 mmol), Na_2CO_3 (0.2 mmol), and *pre*-**C2** (1 mol%) in chloroform (**X** ml) at selected room temperature. [b] Determined by TLC of crude mixture, conversion of **1b**. [c] Isolated yield after column chromatography. [d] Determined by chiral HPLC analysis. *Er* - enantiomeric ratio,

Table S7. Oxidative approach.

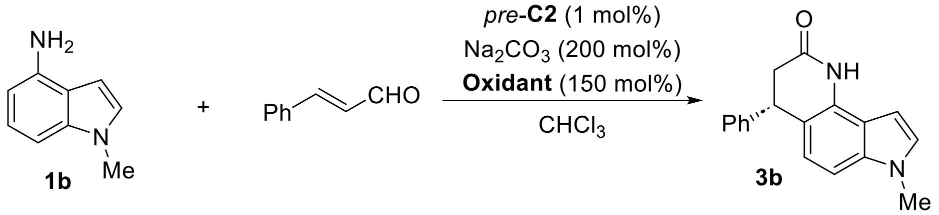

Entry[a]	Oxidant	Time (h)	Conversion[b]	Yield (%)[c]	*er* (%)[d]
1	DQ	72	not full	traces	-
2	MnO_2	72	not full	traces	-

[a] Reactions were conducted with **1b** (0.2 mmol), *trans*-cinnamaldehyde (0.3 mmol), Na_2CO_3 (0.2 mmol), selected oxidant (0.3 mmol), and *pre*-**C2** (1 mol%) in chloroform (1.0 ml) at room temperature. [b] Determined by TLC of crude mixture, conversion of **1b**. [c] Isolated yield after column chromatography. [d] Determined by chiral HPLC analysis. *Er* - enantiomeric ratio.

General procedure for annulation reaction

The vial (4 ml) was charged with magnetic stirrer, corresponding aminoderivative **1** (0.2 mmol, 1.0 equiv.), corresponding α-bromocinnamic aldehyde **2**[12] (0.3 mmol, 1.5 equiv.), Na_2CO_3 (42.2 mg, 0.4 mmol, 2.0 equiv.), *pre*-**C2** (0.8 mg, 0.002 mmol, 0.01 equiv.), followed by chloroform (1.0 ml) at room temperature. The reaction was stirred for the indicated time at room temperature. Once the reaction was completed by thin-layer chromatography (TLC), the solvent was evaporated. The crude product was purified by column chromatography (eluting with hexane/EtOAc mixtures).
Note: Racemic samples were prepared in reactions with *pre*-**C5** (20 mol%).

Characterization data of products

(*S*)-4-Phenyl-1,3,4,7-tetrahydro-2*H*-pyrrolo[2,3-*h*]quinolin-2-one (3a)

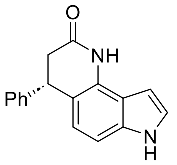

The title compound was synthesized according to the general procedure (reaction time: 15 hours), using aminoindole **1a** (26.4 mg, 0.2 mmol) and (*Z*)-2-bromo-3-phenylacrylaldehyde (63.3 mg, 0.3 mmol). The product was purified by column chromatography (hexane/EtOAc - 2:1 to 1:1), affording **3a** (15 mg, 29%) as a brown amorphous solid. Purity ~ 90% (HPLC, NMR, contamided by amidation product **4a**).
Er = 94:6 (*ee* = 89%), the enantiomeric excess of product **3a** was determined by HPLC using a Chiralpak® IB column (*n*-heptane/*i*-PrOH - 80:20, flow rate = 1.0 ml/min, λ = 252 nm, t = 25 °C): t_R = 13.0 min (minor), t_R = 15.6 min (major). $[\alpha]_D^{20}$ = +38.5 (c = 0.3, MeOH). ^{1}H NMR (400 MHz, chloroform-*d*): δ 8.98 (br s, 1H), 8.34 (br s, 1H), 7.36 – 7.29 (m, 2H), 7.27 – 7.20 (m, 4H), 7.05 (dd, J = 8.3, 1.0 Hz, 1H), 6.82 (d, J = 8.3 Hz, 1H), 6.70 (ddd, J = 3.2, 2.1, 1.0 Hz, 1H), 4.43 (t, J = 6.9 Hz, 1H), 3.16 – 2.91 (m, 2H) ppm. $^{13}C\{^1H\}$ NMR (101 MHz, chloroform-*d*): δ 171.0, 143.0, 136.2, 128.9 (2C), 128.8, 127.9 (2C), 127.1, 124.7, 122.8, 117.1, 116.1, 106.3, 98.5, 42.1, 39.4 ppm. IR (ATR): ν = 3390 (N-H, indole), 3205 (N-H, amide), 1651 (C=O, amide) cm^{-1}. HRMS (ESI+) *m*/*z*: calcd. for $C_{17}H_{15}N_2O$ $[M + H]^+$: 263.1179, found: 263.1179.

(*S*)-7-Methyl-4-phenyl-1,3,4,7-tetrahydro-2*H*-pyrrolo[2,3-*h*]quinolin-2-one (3b)

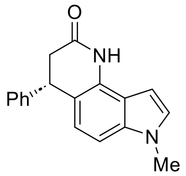

The title compound was synthesized according to the general procedure (reaction time: 15 hours), using aminoindole **1b** (29.2 mg, 0.2 mmol) and (*Z*)-2-bromo-3-phenylacrylaldehyde (63.3 mg, 0.3 mmol). The product was purified by column chromatography (hexane/EtOAc - 2:1 to 1:1), affording **3b** (46 mg, 84%) as a white amorphous solid. Crystals suitable for X-ray analysis were grown by dissolving **3b** in a minimal amount of boiling *i*-PrOH, followed by standing at room temperature overnight.
m.p. = 249.3-250.0 °C (*i*-PrOH). *Er* = 97:3 (*ee* = 94%), the enantiomeric excess of product **3b** was determined by high-performance liquid chromatography (HPLC) using a Chiralpak® IA column (*n*-heptane/*i*-PrOH - 80:20, flow rate = 1.0 ml/min, λ = 310 nm, t = 25 °C): t_R = 12.5 min (minor), t_R = 20.0 min (major). $[\alpha]_D^{20}$ = +87.2 (c = 0.7, $CHCl_3$). ^{1}H NMR (400 MHz, chloroform-*d*): δ 9.94 (s, 1H), 7.14 (d, J = 2.0 Hz, 1H), 7.04 (d, J = 2.0 Hz, 1H), 6.74 (dd, J = 7.8, 2.0 Hz, 1H), 6.58 – 6.52 (m, 2H), 6.47 (d, J = 7.8 Hz, 1H), 4.16 – 4.03 (m, 2H), 3.91 (s, 3H), 3.27 – 3.16 (m, 3H), 3.11 (ddd, J = 13.2, 10.2, 5.7 Hz, 1H), 3.05 – 2.88 (m, 2H) ppm. $^{13}C\{^1H\}$ NMR (101 MHz, chloroform-*d*): δ 171.3, 143.2, 137.1, 129.4, 129.1, 128.9 (2C), 127.9 (2C), 127.0, 122.3, 117.6, 115.6, 104.4, 97.2, 42.1, 39.5, 33.2 ppm. IR (ATR): ν = 3192 (N-H, amide), 1660 (C=O, amide) cm^{-1}. HRMS (ESI+) *m*/*z*: calcd. for $C_{18}H_{17}N_2O$ $[M + H]^+$: 277.1335, found: 277.1336.

(*R*)-7-Methyl-4-phenyl-1,3,4,7-tetrahydro-2*H*-pyrrolo[2,3-*h*]quinolin-2-one (*ent*-3b)

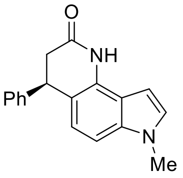

The title compound was synthesized according to the general procedure (reaction time: 15 hours), using aminoindole **1b** (29.2 mg, 0.2 mmol) and (*Z*)-2-bromo-3-phenylacrylaldehyde (63.3 mg, 0.3 mmol), and *ent*-*pre*-**C2** instead of *pre*-**C2**. The product was purified by column chromatography (hexane/EtOAc - 2:1 to 1:1), affording *ent*-**3b** (50 mg, 90%) as a white amorphous solid. Crystals suitable for X-ray analysis were grown by dissolving **3b** in a minimal amount of boiling *i*-PrOH, followed by standing at room temperature overnight.

m.p. = 250.3-251.0 °C (*i*-PrOH). *Er* = 98:2 (*ee* = 96%), the enantiomeric excess of product *ent*-**3b** was determined by HPLC using a Chiralpak® IA column (*n*-heptane/*i*-PrOH - 80:20, flow rate = 1.0 ml/min, λ = 310 nm, *t* = 25 °C): t_R = 12.5 min (major), t_R = 20.0 min (minor). $[\alpha]_D^{20}$ = -69.3 (*c* = 0.5, $CHCl_3$). Other analytical data agree with the data on the opposite enantiomer (**3b**).

(*S*)-4-Phenyl-7-propyl-1,3,4,7-tetrahydro-2*H*-pyrrolo[2,3-*h*]quinolin-2-oneone (3c)

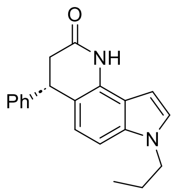

The title compound was synthesized according to the general procedure (reaction time: 15 hours), using aminoindole **1c** (34.8 mg, 0.2 mmol) and (*Z*)-2-bromo-3-phenylacrylaldehyde (63.3 mg, 0.3 mmol). The product was purified by column chromatography (hexane/EtOAc - 3:1), affording **3c** (60 mg, 98%) as a colorless oil.

Er = 97:3 (*ee* = 95%), the enantiomeric excess of product **3c** was determined by HPLC using a Chiralpak® IA column (*n*-heptane/*i*-PrOH - 60:40, flow rate = 1.0 ml/min, λ = 310 nm, *t* = 25 °C): t_R = 8.3 min (minor), t_R = 11.9 min (major). $[\alpha]_D^{20}$ = +64.7 (*c* = 0.7, $CHCl_3$). ^1H NMR (400 MHz, chloroform-*d*): δ 9.24 (br s, 1H), 7.34 – 7.27 (m, 2H), 7.23 (td, *J* = 6.1, 1.6 Hz, 3H), 7.10 (d, *J* = 3.2 Hz, 1H), 6.97 (dd, *J* = 8.4, 0.8 Hz, 1H), 6.80 (d, *J* = 8.4 Hz, 1H), 6.70 (dd, *J* = 3.3, 0.9 Hz, 1H), 4.42 (t, *J* = 6.9 Hz, 1H), 4.05 (t, *J* = 7.0 Hz, 2H), 3.17 – 2.95 (m, 2H), 1.86 (q, *J* = 7.2 Hz, 2H), 0.93 (t, *J* = 7.4 Hz, 3H) ppm. $^{13}C\{^1H\}$ NMR (101 MHz, chloroform-*d*): δ 171.3, 143.2, 136.4, 129.4, 128.9 (2C), 128.3, 127.9 (2C), 127.0, 122.1, 117.7, 115.5, 104.7, 97.0, 48.4, 42.1, 39.5, 23.6, 11.6 ppm. IR (ATR): ν = 3201 (N-H, amide), 1666 (C=O, amide) cm^{-1}. HRMS (ESI+) *m*/*z*: calcd. for $C_{20}H_{21}N_2O$ $[M + H]^+$: 305.1648, found: 305.1650.

(*S*)-7-Allyl-4-phenyl-1,3,4,7-tetrahydro-2*H*-pyrrolo[2,3-*h*]quinolin-2-one (3d)

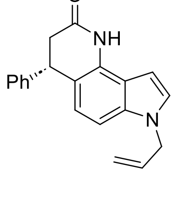

The title compound was synthesized according to the general procedure (reaction time: 15 hours), using aminoindole **1d** (34.5 mg, 0.2 mmol) and (*Z*)-2-bromo-3-phenylacrylaldehyde (63.3 mg, 0.3 mmol). The product was purified by column chromatography (hexane/EtOAc - 3:1 to 2:1), affording **3d** (59 mg, 97%) as a white amorphous solid.

Er = 97:3 (*ee* = 95%), the enantiomeric excess of product **3d** was determined by HPLC using a Chiralpak® IA column (*n*-heptane/*i*-PrOH - 60:40, flow rate = 1.0 ml/min, λ = 222 nm, *t* = 25 °C): t_R = 8.7 min (minor), t_R = 12.3 min (major). $[\alpha]_D^{20}$ = +67.1 (*c* = 2.2, $CHCl_3$). ^1H NMR (400 MHz, chloroform-*d*): δ 9.28 (br s, 1H), 7.30 (dd, *J* = 8.4, 6.2 Hz, 2H), 7.23 (td, *J* = 7.0, 1.7 Hz, 3H), 7.10 (d, *J* = 3.3 Hz, 1H), 6.94 (d, *J* = 8.4 Hz, 1H), 6.80 (d, *J* = 8.4 Hz, 1H), 6.73 (d, *J* = 3.2 Hz, 1H), 5.97 (ddt, *J* = 17.2, 10.5, 5.4 Hz, 1H), 5.20 (dd, *J* = 10.3, 1.4 Hz, 1H), 5.09 (dq, *J* = 17.0, 1.6 Hz, 1H), 4.70 (dt, *J* = 5.5, 1.7 Hz, 2H), 4.41 (t, *J* = 6.9 Hz, 1H), 3.16 – 2.90 (m, 2H) ppm. $^{13}C\{^1H\}$ NMR (101 MHz, chloroform-*d*): δ 171.3, 143.1, 136.5, 133.3, 129.4, 128.9 (2C), 128.2, 127.9 (2C), 127.0, 122.3, 117.8, 117.6, 115.8, 104.8, 97.6, 49.1, 42.1, 39.5 ppm. IR (ATR): ν = 3195 (N-H, amide), 1664 (C=O, amide) cm^{-1}. HRMS (ESI+) *m*/*z*: calcd. for $C_{20}H_{19}N_2O$ $[M + H]^+$: 303.1492, found: 303.1492.

(*S*)-7-Benzyl-4-phenyl-1,3,4,7-tetrahydro-2*H*-pyrrolo[2,3-*h*]quinolin-2-one (3e)

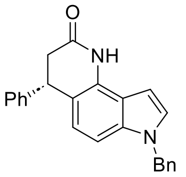

The title compound was synthesized according to the general procedure (reaction time: 15 hours), using aminoindole **1e** (44.5 mg, 0.2 mmol) and (*Z*)-2-bromo-3-phenylacrylaldehyde (63.3 mg, 0.3 mmol). The product was purified by column chromatography (hexane/EtOAc - 3:1 to 2:1), affording **3e** (68 mg, 97%) as a white amorphous solid.

Er = 97:3 (*ee* = 95%), the enantiomeric excess of product **3e** was determined by HPLC using a Chiralpak® IA column (*n*-heptane/*i*-PrOH - 60:40, flow rate = 1.0 ml/min, λ = 300 nm, t = 25 °C): t_R = 11.1 min (minor), t_R = 15.3 min (major). $[\alpha]_D^{20}$ = +32.0 (c = 1.3, $CHCl_3$). ^1H NMR (400 MHz, chloroform-*d*): δ 9.58 (s, 1H), 7.36 – 7.27 (m, 5H), 7.23 (tt, J = 6.5, 1.4 Hz, 3H), 7.14 (d, J = 3.3 Hz, 1H), 7.13 – 7.08 (m, 2H), 6.91 (dd, J = 8.4, 0.8 Hz, 1H), 6.84 (dd, J = 3.3, 0.9 Hz, 1H), 6.77 (d, J = 8.4 Hz, 1H), 5.29 (s, 2H), 4.41 (t, J = 7.0 Hz, 1H), 3.17 – 2.88 (m, 2H) ppm. $^{13}C\{^1H\}$ NMR (101 MHz, chloroform-*d*): δ 171.5, 143.1, 137.3, 136.7, 129.6, 128.9 (2C), 128.8 (2C), 128.6, 127.9, 127.8, 127.0 (2C), 126.9 (2C), 122.4, 118.0, 115.9, 104.9, 98.1, 50.4, 42.1, 39.4 ppm. IR (ATR): ν = 3197 (N-H, amide), 1666 (C=O, amide) cm^{-1}. HRMS (ESI+) *m*/*z*: calcd. for $C_{24}H_{21}N_2O$ $[M + H]^+$: 353.1648, found: 353.1651.

(*S*)-7,9-Dimethyl-4-phenyl-1,3,4,7-tetrahydro-2*H*-pyrrolo[2,3-*h*]quinolin-2-one (3f)

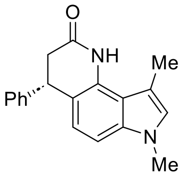

The title compound was synthesized according to the general procedure (reaction time: 15 hours), using aminoindole **1f** (44.5 mg, 0.2 mmol) and (*Z*)-2-bromo-3-phenylacrylaldehyde (63.3 mg, 0.3 mmol). The product was purified by column chromatography (hexane/EtOAc - 3:1 to 2:1), affording **3f** (53 mg, 91%) as a colorless oil.

Er = 96:4 (*ee* = 91%), the enantiomeric excess of product **3f** was determined by HPLC using a Lux® Amylose-1 (*n*-heptane/*i*-PrOH - 80:20, flow rate = 1.0 ml/min, λ = 227 nm, t = 25 °C): t_R = 11.9 min (major), t_R = 13.1 min (minor). $[\alpha]_D^{20}$ = +55.9 (c = 0.9, $CHCl_3$). ^1H NMR (400 MHz, chloroform-*d*): δ 8.05 (br s, 1H), 7.36 – 7.28 (m, 2H), 7.26 – 7.18 (m, 3H), 6.89 (d, J = 8.4 Hz, 1H), 6.83 – 6.74 (m, 2H), 4.38 (t, J = 6.7 Hz, 1H), 3.68 (s, 3H), 3.05 (dd, J = 16.0, 6.4 Hz, 1H), 2.96 (dd, J = 16.0, 7.0 Hz, 1H), 2.56 (d, J = 1.1 Hz, 3H) ppm. $^{13}C\{^1H\}$ NMR (101 MHz, chloroform-*d*): δ 170.3, 142.9, 138.0, 130.7, 128.9 (2C), 127.8 (2C), 127.7, 127.0, 122.6, 116.2, 115.6, 107.8, 104.5, 42.0, 39.1, 32.8, 12.5 ppm. IR (ATR): ν = 3114 (N-H, amide), 1672 (C=O, amide) cm^{-1}. HRMS (ESI+) *m*/*z*: calcd. for $C_{19}H_{19}N_2O$ $[M + H]^+$: 291.1492, found: 291.1495.

(*S*)-7-Methyl-4-(naphthalen-2-yl)-1,3,4,7-tetrahydro-2*H*-pyrrolo[2,3-*h*]quinolin-2-one (3g)

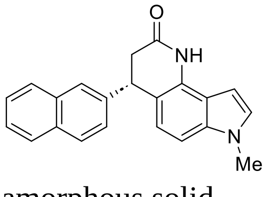

The title compound was synthesized according to the general procedure (reaction time: 15 hours), using aminoindole **1b** (29.2 mg, 0.2 mmol) and (*Z*)-2-bromo-3-(naphthalen-2-yl)acrylaldehyde (78.3 mg, 0.3 mmolThe product was purified by column chromatography (hexane/EtOAc - 2:1 to 1:1), affording **3g** (63 mg, 96%) as a white amorphous solid.

Er = 98:2 (*ee* = 96%), the enantiomeric excess of product **3g** was determined by HPLC using a Chiralpak® IA column (*n*-heptane/*i*-PrOH - 60:40, flow rate = 1.0 ml/min, λ = 225 nm, t = 25 °C): t_R = 9.9 min (minor), t_R = 20.5 min (major). $[\alpha]_D^{20}$ = +79.4 (c = 0.3, $CHCl_3$). ^1H NMR (400 MHz, chloroform-*d*): δ 8.42 (br s, 1H), 7.84 – 7.75 (m, 2H), 7.74 (dd, J = 6.2, 3.4 Hz, 1H), 7.60 (d, J = 1.8 Hz, 1H), 7.45 (dt, J = 6.2, 3.4 Hz, 2H), 7.38 (dd, J = 8.5, 1.8 Hz, 1H), 7.07 (d, J = 3.2 Hz, 1H), 6.95 (dd, J = 8.4, 0.8 Hz, 1H), 6.84 (d, J = 8.4 Hz, 1H), 6.54 (dd, J = 3.3, 0.9 Hz, 1H), 4.59 (t, J = 7.1 Hz, 1H), 3.78 (s, 3H), 3.11 (dd, J = 7.1, 5.3 Hz, 2H) ppm. $^{13}C\{^1H\}$ NMR (101 MHz, chloroform-*d*): δ 170.8, 140.3, 137.2, 133.7, 132.7, 129.4, 129.1, 128.8,

128.0, 127.8, 126.6, 126.3, 126.1, 125.9, 122.4, 117.5, 115.6, 104.7, 96.7, 42.2, 39.3, 33.3 ppm. IR (ATR): ν = 3195 (N-H, amide), 1664 (C=O, amide) cm^{-1}. HRMS (ESI+) *m*/*z*: calcd. for $C_{22}H_{19}N_2O$ [M + H]$^+$: 327.1492, found: 327.1495.

(*S*)-7-Methyl-4-(*p*-tolyl)-1,3,4,7-tetrahydro-2*H*-pyrrolo[2,3-*h*]quinolin-2-one (3h)

The title compound was synthesized according to the general procedure (reaction time: 72 hours), using aminoindole **1b** (29.2 mg, 0.2 mmol) and (*Z*)-2-bromo-3-(*p*-tolyl)acrylaldehyde (67.5 mg, 0.3 mmol). The product was purified by column chromatography (hexane/EtOAc - 3:1 to 2:1), affording **3h** (52 mg, 90%) as a white amorphous solid.

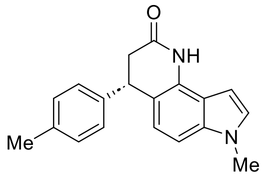

Er = 97:3 (*ee* = 94%), the enantiomeric excess of product **3h** was determined by HPLC using a Chiralpak® IA column (*n*-heptane/*i*-PrOH - 60:40, flow rate = 1.0 ml/min, λ = 223 nm, *t* = 25 °C): t_R = 7.6 min (minor), t_R = 15.6 min (major). $[\alpha]_D^{20}$ = +81.0 (*c* = 1.4, $CHCl_3$). ^{1}H NMR (400 MHz, chloroform-*d*): δ 9.36 (br s, 1H), 7.11 (s, 4H), 7.05 (d, *J* = 3.2 Hz, 1H), 6.95 (dd, *J* = 8.4, 0.9 Hz, 1H), 6.83 (d, *J* = 8.4 Hz, 1H), 6.72 (dd, *J* = 3.2, 0.9 Hz, 1H), 4.39 (t, *J* = 6.9 Hz, 1H), 3.77 (s, 3H), 3.15 – 2.88 (m, 2H), 2.33 (s, 3H) ppm. ^{13}C{^{1}H} NMR (101 MHz, chloroform-*d*): δ 171.4, 140.2, 137.1, 136.5, 129.5 (2C), 129.4, 129.1, 127.7 (2C), 122.2, 117.6, 115.8, 104.3, 97.3, 41.7, 39.6, 33.1, 21.1 ppm. IR (ATR): ν = 3195 (N-H, amide), 1664 (C=O, amide) cm^{-1}. HRMS (ESI+) *m*/*z*: calcd. for $C_{19}H_{19}N_2O$ [M + H]$^+$: 291.1492, found: 291.1496.

(*S*)-4-(4-Methoxyphenyl)-7-methyl-1,3,4,7-tetrahydro-2*H*-pyrrolo[2,3-*h*]quinolin-2-one (3i)

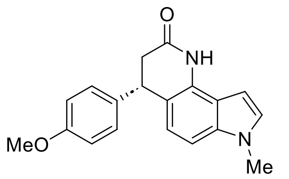

The title compound was synthesized according to the general procedure (reaction time: 40 hours), using aminoindole **1b** (29.2 mg, 0.2 mmol) and (*Z*)-2-bromo-3-(4-methoxyphenyl)acrylaldehyde (72.3 mg, 0.3 mmol). The product was purified by column chromatography (hexane/EtOAc - 2:1 to 1:1), affording **3i** (58 mg, 94%) as a brown amorphous solid.

Er = 95:5 (*ee* = 89%), the enantiomeric excess of product **3i** was determined by HPLC using a Chiralpak® IA column (*n*-heptane/*i*-PrOH - 80:20, flow rate = 1.0 ml/min, λ = 223 nm, *t* = 25 °C): t_R = 16.5 min (minor), t_R = 36.3 min (major). $[\alpha]_D^{20}$ = +69.0 (*c* = 1.8, $CHCl_3$). ^{1}H NMR (400 MHz, chloroform-*d*): δ 9.03 (br s, 1H), 7.14 – 7.08 (m, 2H), 7.05 (d, *J* = 3.2 Hz, 1H), 6.95 (dd, *J* = 8.4, 0.9 Hz, 1H), 6.83 (dd, *J* = 8.6, 2.1 Hz, 3H), 6.64 (dd, *J* = 3.2, 0.9 Hz, 1H), 4.37 (t, *J* = 6.8 Hz, 1H), 3.77 (s, 3H), 3.77 (s, 3H), 3.06 (dd, *J* = 16.0, 6.4 Hz, 1H), 2.95 (dd, *J* = 16.0, 7.4 Hz, 1H) ppm. ^{13}C{^{1}H} NMR (101 MHz, chloroform-*d*): δ 171.2, 158.6, 137.1, 135.2, 129.3, 129.1, 128.8 (2C), 122.2, 117.6, 116.0, 114.2 (2C), 104.4, 97.0, 55.4, 41.3, 39.7, 33.2 ppm. IR (ATR): ν = 3197 (N-H, amide), 1668 (C=O, amide) cm^{-1}. HRMS (ESI+) *m*/*z*: calcd. for $C_{19}H_{19}N_2O_2$ [M + H]$^+$: 307.1441, found: 307.1441.

(*S*)-7-Methyl-4-(4-nitrophenyl)-1,3,4,7-tetrahydro-2*H*-pyrrolo[2,3-*h*]quinolin-2-one (3j)

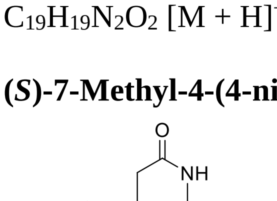

The title compound was synthesized according to the general procedure (reaction time: 4 hours), using aminoindole **1b** (29.2 mg, 0.2 mmol) and (*Z*)-2-bromo-3-(4-nitrophenyl)acrylaldehyde (76.8 mg, 0.3 mmol). The product was purified by column chromatography (hexane/EtOAc - 2:1 to 1:1), affording **3j** (60 mg, 93%) as an orange amorphous solid.

Er = 97:3 (*ee* = 95%), the enantiomeric excess of product **3j** was determined by HPLC using a Chiralpak® IA column (*n*-heptane/*i*-PrOH - 50:50, flow rate = 1.0 ml/min, λ = 289 nm, *t* = 25 °C): t_R = 12.5 min (minor), t_R = 25.2 min (major). $[\alpha]_D^{20}$ = +166.7 (*c* = 1.5, $CHCl_3$). ^{1}H NMR (400 MHz, chloroform-*d*): δ 9.22 (s, 1H), 8.08 – 7.95

(m, 2H), 7.33 – 7.27 (m, 2H), 7.06 (d, *J* = 3.2 Hz, 1H), 6.99 (dd, *J* = 8.4, 0.9 Hz, 1H), 6.83 (d, *J* = 8.4 Hz, 1H), 6.64 (dd, *J* = 3.2, 0.9 Hz, 1H), 4.50 (t, *J* = 6.1 Hz, 1H), 3.79 (s, 3H), 3.17 (dd, *J* = 16.1, 6.8 Hz, 1H), 2.96 (dd, *J* = 16.1, 5.5 Hz, 1H) ppm. $^{13}C\{^{1}H\}$ NMR (101 MHz, chloroform-*d*): δ 170.2, 150.8, 147.0, 137.3, 129.5, 129.4, 128.6 (2C), 124.1 (2C), 122.0, 117.7, 113.7, 105.0, 97.1, 42.0, 39.1, 33.3 ppm. IR (ATR): ν = 3197 (N-H, amide), 1664 (C=O, amide), 1512, 1342 (nitro) cm^{-1}. HRMS (ESI+) *m*/*z*: calcd. for $C_{18}H_{16}N_3O_3$ $[M + H]^+$: 322.1186, found: 322.1187.

(*S*)-4-(7-Methyl-2-oxo-2,3,4,7-tetrahydro-1*H*-pyrrolo[2,3-*h*]quinolin-4-yl)benzonitrile (3k)

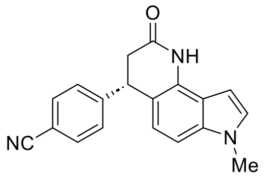

The title compound was synthesized according to the general procedure (reaction time: 15 hours), using aminoindole **1b** (29.2 mg, 0.2 mmol) and (*Z*)-4-(2-bromo-3-oxoprop-1-en-1-yl)benzonitrile (70.8 mg, 0.3 mmol). The product was purified by column chromatography (hexane/EtOAc - 2:1 to 1:1), affording **3k** (59 mg, 97%) as a white amorphous solid.

Er = 97:3 (*ee* = 94%), the enantiomeric excess of product **3k** was determined by HPLC using a Chiralpak® IA column (*n*-heptane/*i*-PrOH - 50:50, flow rate = 1.0 ml/min, λ = 227 nm, *t* = 25 °C): t_R = 9.1 min (minor), t_R = 15.9 min (major). $[\alpha]_D^{20}$ = +142.2 (*c* = 0.8, $CHCl_3$). ^1H NMR (400 MHz, chloroform-*d*): δ 9.35 (br s, 1H), 7.52 – 7.45 (m, 2H), 7.28 – 7.23 (m, 2H), 7.06 (d, *J* = 3.0 Hz, 1H), 6.99 (d, *J* = 8.3 Hz, 1H), 6.82 (d, *J* = 8.4 Hz, 1H), 6.67 (d, *J* = 3.2 Hz, 1H), 4.45 (t, *J* = 5.9 Hz, 1H), 3.79 (s, 3H), 3.15 (dd, *J* = 16.2, 6.6 Hz, 1H), 2.94 (dd, *J* = 16.5, 5.2 Hz, 1H) ppm. $^{13}C\{^{1}H\}$ NMR (101 MHz, chloroform-*d*): δ 170.9, 148.8, 137.3, 132.7 (2C), 129.5 (2C), 128.5 (2C), 122.0, 118.9, 117.7, 113.8, 110.8, 104.9, 97.3, 42.1, 39.1, 33.2 ppm. IR (ATR): ν = 3192 (N-H, amide), 2225 (CN, nitrile) 1664 (C=O, amide) cm^{-1}. HRMS (ESI+) *m*/*z*: calcd. for $C_{19}H_{16}N_3O$ $[M + H]^+$: 302.1288, found: 302.1291.

(*S*)-7-Methyl-4-(4-(trifluoromethyl)phenyl)-1,3,4,7-tetrahydro-2*H*-pyrrolo[2,3-*h*]quinolin-2-one (3l)

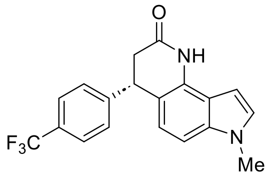

The title compound was synthesized according to the general procedure (reaction time: 15 hours), using aminoindole **1b** (29.2 mg, 0.2 mmol) and (*Z*)-2-bromo-3-(4-(trifluoromethyl)phenyl)acrylaldehyde (83.7 mg, 0.3 mmol). The product was purified by column chromatography (hexane/EtOAc - 2:1), affording **3l** (65 mg, 95%) as a white amorphous solid.

Er = 97:3 (*ee* = 94%), the enantiomeric excess of product **3l** was determined by HPLC using a Chiralpak® IA column (*n*-heptane/*i*-PrOH - 60:40, flow rate = 1.0 ml/min, λ = 221 nm, *t* = 25 °C): t_R = 7.0 min (minor), t_R = 10.8 min (major). $[\alpha]_D^{20}$ = +76.4 (*c* = 1.3, $CHCl_3$). ^1H NMR (400 MHz, chloroform-*d*): δ 9.77 (br s, 1H), 7.49 (d, *J* = 8.1 Hz, 2H), 7.29 (d, *J* = 8.1 Hz, 2H), 7.06 (d, *J* = 3.0 Hz, 1H), 6.98 (d, *J* = 8.4 Hz, 1H), 6.83 (d, *J* = 8.4 Hz, 1H), 6.78 (d, *J* = 3.2 Hz, 1H), 4.47 (t, *J* = 6.3 Hz, 1H), 3.78 (s, 3H), 3.15 (dd, *J* = 16.0, 6.6 Hz, 1H), 2.98 (dd, *J* = 15.9, 6.1 Hz, 1H) ppm. $^{13}C\{^{1}H\}$ NMR (101 MHz, chloroform-*d*): δ 171.0, 147.4, 137.3, 129.5, 129.3, 129.1 (q. *J* = 32.4 Hz), 128.1 (2C), 125.8 (q, *J* = 3.8 Hz, 2C), 124.3 (q, *J* = 272.0 Hz), 122.0, 117.8, 114.4, 104.7, 97.5, 42.0, 39.3, 33.2 ppm. ^{19}F NMR (376 MHz, chloroform-*d*): δ -62.42 (s, 3F) ppm. IR (ATR): ν = 3195 (N-H, amide), 1674 (C=O, amide) cm^{-1}. HRMS (ESI+) *m*/*z*: calcd. for $C_{19}H_{16}F_3N_2O$ $[M + H]^+$: 345.1209, found: 345.1211.

(*S*)-4-(4-Fluorophenyl)-7-methyl-1,3,4,7-tetrahydro-2*H*-pyrrolo[2,3-*h*]quinolin-2-one (3m)

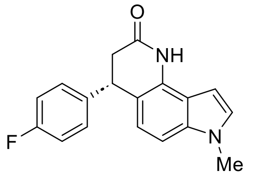

The title compound was synthesized according to the general procedure (reaction time: 40 hours), using aminoindole **1b** (29.2 mg, 0.2 mmol) and (*Z*)-2-bromo-3-(4-fluorophenyl)acrylaldehyde (68.7 mg, 0.3 mmol). The product was purified by column chromatography (hexane/EtOAc - 2:1 to 1:1), affording **3m** (44 mg, 75%) as a brown amorphous solid.

Er = 97:3 (*ee* = 94%), the enantiomeric excess of product **3m** was determined by HPLC using a Chiralpak® IA column (*n*-heptane/*i*-PrOH – 60:40, flow rate = 1.0 ml/min, λ = 223 nm, *t* = 25 °C): t_R = 8.6 min (minor), t_R = 12.7 min (major). $[\alpha]_D^{20}$ = +59.8 (*c* = 1.2, $CHCl_3$). ^{1}H NMR (400 MHz, chloroform-*d*): δ 9.36 (s, 1H), 7.18 – 7.10 (m, 2H), 7.06 (d, *J* = 3.2 Hz, 1H), 6.98 – 6.91 (m, 3H), 6.82 (d, *J* = 8.4 Hz, 1H), 6.70 (dd, *J* = 3.3, 0.9 Hz, 1H), 4.40 (t, *J* = 6.6 Hz, 1H), 3.77 (s, 3H), 3.09 (dd, *J* = 16.0, 6.5 Hz, 1H), 2.94 (dd, *J* = 16.0, 6.8 Hz, 1H) ppm. ^{13}C{^{1}H} NMR (101 MHz, chloroform-*d*): δ 171.1, 161.8 (d, *J* = 245.0 Hz), 138.9 (d, *J* = 3.2 Hz), 137.2, 129.28 (d, *J* = 8.1 Hz, 2C), 129.25 (2C), 122.1, 117.7, 115.6 (d, *J* = 21.3 Hz, 2C), 115.3, 104.6, 97.3, 41.4, 39.6, 33.2 ppm. ^{19}F NMR (376 MHz, chloroform-*d*): δ -116.18 (tt, *J* = 8.7, 5.3 Hz, 1F) ppm. IR (ATR): ν = 3199 (N-H, amide), 1668 (C=O, amide) cm^{-1}. HRMS (ESI+) *m*/*z*: calcd. for $C_{18}H_{16}FN_2O$ [M + H]$^+$: 295.1241, found: 295.1243.

(*S*)-4-(4-Chlorophenyl)-7-methyl-1,3,4,7-tetrahydro-2*H*-pyrrolo[2,3-*h*]quinolin-2-one (3n)

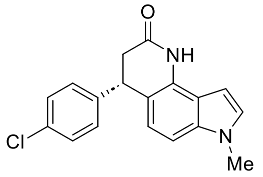

The title compound was synthesized according to the general procedure (reaction time: 15 hours), using aminoindole **1b** (29.2 mg, 0.2 mmol) and (*Z*)-2-bromo-3-(4-chlorophenyl)acrylaldehyde (73.7 mg, 0.3 mmol). The product was purified by column chromatography (hexane/EtOAc - 2:1 to 1:1), affording **3n** (62 mg, 98%) as a white amorphous solid.

Er = 97:3 (*ee* = 95%), the enantiomeric excess of product **3n** was determined by HPLC using a Chiralpak® IA column (*n*-heptane/*i*-PrOH - 80:20, flow rate = 1.0 ml/min, λ = 233 nm, *t* = 25 °C): t_R = 13.5 min (minor), t_R = 30.2 min (major). $[\alpha]_D^{20}$ = +84.7 (*c* = 0.9, $CHCl_3$). ^{1}H NMR (400 MHz, chloroform-*d*): δ 9.27 (br s, 1H), 7.25 – 7.18 (m, 2H), 7.14 – 7.07 (m, 2H), 7.06 (d, *J* = 3.2 Hz, 1H), 6.96 (dd, *J* = 8.4, 0.9 Hz, 1H), 6.81 (d, *J* = 8.3 Hz, 1H), 6.68 (dd, *J* = 3.2, 0.9 Hz, 1H), 4.38 (t, *J* = 6.6 Hz, 1H), 3.78 (s, 3H), 3.09 (dd, *J* = 16.0, 6.6 Hz, 1H), 2.93 (dd, *J* = 16.0, 6.7 Hz, 1H) ppm. ^{13}C{^{1}H} NMR (101 MHz, chloroform-*d*): δ 170.9, 141.7, 137.2, 132.7, 129.4, 129.3, 129.2 (2C), 129.0 (2C), 122.1, 117.7, 115.0, 104.6, 97.2, 41.5, 39.4, 33.2 ppm. IR (ATR): ν = 3197 (N-H, amide), 1672 (C=O, amide) cm^{-1}. HRMS (ESI+) *m*/*z*: calcd. for $C_{18}H_{16}ClN_2O$ [M + H]$^+$: 311.0946, found: 311.0944.

(*S*)-4-(4-Bromophenyl)-7-methyl-1,3,4,7-tetrahydro-2*H*-pyrrolo[2,3-*h*]quinolin-2-one (3o)

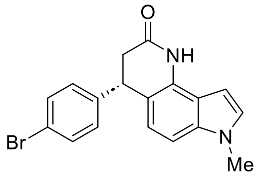

The title compound was synthesized according to the general procedure (reaction time: 15 hours), using aminoindole **1b** (29.2 mg, 0.2 mmol) and (*Z*)-2-bromo-3-(4-bromophenyl)acrylaldehyde (87.0 mg, 0.3 mmol). The product was purified by column chromatography (hexane/EtOAc - 3:1 to 2:1), affording **3o** (70 mg, 99%) as a white amorphous solid.

Er = 97:3 (*ee* = 94%), the enantiomeric excess of product **3o** was determined by HPLC using a Chiralpak® IA column (*n*-heptane/*i*-PrOH - 60:40, flow rate = 1.0 ml/min, λ = 223 nm, *t* = 25 °C): t_R = 8.6 min (minor), t_R = 17.4 min (major). $[\alpha]_D^{20}$ = +95.8 (*c* = 2.0, $CHCl_3$). ^{1}H NMR (400 MHz, chloroform-*d*): δ 9.57 (br s, 1H), 7.37 (d, *J* = 8.4 Hz, 2H), 7.05 (dd, *J* = 5.9, 2.5 Hz, 3H), 6.96 (d, *J* = 8.3 Hz, 1H), 6.81 (d, *J* = 8.4 Hz, 1H), 6.74 (d, *J* = 3.1 Hz, 1H), 4.36 (t, *J* = 6.5 Hz, 1H), 3.77 (s, 3H), 3.09 (ddd, *J* = 16.0, 6.5, 1.2 Hz, 1H),

2.93 (dd, J = 16.0, 6.6 Hz, 1H) ppm. $^{13}C\{^{1}H\}$ NMR (101 MHz, chloroform-d): δ 171.1, 142.3, 137.2, 131.9 (2C), 129.54 (2C), 129.45, 129.2, 122.0, 120.7, 117.7, 114.8, 104.6, 97.4, 41.6, 39.4, 33.2 ppm. IR (ATR): ν = 3197 (N-H, amide), 1644 (C=O, amide), 725 (C-Br) cm^{-1}. HRMS (ESI+) m/z: calcd. for $C_{18}H_{16}BrN_2O$ $[M + H]^+$: 355.0441, found: 355.0441.

(*S*)-4-(3-Chlorophenyl)-7-methyl-1,3,4,7-tetrahydro-2*H*-pyrrolo[2,3-*h*]quinolin-2-one (3p)

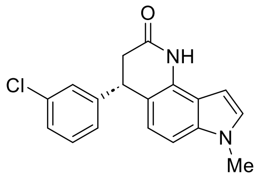

The title compound was synthesized according to the general procedure (reaction time: 15 hours), using aminoindole **1b** (29.2 mg, 0.2 mmol) and (*Z*)-2-bromo-3-(3-chlorophenyl)acrylaldehyde (73.7 mg, 0.3 mmol). The product was purified by column chromatography (hexane/EtOAc - 3:1 to 2:1), affording **3p** (58 mg, 93%) as a colorless oil.

Er = 98:2 (*ee* = 96%), the enantiomeric excess of product **3p** was determined by HPLC using a Chiralpak® IA column (*n*-heptane/*i*-PrOH - 60:40, flow rate = 1.0 ml/min, λ = 222 nm, t = 25 °C): t_R = 8.2 min (minor), t_R = 9.7 min (major). $[\alpha]_D^{20}$ = +85.6 (c = 1.1, $CHCl_3$). ^{1}H NMR (400 MHz, chloroform-d): δ 9.49 (br s, 1H), 7.19 (td, J = 3.5, 1.3 Hz, 3H), 7.07 (dd, J = 5.1, 2.5 Hz, 2H), 6.97 (dd, J = 8.4, 0.9 Hz, 1H), 6.82 (d, J = 8.4 Hz, 1H), 6.74 (dd, J = 3.2, 0.9 Hz, 1H), 4.38 (t, J = 6.6 Hz, 1H), 3.77 (s, 3H), 3.10 (dd, J = 16.1, 6.6 Hz, 1H), 2.96 (dd, J = 16.1, 6.7 Hz, 1H) ppm. $^{13}C\{^{1}H\}$ NMR (101 MHz, chloroform-d): δ 170.9, 145.4, 137.2, 134.6, 130.2, 129.5, 129.3, 128.0, 127.2, 126.0, 122.1, 117.7, 114.6, 104.6, 97.4, 41.9, 39.4, 33.2 ppm. IR (ATR): ν = 3194 (N-H, amide), 1672 (C=O, amide), 733 (C-Cl) cm^{-1}. HRMS (ESI+) m/z: calcd. for $C_{18}H_{16}ClN_2O$ $[M + H]^+$: 311.0946, found: 311.0949.

(*R*)-4-(Furan-2-yl)-7-methyl-1,3,4,7-tetrahydro-2*H*-pyrrolo[2,3-*h*]quinolin-2-one (3q)

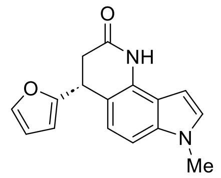

The title compound was synthesized according to the general procedure (reaction time: 40 hours), using aminoindole **1b** (29.2 mg, 0.2 mmol) and (*Z*)-2-bromo-3-(furan-2-yl)acrylaldehyde (60.3 mg, 0.3 mmol). The product was purified by column chromatography (hexane/EtOAc - 2:1 to 1:1), affording **3q** (53 mg, 98%) as a white amorphous solid.

Er = 96:4 (*ee* = 92%), the enantiomeric excess of product **3q** was determined by HPLC using a Chiralpak® IA column (*n*-heptane/*i*-PrOH - 60:40, flow rate = 1.0 ml/min, λ = 296 nm, t = 25 °C): t_R = 7.9 min (minor), t_R = 12.0 min (major). $[\alpha]_D^{20}$ = +79.7 (c = 1.2, $CHCl_3$). ^{1}H NMR (400 MHz, chloroform-d): δ 9.40 (s, 1H), 7.32 (dd, J = 1.9, 0.9 Hz, 1H), 7.04 (t, J = 3.0 Hz, 1H), 7.04 – 6.97 (m, 2H), 6.72 (dd, J = 3.2, 0.8 Hz, 1H), 6.23 (dd, J = 3.3, 1.9 Hz, 1H), 5.95 (dt, J = 3.3, 0.9 Hz, 1H), 4.44 (dd, J = 6.3, 5.2 Hz, 1H), 3.77 (s, 3H), 3.14 (dd, J = 16.2, 5.1 Hz, 1H), 3.04 (dd, J = 16.1, 6.5 Hz, 1H) ppm. $^{13}C\{^{1}H\}$ NMR (101 MHz, chloroform-d): δ 171.0, 155.6, 142.0, 137.3, 129.2, 129.1, 122.0, 117.8, 113.1, 110.3, 106.1, 104.4, 97.3, 36.2, 36.1, 33.2 ppm. IR (ATR): ν = 3199 (N-H, amide), 1664 (C=O, amide) cm^{-1}. HRMS (ESI+) m/z: calcd. for $C_{16}H_{15}N_2O_2$ $[M + H]^+$: 267.1128, found: 267.1133.

(*R*)-7-Methyl-4-(thiophen-2-yl)-1,3,4,7-tetrahydro-2H-pyrrolo[2,3-h]quinolin-2-one (3r)

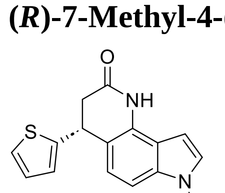

The title compound was synthesized according to the general procedure (reaction time: 15 hours), using aminoindole **1b** (29.2 mg, 0.2 mmol) and (*Z*)-2-bromo-3-(thiophen-2-yl)acrylaldehyde (65.1 mg, 0.3 mmol). The product was purified by column chromatography (hexane/EtOAc - 2:1), affording **3r** (54 mg, 96%) as a white amorphous solid.

Er = 96:4 (*ee* = 93%), the enantiomeric excess of product **3r** was determined by HPLC using a Chiralpak® IA column (*n*-heptane/*i*-PrOH - 60:40, flow rate = 1.0 ml/min, λ = 309 nm, t = 25 °C): t_R = 8.2 min (minor), t_R = 16.1 min (major). $[\alpha]_D^{20}$ = +55.2 (c = 1.8, $CHCl_3$). ^{1}H NMR (400 MHz, chloroform-d): δ 9.27 (br s, 1H), 7.14 (dd, J = 5.1, 1.2 Hz, 1H),

7.09 – 6.97 (m, 3H), 6.88 (dd, *J* = 5.1, 3.5 Hz, 1H), 6.81 (dt, *J* = 3.5, 1.1 Hz, 1H), 6.68 (dd, *J* = 3.1, 0.7 Hz, 1H), 4.68 – 4.57 (m, 1H), 3.78 (s, 3H), 3.23 – 2.97 (m, 2H) ppm. $^{13}C\{^1H\}$ NMR (101 MHz, chloroform-*d*): δ 170.9, 147.0, 137.3, 129.3, 128.9, 126.9, 124.6, 124.4, 121.9, 117.7, 115.6, 104.5, 97.3, 39.7, 37.6, 33.2 ppm. IR (ATR): ν = 3195 (N-H, amide), 1662 (C=O, amide) cm^{-1}. HRMS (ESI+) *m*/*z*: calcd. for $C_{16}H_{15}N_2OS$ $[M + H]^+$: 283.0900, found: 283.0904.

Ethyl (*R*)-7-methyl-2-oxo-2,3,4,7-tetrahydro-1*H*-pyrrolo[2,3-*h*]quinoline-4-carboxylate (3s)

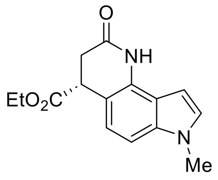

The title compound was synthesized according to the general procedure (reaction time: 4 hours), using aminoindole **1b** (29.2 mg, 0.2 mmol) and ethyl (*Z*)-3-bromo-4-oxobut-2-enoate (62.1 mg, 0.3 mmol). The product was purified by column chromatography (hexane/EtOAc - 2:1 to 1:1), affording **3s** (16 mg, 28%) as a brown amorphous solid.

Er = 97:3 (*ee* = 94%), the enantiomeric excess of product **3s** was determined by HPLC using a Chiralpak® IA column (*n*-heptane/*i*-PrOH - 60:40, flow rate = 1.0 ml/min, λ = 217 nm, *t* = 25 °C): t_R = 7.9 min (minor), t_R = 17.2 min (major). $[\alpha]_D^{20}$ = +38.9 (*c* = 0.3, $CHCl_3$). ^1H NMR (400 MHz, chloroform-*d*): δ 8.35 (br s, 1H), 7.14 (d, *J* = 8.4 Hz, 1H), 7.04 (d, *J* = 3.2 Hz, 1H), 7.00 (dd, *J* = 8.4, 0.9 Hz, 1H), 6.52 (dd, *J* = 3.2, 0.9 Hz, 1H), 4.12 (qd, *J* = 7.1, 1.1 Hz, 2H), 3.99 (dd, *J* = 6.7, 3.6 Hz, 1H), 3.78 (s, 3H), 3.03 (ddd, *J* = 16.3, 3.6, 0.8 Hz, 1H), 2.88 (dd, *J* = 16.3, 6.7 Hz, 1H), 1.20 (t, *J* = 7.1 Hz, 3H) ppm. $^{13}C\{^1H\}$ NMR (101 MHz, chloroform-*d*): δ 172.6, 169.8, 137.5, 129.6, 129.3, 122.4, 117.4, 109.4, 104.5, 96.9, 61.4, 42.6, 33.8, 33.2, 14.2 ppm. IR (ATR): ν = 3209 (N-H, amide), 1720 (C=O, ester) 1668 (C=O, amide) cm^{-1}. HRMS (ESI+) *m*/*z*: calcd. for $C_{15}H_{17}N_2O_3$ $[M + H]^+$: 273.1234, found: 273.1232.

(*R*)-4-Butyl-7-methyl-1,3,4,7-tetrahydro-2*H*-pyrrolo[2,3-*h*]quinolin-2-one (3t)

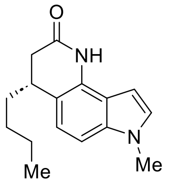

The title compound was synthesized according to the general procedure (reaction time: 15 hours), using aminoindole **1b** (29.2 mg, 0.2 mmol) and (*Z*)-2-bromohept-2-enal (57.3 mg, 0.3 mmol). The product was purified by column chromatography (hexane/EtOAc - 4:1 to 2:1), affording **3t** (44 mg, 85%) as a white amorphous solid.

Er = 98:2 (*ee* = 97%), the enantiomeric excess of product **3t** was determined by HPLC using a Chiralpak® IA column (*n*-heptane/*i*-PrOH - 60:40, flow rate = 1.0 ml/min, λ = 310 nm, *t* = 25 °C): t_R = 5.6 min (minor), t_R = 6.9 min (major). $[\alpha]_D^{20}$ = +41.9 (*c* = 1.3, $CHCl_3$). ^1H NMR (400 MHz, chloroform-*d*): δ 9.21 (s, 1H), 7.04 (d, *J* = 3.2 Hz, 1H), 7.03 – 6.96 (m, 2H), 6.67 (dd, *J* = 3.2, 0.8 Hz, 1H), 3.78 (s, 3H), 3.01 (qd, *J* = 7.1, 3.2 Hz, 1H), 2.89 (dd, *J* = 15.9, 6.3 Hz, 1H), 2.66 (ddd, *J* = 16.0, 3.2, 0.9 Hz, 1H), 1.71 – 1.51 (m, 2H), 1.44 – 1.20 (m, 4H), 0.87 (t, *J* = 7.1 Hz, 3H) ppm. $^{13}C\{^1H\}$ NMR (101 MHz, chloroform-*d*): δ 172.2, 136.9, 128.9, 128.5, 122.0, 117.8, 117.1, 104.0, 97.0, 37.1, 36.4, 34.8, 33.1, 29.3, 22.8, 14.1 ppm. IR (ATR): ν = 3199 (N-H, amide), 1662 (C=O, amide) cm^{-1}. HRMS (ESI+) *m*/*z*: calcd. for $C_{16}H_{21}N_2O$ $[M + H]^+$: 257.1648, found: 257.1647.

3-Methyl-9-phenyl-3,6,8,9-tetrahydro-7*H*-9λ^3-pyrrolo[3,2-*f*]quinolin-7-one (3u)

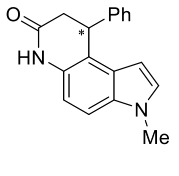

The title compound was synthesized according to the general procedure (reaction time: 15 hours), using aminoindole **1k** (29.2 mg, 0.2 mmol) and (*Z*)-2-bromo-3-phenylacrylaldehyde (63.3 mg, 0.3 mmol). The product was purified by column chromatography (hexane/EtOAc - 2:1 to 1:1), affording **3u** (7 mg, 14%) as a white amorphous solid.

Er = 96:4 (*ee* = 92%), the enantiomeric excess of product **3u** was determined by HPLC using a Chiralpak® IA column (*n*-heptane/*i*-PrOH - 80:20, flow rate = 1.0 ml/min, λ = 256 nm, *t* = 25 °C): t_R = 9.4 min (minor), t_R = 10.6 min (major). $[\alpha]_D^{20}$ = -3.9 (*c* = 0.4, $CHCl_3$). ^1H NMR

(400 MHz, chloroform-*d*): δ 8.19 (s, 1H), 7.26 – 7.11 (m, 6H), 7.00 (d, *J* = 3.2 Hz, 1H), 6.78 (d, *J* = 8.5 Hz, 1H), 6.19 (dd, *J* = 3.1, 0.9 Hz, 1H), 4.62 (dd, *J* = 7.2, 3.2 Hz, 1H), 3.76 (s, 3H), 3.13 (dd, *J* = 16.2, 7.3 Hz, 1H), 2.95 (ddd, *J* = 16.2, 3.2, 0.9 Hz, 1H) ppm. $^{13}C\{^{1}H\}$ NMR (101 MHz, chloroform-*d*): δ 170.1, 142.6, 134.0, 130.2, 129.7, 128.9 (2C), 127.5, 127.3 (2C), 127.0, 116.6, 111.4, 109.1, 99.1, 39.7, 38.8, 33.2 ppm. IR (ATR): ν = 3172 (N-H, amide), 1672 (C=O, amide) cm^{-1}. HRMS (ESI+) *m*/*z*: calcd. for $C_{18}H_{17}N_{2}O$ $[M + H]^{+}$: 277.1335, found: 277.1336.

1-Methyl-6-phenyl-1,6,7,9-tetrahydro-8*H*-6λ^{3}-pyrrolo[3,2-*h*]quinolin-8-one (3v)

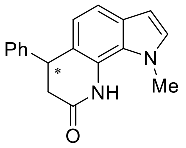

The title compound was synthesized according to the general procedure (reaction time: 15 hours), using aminoindole **1m** (29.2 mg, 0.2 mmol) and (*Z*)-2-bromo-3-phenylacrylaldehyde (63.3 mg, 0.3 mmol). The product was purified by column chromatography (hexane/EtOAc - 2:1 to 1:1), affording **3v** (39 mg, 71%) as a white foam.
Er = 96:4 (*ee* = 93%), the enantiomeric excess of product **3v** was determined by HPLC using a Chiralpak® IB column (*n*-heptane/*i*-PrOH - 80:20, flow rate = 1.0 ml/min, λ = 234 nm, *t* = 25 °C): t_R = 11.0 min (major), t_R = 14.0 min (minor). $[\alpha]_D^{20}$ = +143.8 (*c* = 1.2, $CHCl_3$). ^{1}H NMR (400 MHz, chloroform-*d*): δ 8.34 (br s, 1H), 7.31 – 7.15 (m, 6H), 6.92 (d, *J* = 3.1 Hz, 1H), 6.74 (d, *J* = 8.1 Hz, 1H), 6.43 (d, *J* = 3.1 Hz, 1H), 4.40 (t, *J* = 6.4 Hz, 1H), 4.08 (s, 3H), 3.16 – 2.85 (m, 2H) ppm. $^{13}C\{^{1}H\}$ NMR (101 MHz, chloroform-*d*): δ 170.7, 142.2, 131.5, 130.8, 128.9 (2C), 127.8 (2C), 127.1, 125.2, 123.3, 120.7, 120.4, 116.4, 101.6, 42.3, 39.0, 36.6 ppm. IR (ATR): ν = 3234 (N-H, amide), 1666 (C=O, amide) cm^{-1}. HRMS (ESI+) *m*/*z*: calcd. for $C_{18}H_{17}N_{2}O$ $[M + H]^{+}$: 277.1335, found: 277.1335.

Follow-up transformations

(*S*)-1,7-Dimethyl-4-phenyl-1,3,4,7-tetrahydro-2*H*-pyrrolo[2,3-*h*]quinolin-2-one (5)

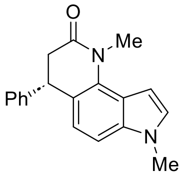

The round-bottom flask (25 ml) was charged with a magnetic stirrer and **3b** (100.0 mg, 0.36 mmol, 1.0 equiv., 97:3 *er*), followed by anhydrous THF (5.0 ml) at room temperature. Then, mixture was cooled to 0 °C (ice/water bath) and suspension of sodium hydride (60%, *w*/*w*, in oil, 15.8 mg, 0.40 mmol, 1.1 equiv.) was added portionwise (gas evolution). At 0°C, the mixture was stirred for 30 minutes. After this time, methyl iodide (9 µl, 0.47 mmol, 1.3 equiv.) was added in one portion. Reaction mixture was heated up to room temperature, and left to stir for 3 hours (minor amount of starting material detected by TLC), followed by addition of sodium hydride suspension of sodium hydride (60%, *w*/*w*, in oil, 15.8 mg, 0.40 mmol, 1.1 equiv.) and methyl iodide (9 µl, 0.47 mmol, 1.3 equiv.). The reaction mixture was stirred for 2 hours at room temperature. Once the starting amide was no longer detected (by TLC), the reaction was quenched by slowly adding a saturated aqueous solution of ammonium chloride (10 ml), and diluted with EtOAc (20 ml). The organic phase was separated. The water phase was extracted with EtOAc (3 × 20 ml). The collected organic phases were washed with brine (1 × 20 ml) and dried under anhydrous $MgSO_4$. After filtration of the solid, the filtrate was concentrated under reduced pressure. The crude product was purified by column chromatography (eluting by hexane/EtOAc - 3:1 to 2:1), affording **5** (84 mg, 81%) as a green oil.
Er = 97:3 (*ee* = 95%), the enantiomeric excess of product **5** was determined by HPLC using a Chiralpak® IA column (*n*-heptane/*i*-PrOH - 80:20, flow rate = 1.0 ml/min, λ = 190 nm, *t* = 25 °C): t_R = 9.0 min (minor), t_R = 13.0 min (major). $[\alpha]_D^{20}$ = +26.7 (*c* = 1.3, $CHCl_3$). ^{1}H NMR (400 MHz, chloroform-*d*): δ 7.35 – 7.27 (m, 2H), 7.26 – 7.21 (m, 1H), 7.20 – 7.14 (m, 2H), 7.09 (d, *J* = 3.3 Hz, 1H), 7.02 (dd, *J* = 8.3, 0.9 Hz, 1H), 6.87 (d, *J* = 8.3 Hz, 1H), 6.66 (dd, *J* = 3.3, 0.9 Hz, 1H), 4.30 (t, *J* = 6.3 Hz, 1H), 3.78 (s, 3H), 3.69 (s, 3H), 3.03 – 2.95 (m, 2H) ppm. $^{13}C\{^{1}H\}$

NMR (101 MHz, chloroform-*d*): δ 170.9, 142.0, 138.1, 133.9, 128.9, 128.8 (2C), 127.8 (2C), 126.9, 122.1, 120.4, 119.0, 104.9, 100.2, 41.8, 39.8, 33.7, 33.2 ppm. IR (ATR): ν = 1658 (C=O, amide) cm^{-1}. HRMS (ESI+) *m*/*z*: calcd. for $C_{19}H_{19}N_2O$ $[M + H]^+$: 291.1492, found: 291.1493.

(*S*)-7-Methyl-4-phenyl-2,3,4,7-tetrahydro-1*H*-pyrrolo[2,3-*h*]quinoline (6)

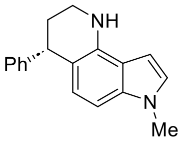

The round-bottom flask (25 ml) was charged with a magnetic stirrer and **3b** (100.0 mg, 0.36 mmol, 1.0 equiv., 97:3 *er*), followed by anhydrous THF (5.0 ml) at room temperature. Then, mixture was cooled to 0 °C (ice/water bath) and lithium aluminum hydride (41.0 mg, 1.08 mmol, 3.0 equiv.) was added portionwise (gas evolution). Then, the reaction mixture was heated up to room temperature and left to stir for 2 hours. Once the starting amide was no longer detected (by TLC), the reaction was quenched by slowly adding a saturated aqueous solution of ammonium chloride (20 ml), and diluted with EtOAc (20 ml). The organic phase was separated. The water phase was extracted with EtOAc (3 × 20 ml). The collected organic phases were washed with brine (1 × 20 ml) and dried under anhydrous $MgSO_4$. After filtration of the solid, the filtrate was concentrated under reduced pressure. The crude product was purified by column chromatography (eluting by hexane/EtOAc - 7:1 to 6:1), affording **6** (50 mg, 53%) as a green oil.as a light-yellow oil.

Er = 97:3 (*ee* = 95%), the enantiomeric excess of product **6** was determined by HPLC using a Chiralpak® IB column (*n*-heptane/*i*-PrOH - 80:20, flow rate = 1.0 ml/min, λ = 313 nm, *t* = 25 °C): t_R = 14.4 min (minor), t_R = 23.9 min (major). $[\alpha]_D^{20}$ = +91.8 (*c* = 0.7, $CHCl_3$). ^{1}H NMR (400 MHz, chloroform-*d*): δ 7.35 – 7.28 (m, 2H), 7.25 – 7.16 (m, 3H), 6.97 (d, *J* = 3.1 Hz, 1H), 6.73 (d, *J* = 8.4 Hz, 1H), 6.70 – 6.64 (m, 1H), 6.40 (dd, *J* = 3.2, 0.8 Hz, 1H), 4.33 (t, *J* = 5.6 Hz, 1H), 3.75 (s, 3H), 3.47 – 3.30 (m, 2H), 2.37 (dddd, *J* = 12.9, 9.2, 5.5, 4.0 Hz, 1H), 2.15 (dtd, *J* = 13.0, 6.0, 3.5 Hz, 1H) ppm. ^{13}C{^{1}H} NMR (101 MHz, chloroform-*d*): δ 148.1, 137.5, 136.4, 128.9 (2C), 128.2 (2C), 126.8, 125.9, 125.1, 116.8, 112.1, 99.4, 96.8, 42.5, 39.0, 33.0, 31.9 ppm. IR (ATR): ν = 3406 (N-H, amine) cm^{-1}. HRMS (ESI+) *m*/*z*: calcd. for $C_{18}H_{19}N_2$ $[M + H]^+$: 263.1543, found: 263.1543.

(*S*)-7-Methyl-9-(3-oxobutyl)-4-phenyl-1,3,4,7-tetrahydro-2*H*-pyrrolo[2,3-*h*]quinolin-2-one (7)

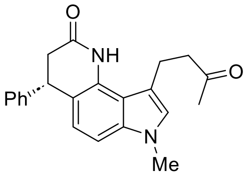

The vial (4 ml) was charged with s magnetic stirrer, **3b** (27.6 mg, 0.1 mmol, 97:3 *er*), corresponding catalyst (0.02 mmol, 0.2 equiv.) and corresponding solvent (1.0 ml). Then, but-3-en-2-one (25 µl, 0.3 mmol, 3.0 equiv.) was added and mixture was left to stir for 48 hours. Once the reaction was completed by TLC, the solvent was evaporated. The crude product was purified by column chromatography (eluting by hexane/EtOAc - 1:1), affording **7** as a white amorphous solid.

Condition A: Reaction was performed in MeCN, using $Yb(OTf)_3$ (12.4 mg) as a catalyst, affording **7** (19 mg, 56%, 97:3 *er*).

Condition B: Reaction was performed in DCM, using diphenyl phosphate (5.0 mg) as a catalyst, affording **7** (34 mg, 99%, 97:3 *er*).

Er = 97:3 (*ee* = 94%), the enantiomeric excess of product **7** was determined by HPLC using a Chiralpak® IB column (*n*-heptane/*i*-PrOH - 80:20, flow rate = 1.0 ml/min, λ = 227 nm, *t* = 25 °C): t_R = 22.5 min (major), t_R = 24.7 min (minor). $[\alpha]_D^{20}$ = +84.0 (*c* = 0.9, $CHCl_3$). ^{1}H NMR (400 MHz, chloroform-*d*): δ 8.58 (br s, 1H), 7.34 – 7.28 (m, 2H), 7.26 – 7.22 (m, 1H), 7.22 – 7.16 (m, 2H), 6.88 (d, *J* = 8.4 Hz, 1H), 6.80 (t, *J* = 4.2 Hz, 2H), 4.38 (t, *J* = 6.8 Hz, 1H), 3.68 (s, 3H), 3.21 – 3.14 (m, 2H), 3.00 (qd, *J* = 15.8, 6.8 Hz, 2H), 2.90 (t, *J* = 6.7 Hz, 2H), 2.17 (s, 3H) ppm. ^{13}C{^{1}H} NMR (101 MHz, chloroform-*d*): δ 208.2, 170.4, 142.9, 137.8, 130.6, 128.9 (2C), 127.9 (2C), 127.6, 127.0, 122.6, 116.6, 115.7, 112.3, 104.5, 45.2, 42.2, 39.2, 32.9, 30.2,

20.4 ppm. IR (ATR): ν = 3232 (N-H, amide), 1711 (C=O, ketone), 1676 (C=O, amide) cm^{-1}. HRMS (ESI+) *m*/*z*: calcd. for $C_{22}H_{23}N_2O_2$ $[M + H]^+$: 347.1754, found: 347.1754.

(*S*)-7-Methyl-4-phenyl-1,3,4,7,8,9-hexahydro-2*H*-pyrrolo[2,3-*h*]quinolin-2-one (8)

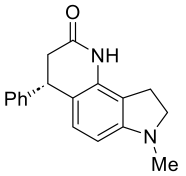

The vial (4 ml) was charged with a magnetic stirrer and **3b** (27.6 mg, 0.1 mmol, 1.0 equiv., 97:3 *er*), followed by acetic acid (1.0 ml) at room temperature. Then, $NaBH_3CN$ (62.8 mg, 1.0 mmol, 10.0 equiv.) was added portionwise (gas evolution). Then, the reaction mixture was left to stir for 3 hours. Once the starting amide was no longer detected (by TLC), the reaction was poured into diluted aqueous solution of sodium hydroxide (1M, 30 ml). Resulting mixture was was extracted with EtOAc (3 × 10 ml). The collected organic phases were washed with brine (1 × 10 ml) and dried under anhydrous $MgSO_4$. After filtration of the solid, the filtrate was concentrated under reduced pressure. The crude product was purified by column chromatography (eluting by hexane/EtOAc - 2:1 to 1:1), affording **8** (18 mg, 66%) as a white amorphous solid.

Er = 97:3 (*ee* = 94%), the enantiomeric excess of product **8** was determined by HPLC using a Chiralpak® IB column (*n*-heptane/*i*-PrOH - 80:20, flow rate = 1.0 ml/min, λ = 237 nm, *t* = 25 °C): t_R = 9.7 min (major), t_R = 13.0 min (minor). $[\alpha]_D^{20}$ = +63.7 (*c* = 0.7, $CHCl_3$). ^1H NMR (400 MHz, chloroform-*d*): δ 8.45 (br s, 1H), 7.36 – 7.27 (m, 2H), 7.26 – 7.22 (m, 1H), 7.21 – 7.17 (m, 2H), 6.63 (dt, *J* = 8.0, 0.9 Hz, 1H), 6.11 (d, *J* = 7.9 Hz, 1H), 4.22 (t, *J* = 7.2 Hz, 1H), 3.38 (t, *J* = 8.2 Hz, 2H), 2.92 (dd, *J* = 7.2, 2.2 Hz, 4H), 2.74 (s, 3H) ppm. $^{13}C\{^1H\}$ NMR (101 MHz, chloroform-*d*): δ 171.3, 153.8, 142.7, 133.5, 128.9 (2C), 127.9 (2C), 127.7, 127.0, 116.9, 115.3, 102.1, 56.3, 41.7, 39.2, 36.3, 25.6 ppm. IR (ATR): ν = 3199 (N-H, amide), 1666 (C=O, amide) cm^{-1}. HRMS (ESI+) *m*/*z*: calcd. for $C_{18}H_{19}N_2O$ $[M + H]^+$: 279.1492, found: 279.1495.

Crystallographic data

The diffraction data of single crystals of **3b** and *ent*-**3b** samples were obtained on Bruker D8 VENTURE Kappa Duo PHOTONIII by IμS micro-focus sealed tube with CuKα (λ = 1.54178) radiation at low temperature preserved by Cryostream Cooler. The structures were solved by direct methods (XT)[13] and refined by full matrix least squares based on F^2 (SHELXL2019).[14] The hydrogen atoms on carbon were fixed into idealized positions (riding model) and assigned temperature factors either $H_{iso}(H)$ = 1.2 U_{eq}(pivot atom) or 1.5 U_{eq}(pivot atom) for methyl moiety. The hydrogen atoms on N-H moiety were found on difference Fourier map and restrain during refinement under rigid-body assumption.

Each crystal contains one enantiomer of the same compound, however the determination of absolute structure[15] was satisfactory only for *ent*-**3b** crystal, whereas the quality of the crystal of **3b** was not suitable for such refinement based only on weak anomalous scattering of N and O atoms. Basic crystallographic data are given in Table S8.

X-ray crystallographic data have been deposited with the Cambridge Crystallographic Data Centre (CCDC), the deposition numbers 2360229 and 2360228 for **3b** and *ent*-**3b** and can be obtained free of charge from the Centre via its website (www.ccdc.cam.ac.uk/structures/).

Table S8. Crystal data, data collection, and refinement parameters for **3b** and *ent*-**3b**.

**Compound**	**3b**	*ent*-**3b**
CCDC	2360229	2360228
Formula	$C_{18}H_{16}N_2O$	$C_{18}H_{16}N_2O$
M.w.	276.33	276.33
Crystal system	Monoclinic	Monoclinic
Space group	$P2_1$ (No.4)	$P2_1$ (No. 4)
a [Å]	9.2976 (4)	9.2712 (4)
b [Å]	6.4704 (3)	6.4954 (3)
c [Å]	12.1348 (5)	12.1236 (5)
β [°]	101.724 (2)°	101.916 (1)°
Z	2	2
$V[Å^3]$	714.79 (5)	714.35 (5)
Temperature	120	120
D_x [g cm^{-3}]	1.284	1.285
Wavelength, Å	1.54178	1.54178
Crystal size [mm]	0.41 × 0.11 × 0.07	0.21 × 0.20 × 0.05
Crystal color, shape	bar, ligh yellow	Plate, colourless
μ [mm^{-1}]	0.64	0.64
T_{min}, T_{max}	0.77, 0.96	0.87, 0.97
Measured reflections	23337	15403
Independent diffractions (R_{int}^a)	3019, (0.037)	3011, (0.025)
Observed diffract. [I>2σ(I)]	2931	2978
No. of parameters	191	191
R^b	0.047	0.031
$wR(F^2)$ for all data	0.124	0.083
GOF^c	1.04	1.06
Residual electron density [e/Å^3]	0.22, −0.16	0.19, −0.16
Absolute structure parameter	0.2(5)	-0.02 (15)

$^aR_{int} = \Sigma|F_o^2 - F_{o,mean}^2|/\Sigma F_o^2$; $^bR(F) = \Sigma||F_o| - |F_c||/\Sigma|F_o|$; $wR(F^2) = [\Sigma(w(F_o^2 - F_c^2)^2)/(\Sigma w(F_o^2)^2)]^{½}$; $^c\mathrm{GOF} = [\Sigma(w(F_o^2 - F_c^2)^2)/(N_{diffrs} - N_{params})]^{½}$

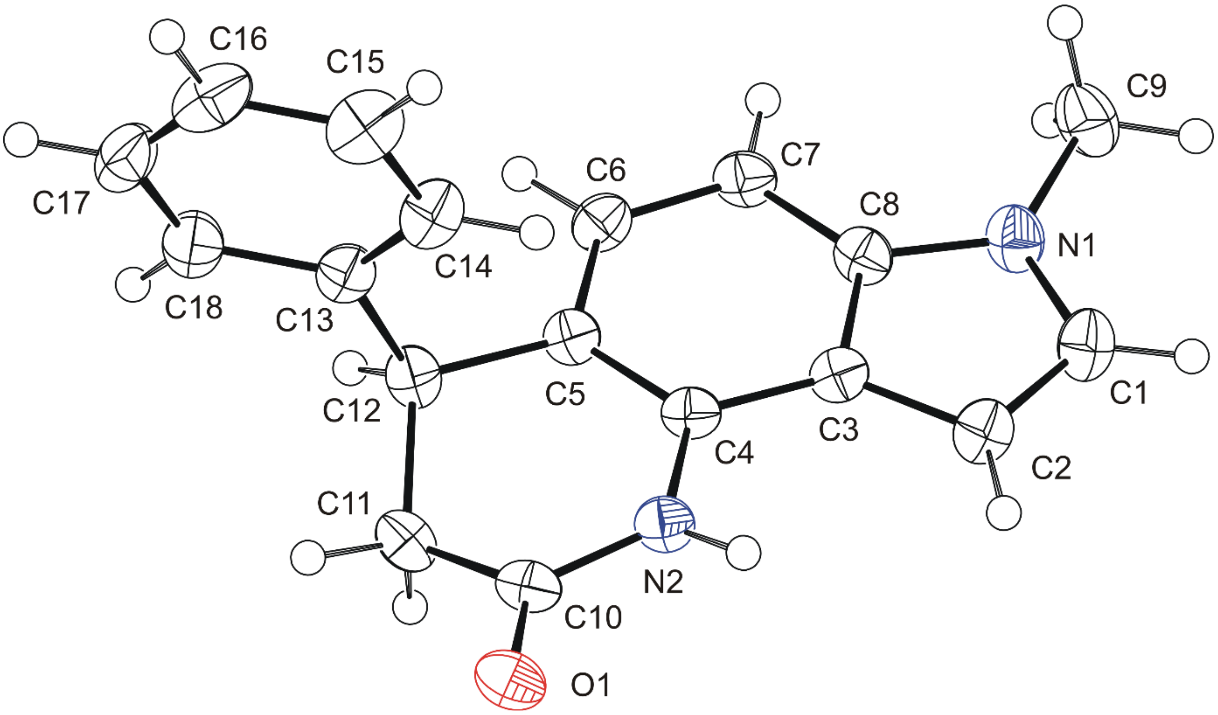

Figure S2. View on the molecule of **3b** with atom numbering schema (CCDC: 2360229). The displacement ellipsoids are drawn on 50% probability level. Chirality on C(12) is *S*.

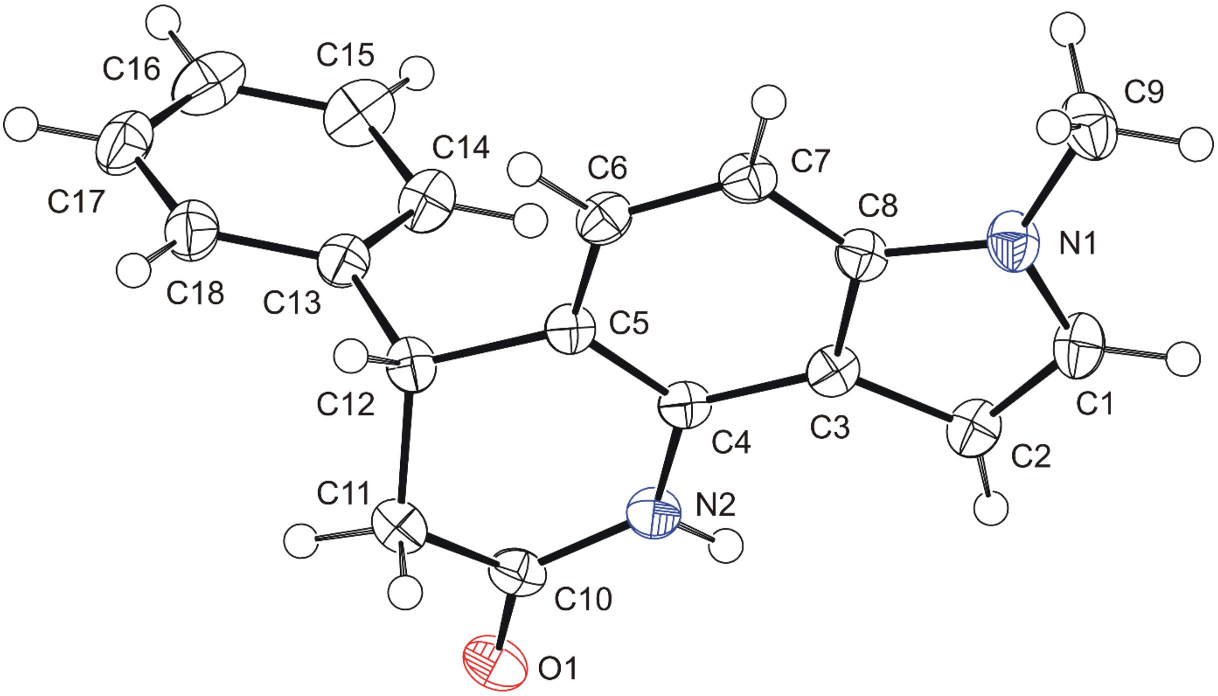

Figure S3. View on the molecule of *ent*-**3b** with atom numbering schema (CCDC: 2360228). The displacement ellipsoids are drawn on 50% probability level. Chirality on C(12) is *R*.

Computational methods

Calculations were performed using Orca 5.0.4,[16] Crest 2.12[17] and xTB 6.6.0[18] software. Geometry optimizations as well as the characterization of the stationary points by full calculation of vibrational frequencies were carried out at the M06-2X/6-311+G* level of theory (298.15 K), including an implicit description of chloroform solvent using conductor-like PCM (CPCM) method as implemented in Orca. Because the M06 functional is known to be more sensitive to the integration grid[19] a dense integration grid was used specifying the !defgrid3 keyword. The relevant conformers for each compound were generated by the default Crest/xTB procedure at the GFN2-xTB/ALPB (chloroform) level. Additionally, the stationary points were recalculated by the same procedure without solvent model. All calculations except the cationic species were performed for a singlet state of the molecules. The cationic species were calculated as doublet. All stationary points were characterized as local minima, having no imaginary frequencies. The condensed Fukui function[20] (f_A^-) was obtained from Hirshfeld atomic charges[21] (as implemented in Orca) using *Equation 1*.

Regiochemistry study – stability of products

For the two series of products (P1_H, P2_H, P3_H) and (P1_Me, P2_Me) Figure S4 relevant conformers were found using Crest/xTB procedure. The structure of each conformer was optimized, and a calculation of vibrational frequencies was carried out at the described level of theory Table S9-12. The obtained Gibbs free energies (at 298.15 K) were compared, and the lowest-energy conformer of each corresponding product was considered. The Gibbs free energy of the lowest-energy product was set to 0 and used as a reference to compare with other products.

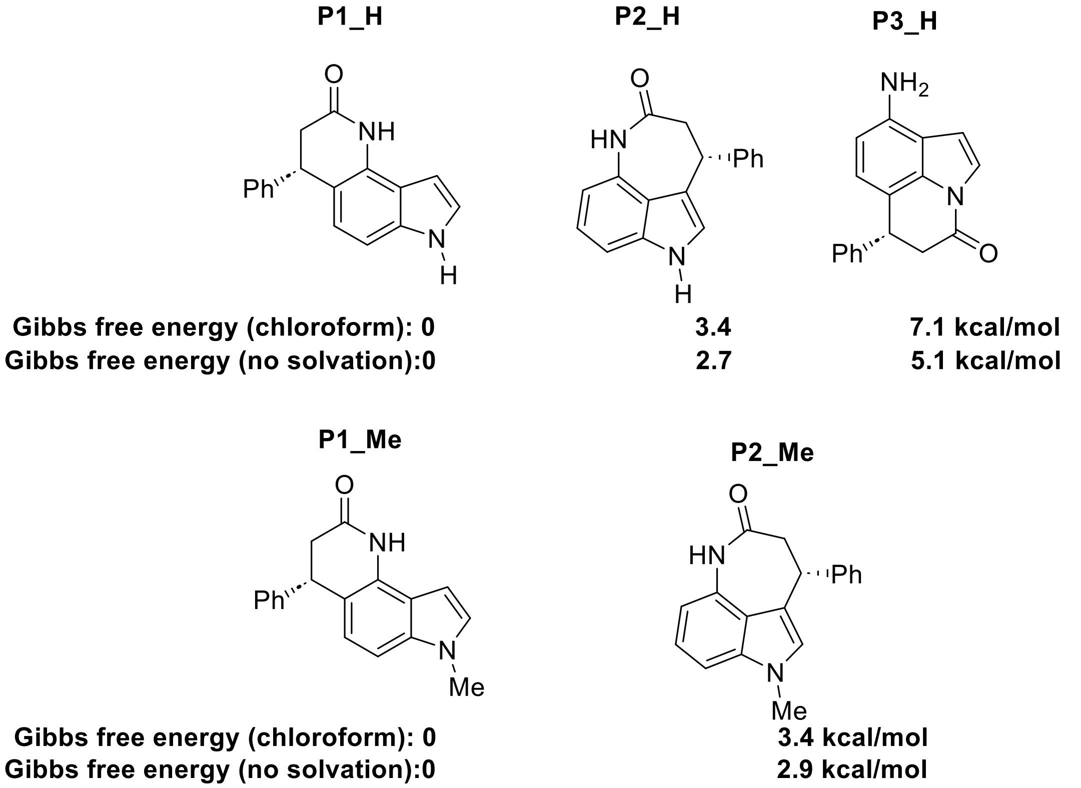

Figure S4. Structures of corresponding regioisomers and comparison of their relative Gibbs free energy at 298.15K.

Table S9. Gibbs free energies of corresponding conformers of P1_Me and P2_Me carried out at the described level of theory at 298.15 K including implicit effect of solvent (chloroform).

Product	Conformer	Gibbs free energy [Eh]	Gibbs free energy [kcal/mol]	Normalized (lowest = 0) [kcal/mol]
**P1_Me**	**1**	**-879.9208572**	**-552152.9777**	**0**
P1_Me	2	-879.9204708	-552152.7352	
P1_Me	3	-879.9195146	-552152.1352	
P1_Me	4	-879.9207492	-552152.9099	
P1_Me	5	-879.9192219	-552151.9515	
P1_Me	6	-879.9208314	-552152.9614	
**P2_Me**	**1**	**-879.9154243**	**-552149.5685**	**3.4**
P2_Me	2	-879.9148925	-552149.2348	
P2_Me	3	-879.9149798	-552149.2895	
P2_Me	4	-879.9149431	-552149.2665	

Table S10. Gibbs free energies of corresponding conformers of P1_H, P2_H and P3_H carried out at the described level of theory at 298.15 K including implicit effect of solvent (chloroform).

Product	Conformer	Gibbs free energy [Eh]	Gibbs free energy [kcal/mol]	Normalized (lowest = 0) [kcal/mol]
P1_H	1	-840.6497008	-527510.2092	
P1_H	2	-840.6491763	-527509.8801	
**P1_H**	**3**	**-840.6497995**	**-527510.2711**	**0**
P1_H	4	-840.6476471	-527508.9205	
P1_H	5	-840.6495062	-527510.0871	
P1_H	6	-840.6476365	-527508.9138	
**P2_H**	**1**	**-840.6444675**	**-527506.9253**	**3.4**
P2_H	2	-840.6439827	-527506.621	
P2_H	3	-840.6443069	-527506.8245	
P2_H	4	-840.641255	-527504.9094	
P3_H	1	-840.6380857	-527502.9207	
P3_H	2	-840.637534	-527502.5745	
**P3_H**	**3**	**-840.6384466**	**-527503.1471**	**7.1**
P3_H	4	-840.6382752	-527503.0396	
P3_H	5	-840.6384466	-527503.1471	
P3_H	6	-840.6372269	-527502.3818	

Table S11. Gibbs free energies of corresponding conformers of P1_H, P2_H and P3_H carried out at the described level of theory at 298.15 K without any solvation model.

Product	Conformer	Gibbs free energy [Eh]	Gibbs free energy [kcal/mol]	Normalized (lowest = 0) [kcal/mol]
P1_H	1	-840.6284007	-527496.8433	
**P1_H**	**2**	**-840.6287155**	**-527497.0408**	**0**
P1_H	3	-840.6266877	-527495.7684	
P1_H	4	-840.6285047	-527496.9086	
P1_H	5	-840.6266868	-527495.7678	
P2_H	1	-840.6235662	-527493.8096	
**P2_H**	**2**	**-840.6243849**	**-527494.3234**	**2.7**
P2_H	3	-840.6234905	-527493.7622	
P3_H	1	-840.6196582	-527491.3574	
P3_H	2	-840.6191121	-527491.0147	
P3_H	3	-840.6188159	-527490.8288	
**P3_H**	**4**	**-840.6205686**	**-527491.9287**	**5.1**
P3_H	5	-840.6205344	-527491.9072	
P3_H	6	-840.6198096	-527491.4524	

Table S12. Gibbs free energies of corresponding conformers of P1_Me and P2_me carried out at the described level of theory at 298.15 K without any solvation model.

Product	Conformer	Gibbs free energy [Eh]	Gibbs free energy [kcal/mol]	Normalized (lowest = 0) [kcal/mol]
P1_Me	1	-879.9012179	-552140.6539	
**P1_Me**	**2**	**-879.9016186**	**-552140.9054**	**0**
P1_Me	3	-879.8995922	-552139.6338	
P1_Me	4	-879.9011241	-552140.595	
P1_Me	5	-879.8996067	-552139.6429	
P1_Me	6	-879.9011561	-552140.6151	
P2_Me	1	-879.8960649	-552137.4204	
P2_Me	2	-879.8970207	-552138.0202	
**P2_Me**	**3**	**-879.897066**	**-552138.0486**	**2.9**
P2_Me	4	-879.8970219	-552138.0209	

Regiochemistry study – Condensed Fukui function as a measure of electrophilicity

The geometries of respective compounds R1_H and R1_Me were optimized at the described level of theory together with calculation of the vibrational frequencies. Two series of optimizations + frequencies were done: one including implicit solvent effect (chloroform) and one without solvation. For the respective neutral (singlet) and cationic (doublet) species with the same geometry Hirshfeld atomic charges were calculated. The condensed Fukui function was calculated using *Equation 1*, using Hirshfeld atomic charges.

$$Electrophilic\ attack: \ f_A^- = q_{N-1}^A - q_N^A$$

Equation S1. The definition of condensed Fukui function used as a measure of electrophilicity. The q_N^A is the Hirshfeld partial atomic charge of atom A in molecule with N electrons (neutral), while q_{N-1}^A is the Hirshfeld partial atomic charge of atom A in molecule with N-1 electrons (cation).

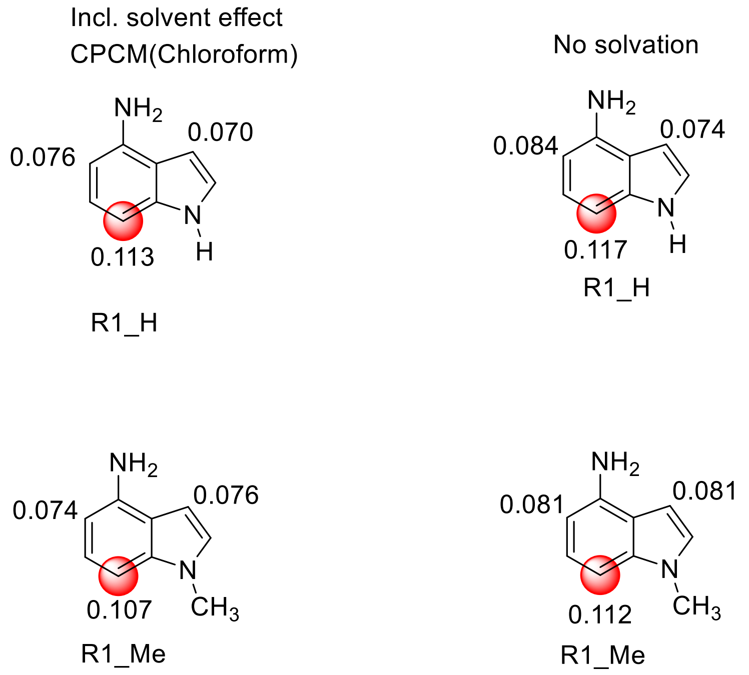

Figure S5. The depicted f_A^- values predict electrophilicity of selected carbon atoms. In all cases the C7 (depicted red) is expected to be the most electrophilic.

Table S13. Hirshfeld atomic charges of optimized structure of R1_H including implicit solvation CPCM(Chloroform).

Atom index	Atom	Charge (neutral)	Charge (cation)	*f-*
**9**	**C**	-0.0851	-0.00872	0.076
**6**	**C**	-0.08039	0.033105	0.113
**13**	**C**	-0.09349	-0.02384	0.070

Table S14. Hirshfeld atomic charges of optimized structure of R1_H without solvation.

Atom index	Atom	Charge (neutral)	Charge (cation)	*f*-
**9**	**C**	-0.09544	-0.01171	0.084
**6**	**C**	-0.08672	0.030564	0.117
**13**	**C**	-0.10475	-0.03115	0.074

Table S15. Hirshfeld atomic charges of optimized structure of R1_Me without solvation.

Atom index	Atom	Charge (neutral)	Charge (cation)	*f*-
**0**	**C**	-0.08517	-0.01126	0.074
**8**	**C**	-0.08165	0.025776	0.107
**12**	**C**	-0.09736	-0.02178	0.076

Table S16. Hirshfeld atomic charges of optimized structure of R1_Me without solvation.

Atom index	Atom	Charge (neutral)	Charge (cation)	*f*-
**0**	**C**	-0.09524	-0.01452	0.081
**8**	**C**	-0.0881	0.024141	0.112
**12**	**C**	-0.10822	-0.02749	0.081

XYZ data

Table S17. XYZ of R1 H chloroform

C	-2.409790000	0.692069000	-0.005960000
N	1.789546000	1.961633000	-0.074744000
H	3.103913000	-0.363004000	-0.006742000
N	-2.081498000	-0.643785000	0.005909000
H	-1.224003000	2.522131000	-0.031719000
C	1.447029000	-1.749730000	-0.001305000
C	0.077564000	-1.939800000	0.004998000
H	2.103973000	-2.612820000	0.000168000
C	1.235953000	0.681207000	-0.006071000
C	2.023157000	-0.464521000	-0.004708000
H	-0.362934000	-2.929884000	0.010239000
H	1.233583000	2.681446000	0.367980000
C	-0.166783000	0.519105000	-0.006204000
C	-1.267545000	1.443398000	-0.014283000
H	2.750771000	2.013106000	0.235562000
C	-0.714557000	-0.782162000	0.004253000
H	-2.743479000	-1.403861000	0.010546000
H	-3.444793000	0.998046000	-0.010044000

Table S18. XYZ of R1 H.

C	-2.408241000	0.695112000	-0.005649000
N	1.784608000	1.960655000	-0.062233000
H	3.102073000	-0.365101000	-0.013647000
N	-2.080653000	-0.642598000	0.011549000
H	-1.226954000	2.519499000	-0.053196000
C	1.446987000	-1.748056000	-0.004589000
C	0.079047000	-1.937742000	0.007360000
H	2.103427000	-2.610995000	-0.005940000
C	1.234161000	0.678774000	-0.005867000
C	2.021260000	-0.464550000	-0.008124000
H	-0.357618000	-2.929433000	0.015134000
H	1.228882000	2.673904000	0.387852000
C	-0.166621000	0.517484000	-0.007893000
C	-1.266519000	1.441634000	-0.018483000
H	2.749446000	2.012477000	0.231063000
C	-0.712154000	-0.782003000	0.007172000
H	-2.739615000	-1.401984000	0.016518000
H	-3.441407000	1.005495000	-0.013151000

Table S19. XYZ of R1 Me chloroform.

C	-2.103421000	-1.003449000	-0.077822000
C	-1.146666000	-2.036009000	-0.113409000
C	0.589513000	-0.428805000	-0.010700000
C	-0.346165000	0.628889000	0.033318000
C	-1.725843000	0.333545000	-0.006338000
N	-2.663131000	1.363835000	0.090679000
H	-3.159696000	-1.252098000	-0.107531000
H	0.945210000	-2.576425000	-0.113742000
C	0.212563000	-1.778421000	-0.082753000
H	-1.492752000	-3.062419000	-0.170662000
H	2.614087000	2.106280000	0.157333000
C	1.726551000	1.492184000	0.109471000
C	0.405365000	1.850058000	0.110632000
N	1.845822000	0.123787000	0.035806000
H	0.026248000	2.859387000	0.167882000
C	3.081124000	-0.633516000	0.015426000
H	3.156071000	-1.217605000	-0.903931000
H	3.918825000	0.059582000	0.064113000
H	3.128673000	-1.309745000	0.871257000
H	-2.358739000	2.245292000	-0.301067000
H	-3.583487000	1.120074000	-0.250190000

Table S20. XYZ of R1 Me.

C	-2.100795000	-1.003355000	-0.075399000
C	-1.146011000	-2.034359000	-0.109617000
C	0.587886000	-0.428543000	-0.011375000
C	-0.345759000	0.627579000	0.035436000
C	-1.722936000	0.331727000	-0.006950000
N	-2.658474000	1.364455000	0.078214000
H	-3.156493000	-1.254343000	-0.101854000
H	0.942624000	-2.575739000	-0.115268000
C	0.211756000	-1.776445000	-0.082666000
H	-1.491543000	-3.060716000	-0.163974000
H	2.608696000	2.111373000	0.161489000
C	1.724032000	1.494182000	0.110135000
C	0.405014000	1.848318000	0.114612000
N	1.845305000	0.124722000	0.033242000
H	0.028309000	2.856844000	0.188474000
C	3.075059000	-0.632245000	0.011774000
H	3.152568000	-1.216846000	-0.907920000
H	3.916940000	0.056516000	0.060594000
H	3.125507000	-1.309745000	0.867406000
H	-2.350531000	2.241183000	-0.317364000
H	-3.581002000	1.119856000	-0.251218000

Table S21. XYZ of P1 Me chloroform c 1.

O	-1.698137000	3.295006000	-0.297824000
N	0.048808000	1.863785000	-0.354678000
H	0.427961000	2.380184000	-1.138550000
N	4.010134000	-0.633643000	-0.299276000
C	-2.310288000	-0.568580000	0.348875000
C	0.809125000	-1.297850000	1.428262000
H	0.286227000	-1.988512000	2.082736000
C	-1.118493000	2.332835000	0.177266000
C	0.748972000	0.745079000	0.129220000
C	0.116296000	-0.159388000	0.971676000
C	-1.333269000	0.081602000	1.323912000
H	-1.528727000	-0.339724000	2.313983000
C	2.080380000	0.522502000	-0.265590000
C	3.018474000	1.225786000	-1.092219000
H	2.871413000	2.154589000	-1.622409000
C	2.736093000	-0.635694000	0.211841000
C	2.117045000	-1.560244000	1.063064000
H	2.637750000	-2.440470000	1.422207000
C	-1.954985000	-0.922664000	-0.953237000
H	-0.934885000	-0.779210000	-1.294621000
C	-2.896850000	-1.475228000	-1.820632000
H	-2.602963000	-1.745022000	-2.829211000
C	4.170188000	0.485143000	-1.080174000
H	5.115261000	0.664769000	-1.571922000
C	-3.628539000	-0.782489000	0.764091000

H	-3.916866000	-0.514072000	1.776955000
C	-1.586887000	1.597018000	1.412081000
H	-2.641095000	1.826538000	1.564411000
H	-1.023514000	2.008871000	2.257347000
C	5.007777000	-1.652900000	-0.037238000
H	5.220352000	-1.711183000	1.031831000
H	5.921568000	-1.392044000	-0.567367000
H	4.658633000	-2.626405000	-0.386141000
C	-4.204968000	-1.684184000	-1.396856000
H	-4.935219000	-2.117896000	-2.070993000
C	-4.569153000	-1.336916000	-0.096716000
H	-5.584645000	-1.501411000	0.246901000

Table S22. XYZ of P1 Me chloroform c 2.

O	-1.035764000	4.006446000	0.114670000
N	0.519782000	2.375673000	0.257577000
H	1.229553000	3.086750000	0.381938000
N	4.011661000	-0.714523000	-0.090832000
C	-2.496041000	-0.627454000	0.140259000
C	0.382940000	-1.331116000	0.287862000
H	-0.368392000	-2.107642000	0.379900000
C	-0.756576000	2.819501000	0.071165000
C	0.912321000	1.027260000	0.196887000
C	-0.035089000	0.014817000	0.303238000
C	-1.481577000	0.428963000	0.525457000
H	-1.606068000	0.634946000	1.596598000
C	2.274527000	0.706962000	0.064754000
C	3.479364000	1.473229000	-0.075540000
H	3.585923000	2.546959000	-0.111073000
C	2.647538000	-0.656397000	0.051738000
C	1.713200000	-1.692335000	0.165679000
H	2.014289000	-2.733628000	0.156348000
C	-2.544412000	-1.126640000	-1.165340000
H	-1.826926000	-0.775016000	-1.901353000
C	-3.490928000	-2.078463000	-1.526857000
H	-3.514335000	-2.456657000	-2.543094000
C	4.501808000	0.566944000	-0.165842000
H	5.563636000	0.728761000	-0.281895000
C	-3.413652000	-1.105020000	1.074132000
H	-3.384599000	-0.727988000	2.092033000
C	-1.764941000	1.736176000	-0.231029000
H	-1.719142000	1.554591000	-1.311656000
H	-2.758165000	2.121740000	-0.001477000
C	4.784835000	-1.939329000	-0.155751000
H	4.668042000	-2.513960000	0.764918000
H	5.834796000	-1.684124000	-0.284315000
H	4.460615000	-2.549073000	-1.001150000
C	-4.407081000	-2.548070000	-0.585460000
H	-5.145330000	-3.290677000	-0.867199000

C	-4.365829000	-2.059663000	0.715847000
H	-5.072256000	-2.419682000	1.455824000

Table S23. XYZ of P1 Me chloroform c 3.

O	-1.469553000	3.549093000	-0.408688000
N	0.155499000	1.983981000	-0.400984000
H	0.617511000	2.491265000	-1.145350000
N	3.923207000	-0.786551000	-0.227155000
C	-2.355718000	-0.468690000	0.339915000
C	0.641923000	-1.212172000	1.421810000
H	0.059221000	-1.855783000	2.073823000
C	-1.000114000	2.530206000	0.072875000
C	0.749297000	0.811743000	0.097999000
C	0.035728000	-0.037679000	0.931727000
C	-1.410847000	0.291456000	1.259912000
H	-1.609072000	-0.056403000	2.276971000
C	2.073785000	0.494780000	-0.255974000
C	3.078500000	1.125662000	-1.062098000
H	3.008493000	2.055466000	-1.606319000
C	2.638860000	-0.698120000	0.249453000
C	1.937585000	-1.566781000	1.095803000
H	2.389447000	-2.473141000	1.482216000
C	-2.704454000	-0.000159000	-0.930083000
H	-2.335274000	0.954168000	-1.290897000
C	-3.538859000	-0.746255000	-1.758661000
H	-3.800499000	-0.363500000	-2.739148000
C	4.177901000	0.310744000	-1.012939000
H	5.144819000	0.421042000	-1.482097000
C	-2.867879000	-1.699803000	0.757263000
H	-2.613920000	-2.073475000	1.744989000
C	-1.618900000	1.811716000	1.250727000
H	-2.675307000	2.078225000	1.287244000
H	-1.140212000	2.237216000	2.140221000
C	4.842082000	-1.868800000	0.066835000
H	4.437600000	-2.818830000	-0.287090000
H	5.021516000	-1.934327000	1.141549000
H	5.785395000	-1.673671000	-0.439367000
C	-4.038355000	-1.974263000	-1.333057000
H	-4.689698000	-2.552548000	-1.978837000
C	-3.700307000	-2.449358000	-0.069107000
H	-4.088776000	-3.400831000	0.277642000

Table S24. XYZ of P1 Me chloroform c 4.

O	-1.834369000	3.210301000	0.143150000
N	-0.046816000	1.855472000	-0.126309000
H	0.293483000	2.470307000	-0.855105000
N	3.994786000	-0.481123000	-0.472251000
C	-2.288923000	-0.708693000	0.352688000

C	0.885295000	-1.443925000	1.284068000
H	0.410847000	-2.219940000	1.876843000
C	-1.208573000	2.222667000	0.490595000
C	0.708176000	0.720089000	0.213642000
C	0.136809000	-0.292292000	0.972714000
C	-1.310852000	-0.146619000	1.381124000
H	-1.470066000	-0.688503000	2.317618000
C	2.028680000	0.589516000	-0.251598000
C	2.907961000	1.409931000	-1.032487000
H	2.707851000	2.382220000	-1.456851000
C	2.742528000	-0.585092000	0.080844000
C	2.187880000	-1.615537000	0.851158000
H	2.752140000	-2.507196000	1.099226000
C	-3.598509000	-0.987118000	0.757840000
H	-3.877683000	-0.832366000	1.796693000
C	-4.542484000	-1.460335000	-0.146340000
H	-5.551222000	-1.675318000	0.189143000
C	4.084882000	0.717459000	-1.137468000
H	5.002722000	0.983960000	-1.641109000
C	-1.945789000	-0.916809000	-0.983514000
H	-0.933123000	-0.720167000	-1.320303000
C	-1.611787000	1.338499000	1.649110000
H	-2.667567000	1.510971000	1.855781000
H	-1.035575000	1.674096000	2.519116000
C	5.037026000	-1.482265000	-0.354099000
H	4.706714000	-2.428775000	-0.785830000
H	5.297222000	-1.639153000	0.694340000
H	5.917993000	-1.135525000	-0.890611000
C	-4.190582000	-1.661059000	-1.480330000
H	-4.923750000	-2.030025000	-2.188930000
C	-2.891111000	-1.388515000	-1.893937000
H	-2.606550000	-1.544070000	-2.928939000

Table S25. XYZ of P1 Me chloroform c 5.

O	-1.467548000	3.466327000	-0.571696000
N	0.088233000	1.829938000	-0.564866000
H	0.519897000	2.265058000	-1.370634000
N	3.908908000	-0.852453000	-0.168315000
C	-2.352920000	-0.407483000	0.317973000
C	0.678262000	-1.110510000	1.614052000
H	0.133174000	-1.665741000	2.370660000
C	-1.002071000	2.466976000	-0.047997000
C	0.716783000	0.725314000	0.035933000
C	0.033687000	-0.029394000	0.981037000
C	-1.413142000	0.333245000	1.268644000
H	-1.651154000	0.004187000	2.284203000
C	2.037268000	0.388911000	-0.308677000
C	3.010522000	0.926774000	-1.215044000
H	2.908774000	1.777295000	-1.872152000

C	2.635913000	-0.722246000	0.329252000
C	1.975853000	-1.478761000	1.305325000
H	2.459275000	-2.311878000	1.802562000
C	-3.103337000	0.235267000	-0.667527000
H	-3.069689000	1.313143000	-0.780906000
C	-3.928766000	-0.494129000	-1.525553000
H	-4.500224000	0.027121000	-2.285819000
C	4.124248000	0.139789000	-1.093745000
H	5.076095000	0.203119000	-1.600757000
C	-2.461827000	-1.798840000	0.431430000
H	-1.893265000	-2.317368000	1.197364000
C	-1.557743000	1.856158000	1.220584000
H	-2.592045000	2.182979000	1.327961000
H	-0.986526000	2.290119000	2.048705000
C	4.856491000	-1.871044000	0.240121000
H	5.083629000	-1.777053000	1.303744000
H	5.773866000	-1.745497000	-0.331550000
H	4.450844000	-2.865950000	0.047810000
C	-4.023566000	-1.874723000	-1.404919000
H	-4.667479000	-2.439784000	-2.069562000
C	-3.287890000	-2.526858000	-0.415220000
H	-3.359816000	-3.603303000	-0.303521000

Table S26. XYZ of P1 Me chloroform c 6.

O	-1.744832000	3.276186000	-0.170037000
N	0.005090000	1.853452000	-0.294837000
H	0.375348000	2.399716000	-1.062582000
N	3.992788000	-0.600817000	-0.353395000
C	-2.312250000	-0.623898000	0.369557000
C	0.824365000	-1.338683000	1.404781000
H	0.318057000	-2.050036000	2.050067000
C	-1.156818000	2.300715000	0.266540000
C	0.724179000	0.732150000	0.153563000
C	0.112948000	-0.198415000	0.983226000
C	-1.336240000	0.015401000	1.353489000
H	-1.518454000	-0.434008000	2.333748000
C	2.051084000	0.532159000	-0.267372000
C	2.968960000	1.263516000	-1.092073000
H	2.803813000	2.201580000	-1.600190000
C	2.726653000	-0.628329000	0.176161000
C	2.129944000	-1.577778000	1.015762000
H	2.665473000	-2.459588000	1.348319000

C	-1.956809000	-0.962897000	-0.936428000
H	-0.937773000	-0.811372000	-1.277416000
C	-2.897161000	-1.510568000	-1.808773000
H	-2.602816000	-1.768770000	-2.820255000
C	4.128914000	0.536017000	-1.112484000
H	5.064434000	0.737194000	-1.614014000
C	-3.629473000	-0.847618000	0.783455000
H	-3.918138000	-0.591558000	1.799439000
C	-1.608689000	1.524507000	1.482636000
H	-2.664736000	1.736565000	1.647132000
H	-1.046301000	1.921713000	2.335594000
C	5.005655000	-1.613603000	-0.127713000
H	5.232466000	-1.694052000	0.936999000
H	5.909831000	-1.330068000	-0.662693000
H	4.663854000	-2.583229000	-0.494327000
C	-4.203901000	-1.729558000	-1.386205000
H	-4.932860000	-2.159674000	-2.064036000
C	-4.568434000	-1.397029000	-0.082173000
H	-5.582913000	-1.569461000	0.260534000

Table 1 XYZ of P2 Me chloroform c 1.

O	-2.028947000	-2.679238000	0.789780000
N	-0.002438000	-2.199850000	-0.027731000
H	-0.149503000	-3.017360000	-0.608347000
N	2.553085000	1.747672000	0.279767000
C	-1.931908000	0.763311000	0.233665000
C	3.482452000	-1.544960000	-1.114309000
H	-4.858131000	1.120654000	-2.304427000
C	-1.035554000	-1.967857000	0.839749000
C	1.218706000	-1.525594000	-0.214619000
C	2.264516000	-2.192349000	-0.841113000
C	-0.827810000	0.566678000	1.264237000
H	4.269927000	-2.104587000	-1.606197000
C	1.401015000	-0.177168000	0.141741000
C	0.559886000	0.807502000	0.755827000
H	4.636254000	0.275486000	-0.988293000
C	2.639726000	0.446243000	-0.143227000
C	3.697136000	-0.220874000	-0.772521000
H	-3.427049000	1.100603000	1.741679000
C	-1.700489000	0.648781000	-1.137410000
H	-5.287158000	1.332238000	0.132980000
C	-2.749584000	0.775305000	-2.046943000

H	-0.694534000	0.466315000	-1.502047000
C	1.304091000	1.953855000	0.820613000
H	2.132630000	-3.230506000	-1.127986000
C	-3.234946000	1.008560000	0.676081000
H	-2.552272000	0.684978000	-3.109642000
C	-0.917608000	-0.858797000	1.865780000
H	-0.040865000	-1.039825000	2.494644000
H	-1.809527000	-0.942658000	2.485820000
C	3.618757000	2.725381000	0.176895000
H	3.898773000	2.875338000	-0.867483000
H	4.495257000	2.396113000	0.738225000
H	3.267657000	3.669717000	0.588279000
C	-4.043030000	1.019936000	-1.596429000
H	1.044311000	2.921810000	1.226135000
C	-4.283299000	1.137628000	-0.228624000
H	-1.018747000	1.262512000	2.086668000

Table S28. XYZ of P2 Me chloroform c 2.

O	-1.307504000	-3.625558000	0.958349000
N	0.726479000	-2.729556000	0.709458000
H	1.050027000	-3.587246000	1.141528000
N	2.032877000	1.679960000	-0.316707000
C	-2.176312000	0.797062000	0.060726000
C	4.087772000	-1.381451000	-0.091652000
H	-4.953783000	3.295052000	-1.018435000
C	-0.629258000	-2.703032000	0.527368000
C	1.728893000	-1.814727000	0.336245000
C	3.039510000	-2.263200000	0.224968000
C	-1.093331000	-0.174984000	0.484791000
H	5.094897000	-1.775758000	-0.165582000
C	1.467558000	-0.449401000	0.126225000
C	0.283478000	0.355271000	0.200083000
H	4.675862000	0.645174000	-0.549973000
C	2.543015000	0.413177000	-0.195324000
C	3.864669000	-0.032834000	-0.310920000
H	-1.685809000	0.705459000	-2.033861000
C	-3.022441000	1.370682000	1.008361000
H	-3.457938000	2.281066000	-2.720503000
C	-4.018460000	2.268423000	0.624652000
H	-2.902565000	1.113060000	2.056369000
C	0.677028000	1.636233000	-0.080251000
H	3.254559000	-3.313722000	0.390571000
C	-2.343537000	1.137388000	-1.285180000
H	-4.667237000	2.706262000	1.375431000
C	-1.268765000	-1.546889000	-0.212824000
H	-2.326664000	-1.797710000	-0.282180000
H	-0.861279000	-1.496225000	-1.227422000
C	2.813688000	2.862174000	-0.624122000
H	3.306212000	2.754367000	-1.592218000

H	3.570291000	3.030348000	0.144865000
H	2.146961000	3.721509000	-0.658881000
C	-4.179198000	2.598719000	-0.716774000
H	0.087890000	2.540093000	-0.145152000
C	-3.338219000	2.028548000	-1.672531000
H	-1.205800000	-0.337533000	1.563112000

Table S29. XYZ of P2 Me chloroform c 3.

O	-1.328995000	-3.624315000	0.940179000
N	0.709682000	-2.724582000	0.748633000
H	1.022147000	-3.579452000	1.194320000
N	2.034668000	1.687492000	-0.242653000
C	-2.182075000	0.791539000	-0.000462000
C	4.090500000	-1.367314000	0.051581000
H	-4.932502000	3.278388000	-1.170672000
C	-0.640007000	-2.702132000	0.525748000
C	1.720474000	-1.807401000	0.404740000
C	3.035257000	-2.251973000	0.335102000
C	-1.109998000	-0.176172000	0.459604000
H	5.100598000	-1.758747000	0.010083000
C	1.461690000	-0.443325000	0.183255000
C	0.273327000	0.357618000	0.216440000
H	4.686911000	0.660839000	-0.388880000
C	2.544494000	0.422539000	-0.102539000
C	3.870503000	-0.019496000	-0.175561000
H	-2.965172000	1.116219000	1.972129000
C	-2.311521000	1.124785000	-1.352231000
H	-4.712740000	2.702626000	1.233337000
C	-3.296500000	2.011965000	-1.772173000
H	-1.632107000	0.690152000	-2.079693000
C	0.671772000	1.639590000	-0.051729000
H	3.248099000	-3.301626000	0.508857000
C	-3.055831000	1.368309000	0.919858000
H	-3.387043000	2.258879000	-2.824402000
C	-1.259436000	-1.550768000	-0.238605000
H	-2.313890000	-1.805043000	-0.340137000
H	-0.820410000	-1.502411000	-1.240068000
C	2.821625000	2.871648000	-0.525880000
H	2.153647000	3.728654000	-0.585714000
H	3.347346000	2.763215000	-1.476371000
H	3.550886000	3.044380000	0.268103000
C	-4.165364000	2.585302000	-0.843595000
H	0.082238000	2.541534000	-0.136746000
C	-4.042119000	2.262192000	0.503558000
H	-1.255213000	-0.335867000	1.534450000

Table S30. XYZ of P2 Me chloroform c 4.

O	-1.328615000	-3.626428000	0.929171000
N	0.710151000	-2.730551000	0.721618000
H	1.025053000	-3.588819000	1.158957000
N	2.037286000	1.682105000	-0.264169000
C	-2.179997000	0.796855000	0.022803000
C	4.087139000	-1.379836000	-0.005889000
H	-4.940124000	3.293310000	-1.103348000
C	-0.641729000	-2.703483000	0.512954000
C	1.719785000	-1.814604000	0.371184000
C	3.032498000	-2.262617000	0.285594000
C	-1.104839000	-0.175510000	0.465510000
H	5.095590000	-1.773958000	-0.060069000
C	1.462592000	-0.448826000	0.158641000
C	0.277122000	0.355587000	0.209007000
H	4.684456000	0.648482000	-0.444737000
C	2.544706000	0.414987000	-0.135653000
C	3.868568000	-0.030460000	-0.225057000
H	-2.946169000	1.105294000	2.004625000
C	-2.321131000	1.141040000	-1.325089000
H	-4.699862000	2.697689000	1.293933000
C	-3.309576000	2.031723000	-1.729227000
H	-1.648137000	0.712259000	-2.061923000
C	0.676439000	1.637507000	-0.058364000
H	3.244170000	-3.313613000	0.452528000
C	-3.045772000	1.366061000	0.955271000
H	-3.409125000	2.287250000	-2.778582000
C	-1.266317000	-1.546112000	-0.237993000
H	-2.322617000	-1.796764000	-0.328823000
H	-0.838697000	-1.493628000	-1.244185000
C	2.825275000	2.864110000	-0.553261000
H	3.333384000	2.760649000	-1.513650000
H	3.569849000	3.026188000	0.228736000
H	2.161784000	3.725631000	-0.593895000
C	-4.170339000	2.597470000	-0.788562000
H	0.088786000	2.541600000	-0.132840000
C	-4.035576000	2.263294000	0.554798000
H	-1.237997000	-0.340322000	1.541132000

Table S31. XYZ of P1 H chloroform c 1.

O	-1.091434000	-3.320296000	0.304985000
N	0.510662000	-1.728685000	0.354941000
H	0.939610000	-2.207696000	1.136854000
N	4.201399000	1.140330000	0.295789000
C	-2.100067000	0.458346000	-0.344521000
C	0.932342000	1.510118000	-1.400167000
H	-2.560049000	1.478784000	2.867344000
C	-0.609030000	-2.306699000	-0.171949000

C	1.089329000	-0.539887000	-0.122966000
C	0.361497000	0.302561000	-0.950951000
C	-1.054356000	-0.085452000	-1.311988000
H	2.638003000	2.829693000	-1.397798000
C	2.397048000	-0.187893000	0.260558000
C	3.407375000	-0.807793000	1.071062000
H	-1.284457000	0.315267000	-2.302983000
C	2.928979000	1.033814000	-0.210056000
C	2.209793000	1.898897000	-1.044629000
H	-0.785731000	0.702610000	1.343968000
C	-1.804902000	0.784188000	0.979937000
H	-3.661076000	0.343494000	-1.818519000
C	-2.807171000	1.229445000	1.841126000
H	5.444566000	-0.060702000	1.544936000
C	4.484253000	0.035770000	1.061984000
H	0.336920000	2.150086000	-2.044034000
C	-3.419484000	0.591458000	-0.788440000
H	-5.436475000	1.142797000	-0.299031000
C	-1.151410000	-1.618896000	-1.404107000
H	-2.176908000	-1.954058000	-1.556585000
H	-0.549350000	-1.968814000	-2.250686000
H	3.352697000	-1.752873000	1.590002000
C	-4.420562000	1.039357000	0.066431000
C	-4.116361000	1.358550000	1.388770000
H	4.832891000	1.907929000	0.127317000
H	-4.893852000	1.709117000	2.058382000

Table S32. XYZ of P1 H chloroform c 2.

O	-0.338587000	-3.956492000	-0.142696000
N	1.024832000	-2.161671000	-0.275292000
H	1.806447000	-2.790420000	-0.411749000
N	4.158674000	1.274966000	0.125837000
C	-2.305113000	0.487332000	-0.130824000
C	0.479774000	1.506668000	-0.232204000
H	-3.557061000	2.154602000	2.559071000
C	-0.193503000	-2.746325000	-0.087352000
C	1.267151000	-0.779210000	-0.193506000
C	0.213460000	0.122828000	-0.277992000
C	-1.176733000	-0.446804000	-0.513359000
H	1.947327000	3.077037000	-0.070595000
C	2.586699000	-0.311935000	-0.056691000
C	3.869255000	-0.947618000	0.064376000
H	-1.268819000	-0.656400000	-1.587230000
C	2.805930000	1.083132000	-0.014894000
C	1.761110000	2.009880000	-0.103365000
H	-1.690227000	0.671546000	1.925070000
C	-2.428341000	0.957010000	1.180846000
H	-3.165564000	0.526504000	-2.097409000
C	-3.476143000	1.797676000	1.538107000

H	5.864006000	0.004124000	0.282251000
C	4.791581000	0.056535000	0.172628000
H	-0.352964000	2.197287000	-0.306607000
C	-3.251909000	0.881315000	-1.074807000
H	-5.032516000	2.020713000	-1.468736000
C	-1.315317000	-1.785124000	0.228676000
H	-1.289539000	-1.613035000	1.311462000
H	-2.259682000	-2.275638000	-0.006869000
H	4.088021000	-2.004676000	0.076612000
C	-4.305076000	1.724535000	-0.720848000
C	-4.420178000	2.184069000	0.586465000
H	4.617190000	2.170753000	0.187397000
H	-5.237310000	2.840381000	0.864671000

Table S33. XYZ of P1 H chloroform c 3.

O	-1.308470000	-3.234535000	-0.357189000
N	0.374681000	-1.779554000	0.034769000
H	0.740682000	-2.412658000	0.734916000
N	4.203859000	0.820982000	0.668320000
C	-2.054107000	0.654419000	-0.347937000
C	1.073879000	1.674587000	-1.115980000
H	-5.356683000	1.483242000	-0.255098000
C	-0.748537000	-2.185442000	-0.628096000
C	1.043156000	-0.566759000	-0.204566000
C	0.411724000	0.448277000	-0.908772000
C	-1.008880000	0.224038000	-1.372632000
H	2.845805000	2.863395000	-0.801267000
C	2.338937000	-0.365648000	0.305064000
C	3.257878000	-1.175103000	1.054614000
H	-1.179180000	0.808510000	-2.281042000
C	2.964367000	0.881325000	0.079999000
C	2.346757000	1.915966000	-0.634302000
H	-3.581008000	0.848585000	-1.849322000
C	-3.354619000	0.919269000	-0.788772000
H	-0.774810000	0.565275000	1.382623000
C	-4.356122000	1.274929000	0.107973000
H	5.294994000	-0.646802000	1.765061000
C	4.375463000	-0.411079000	1.251501000
H	0.553635000	2.451199000	-1.668195000
C	-1.779136000	0.752768000	1.016666000
H	-2.549641000	1.179195000	2.975961000
C	-1.193347000	-1.259684000	-1.737654000
H	-2.229358000	-1.494133000	-1.980431000
H	-0.576554000	-1.496130000	-2.612395000
H	3.119087000	-2.188527000	1.399893000
C	-2.781809000	1.106267000	1.918988000
C	-4.071803000	1.368243000	1.469395000
H	4.884196000	1.565039000	0.672446000
H	-4.849290000	1.646919000	2.171986000

Table S34. XYZ of P1 H chloroform c 4.

O	-0.998163000	-3.391132000	0.338244000
N	0.455560000	-1.663716000	0.459081000
H	0.871933000	-2.091739000	1.276518000
N	4.134777000	1.219451000	0.238250000
C	-2.095871000	0.344828000	-0.340224000
C	0.921736000	1.383810000	-1.594121000
H	-4.919039000	-0.487121000	1.372166000
C	-0.563563000	-2.354361000	-0.134857000
C	1.038826000	-0.506612000	-0.086656000
C	0.331246000	0.243361000	-1.016566000
C	-1.078278000	-0.208096000	-1.345020000
H	2.634212000	2.692353000	-1.694105000
C	2.333879000	-0.113918000	0.293704000
C	3.320838000	-0.637752000	1.196031000
H	-1.341823000	0.174952000	-2.336042000
C	2.878540000	1.052157000	-0.291768000
C	2.190187000	1.811265000	-1.245733000
H	-3.292834000	-1.448759000	-0.172658000
C	-3.167564000	-0.416664000	0.132528000
H	-1.155485000	2.279592000	-0.239840000
C	-4.099851000	0.127984000	1.015916000
H	5.340528000	0.168197000	1.647949000
C	4.395566000	0.205469000	1.127790000
H	0.352481000	1.941484000	-2.331488000
C	-1.984255000	1.668595000	0.101168000
H	-2.807135000	3.246334000	1.299142000
C	-1.069155000	-1.738302000	-1.420774000
H	-2.040951000	-2.171005000	-1.649835000
H	-0.383271000	-2.045482000	-2.217929000
H	3.251776000	-1.519600000	1.814837000
C	-2.916314000	2.217017000	0.975005000
C	-3.981147000	1.446670000	1.438543000
H	4.771788000	1.964648000	0.003029000
H	-4.707094000	1.870576000	2.123447000

Table S35. XYZ of P1 H chloroform c 5.

O	-0.877857000	-3.277818000	0.902121000
N	0.624003000	-1.610920000	0.639411000
H	1.106062000	-1.933079000	1.469388000
N	4.160550000	1.369186000	-0.029629000
C	-2.105652000	0.299013000	-0.327077000
C	0.839392000	1.286934000	-1.663112000
H	-5.479202000	0.760437000	-0.232207000
C	-0.470529000	-2.329925000	0.251540000
C	1.129511000	-0.499524000	-0.056587000
C	0.340511000	0.151513000	-0.993785000

C	-1.063764000	-0.355271000	-1.228977000
H	2.475242000	2.665039000	-1.940820000
C	2.427053000	-0.028805000	0.215702000
C	3.483865000	-0.451121000	1.091364000
H	-1.344639000	-0.140087000	-2.263794000
C	2.885103000	1.117166000	-0.472383000
C	2.103476000	1.790375000	-1.420028000
H	-3.714361000	-0.211351000	-1.659713000
C	-3.448891000	0.254122000	-0.714228000
H	-0.743881000	0.965451000	1.202266000
C	-4.443983000	0.799542000	0.089109000
H	5.488806000	0.462164000	1.370840000
C	4.514968000	0.428496000	0.906799000
H	0.197470000	1.778154000	-2.387884000
C	-1.780564000	0.905155000	0.886703000
H	-2.505629000	1.916359000	2.637816000
C	-1.085410000	-1.884591000	-1.056155000
H	-2.096855000	-2.286371000	-1.109536000
H	-0.494065000	-2.347362000	-1.854717000
H	3.488664000	-1.294435000	1.765325000
C	-2.776657000	1.448918000	1.697359000
C	-4.109496000	1.398787000	1.302627000
H	4.747546000	2.125956000	-0.344614000
H	-4.882554000	1.825884000	1.931713000

Table S36. XYZ of P1 H chloroform c 6.

O	-0.959926000	-3.472855000	0.180213000
N	0.485219000	-1.747850000	0.369629000
H	0.925814000	-2.225329000	1.146001000
N	4.120194000	1.190602000	0.337799000
C	-2.101045000	0.376512000	-0.359428000
C	0.905633000	1.433480000	-1.483373000
H	-4.352617000	-0.505546000	2.035309000
C	-0.547393000	-2.402484000	-0.235974000
C	1.045083000	-0.548244000	-0.103632000
C	0.326767000	0.250341000	-0.982360000
C	-1.081202000	-0.186018000	-1.349786000
H	2.602290000	2.764209000	-1.502336000
C	2.334266000	-0.162964000	0.305417000
C	3.327451000	-0.734253000	1.171085000
H	-1.319665000	0.227780000	-2.333869000
C	2.865627000	1.044407000	-0.202054000
C	2.167620000	1.853784000	-1.106509000
H	-2.772419000	-1.507967000	0.464314000
C	-2.863664000	-0.427809000	0.488329000
H	-1.703826000	2.406999000	-0.952596000
C	-3.772911000	0.139139000	1.383789000
H	5.338145000	0.061774000	1.675997000
C	4.392808000	0.123661000	1.158974000

H	0.337463000	2.026437000	-2.192955000
C	-2.281403000	1.763607000	-0.296254000
H	-3.316040000	3.407637000	0.614669000
C	-1.106413000	-1.712231000	-1.461717000
H	-2.106252000	-2.104666000	-1.646045000
H	-0.473045000	-2.013610000	-2.303435000
H	3.268514000	-1.655779000	1.730370000
C	-3.189806000	2.330743000	0.588309000
C	-3.939295000	1.517064000	1.438087000
H	4.746931000	1.958932000	0.155521000
H	-4.647988000	1.956504000	2.131230000

Table S37. XYZ of P2 H chloroform c 1.

O	-1.417165000	-2.820213000	0.699995000
N	0.509017000	-1.997128000	-0.085153000
H	0.494279000	-2.808280000	-0.692480000
N	2.423603000	2.274457000	0.402667000
C	-1.849363000	0.628314000	0.250672000
C	3.851895000	-0.774939000	-1.124901000
H	-2.424680000	0.530539000	-3.099958000
C	-0.550870000	-1.960955000	0.780308000
C	1.610776000	-1.135607000	-0.240968000
C	2.745307000	-1.609795000	-0.886818000
C	-0.738831000	0.560831000	1.290335000
H	4.716144000	1.193028000	-0.912251000
C	1.586796000	0.210894000	0.169415000
C	0.600470000	1.030585000	0.813568000
H	2.773016000	-2.643260000	-1.216173000
C	2.717463000	1.023958000	-0.079911000
C	3.864007000	0.549667000	-0.726966000
H	-0.568344000	0.530585000	-1.477497000
C	-1.592438000	0.570171000	-1.119830000
H	-3.388905000	0.740501000	1.746738000
C	-2.641412000	0.574481000	-2.038095000
H	0.748586000	3.167761000	1.386594000
C	1.157123000	2.272453000	0.941476000
H	4.715548000	-1.188433000	-1.633136000
C	-3.177244000	0.692901000	0.681986000
H	-5.249928000	0.753834000	0.121873000
C	-0.616327000	-0.881838000	1.842089000
H	0.270514000	-0.948489000	2.479386000
H	-1.492287000	-1.122268000	2.443722000
H	-1.041909000	1.188649000	2.133496000
C	-4.226071000	0.699036000	-0.231674000
C	-3.959996000	0.638033000	-1.598288000
H	3.043669000	3.068874000	0.377910000
H	-4.774776000	0.643256000	-2.313769000

Table S38. XYZ of P2 H chloroform c 2.

O	-0.550502000	-3.548789000	1.121147000
N	1.310449000	-2.367452000	0.737959000
H	1.780634000	-3.137829000	1.198918000
N	1.840325000	2.158114000	-0.431917000
C	-2.148652000	0.610948000	0.051100000
C	4.378211000	-0.523284000	-0.233456000
H	-4.899603000	2.140870000	1.342940000
C	-0.037776000	-2.560907000	0.613386000
C	2.137673000	-1.316514000	0.297752000
C	3.496766000	-1.553695000	0.139751000
C	-0.915422000	-0.154281000	0.488433000
H	4.613853000	1.561695000	-0.746111000
C	1.652044000	-0.015253000	0.072320000
C	0.350647000	0.585744000	0.158951000
H	3.882904000	-2.552370000	0.315915000
C	2.563853000	0.999997000	-0.302801000
C	3.934027000	0.766415000	-0.463456000
H	-2.888451000	0.886518000	2.047674000
C	-3.063658000	1.077744000	0.993283000
H	-1.679705000	0.514884000	-2.048568000
C	-4.198350000	1.784882000	0.596114000
H	-0.219328000	2.693958000	-0.230762000
C	0.515039000	1.905448000	-0.160244000
H	5.431841000	-0.753726000	-0.342628000
C	-2.388161000	0.864504000	-1.303190000
H	-3.693473000	1.753394000	-2.757830000
C	-0.870524000	-1.560497000	-0.159581000
H	-1.873122000	-1.984622000	-0.198711000
H	-0.489936000	-1.480983000	-1.182579000
H	-0.985395000	-0.294363000	1.573425000
C	-3.519640000	1.566627000	-1.703710000
C	-4.429461000	2.030248000	-0.753221000
H	2.215835000	3.053400000	-0.703049000
H	-5.311859000	2.577882000	-1.065210000

Table S39. XYZ of P2 H chloroform c 3.

O	-0.523774000	-0.278618000	3.989364000
N	-0.222084000	-2.495450000	4.002125000
H	-1.036315000	-2.505255000	4.605386000
N	2.341068000	-5.230718000	1.157590000
C	0.123760000	-1.245641000	0.697272000
C	0.517406000	-6.121006000	4.248931000
H	-1.141713000	1.439158000	-0.971209000
C	0.157298000	-1.231621000	3.638286000
C	0.339528000	-3.752430000	3.708970000
C	0.038217000	-4.829375000	4.532288000
C	1.399618000	-1.656816000	1.418834000

H	1.676291000	-7.373063000	2.924443000
C	1.142041000	-3.979028000	2.574650000
C	1.620476000	-3.134898000	1.517314000
H	-0.585182000	-4.667817000	5.405642000
C	1.611183000	-5.289004000	2.318097000
C	1.310088000	-6.377118000	3.144590000
H	0.932319000	0.661683000	0.122487000
C	0.057412000	0.017517000	0.103022000
H	-0.973209000	-3.053528000	1.104761000
C	-1.110617000	0.457332000	-0.511423000
H	2.887724000	-3.686869000	-0.216873000
C	2.348408000	-3.938321000	0.684571000
H	0.254881000	-6.932222000	4.918374000
C	-1.006874000	-2.063351000	0.661191000
H	-3.050294000	-2.271089000	0.032143000
C	1.428356000	-1.033256000	2.837225000
H	2.274954000	-1.445747000	3.394066000
H	1.557133000	0.046047000	2.762564000
H	2.240780000	-1.220395000	0.872368000
C	-2.180474000	-1.623486000	0.051106000
C	-2.236658000	-0.363142000	-0.536397000
H	2.812187000	-6.009838000	0.724847000
H	-3.148969000	-0.023552000	-1.014022000

Table S40. XYZ of P2 H chloroform c 4.

O	-0.905684000	-0.731495000	4.569577000
N	-0.501904000	-2.833374000	3.931076000
H	-1.312805000	-3.038419000	4.504028000
N	2.795811000	-4.706524000	1.150138000
C	0.008365000	-1.120640000	0.908835000
C	0.643422000	-6.352101000	3.667411000
H	-1.374598000	-2.068345000	-2.056814000
C	-0.182270000	-1.501373000	3.952818000
C	0.226772000	-3.958822000	3.501666000
C	-0.047009000	-5.194967000	4.071945000
C	1.245144000	-1.395147000	1.770611000
H	2.166078000	-7.203246000	2.394327000
C	1.205718000	-3.888606000	2.495659000
C	1.716405000	-2.815302000	1.695988000
H	-0.803944000	-5.265868000	4.846118000
C	1.897911000	-5.064280000	2.124109000
C	1.629405000	-6.312171000	2.698184000
H	0.554957000	-2.543829000	-0.608348000
C	-0.166719000	-1.793515000	-0.304681000
H	-0.850960000	0.380273000	2.206977000
C	-1.258979000	-1.526359000	-1.124363000
H	3.322649000	-2.879611000	0.167954000
C	2.689557000	-3.355180000	0.902825000
H	0.396987000	-7.295921000	4.140402000

C	-0.945490000	-0.165799000	1.275819000
H	-2.763222000	0.852874000	0.761137000
C	1.061586000	-0.988384000	3.246428000
H	1.935743000	-1.326577000	3.811239000
H	1.016684000	0.095376000	3.345725000
H	2.050847000	-0.764667000	1.376835000
C	-2.038807000	0.106071000	0.454783000
C	-2.201619000	-0.572545000	-0.748320000
H	3.447405000	-5.330576000	0.700557000
H	-3.054346000	-0.363592000	-1.384680000

Table S41. XYZ of P3 H chloroform c 1.

O	-0.425421000	-3.336167000	0.158650000
N	4.197042000	1.798244000	-0.636391000
H	-4.897762000	1.133288000	-2.157387000
N	0.945706000	-1.537800000	-0.064693000
C	-2.019003000	0.433561000	0.364984000
C	0.923526000	1.914706000	1.157299000
H	-5.404419000	0.783757000	0.249096000
C	-0.140366000	-2.207760000	0.486070000
C	2.953541000	1.421689000	-0.135745000
C	2.164099000	2.295697000	0.618369000
C	-0.931727000	0.100818000	1.382018000
H	4.486551000	1.304927000	-1.470274000
C	2.469069000	0.111499000	-0.321015000
C	2.916053000	-1.070443000	-1.034646000
H	4.323320000	2.795817000	-0.742768000
C	1.245923000	-0.221155000	0.263302000
C	0.424997000	0.631410000	0.986136000
H	-3.574163000	0.334136000	1.845652000
C	-3.348318000	0.487577000	0.793929000
H	-0.719177000	0.614244000	-1.345131000
C	-4.380075000	0.738494000	-0.104447000
H	1.938290000	-3.056520000	-1.215894000
C	1.977482000	-2.037960000	-0.863266000
H	0.340398000	2.649542000	1.704144000
C	-1.744330000	0.636316000	-0.988595000
H	-2.545166000	1.039859000	-2.940021000
C	-0.844903000	-1.431424000	1.585836000
H	-0.267020000	-1.637710000	2.494999000
H	-1.833840000	-1.870145000	1.716669000
H	3.829031000	-1.184339000	-1.600476000
C	-2.776486000	0.883541000	-1.892035000
C	-4.096222000	0.935913000	-1.454324000
H	2.519341000	3.309050000	0.776981000
H	-1.231151000	0.533209000	2.340516000

Table S42. XYZ of P3 H chloroform c 2.

O	-0.336474000	-3.429619000	0.325925000
N	4.135605000	1.778644000	-0.785900000
H	-4.836156000	1.413579000	-2.138111000
N	1.001009000	-1.618084000	0.029649000
C	-2.036620000	0.436442000	0.380959000
C	0.856722000	1.915478000	0.994686000
H	-3.020460000	-0.133438000	-2.826851000
C	-0.086258000	-2.275976000	0.588622000
C	2.902875000	1.397965000	-0.260900000
C	2.083703000	2.298002000	0.427754000
C	-0.941957000	0.072866000	1.372570000
H	4.448094000	1.231375000	-1.576704000
C	2.461490000	0.063266000	-0.351559000
C	2.956160000	-1.152827000	-0.970070000
H	4.225244000	2.768392000	-0.972304000
C	1.248016000	-0.268337000	0.255209000
C	0.400085000	0.607215000	0.916488000
H	-1.240758000	-0.739484000	-1.249778000
C	-2.034426000	-0.073724000	-0.921866000
H	-3.074572000	1.709527000	1.764165000
C	-3.036577000	0.273034000	-1.821555000
H	2.053030000	-3.181645000	-1.002453000
C	2.053551000	-2.138688000	-0.727617000
H	0.249795000	2.667042000	1.491056000
C	-3.060438000	1.305676000	0.756301000
H	-4.851761000	2.337371000	0.166126000
C	-0.850571000	-1.452594000	1.611348000
H	-0.316977000	-1.625835000	2.553870000
H	-1.841208000	-1.895260000	1.712967000
H	3.875886000	-1.275438000	-1.523025000
C	-4.064163000	1.659531000	-0.144526000
C	-4.056028000	1.142488000	-1.435605000
H	2.405552000	3.331170000	0.513156000
H	-1.203492000	0.528366000	2.330884000

Table S43. XYZ of P3 H chloroform c 3.

O	0.295524000	-3.748155000	0.364133000
N	4.208449000	1.967190000	-0.225971000
H	-5.493241000	2.264185000	-1.027646000
N	1.423807000	-1.779870000	0.253790000
C	-2.273799000	0.422417000	0.138292000
C	0.496451000	1.763109000	0.139305000
H	-5.224609000	1.606502000	1.349887000
C	0.264096000	-2.544476000	0.253525000
C	2.919749000	1.448345000	-0.118480000

C	1.792932000	2.272181000	-0.053564000
C	-1.029176000	-0.313022000	0.587952000
H	4.870107000	1.366120000	-0.698907000
C	2.710296000	0.059775000	0.000352000
C	3.556198000	-1.119233000	0.017510000
H	4.249928000	2.908235000	-0.593597000
C	1.399489000	-0.393407000	0.154390000
C	0.264498000	0.400047000	0.251533000
H	-3.174750000	0.436290000	2.087491000
C	-3.288944000	0.720782000	1.045764000
H	-1.644218000	0.585721000	-1.913977000
C	-4.445199000	1.380740000	0.630363000
H	2.985005000	-3.255998000	0.220834000
C	2.752999000	-2.203853000	0.171613000
H	-0.335522000	2.457816000	0.199389000
C	-2.433053000	0.800415000	-1.198547000
H	-3.693422000	1.745030000	-2.657157000
C	-1.004232000	-1.744866000	0.006908000
H	-1.842583000	-2.331224000	0.383026000
H	-1.104888000	-1.694547000	-1.084737000
H	4.632241000	-1.150562000	-0.070973000
C	-3.584628000	1.457399000	-1.617135000
C	-4.595882000	1.749852000	-0.702082000
H	1.929160000	3.345271000	-0.144079000
H	-1.075416000	-0.407144000	1.680098000

Table S44. XYZ of P3 H chloroform c 4.

O	0.293395000	-3.748070000	0.361787000
N	4.197845000	1.959071000	-0.355399000
H	-5.495302000	2.262692000	-1.027594000
N	1.423470000	-1.781291000	0.244900000
C	-2.272692000	0.423863000	0.134192000
C	0.499266000	1.762414000	0.124816000
H	-5.220787000	1.609886000	1.350651000
C	0.262974000	-2.544512000	0.248890000
C	2.923280000	1.445810000	-0.125294000
C	1.796697000	2.270293000	-0.062875000
C	-1.027190000	-0.311263000	0.581833000
H	4.955147000	1.402009000	0.016943000
C	2.711700000	0.056697000	-0.015377000

C	3.554908000	-1.124584000	-0.009842000
H	4.308477000	2.932609000	-0.105652000
C	1.400756000	-0.394727000	0.145586000
C	0.266397000	0.399788000	0.240296000
H	-3.168948000	0.441402000	2.085616000
C	-3.285692000	0.723846000	1.043594000
H	-1.647949000	0.583119000	-1.919799000
C	-4.443040000	1.382822000	0.629709000
H	2.981176000	-3.260654000	0.191414000
C	2.751117000	-2.207731000	0.150194000
H	-0.331744000	2.458190000	0.185721000
C	-2.435153000	0.799226000	-1.202999000
H	-3.699361000	1.740577000	-2.660441000
C	-1.005168000	-1.744335000	0.003755000
H	-1.843542000	-2.328769000	0.382773000
H	-1.108012000	-1.695813000	-1.087772000
H	4.629068000	-1.159476000	-0.118333000
C	-3.587959000	1.455115000	-1.620090000
C	-4.597035000	1.749198000	-0.703165000
H	1.933928000	3.343413000	-0.151890000
H	-1.069974000	-0.402969000	1.674359000

Table S45. XYZ of P3 H chloroform c 5.

O	0.295524000	-3.748155000	0.364133000
N	4.208449000	1.967190000	-0.225971000
H	-5.493241000	2.264185000	-1.027646000
N	1.423807000	-1.779870000	0.253790000
C	-2.273799000	0.422417000	0.138292000
C	0.496451000	1.763109000	0.139305000
H	-5.224609000	1.606502000	1.349887000
C	0.264096000	-2.544476000	0.253525000
C	2.919749000	1.448345000	-0.118480000
C	1.792932000	2.272181000	-0.053564000
C	-1.029176000	-0.313022000	0.587952000
H	4.870107000	1.366120000	-0.698907000
C	2.710296000	0.059775000	0.000352000
C	3.556198000	-1.119233000	0.017510000
H	4.249928000	2.908235000	-0.593597000
C	1.399489000	-0.393407000	0.154390000
C	0.264498000	0.400047000	0.251533000
H	-3.174750000	0.436290000	2.087491000
C	-3.288944000	0.720782000	1.045764000
H	-1.644218000	0.585721000	-1.913977000
C	-4.445199000	1.380740000	0.630363000
H	2.985005000	-3.255998000	0.220834000
C	2.752999000	-2.203853000	0.171613000
H	-0.335522000	2.457816000	0.199389000
C	-2.433053000	0.800415000	-1.198547000
H	-3.693422000	1.745030000	-2.657157000

C	-1.004232000	-1.744866000	0.006908000
H	-1.842583000	-2.331224000	0.383026000
H	-1.104888000	-1.694547000	-1.084737000
H	4.632241000	-1.150562000	-0.070973000
C	-3.584628000	1.457399000	-1.617135000
C	-4.595882000	1.749852000	-0.702082000
H	1.929160000	3.345271000	-0.144079000
H	-1.075416000	-0.407144000	1.680098000

Table S46. XYZ of P3 H chloroform c 6.

O	-0.498219000	-3.326540000	0.946602000
N	4.157027000	1.343797000	-1.262142000
H	-4.829473000	1.349619000	-2.164387000
N	0.917653000	-1.674698000	0.294539000
C	-1.986434000	0.605686000	0.384891000
C	1.010508000	1.973361000	0.645270000
H	-3.184771000	-0.453809000	-2.618555000
C	-0.179472000	-2.160765000	0.992494000
C	2.983160000	1.123272000	-0.544600000
C	2.239844000	2.176657000	-0.003417000
C	-0.872808000	0.337790000	1.385339000
H	4.824395000	0.584773000	-1.229225000
C	2.458354000	-0.176199000	-0.403438000
C	2.858468000	-1.507189000	-0.821022000
H	4.606646000	2.227861000	-1.065439000
C	1.249344000	-0.324303000	0.280034000
C	0.476928000	0.702135000	0.801784000
H	-1.378843000	-0.918191000	-1.023787000
C	-2.095701000	-0.133648000	-0.797708000
H	-2.840203000	2.200585000	1.541948000
C	-3.113353000	0.130560000	-1.707784000
H	1.838244000	-3.451730000	-0.487141000
C	1.910750000	-2.380003000	-0.390123000
H	0.466499000	2.834069000	1.023230000
C	-2.914003000	1.618401000	0.628324000
H	-4.645126000	2.680524000	-0.076896000
C	-0.860156000	-1.129061000	1.875730000
H	-0.306511000	-1.175302000	2.821458000
H	-1.870453000	-1.485955000	2.074930000
H	3.742771000	-1.776930000	-1.379452000
C	-3.932965000	1.889152000	-0.283964000
C	-4.036598000	1.144179000	-1.453897000
H	2.624534000	3.187025000	-0.101025000
H	-1.064506000	0.962408000	2.261338000

Table S47. XYZ of P1 Me c 1.

O	-1.685266000	3.315570000	-0.271040000
N	0.052276000	1.872652000	-0.338370000
H	0.432025000	2.404220000	-1.109348000
N	4.002003000	-0.634429000	-0.312268000
C	-2.305566000	-0.571473000	0.351662000
C	0.807665000	-1.293304000	1.426783000
H	0.282858000	-1.988145000	2.074895000
C	-1.124308000	2.349462000	0.193070000
C	0.748365000	0.754549000	0.137243000
C	0.116881000	-0.152138000	0.979270000
C	-1.334087000	0.084272000	1.328671000
H	-1.527810000	-0.342181000	2.317976000
C	2.077195000	0.526568000	-0.261291000
C	3.013086000	1.230716000	-1.089203000
H	2.873568000	2.164896000	-1.610957000
C	2.729868000	-0.634330000	0.206697000
C	2.111986000	-1.559774000	1.055972000
H	2.628002000	-2.445193000	1.408416000
C	-1.933246000	-0.961698000	-0.933561000
H	-0.904648000	-0.841896000	-1.256794000
C	-2.868514000	-1.518532000	-1.803160000
H	-2.562150000	-1.815689000	-2.800015000
C	4.160981000	0.489577000	-1.089030000
H	5.102685000	0.671377000	-1.585324000
C	-3.632806000	-0.756159000	0.748102000
H	-3.935573000	-0.461166000	1.749493000
C	-1.596899000	1.598406000	1.420083000
H	-2.653584000	1.825202000	1.557945000
H	-1.045774000	2.011131000	2.272880000
C	4.993080000	-1.655214000	-0.057397000
H	5.212074000	-1.720089000	1.010761000
H	5.909122000	-1.400369000	-0.587520000
H	4.643197000	-2.628427000	-0.408699000
C	-4.185588000	-1.696967000	-1.398573000
H	-4.911066000	-2.133420000	-2.075742000
C	-4.566625000	-1.315100000	-0.114724000
H	-5.590428000	-1.454928000	0.214107000

Table S48. XYZ of P1 Me c 2.

O	-1.041783000	3.998282000	0.157359000
N	0.520368000	2.369062000	0.261713000
H	1.223364000	3.083684000	0.389420000
N	4.014334000	-0.710883000	-0.101946000
C	-2.491529000	-0.634919000	0.148049000
C	0.389013000	-1.333724000	0.290676000
H	-0.362167000	-2.109706000	0.386014000
C	-0.766390000	2.821107000	0.094002000

C	0.914100000	1.025415000	0.196500000
C	-0.030596000	0.010301000	0.306885000
C	-1.477009000	0.420096000	0.536001000
H	-1.597097000	0.620316000	1.609281000
C	2.274554000	0.704593000	0.056628000
C	3.476848000	1.473400000	-0.090950000
H	3.583607000	2.546294000	-0.136413000
C	2.649313000	-0.655742000	0.045564000
C	1.718356000	-1.692845000	0.164223000
H	2.018824000	-2.734163000	0.156416000
C	-2.510776000	-1.159679000	-1.147504000
H	-1.761706000	-0.837704000	-1.865201000
C	-3.462199000	-2.101338000	-1.518000000
H	-3.462600000	-2.499109000	-2.526789000
C	4.500771000	0.573568000	-0.182198000
H	5.560607000	0.741308000	-0.303881000
C	-3.443130000	-1.079675000	1.062510000
H	-3.437485000	-0.683986000	2.073614000
C	-1.769723000	1.730939000	-0.212028000
H	-1.728435000	1.550883000	-1.292781000
H	-2.763733000	2.112511000	0.019623000
C	4.785655000	-1.931548000	-0.165549000
H	4.675723000	-2.506165000	0.756812000
H	5.836567000	-1.680374000	-0.298484000
H	4.462491000	-2.546604000	-1.008435000
C	-4.412430000	-2.536583000	-0.595934000
H	-5.154967000	-3.271781000	-0.884786000
C	-4.399914000	-2.024487000	0.695445000
H	-5.133098000	-2.358484000	1.421107000

Table S49. XYZ of P1 Me c 3.

O	-1.499327000	3.400707000	-0.621594000
N	0.076111000	1.779848000	-0.584282000
H	0.488974000	2.193102000	-1.408821000
N	3.911997000	-0.866081000	-0.132175000
C	-2.354274000	-0.400692000	0.294047000
C	0.683203000	-1.102195000	1.654641000
H	0.142406000	-1.642266000	2.425546000
C	-1.020987000	2.431332000	-0.074862000
C	0.711358000	0.695409000	0.035285000
C	0.031365000	-0.044551000	0.995278000
C	-1.417022000	0.319893000	1.265121000
H	-1.672774000	-0.014231000	2.275787000
C	2.032001000	0.356637000	-0.299704000
C	3.002577000	0.883822000	-1.214660000
H	2.901327000	1.722423000	-1.886063000
C	2.636152000	-0.734816000	0.361750000
C	1.982291000	-1.471340000	1.355029000
H	2.468798000	-2.288976000	1.873981000

C	-3.200362000	0.272587000	-0.586781000
H	-3.224648000	1.355851000	-0.620674000
C	-4.036267000	-0.434389000	-1.451481000
H	-4.681515000	0.110961000	-2.131142000
C	4.120342000	0.110373000	-1.078344000
H	5.071140000	0.174109000	-1.586368000
C	-2.370222000	-1.799474000	0.288625000
H	-1.712029000	-2.342806000	0.959134000
C	-1.544971000	1.844580000	1.221962000
H	-2.568815000	2.191577000	1.357278000
H	-0.940891000	2.271846000	2.029573000
C	4.860302000	-1.869609000	0.294863000
H	4.469051000	-2.872257000	0.108969000
H	5.079713000	-1.764593000	1.359843000
H	5.784748000	-1.745297000	-0.266457000
C	-4.044487000	-1.821862000	-1.443861000
H	-4.696102000	-2.369686000	-2.115139000
C	-3.205944000	-2.504998000	-0.565104000
H	-3.202594000	-3.589316000	-0.548072000

Table S50. XYZ of P1 Me c 4.

O	-1.814979000	3.237143000	0.164452000
N	-0.037927000	1.868966000	-0.113080000
H	0.303464000	2.494843000	-0.829203000
N	3.986281000	-0.486457000	-0.483492000
C	-2.285712000	-0.707614000	0.354202000
C	0.878888000	-1.436354000	1.279247000
H	0.399930000	-2.214653000	1.865006000
C	-1.210507000	2.245497000	0.501885000
C	0.709844000	0.732597000	0.219481000
C	0.135978000	-0.280620000	0.976703000
C	-1.312738000	-0.136215000	1.382066000
H	-1.469327000	-0.680170000	2.318845000
C	2.028370000	0.593697000	-0.248467000
C	2.910116000	1.412365000	-1.028956000
H	2.720999000	2.389825000	-1.445136000
C	2.734645000	-0.584979000	0.074909000
C	2.177870000	-1.614947000	0.842345000
H	2.734816000	-2.512686000	1.083885000
C	-3.596650000	-0.982396000	0.753135000
H	-3.884038000	-0.817312000	1.788289000
C	-4.533612000	-1.466487000	-0.150408000
H	-5.544434000	-1.677762000	0.180363000
C	4.080712000	0.716782000	-1.143272000
H	4.996724000	0.983585000	-1.649138000
C	-1.933118000	-0.931010000	-0.975601000
H	-0.917469000	-0.738657000	-1.304481000
C	-1.621494000	1.347554000	1.650377000

H	-2.680578000	1.519862000	1.839306000
H	-1.059235000	1.682164000	2.529706000
C	5.019779000	-1.490181000	-0.366347000
H	4.689120000	-2.436189000	-0.800685000
H	5.281754000	-1.652046000	0.681646000
H	5.905659000	-1.150833000	-0.900326000
C	-4.172512000	-1.681455000	-1.478099000
H	-4.900654000	-2.057991000	-2.187566000
C	-2.871643000	-1.413079000	-1.885329000
H	-2.580660000	-1.579717000	-2.916580000

Table S51. XYZ of P1 Me c 5.

O	-1.500033000	3.394518000	-0.661979000
N	0.076879000	1.775490000	-0.610947000
H	0.481982000	2.174919000	-1.446067000
N	3.909304000	-0.874652000	-0.156293000
C	-2.350119000	-0.390934000	0.312673000
C	0.695241000	-1.078244000	1.661202000
H	0.159871000	-1.606380000	2.444038000
C	-1.017315000	2.433622000	-0.104171000
C	0.714333000	0.697409000	0.017718000
C	0.040337000	-0.028010000	0.992873000
C	-1.405730000	0.341869000	1.267883000
H	-1.654909000	0.021781000	2.284722000
C	2.031003000	0.350474000	-0.324439000
C	2.995760000	0.864071000	-1.253203000
H	2.891010000	1.694294000	-1.934436000
C	2.638025000	-0.733685000	0.346502000
C	1.990853000	-1.454597000	1.355407000
H	2.479835000	-2.266292000	1.881379000
C	-3.201687000	0.271003000	-0.571496000
H	-3.226434000	1.353753000	-0.619236000
C	-4.042721000	-0.447197000	-1.421955000
H	-4.691877000	0.089496000	-2.104804000
C	4.112835000	0.089795000	-1.115850000
H	5.059898000	0.144940000	-1.631799000
C	-2.366398000	-1.789735000	0.325524000
H	-1.704458000	-2.324490000	0.999222000
C	-1.532484000	1.865959000	1.204587000
H	-2.555101000	2.215478000	1.342522000
H	-0.922183000	2.303550000	2.001910000
C	4.861170000	-1.870588000	0.280713000
H	5.089261000	-1.746704000	1.341857000
H	5.780983000	-1.756484000	-0.290321000
H	4.468607000	-2.876372000	0.115876000
C	-4.050864000	-1.834460000	-1.396603000
H	-4.706207000	-2.390890000	-2.057087000
C	-3.207284000	-2.506251000	-0.513832000
H	-3.203863000	-3.590259000	-0.482779000

Table S52. XYZ of P1 Me c 6.

O	-1.734495000	3.297616000	-0.081303000
N	0.015233000	1.874986000	-0.224887000
H	0.390428000	2.447943000	-0.967905000
N	3.987875000	-0.591534000	-0.340651000
C	-2.309854000	-0.634763000	0.343253000
C	0.809212000	-1.366295000	1.379311000
H	0.294076000	-2.098193000	1.993544000
C	-1.165764000	2.312494000	0.330122000
C	0.724178000	0.741787000	0.193617000
C	0.105025000	-0.211516000	0.991588000
C	-1.349002000	-0.010876000	1.351138000
H	-1.536614000	-0.486931000	2.318783000
C	2.051859000	0.545740000	-0.225533000
C	2.975890000	1.297591000	-1.024508000
H	2.823792000	2.254330000	-1.499806000
C	2.718632000	-0.629099000	0.183942000
C	2.114285000	-1.600974000	0.990130000
H	2.640481000	-2.497533000	1.296377000
C	-1.931743000	-0.958847000	-0.958594000
H	-0.905718000	-0.806168000	-1.276110000
C	-2.857507000	-1.492397000	-1.852675000
H	-2.546497000	-1.738468000	-2.861959000
C	4.131128000	0.569011000	-1.064947000
H	5.067825000	0.784523000	-1.557186000
C	-3.633621000	-0.860548000	0.730432000
H	-3.941202000	-0.616981000	1.744100000
C	-1.633221000	1.493084000	1.515004000
H	-2.693683000	1.698182000	1.658080000
H	-1.092856000	1.871007000	2.390512000
C	4.989395000	-1.615100000	-0.144014000
H	5.899713000	-1.324559000	-0.665530000
H	4.646708000	-2.572952000	-0.541504000
H	5.215880000	-1.731135000	0.918257000
C	-4.170862000	-1.712859000	-1.456963000
H	-4.888666000	-2.131583000	-2.153229000
C	-4.557892000	-1.396182000	-0.157065000
H	-5.578741000	-1.569201000	0.165081000

Table S53. XYZ of P2 Me c 1.

O	-2.006684000	-2.710565000	0.839960000
N	-0.002913000	-2.199176000	-0.015485000
H	-0.158301000	-3.022241000	-0.583223000
N	2.548535000	1.745641000	0.283608000
C	-1.926932000	0.762189000	0.235275000
C	3.466364000	-1.535953000	-1.137822000
H	-4.829234000	1.126520000	-2.323596000
C	-1.039700000	-1.980056000	0.868813000

C	1.210510000	-1.525136000	-0.216368000
C	2.253704000	-2.184765000	-0.855628000
C	-0.830229000	0.563735000	1.273120000
H	4.249296000	-2.092414000	-1.639936000
C	1.398269000	-0.178791000	0.144541000
C	0.559355000	0.802882000	0.768917000
H	4.621845000	0.281242000	-1.012323000
C	2.632709000	0.445634000	-0.147300000
C	3.684471000	-0.215155000	-0.790193000
H	-3.435924000	1.089187000	1.731087000
C	-1.682995000	0.654242000	-1.133018000
H	-5.279710000	1.325880000	0.109101000
C	-2.723857000	0.783599000	-2.049262000
H	-0.673561000	0.474522000	-1.488107000
C	1.300601000	1.947870000	0.833874000
H	2.125885000	-3.223517000	-1.141792000
C	-3.232467000	1.004512000	0.666947000
H	-2.517904000	0.696959000	-3.110413000
C	-0.924252000	-0.856643000	1.882659000
H	-0.051548000	-1.032363000	2.519327000
H	-1.821802000	-0.942896000	2.494327000
C	3.609907000	2.720348000	0.173670000
H	3.880554000	2.879273000	-0.872629000
H	4.494828000	2.392649000	0.724064000
H	3.267447000	3.665070000	0.592366000
C	-4.020016000	1.025250000	-1.609284000
H	1.038307000	2.914509000	1.239955000
C	-4.272402000	1.136558000	-0.244798000
H	-1.021379000	1.266396000	2.090782000

Table S54. XYZ of P2 Me c 2.

O	-1.304681000	-3.612171000	0.991342000
N	0.730765000	-2.725556000	0.712614000
H	1.044437000	-3.580015000	1.154581000
N	2.031111000	1.676932000	-0.324694000
C	-2.173369000	0.801047000	0.063494000
C	4.088391000	-1.381363000	-0.099931000
H	-4.952266000	3.291918000	-1.024032000
C	-0.637083000	-2.701784000	0.545292000
C	1.731255000	-1.816093000	0.334884000
C	3.042316000	-2.261761000	0.218340000
C	-1.092391000	-0.173231000	0.487247000
H	5.095142000	-1.775356000	-0.176692000
C	1.470364000	-0.451660000	0.121617000
C	0.284839000	0.352330000	0.197327000
H	4.675767000	0.642545000	-0.561017000
C	2.543364000	0.409674000	-0.202796000
C	3.864353000	-0.034180000	-0.319833000
H	-1.646558000	0.745993000	-2.021311000

C	-3.039074000	1.354706000	1.003960000
H	-3.422201000	2.315185000	-2.715152000
C	-4.035100000	2.249416000	0.617617000
H	-2.935303000	1.082172000	2.049709000
C	0.674188000	1.631709000	-0.084116000
H	3.260021000	-3.312758000	0.377431000
C	-2.322059000	1.160628000	-1.279015000
H	-4.699731000	2.670452000	1.363777000
C	-1.275795000	-1.547389000	-0.201982000
H	-2.333958000	-1.798222000	-0.263578000
H	-0.871608000	-1.501264000	-1.218203000
C	2.811057000	2.855317000	-0.627633000
H	3.305373000	2.753725000	-1.596350000
H	3.568256000	3.025024000	0.141534000
H	2.148372000	3.718262000	-0.661845000
C	-4.176659000	2.598145000	-0.719987000
H	0.078524000	2.531012000	-0.147652000
C	-3.316496000	2.049105000	-1.669203000
H	-1.199997000	-0.332293000	1.566866000

Table S55. XYZ of P2 Me c 3.

O	-1.333275000	-3.593065000	1.020918000
N	0.708246000	-2.705373000	0.794995000
H	1.008903000	-3.550046000	1.264014000
N	2.035904000	1.675820000	-0.296020000
C	-2.177621000	0.800576000	-0.017674000
C	4.089027000	-1.372158000	0.063988000
H	-4.924533000	3.268446000	-1.230940000
C	-0.653144000	-2.689495000	0.580099000
C	1.719584000	-1.802522000	0.430488000
C	3.034261000	-2.247937000	0.365938000
C	-1.108673000	-0.163775000	0.455848000
H	5.098345000	-1.765525000	0.028921000
C	1.464110000	-0.443575000	0.179199000
C	0.275577000	0.358990000	0.197037000
H	4.688744000	0.642547000	-0.420349000
C	2.545802000	0.412890000	-0.127995000
C	3.870602000	-0.030458000	-0.191930000
H	-2.982395000	1.135283000	1.943190000
C	-2.295110000	1.125752000	-1.371806000
H	-4.726569000	2.709386000	1.177015000
C	-3.278037000	2.005979000	-1.807060000
H	-1.604599000	0.690166000	-2.087841000
C	0.671720000	1.632606000	-0.100283000
H	3.248278000	-3.294828000	0.554287000
C	-3.062262000	1.380722000	0.888688000
H	-3.359743000	2.244951000	-2.861655000
C	-1.268708000	-1.551699000	-0.210961000
H	-2.324047000	-1.806014000	-0.301026000

H	-0.832434000	-1.525807000	-1.214514000
C	2.823525000	2.848832000	-0.599415000
H	2.159994000	3.707766000	-0.683881000
H	3.356283000	2.722992000	-1.544617000
H	3.548857000	3.042923000	0.194329000
C	-4.157561000	2.581378000	-0.891810000
H	0.076747000	2.528701000	-0.203409000
C	-4.046650000	2.267456000	0.457174000
H	-1.247520000	-0.301895000	1.534814000

Table S56. XYZ of P2 Me c 4.

O	-1.332701000	-3.595083000	1.010474000
N	0.708756000	-2.711564000	0.768311000
H	1.011966000	-3.559659000	1.229442000
N	2.038090000	1.670608000	-0.316584000
C	-2.175760000	0.806295000	0.005646000
C	4.085375000	-1.385369000	0.006044000
H	-4.932647000	3.284382000	-1.163522000
C	-0.654754000	-2.690849000	0.567650000
C	1.718792000	-1.810058000	0.397034000
C	3.031334000	-2.259184000	0.316099000
C	-1.103695000	-0.162915000	0.461934000
H	5.093014000	-1.781657000	-0.042005000
C	1.464786000	-0.449214000	0.154760000
C	0.279129000	0.357090000	0.190180000
H	4.685798000	0.629636000	-0.476548000
C	2.545652000	0.405179000	-0.160877000
C	3.868274000	-0.041909000	-0.241680000
H	-2.966170000	1.121773000	1.975501000
C	-2.303671000	1.144093000	-1.344495000
H	-4.716666000	2.702203000	1.237478000
C	-3.290174000	2.028042000	-1.763910000
H	-1.618424000	0.715615000	-2.069809000
C	0.676077000	1.630864000	-0.105571000
H	3.244159000	-3.307557000	0.497412000
C	-3.053948000	1.377265000	0.924015000
H	-3.379858000	2.277019000	-2.815542000
C	-1.275426000	-1.546865000	-0.210386000
H	-2.332488000	-1.797432000	-0.290122000
H	-0.850288000	-1.516811000	-1.218597000
C	2.826489000	2.841929000	-0.624551000
H	3.341449000	2.721804000	-1.580249000
H	3.567156000	3.024547000	0.157755000
H	2.166528000	3.705263000	-0.688793000
C	-4.163020000	2.594323000	-0.836705000
H	0.082994000	2.529421000	-0.197475000
C	-4.041970000	2.267524000	0.508370000
H	-1.230670000	-0.306372000	1.541662000

Table S57. XYZ of P1 H c 1.

O	-1.069376000	-3.345367000	0.277730000
N	0.518853000	-1.738830000	0.336399000
H	0.954912000	-2.229680000	1.104492000
N	4.186323000	1.147487000	0.320526000
C	-2.096188000	0.458523000	-0.348153000
C	0.927565000	1.506467000	-1.397106000
H	-2.525775000	1.534397000	2.846384000
C	-0.609473000	-2.328250000	-0.187690000
C	1.089792000	-0.549444000	-0.133931000
C	0.361258000	0.293955000	-0.960738000
C	-1.055994000	-0.092483000	-1.317594000
H	2.617925000	2.840584000	-1.375895000
C	2.392383000	-0.189662000	0.257282000
C	3.399470000	-0.810355000	1.070836000
H	-1.286677000	0.311407000	-2.308259000
C	2.917673000	1.036901000	-0.199180000
C	2.198948000	1.902280000	-1.031963000
H	-0.762896000	0.745558000	1.314704000
C	-1.787675000	0.811560000	0.964779000
H	-3.673895000	0.305460000	-1.800494000
C	-2.782584000	1.264613000	1.827986000
H	5.425239000	-0.061800000	1.572386000
C	4.469774000	0.036391000	1.081294000
H	0.329771000	2.150717000	-2.034131000
C	-3.420690000	0.573563000	-0.778035000
H	-5.435310000	1.117651000	-0.275839000
C	-1.160973000	-1.625843000	-1.410847000
H	-2.189518000	-1.958690000	-1.546256000
H	-0.572853000	-1.977714000	-2.266266000
H	3.352872000	-1.762055000	1.577346000
C	-4.414369000	1.029655000	0.078830000
C	-4.096785000	1.375238000	1.389786000
H	4.811888000	1.919320000	0.164274000
H	-4.868928000	1.731316000	2.062323000

Table S58. XYZ of P1 H c 2.

O	-0.344523000	-3.945767000	-0.177322000
N	1.023572000	-2.151229000	-0.296169000
H	1.800324000	-2.783293000	-0.431436000
N	4.167253000	1.263867000	0.147654000
C	-2.301859000	0.498265000	-0.140285000
C	0.489354000	1.513200000	-0.212253000
H	-3.510342000	2.220913000	2.532691000
C	-0.202771000	-2.745620000	-0.111730000
C	1.269723000	-0.774796000	-0.197238000
C	0.219245000	0.132095000	-0.273151000
C	-1.170561000	-0.432099000	-0.521260000

H	1.956929000	3.077496000	-0.033333000
C	2.588571000	-0.311237000	-0.051827000
C	3.867862000	-0.954299000	0.066159000
H	-1.253458000	-0.634759000	-1.597696000
C	2.811602000	1.079918000	0.003433000
C	1.771155000	2.010655000	-0.076271000
H	-1.627758000	0.762476000	1.885452000
C	-2.397457000	1.006009000	1.158661000
H	-3.221686000	0.457939000	-2.078929000
C	-3.452216000	1.833555000	1.521526000
H	5.866532000	-0.023092000	0.295433000
C	4.795283000	0.039734000	0.184035000
H	-0.341922000	2.205703000	-0.281847000
C	-3.285110000	0.841819000	-1.065216000
H	-5.100865000	1.929118000	-1.439815000
C	-1.318983000	-1.775937000	0.210153000
H	-1.296561000	-1.607026000	1.293319000
H	-2.265302000	-2.261338000	-0.027126000
H	4.084758000	-2.011439000	0.074797000
C	-4.345311000	1.671930000	-0.705763000
C	-4.431759000	2.169238000	0.588483000
H	4.627706000	2.155266000	0.216488000
H	-5.254550000	2.816279000	0.870826000

Table S59. XYZ of P1 H c 3.

O	-1.009155000	-3.413681000	0.189783000
N	0.463516000	-1.707907000	0.364653000
H	0.888034000	-2.183112000	1.148871000
N	4.110898000	1.207144000	0.324627000
C	-2.117577000	0.401621000	-0.319323000
C	0.889022000	1.469559000	-1.480354000
H	-3.256199000	3.409565000	0.799647000
C	-0.585144000	-2.361461000	-0.234854000
C	1.025824000	-0.513630000	-0.106018000
C	0.305717000	0.291723000	-0.977811000
C	-1.106699000	-0.138428000	-1.332237000
H	2.587609000	2.793946000	-1.508835000
C	2.319004000	-0.135200000	0.291781000
C	3.313635000	-0.717028000	1.148338000
H	-1.359945000	0.290708000	-2.306986000
C	2.851443000	1.070336000	-0.211719000
C	2.154115000	1.884291000	-1.110200000
H	-1.644040000	2.447011000	-0.787408000
C	-2.257604000	1.786726000	-0.182793000
H	-2.843421000	-1.503304000	0.405075000
C	-3.163166000	2.332422000	0.715653000
H	5.327944000	0.058684000	1.652706000
C	4.381916000	0.131962000	1.139440000
H	0.321909000	2.066372000	-2.187753000

C	-2.912488000	-0.423726000	0.475594000
H	-4.422021000	-0.539342000	1.996550000
C	-1.128279000	-1.663229000	-1.466595000
H	-2.123822000	-2.059078000	-1.664430000
H	-0.483642000	-1.952190000	-2.303666000
H	3.258474000	-1.643068000	1.699498000
C	-3.818239000	0.122296000	1.385478000
C	-3.948859000	1.498182000	1.508711000
H	4.737831000	1.972848000	0.145733000
H	-4.655184000	1.920616000	2.214385000

Table S60. XYZ of P1 H c 4.

O	-1.266115000	-3.268318000	-0.363317000
N	0.389961000	-1.783988000	0.037431000
H	0.766932000	-2.425083000	0.721475000
N	4.194977000	0.845336000	0.664537000
C	-2.057781000	0.646612000	-0.343529000
C	1.057078000	1.668677000	-1.117721000
H	-5.365477000	1.445359000	-0.239473000
C	-0.738608000	-2.211943000	-0.624994000
C	1.045919000	-0.570467000	-0.204126000
C	0.405650000	0.438640000	-0.909764000
C	-1.015784000	0.206162000	-1.367146000
H	2.812335000	2.877198000	-0.806935000
C	2.340838000	-0.355030000	0.301432000
C	3.267202000	-1.156066000	1.051299000
H	-1.187728000	0.787178000	-2.278563000
C	2.953199000	0.895091000	0.075283000
C	2.326990000	1.923051000	-0.638663000
H	-3.600071000	0.793762000	-1.834213000
C	-3.363546000	0.889518000	-0.777666000
H	-0.760223000	0.612876000	1.371377000
C	-4.359669000	1.255704000	0.118572000
H	5.297492000	-0.619308000	1.761657000
C	4.377276000	-0.387189000	1.248719000
H	0.527688000	2.442477000	-1.664732000
C	-1.770659000	0.780389000	1.013976000
H	-2.527589000	1.242950000	2.968555000
C	-1.201294000	-1.279573000	-1.725167000
H	-2.240217000	-1.521965000	-1.946441000
H	-0.600160000	-1.516654000	-2.610515000
H	3.143908000	-2.171774000	1.394198000
C	-2.768221000	1.143761000	1.915856000
C	-4.063602000	1.382114000	1.473297000
H	4.866686000	1.593824000	0.666202000
H	-4.837400000	1.667925000	2.176757000

Table S61. XYZ of P1 H c 5.

O	-0.995180000	-3.439867000	0.194403000
N	0.476258000	-1.733025000	0.368345000
H	0.893108000	-2.201564000	1.160647000
N	4.123600000	1.181775000	0.342780000
C	-2.096518000	0.373963000	-0.361647000
C	0.921477000	1.428539000	-1.499197000
H	-4.413554000	-0.539198000	1.952779000
C	-0.566998000	-2.391320000	-0.235222000
C	1.043496000	-0.542888000	-0.106941000
C	0.332827000	0.254838000	-0.993294000
C	-1.076171000	-0.177699000	-1.358534000
H	2.620155000	2.752867000	-1.520322000
C	2.332202000	-0.161036000	0.301879000
C	3.317264000	-0.735187000	1.174507000
H	-1.318626000	0.241974000	-2.340103000
C	2.870084000	1.040224000	-0.206079000
C	2.182427000	1.846527000	-1.118774000
H	-2.822269000	-1.522175000	0.385583000
C	-2.894446000	-0.441862000	0.440002000
H	-1.626220000	2.413417000	-0.857657000
C	-3.807576000	0.114997000	1.335903000
H	5.325937000	0.045029000	1.694124000
C	4.385571000	0.113814000	1.169950000
H	0.362148000	2.019375000	-2.217709000
C	-2.241636000	1.760498000	-0.246943000
H	-3.251407000	3.394866000	0.704442000
C	-1.098568000	-1.703748000	-1.477948000
H	-2.093060000	-2.100023000	-1.680273000
H	-0.447338000	-2.001892000	-2.306669000
H	3.256192000	-1.656448000	1.733005000
C	-3.154609000	2.316878000	0.637334000
C	-3.942924000	1.492142000	1.437741000
H	4.752340000	1.946107000	0.164350000
H	-4.655033000	1.922942000	2.132453000

Table S62. XYZ of P2 H c 1.

O	-1.388205000	-2.851762000	0.751617000
N	0.508475000	-1.997258000	-0.073331000
H	0.485584000	-2.814899000	-0.669064000
N	2.415333000	2.272532000	0.398702000
C	-1.845432000	0.625457000	0.252249000
C	3.836160000	-0.770332000	-1.145210000
H	-2.394963000	0.560558000	-3.100135000
C	-0.551828000	-1.976192000	0.810056000
C	1.603765000	-1.137865000	-0.241497000
C	2.735103000	-1.606033000	-0.897787000
C	-0.741018000	0.555430000	1.298041000

H	4.701845000	1.194359000	-0.939460000
C	1.585109000	0.207658000	0.172252000
C	0.599466000	1.024488000	0.823386000
H	2.767119000	-2.640229000	-1.224242000
C	2.710525000	1.021520000	-0.086301000
C	3.851034000	0.552158000	-0.744787000
H	-0.550726000	0.547455000	-1.464101000
C	-1.577729000	0.581660000	-1.115416000
H	-3.396653000	0.716789000	1.737298000
C	-2.619264000	0.594188000	-2.039697000
H	0.736285000	3.161152000	1.387269000
C	1.149824000	2.266383000	0.946647000
H	4.695694000	-1.181606000	-1.661625000
C	-3.175157000	0.683054000	0.673922000
H	-5.242687000	0.744132000	0.100478000
C	-0.621598000	-0.882336000	1.859369000
H	0.261011000	-0.943472000	2.503803000
H	-1.501293000	-1.125471000	2.454516000
H	-1.044135000	1.191265000	2.136233000
C	-4.216299000	0.697463000	-0.246051000
C	-3.939784000	0.652123000	-1.609540000
H	3.031284000	3.066809000	0.367511000
H	-4.749182000	0.662424000	-2.330744000

Table S63. XYZ of P2 H c 2.

O	-0.552980000	-3.524768000	1.162972000
N	1.311473000	-2.359423000	0.744848000
H	1.769284000	-3.126888000	1.219477000
N	1.846590000	2.152524000	-0.451266000
C	-2.144455000	0.621644000	0.054152000
C	4.379766000	-0.530721000	-0.241943000
H	-4.948038000	2.060719000	1.332692000
C	-0.048221000	-2.553550000	0.638120000
C	2.139714000	-1.317399000	0.298479000
C	3.498049000	-1.555748000	0.136779000
C	-0.911389000	-0.143944000	0.490619000
H	4.622319000	1.547165000	-0.765872000
C	1.657621000	-0.016682000	0.063625000
C	0.356605000	0.587039000	0.150682000
H	3.884240000	-2.554667000	0.310401000
C	2.569854000	0.992452000	-0.317944000
C	3.938293000	0.757208000	-0.478803000
H	-2.929495000	0.824070000	2.040799000
C	-3.086400000	1.044029000	0.989334000
H	-1.631494000	0.597427000	-2.034748000
C	-4.225253000	1.740787000	0.590464000
H	-0.218874000	2.687110000	-0.245358000
C	0.519552000	1.902925000	-0.173967000
H	5.432512000	-0.763481000	-0.352216000

C	-2.363028000	0.910726000	-1.295863000
H	-3.655643000	1.816686000	-2.749671000
C	-0.878473000	-1.555696000	-0.144002000
H	-1.882300000	-1.977606000	-0.171029000
H	-0.503682000	-1.485792000	-1.169924000
H	-0.974858000	-0.275602000	1.577291000
C	-3.498334000	1.603088000	-1.698225000
C	-4.434367000	2.021198000	-0.754059000
H	2.223483000	3.043312000	-0.726178000
H	-5.320984000	2.560564000	-1.067623000

Table S64. XYZ of P2 H c 3.

O	-1.272006000	-2.946449000	0.204995000
N	0.598146000	-1.887824000	-0.420213000
H	0.627293000	-2.597484000	-1.140897000
N	2.279287000	2.334275000	0.775520000
C	-1.889248000	0.526455000	0.253041000
C	3.887438000	-0.360337000	-1.177844000
H	-2.367337000	0.978495000	-3.080758000
C	-0.478124000	-2.057671000	0.426864000
C	1.654867000	-0.965742000	-0.414641000
C	2.824220000	-1.274066000	-1.097952000
C	-0.803139000	0.349570000	1.305115000
H	4.647497000	1.585972000	-0.644041000
C	1.559696000	0.294417000	0.205196000
C	0.520350000	0.951386000	0.946892000
H	2.915549000	-2.241547000	-1.580367000
C	2.648411000	1.190101000	0.110907000
C	3.825779000	0.882720000	-0.577486000
H	-0.559025000	0.815228000	-1.412350000
C	-1.592701000	0.726496000	-1.094487000
H	-3.472068000	0.271933000	1.685332000
C	-2.614063000	0.823174000	-2.036313000
H	0.535713000	2.976530000	1.840991000
C	1.003996000	2.183060000	1.278140000
H	4.778977000	-0.645497000	-1.724203000
C	-3.227639000	0.428445000	0.638098000
H	-5.282551000	0.450086000	0.019464000
C	-0.618103000	-1.151740000	1.636331000
H	0.257470000	-1.271104000	2.282146000
H	-1.492205000	-1.529555000	2.165746000
H	-1.155959000	0.829811000	2.223644000
C	-4.248880000	0.527328000	-0.298806000
C	-3.943600000	0.724024000	-1.642522000
H	2.855339000	3.150820000	0.888267000
H	-4.737618000	0.799938000	-2.376722000

Table S65. XYZ of P3 H c 1.

O	-0.360279000	-3.368476000	0.081096000
N	4.178795000	1.854563000	-0.599875000
H	-4.918010000	1.194353000	-2.117137000
N	0.994200000	-1.549090000	-0.084713000
C	-2.030773000	0.412946000	0.365713000
C	0.884821000	1.893658000	1.154544000
H	-5.352833000	1.149756000	0.325766000
C	-0.102240000	-2.246018000	0.426817000
C	2.938024000	1.448921000	-0.112029000
C	2.123460000	2.301932000	0.634541000
C	-0.935649000	0.047949000	1.359440000
H	4.485610000	1.374658000	-1.433422000
C	2.486265000	0.130745000	-0.311353000
C	2.968931000	-1.039805000	-1.017808000
H	4.293243000	2.853765000	-0.687003000
C	1.260607000	-0.229643000	0.252388000
C	0.414341000	0.603348000	0.969117000
H	-3.517778000	0.646859000	1.898556000
C	-3.321601000	0.667477000	0.830175000
H	-0.793570000	0.275776000	-1.392556000
C	-4.357605000	0.948553000	-0.054813000
H	2.027126000	-3.044885000	-1.215992000
C	2.046109000	-2.025816000	-0.864283000
H	0.279506000	2.613957000	1.696142000
C	-1.794743000	0.448606000	-1.009968000
H	-2.630444000	0.747847000	-2.962955000
C	-0.843477000	-1.490084000	1.520660000
H	-0.295461000	-1.718500000	2.442843000
H	-1.833300000	-1.937907000	1.605985000
H	3.896600000	-1.142662000	-1.560570000
C	-2.829753000	0.724330000	-1.897392000
C	-4.113926000	0.975250000	-1.423839000
H	2.454813000	3.321235000	0.805951000
H	-1.226187000	0.452275000	2.333515000

Table S66. XYZ of P3 H c 2.

O	-0.283869000	-3.450641000	0.324234000
N	4.131806000	1.802970000	-0.756745000
H	-4.841431000	1.424801000	-2.134449000
N	0.997832000	-1.605209000	-0.017758000
C	-2.045092000	0.417968000	0.372458000
C	0.849423000	1.904736000	1.018422000
H	-3.166911000	-0.296979000	-2.756639000
C	-0.065172000	-2.292443000	0.566743000
C	2.896802000	1.414295000	-0.239374000
C	2.077116000	2.298925000	0.462725000
C	-0.946245000	0.051938000	1.358118000

H	4.424945000	1.296607000	-1.579667000
C	2.460302000	0.081531000	-0.357575000
C	2.968122000	-1.127275000	-0.976680000
H	4.242322000	2.798823000	-0.880407000
C	1.239111000	-0.259624000	0.226046000
C	0.391156000	0.601884000	0.905639000
H	-1.391132000	-0.926143000	-1.187348000
C	-2.120175000	-0.178850000	-0.889036000
H	-2.947180000	1.855118000	1.688260000
C	-3.121124000	0.179426000	-1.783753000
H	2.067216000	-3.159631000	-1.031445000
C	2.064808000	-2.117504000	-0.754919000
H	0.242728000	2.645841000	1.530003000
C	-2.991164000	1.384321000	0.710577000
H	-4.720062000	2.502452000	0.097031000
C	-0.840605000	-1.473530000	1.589575000
H	-0.312215000	-1.647841000	2.534449000
H	-1.826713000	-1.928507000	1.681350000
H	3.899396000	-1.249518000	-1.509117000
C	-3.992458000	1.749748000	-0.185581000
C	-4.061077000	1.146343000	-1.435418000
H	2.396921000	3.330229000	0.573765000
H	-1.209464000	0.502222000	2.319427000

Table S67. XYZ of P3 H c 3.

O	-0.163888000	-3.488154000	0.039157000
N	4.022693000	2.013766000	-0.647147000
H	-4.837704000	1.492097000	-2.084683000
N	1.066955000	-1.584034000	-0.110848000
C	-2.063447000	0.327831000	0.377342000
C	0.754261000	1.841079000	1.148649000
H	-4.779122000	2.371671000	0.234030000
C	0.008978000	-2.346344000	0.378893000
C	2.845880000	1.515218000	-0.089160000
C	1.977724000	2.317974000	0.651915000
C	-0.967033000	-0.098601000	1.340457000
H	4.773596000	1.342811000	-0.721996000
C	2.459079000	0.181465000	-0.315817000
C	3.012352000	-0.952436000	-1.029590000
H	4.345332000	2.880956000	-0.243282000
C	1.251511000	-0.251775000	0.234793000
C	0.357233000	0.527918000	0.952417000
H	-3.018779000	1.625792000	1.796288000
C	-3.036084000	1.240747000	0.780925000
H	-1.352854000	-0.861738000	-1.279159000
C	-4.030294000	1.661566000	-0.098896000
H	2.188501000	-3.005803000	-1.254333000
C	2.150704000	-1.992923000	-0.886751000
H	0.106044000	2.523056000	1.690721000

C	-2.104283000	-0.158560000	-0.932154000
H	-3.114718000	-0.133591000	-2.823169000
C	-0.829419000	-1.637132000	1.433841000
H	-0.344992000	-1.891363000	2.383945000
H	-1.807618000	-2.118034000	1.433936000
H	3.930958000	-0.984317000	-1.595961000
C	-3.096835000	0.255882000	-1.811508000
C	-4.063739000	1.169108000	-1.397839000
H	2.251992000	3.351910000	0.836353000
H	-1.249703000	0.256170000	2.335715000

Table S68. XYZ of P3 H c 4.

O	0.283091000	-3.739226000	0.396384000
N	4.208175000	1.943499000	-0.364612000
H	-5.509207000	2.238380000	-1.024788000
N	1.422730000	-1.776079000	0.259939000
C	-2.269611000	0.435255000	0.139099000
C	0.507773000	1.766680000	0.100539000
H	-5.268792000	1.506923000	1.332450000
C	0.256997000	-2.543103000	0.270861000
C	2.928121000	1.439901000	-0.140224000
C	1.806376000	2.267897000	-0.090841000
C	-1.021827000	-0.296224000	0.584481000
H	4.957308000	1.388959000	0.023423000
C	2.715730000	0.053386000	-0.019108000
C	3.553597000	-1.129622000	-0.006157000
H	4.322184000	2.921996000	-0.144049000
C	1.404417000	-0.392705000	0.148225000
C	0.272685000	0.407366000	0.233316000
H	-3.206543000	0.363011000	2.069116000
C	-3.308187000	0.678868000	1.035146000
H	-1.602951000	0.689056000	-1.889662000
C	-4.471290000	1.324088000	0.620967000
H	2.963696000	-3.262549000	0.213636000
C	2.744972000	-2.207882000	0.165777000
H	-0.322978000	2.463060000	0.151558000
C	-2.413500000	0.855075000	-1.186003000
H	-3.668467000	1.819368000	-2.634512000
C	-1.006666000	-1.733489000	0.015286000
H	-1.849218000	-2.313643000	0.391525000
H	-1.110410000	-1.685623000	-1.075848000
H	4.625576000	-1.175778000	-0.126741000
C	-3.572201000	1.497993000	-1.603412000
C	-4.606079000	1.734470000	-0.699761000
H	1.944492000	3.339591000	-0.193950000
H	-1.059939000	-0.381054000	1.678450000

Table S69. XYZ of P3 H c 5.

O	0.284276000	-3.739148000	0.396136000
N	4.216399000	1.952743000	-0.257925000
H	-5.509363000	2.240858000	-1.019553000
N	1.422644000	-1.774753000	0.265723000
C	-2.271032000	0.433763000	0.142040000
C	0.505531000	1.767821000	0.114491000
H	-5.271973000	1.500592000	1.335221000
C	0.257814000	-2.542910000	0.271571000
C	2.924935000	1.442021000	-0.138495000
C	1.803026000	2.269443000	-0.084541000
C	-1.023331000	-0.298265000	0.586724000
H	4.871741000	1.345452000	-0.728172000
C	2.715421000	0.056528000	-0.001464000
C	3.556037000	-1.124061000	0.032142000
H	4.266310000	2.895148000	-0.616358000
C	1.403262000	-0.391476000	0.152832000
C	0.271015000	0.407802000	0.240008000
H	-3.210558000	0.354359000	2.070472000
C	-3.310947000	0.673802000	1.037504000
H	-1.601722000	0.695066000	-1.884741000
C	-4.473567000	1.320386000	0.624096000
H	2.968406000	-3.257068000	0.258324000
C	2.747159000	-2.203826000	0.193551000
H	-0.325564000	2.463584000	0.168450000
C	-2.413359000	0.858397000	-1.181686000
H	-3.666598000	1.827824000	-2.628435000
C	-1.005628000	-1.733684000	0.013034000
H	-1.848944000	-2.315536000	0.384912000
H	-1.106008000	-1.682758000	-1.078281000
H	4.632566000	-1.164177000	-0.039809000
C	-3.571584000	1.502634000	-1.598397000
C	-4.606664000	1.735720000	-0.695244000
H	1.940393000	3.341488000	-0.185142000
H	-1.062731000	-0.386706000	1.680316000

Table S70. XYZ of P3 H c 6.

O	-0.299895000	-3.354600000	-0.140801000
N	4.119044000	1.991356000	-0.613900000
H	-4.903719000	1.191364000	-2.088161000
N	1.022926000	-1.505689000	-0.214732000
C	-2.004947000	0.355905000	0.363061000
C	0.890833000	1.843508000	1.254863000
H	-5.393821000	0.662786000	0.287352000
C	-0.052551000	-2.252379000	0.271105000
C	2.939535000	1.505717000	-0.051991000
C	2.123157000	2.297794000	0.757119000
C	-0.906094000	-0.035114000	1.345160000

H	4.832293000	1.294418000	-0.771855000
C	2.494007000	0.202664000	-0.343926000
C	2.970429000	-0.903996000	-1.150108000
H	4.498357000	2.808967000	-0.159225000
C	1.283119000	-0.211942000	0.212913000
C	0.435607000	0.561300000	0.993138000
H	-3.550861000	0.127793000	1.838948000
C	-3.331330000	0.359395000	0.800044000
H	-0.714371000	0.686631000	-1.328239000
C	-4.370943000	0.658542000	-0.072546000
H	2.039965000	-2.896147000	-1.486153000
C	2.061423000	-1.909831000	-1.051068000
H	0.282229000	2.519596000	1.847351000
C	-1.739062000	0.662163000	-0.971458000
H	-2.555605000	1.193379000	-2.883259000
C	-0.783877000	-1.577394000	1.423562000
H	-0.212324000	-1.844872000	2.320584000
H	-1.762854000	-2.049507000	1.502354000
H	3.870235000	-0.941139000	-1.745588000
C	-2.778997000	0.957097000	-1.848897000
C	-4.096199000	0.956656000	-1.404027000
H	2.443932000	3.307847000	0.991870000
H	-1.211306000	0.312705000	2.336715000

NMR spectra

1*H*-Indol-4-amine (1a)

^{1}H NMR of **1a** (400 MHz, DMSO-d_6)

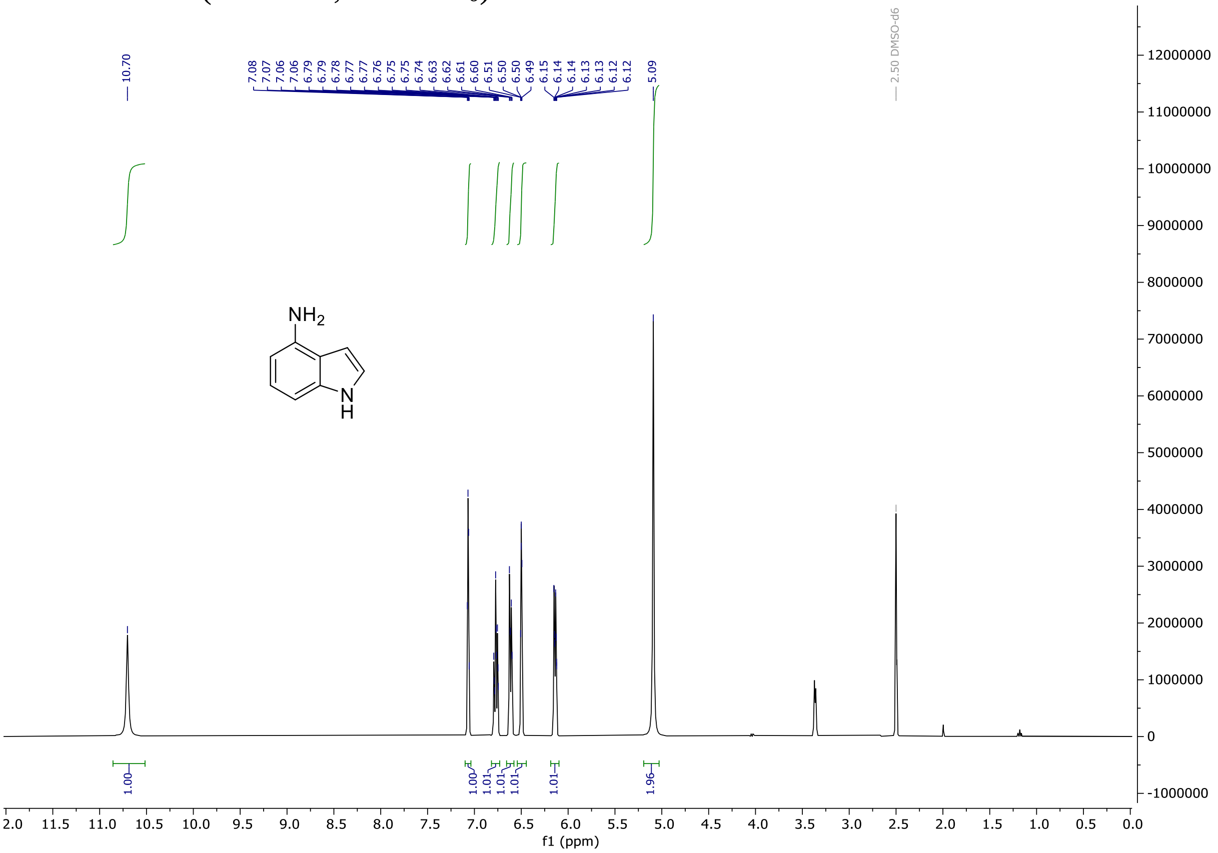

^{13}C{^{1}H} NMR of **1a** (101 MHz, DMSO-d_6)

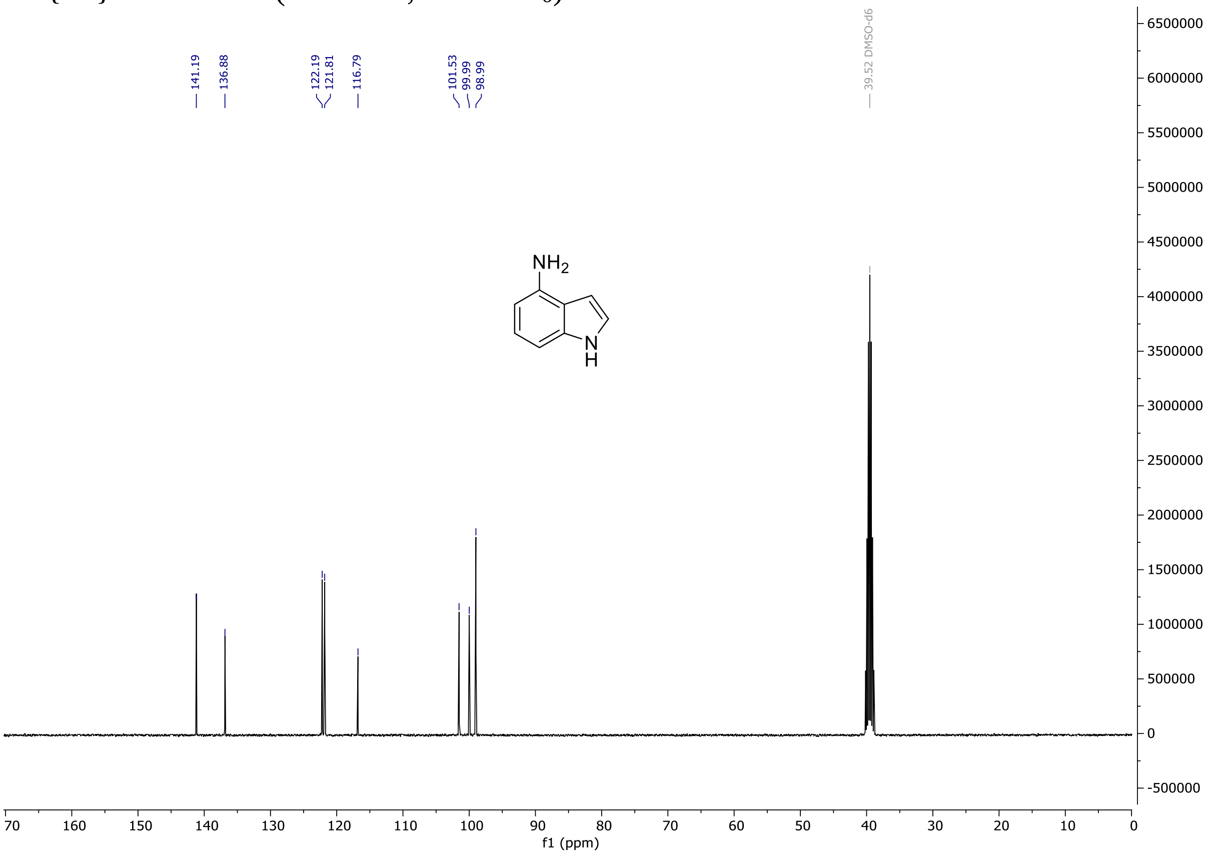

1-Methyl-1*H*-indol-4-amine (1b)

^{1}H NMR of **1b** (400 MHz, $CDCl_3$)

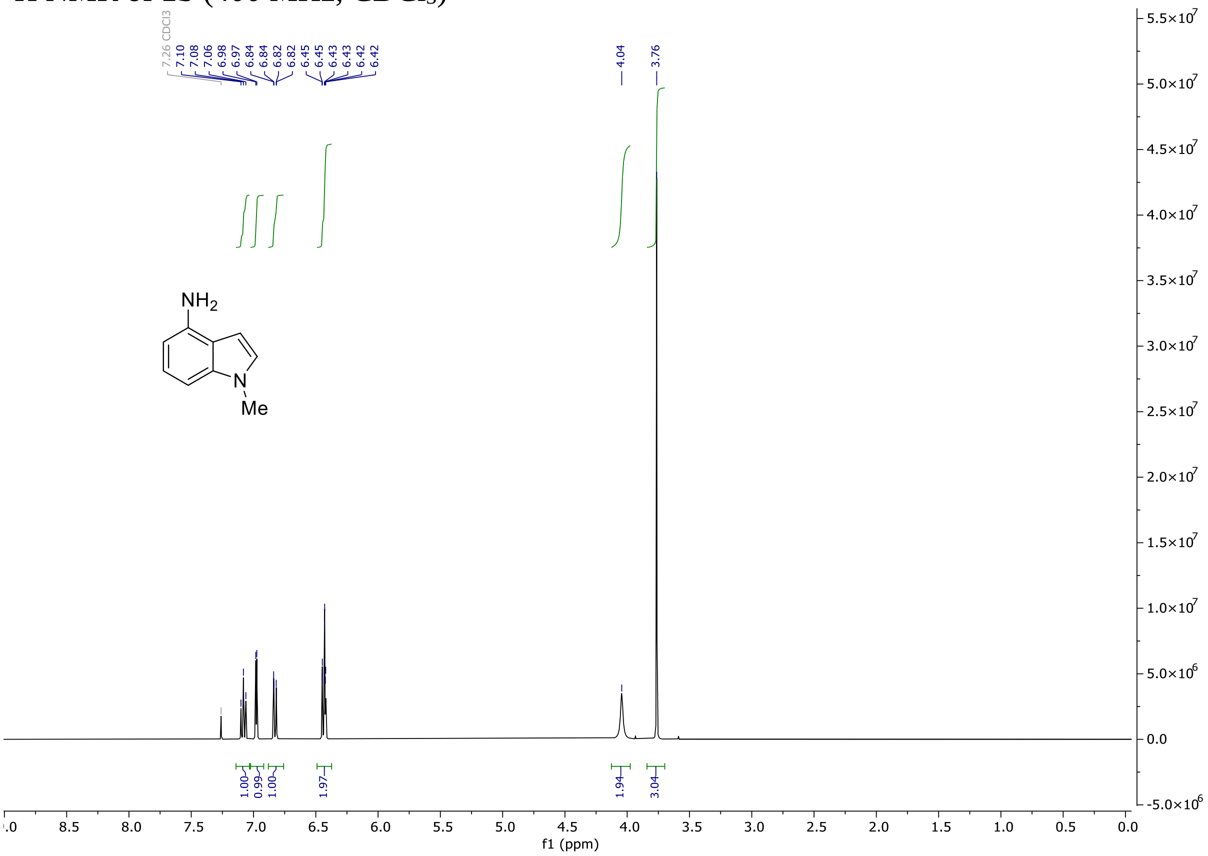

^{13}C{^{1}H} NMR of **1b** (101 MHz, $CDCl_3$)

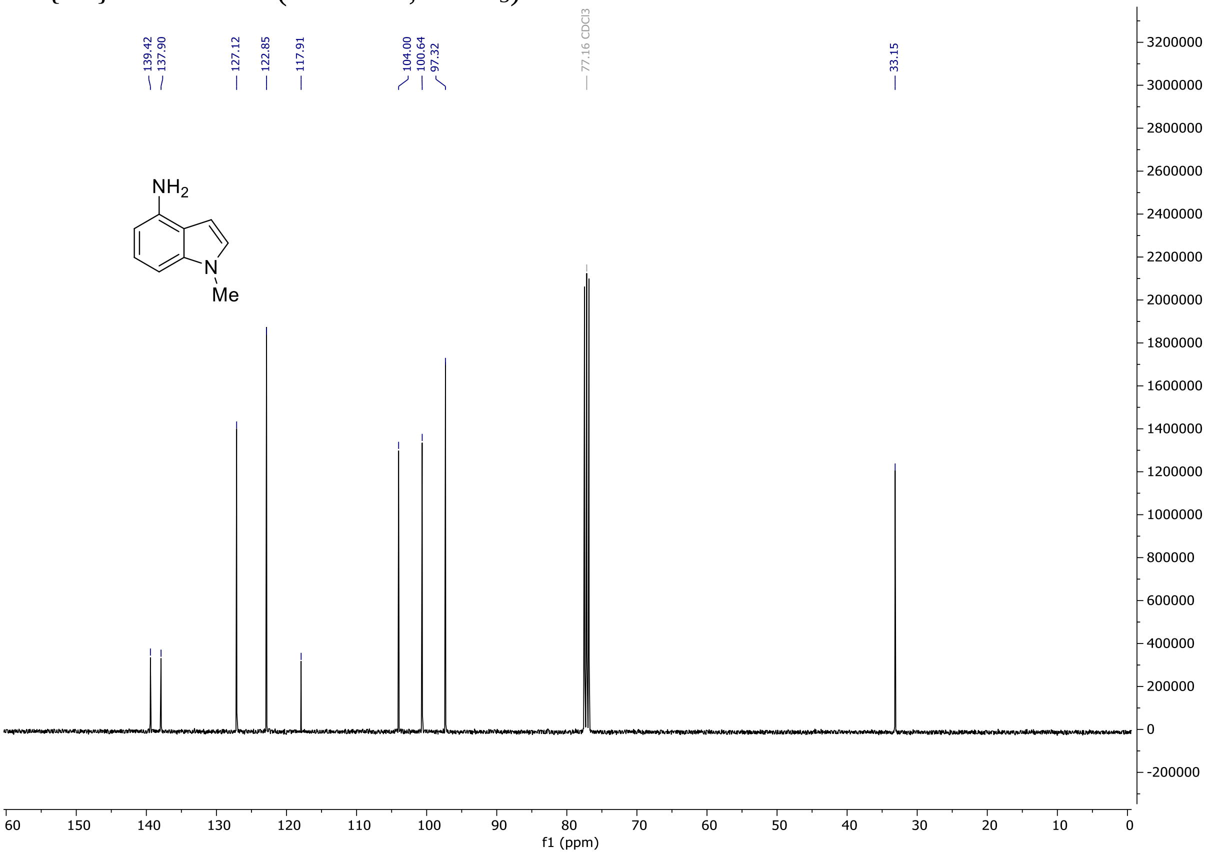

1-Propyl-1*H*-indol-4-amine (1c)

^{1}H NMR of **1c** (400 MHz, $CDCl_3$)

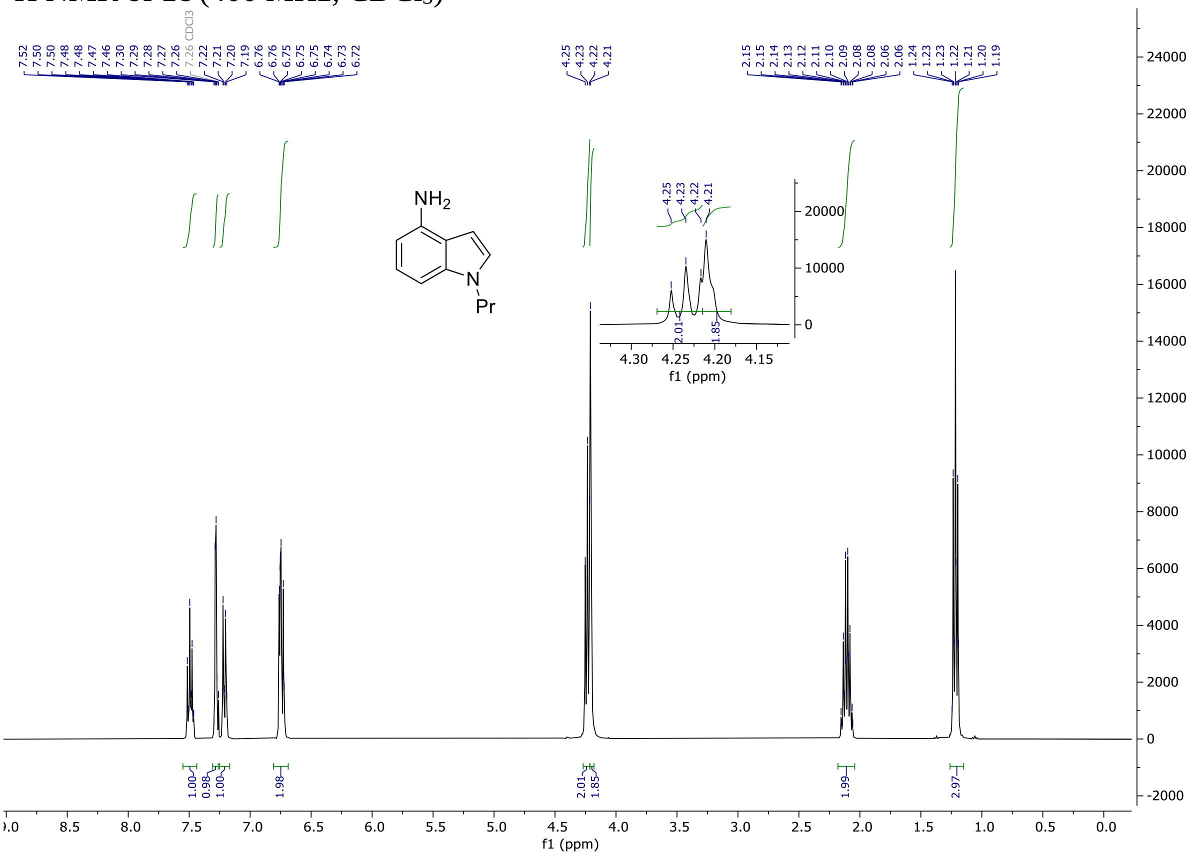

^{13}C{^{1}H} NMR of **1c** (101 MHz, $CDCl_3$)

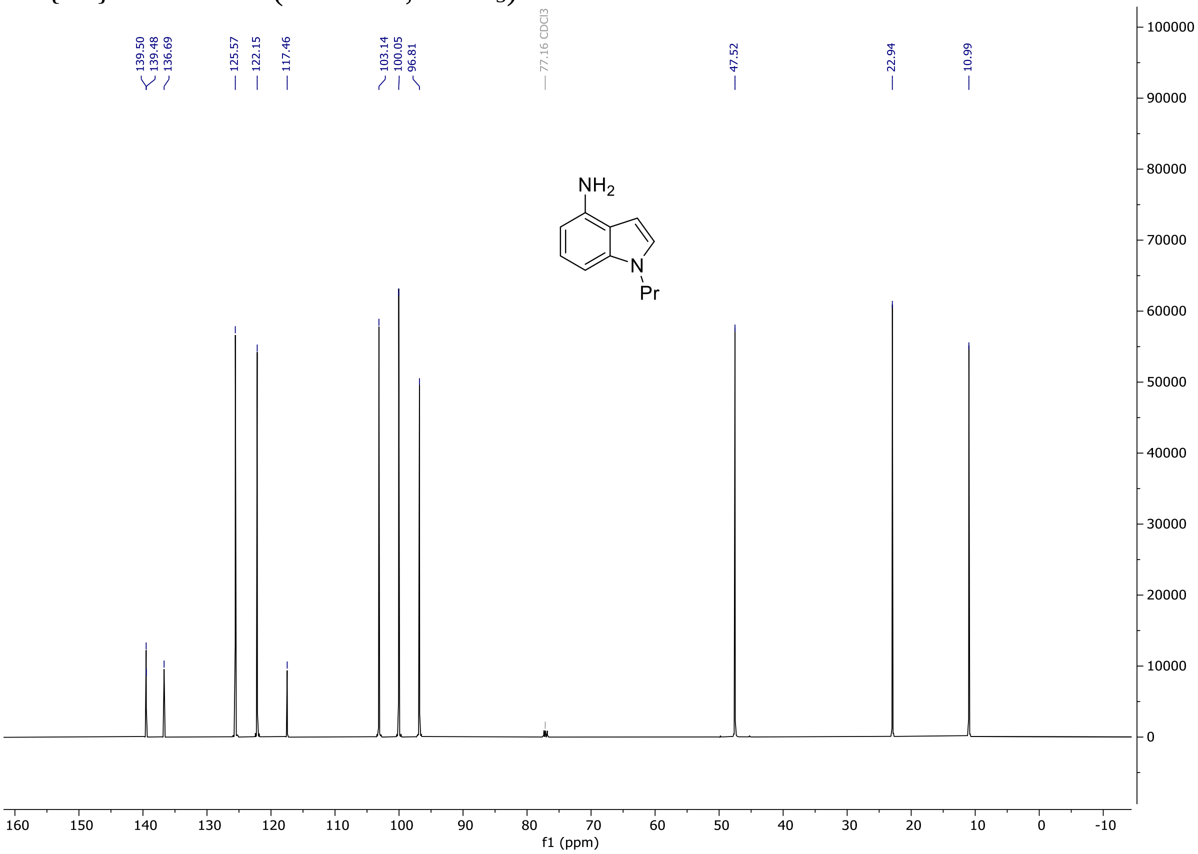

1-Allyl-1*H*-indol-4-amine (1d)

^{1}H NMR of **1d** (400 MHz, $CDCl_3$)

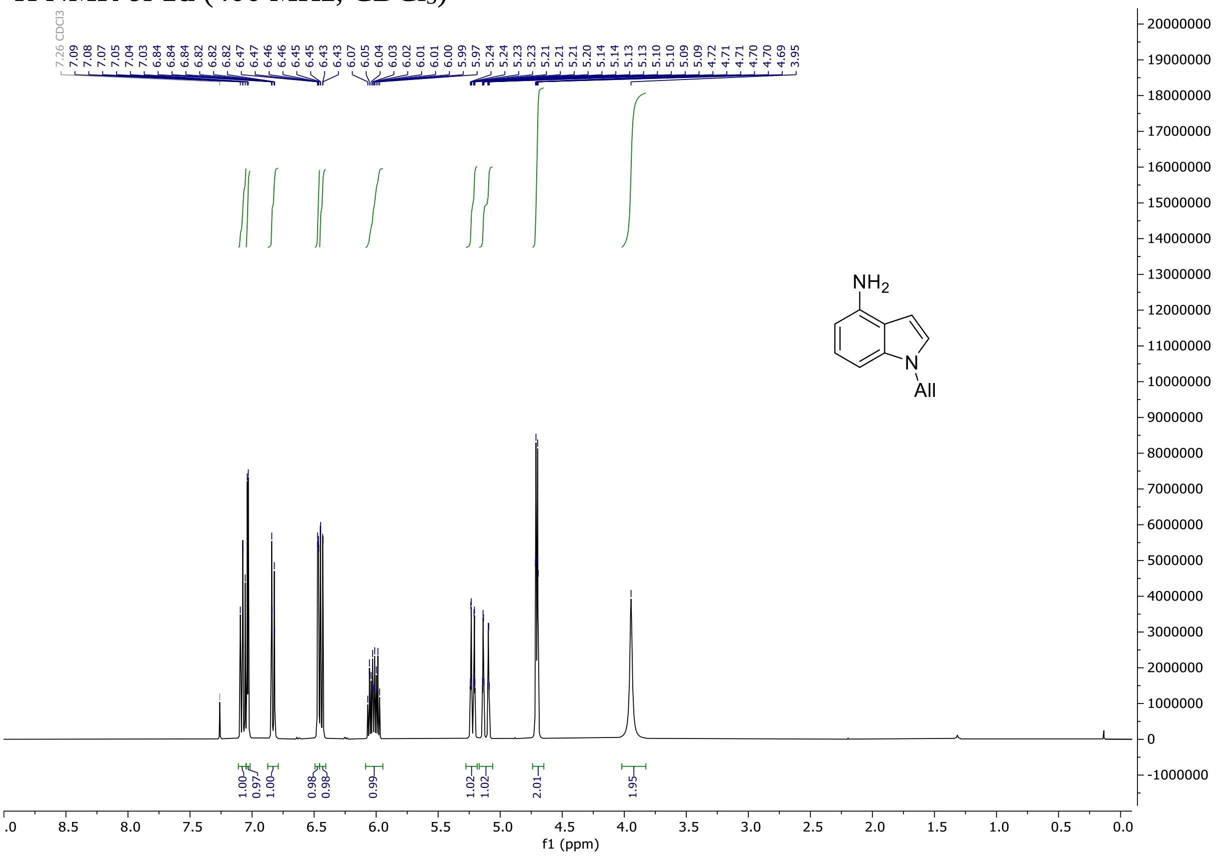

^{13}C{^{1}H} NMR of **1d** (101 MHz, $CDCl_3$)

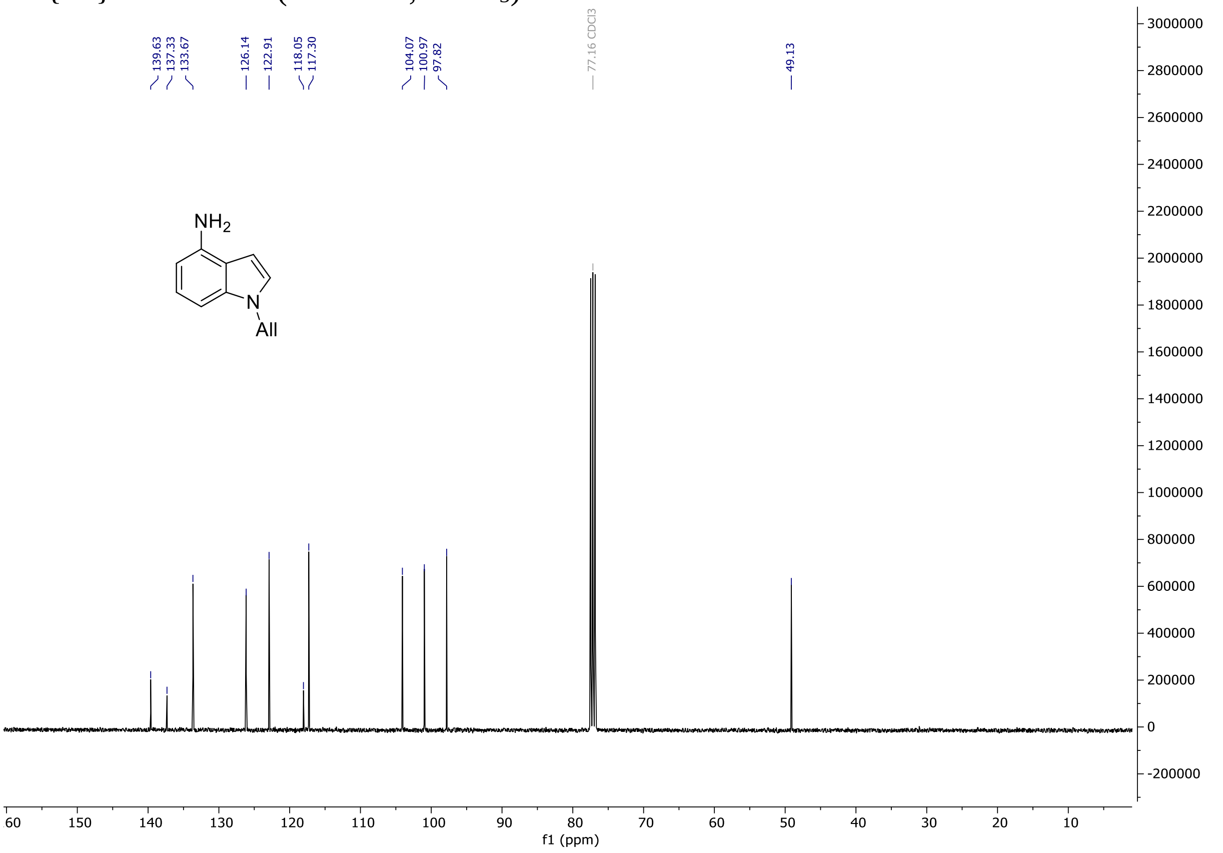

1-Benzyl-1*H*-indol-4-amine (1e)

^{1}H NMR of **1e** (400 MHz, $CDCl_3$)

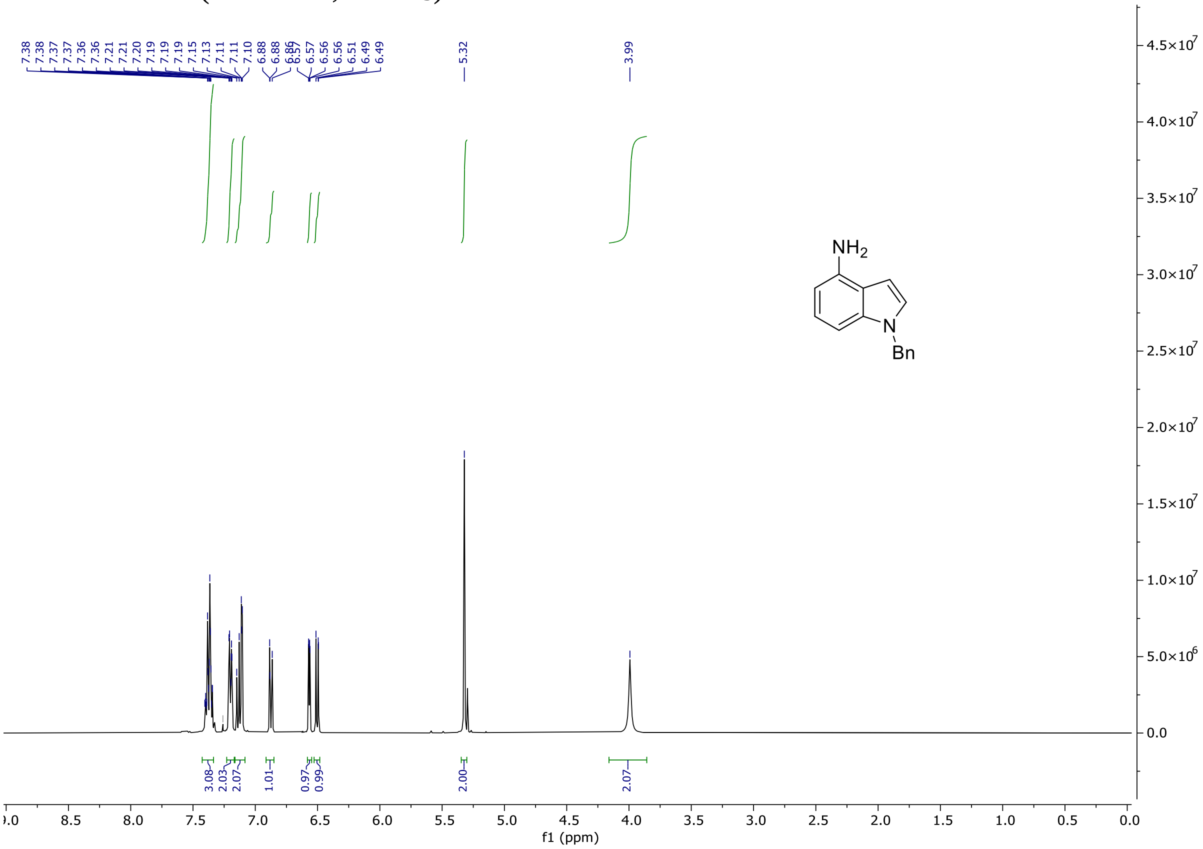

$^{13}C\{^{1}H\}$ NMR of **1e** (101 MHz, $CDCl_3$)

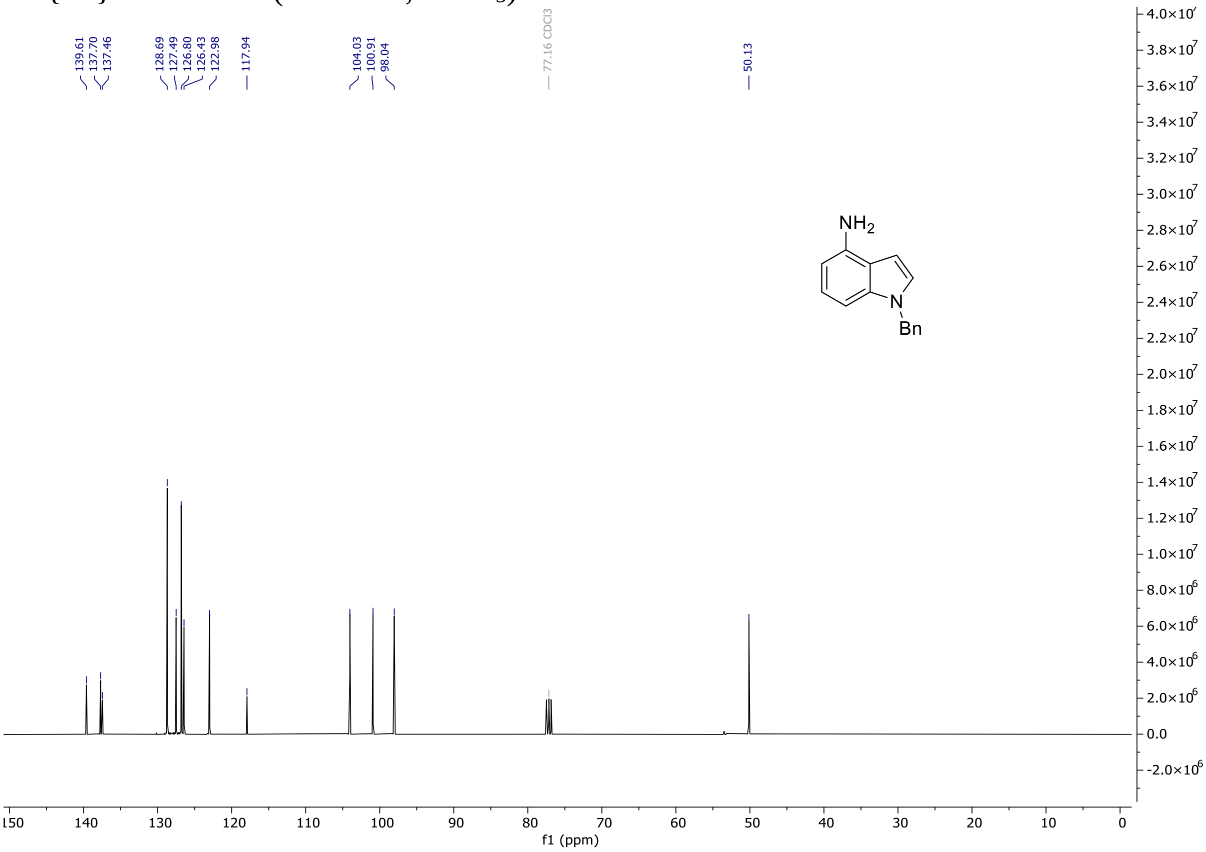

1,3-Dimethyl-1*H*-indol-4-amine (1f)

^{1}H NMR of **1f** (400 MHz, $CDCl_3$)

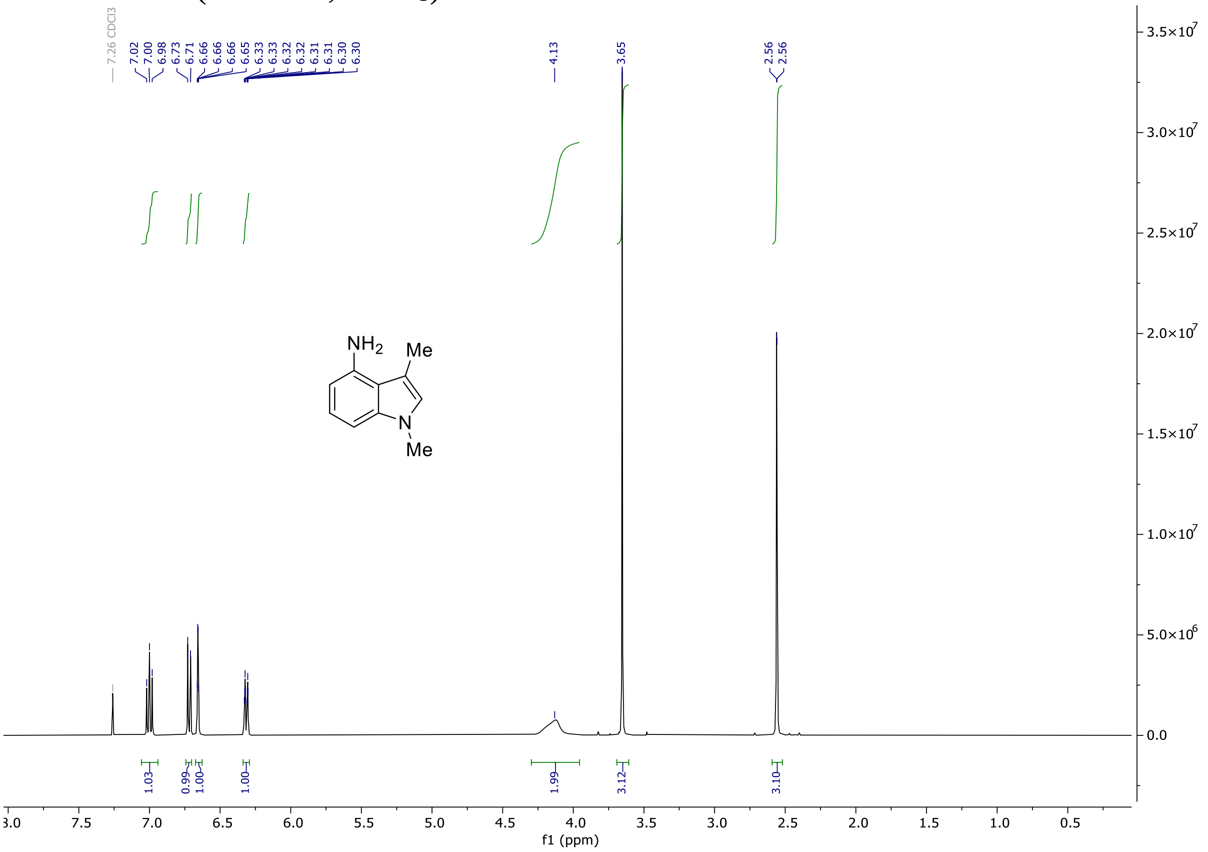

^{13}C{^{1}H} NMR of **1f** (101 MHz, $CDCl_3$)

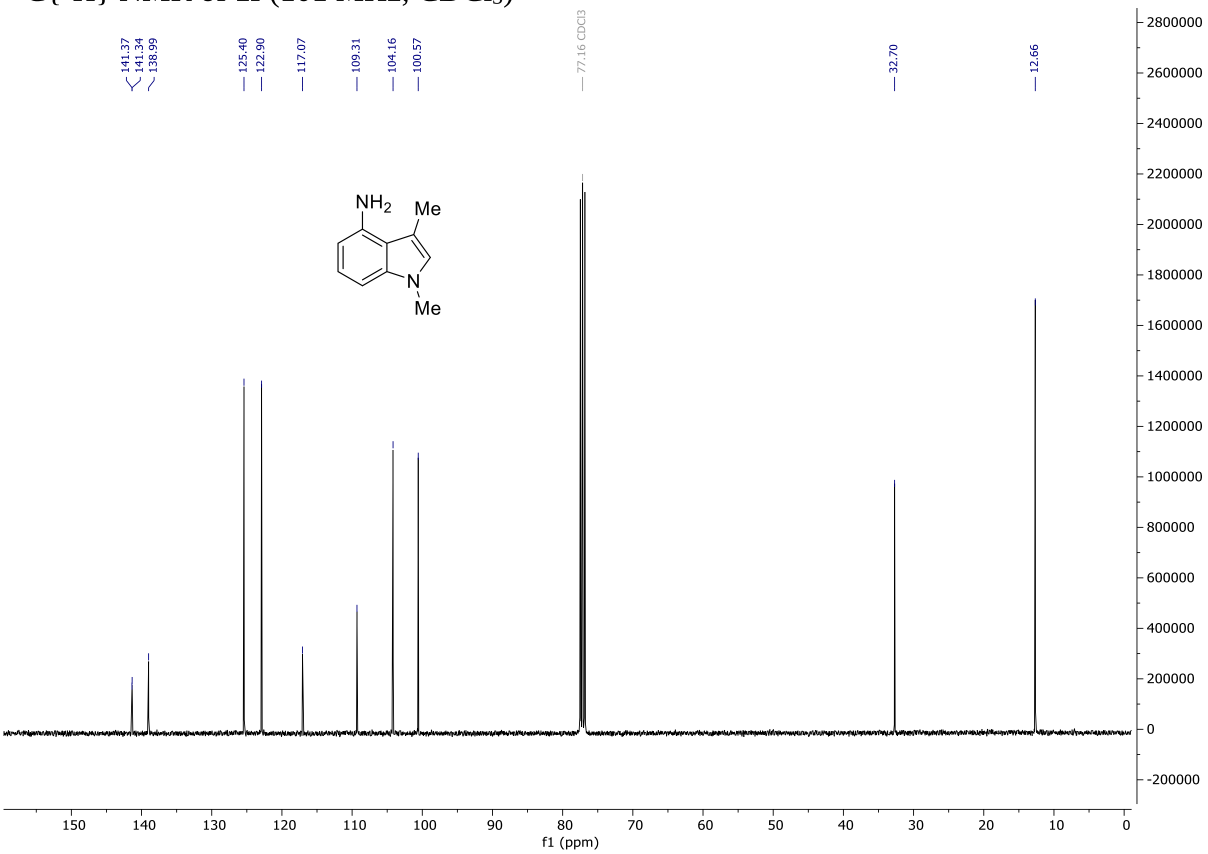

***N*-Benzyl-1-methyl-1*H*-indol-4-amine (1g)**

^{1}H NMR of **1g** (400 MHz, $CDCl_3$)

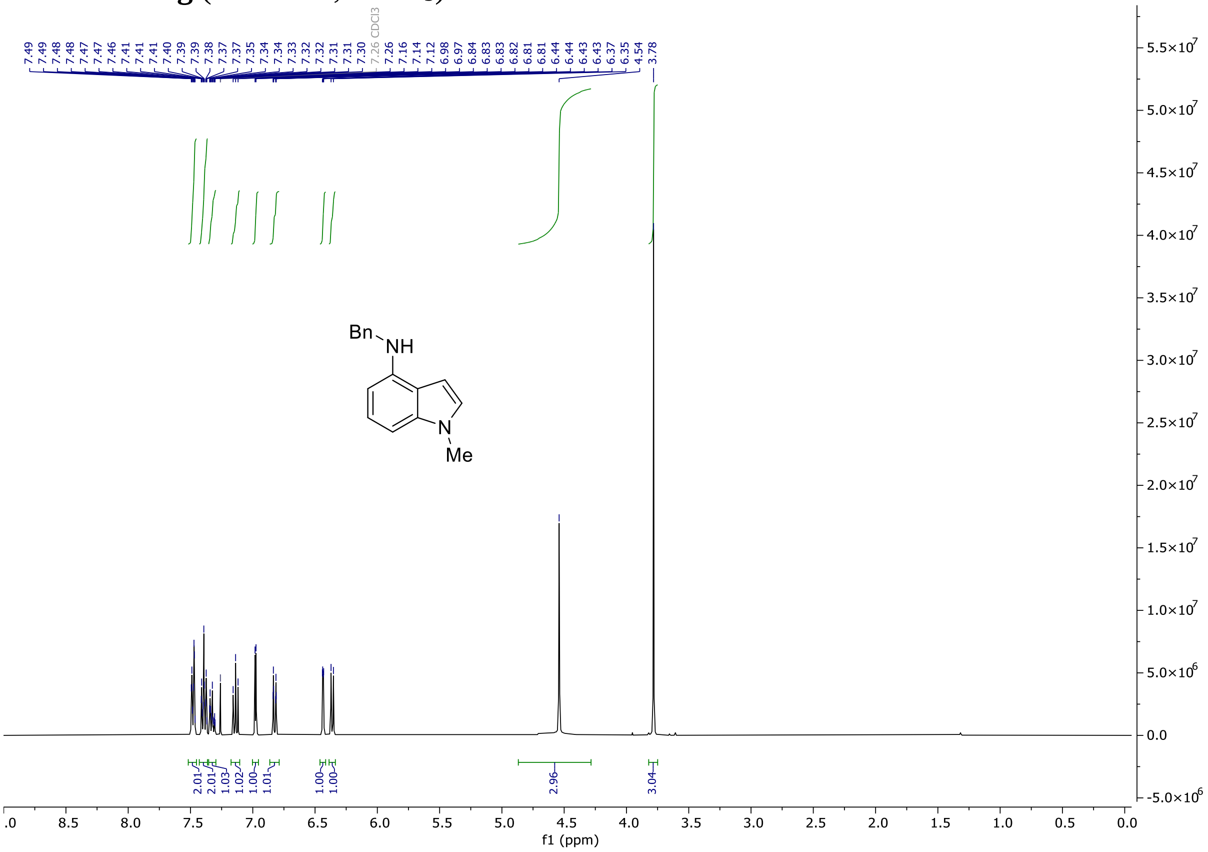

^{13}C{^{1}H} NMR of **1g** (101 MHz, $CDCl_3$)

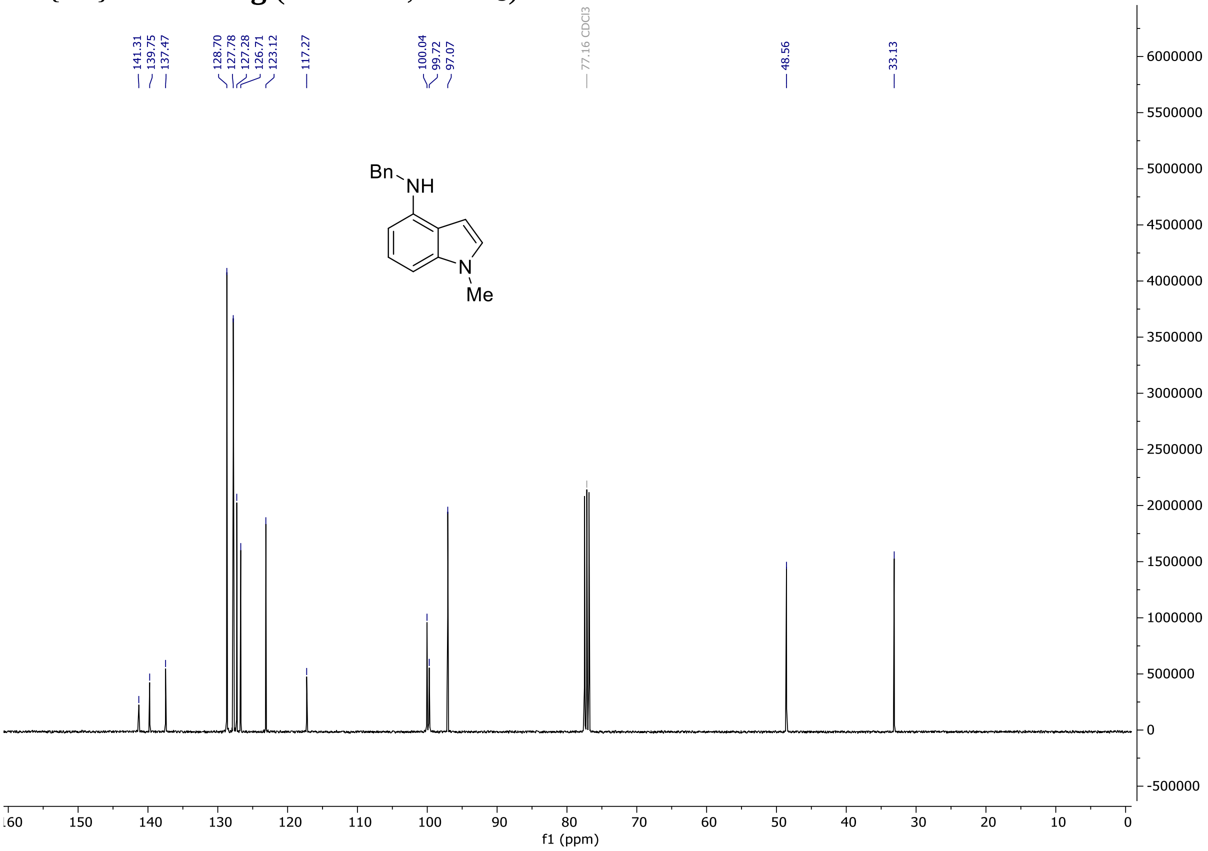

4-Methyl-*N*-(1-methyl-1*H*-indol-4-yl)benzenesulfonamide (1h)

^{1}H NMR of **1h** (400 MHz, $CDCl_3$)

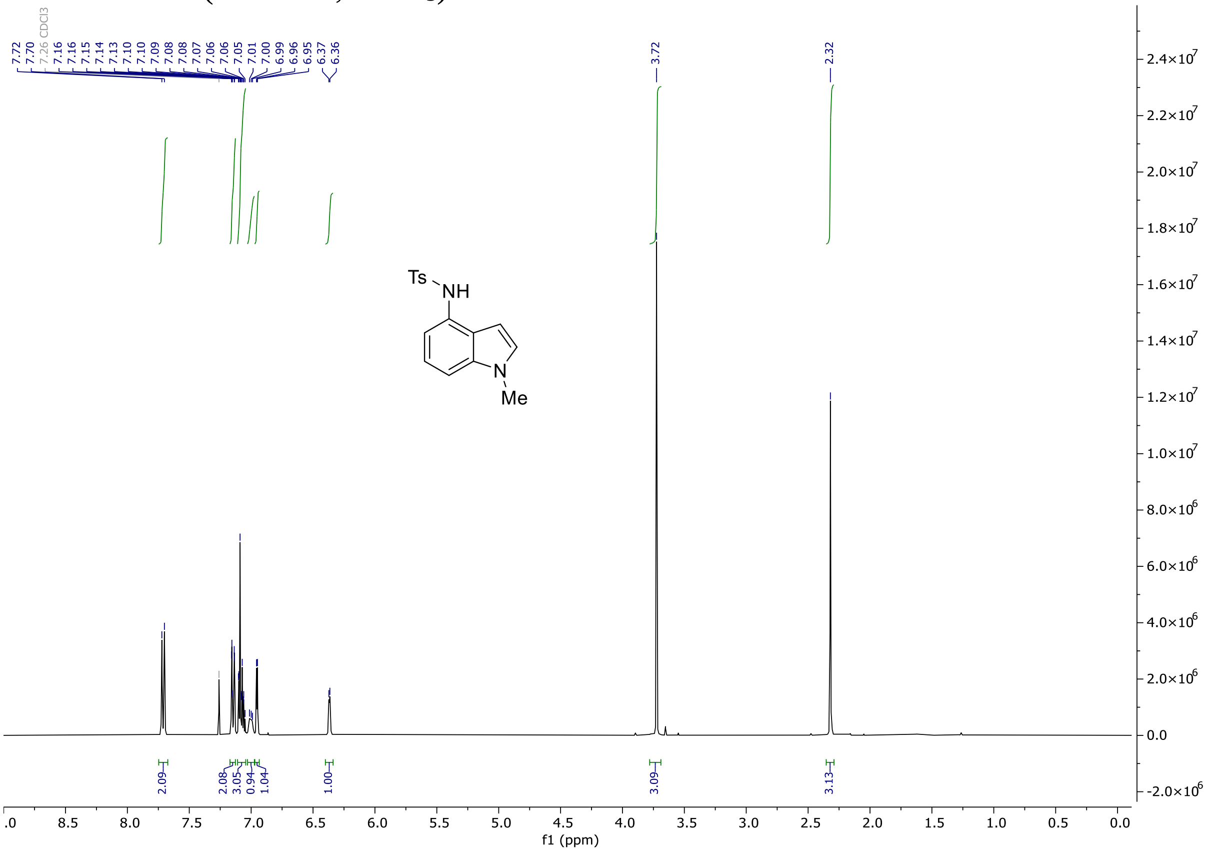

^{13}C{^{1}H} NMR of **1h** (101 MHz, $CDCl_3$)

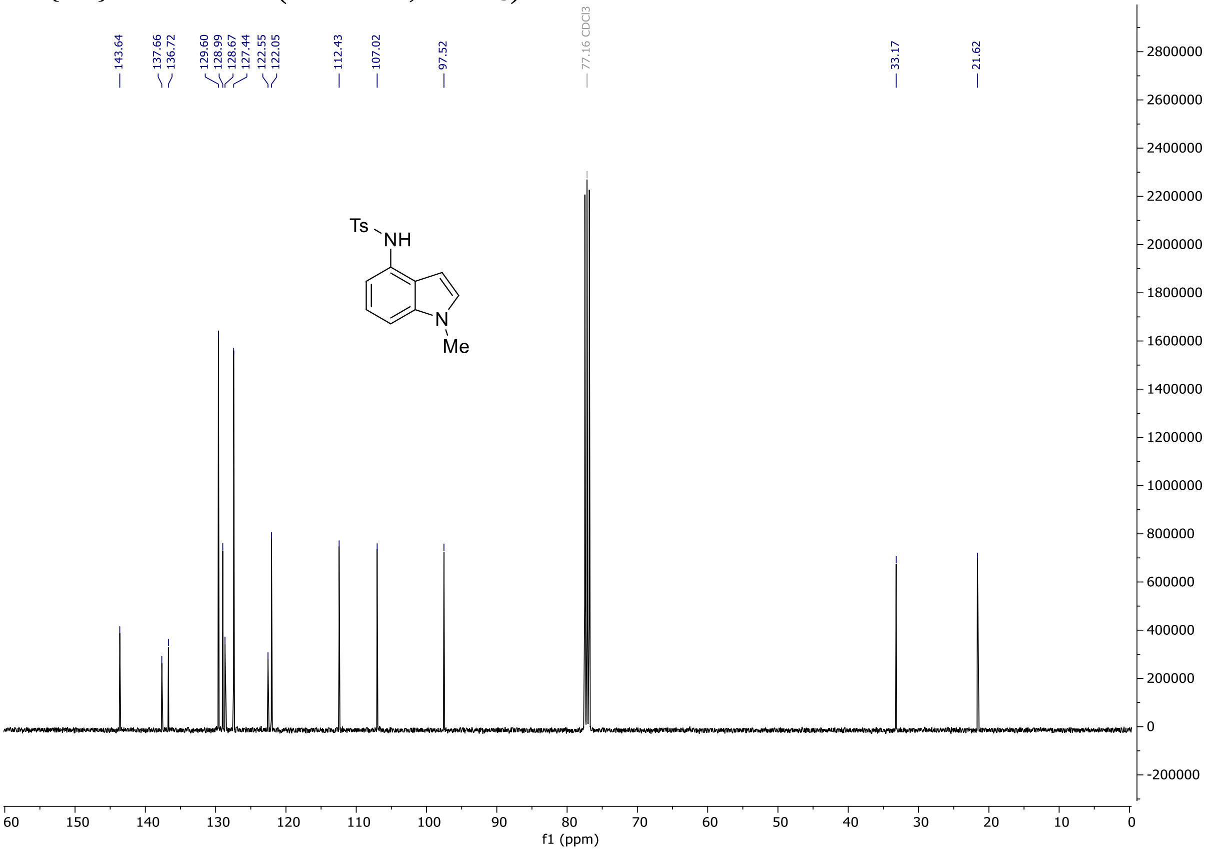

tert-Butyl 4-amino-1_H_-indole-1-carboxylate (1i)

^{1}H NMR of **1i** (400 MHz, $CDCl_3$)

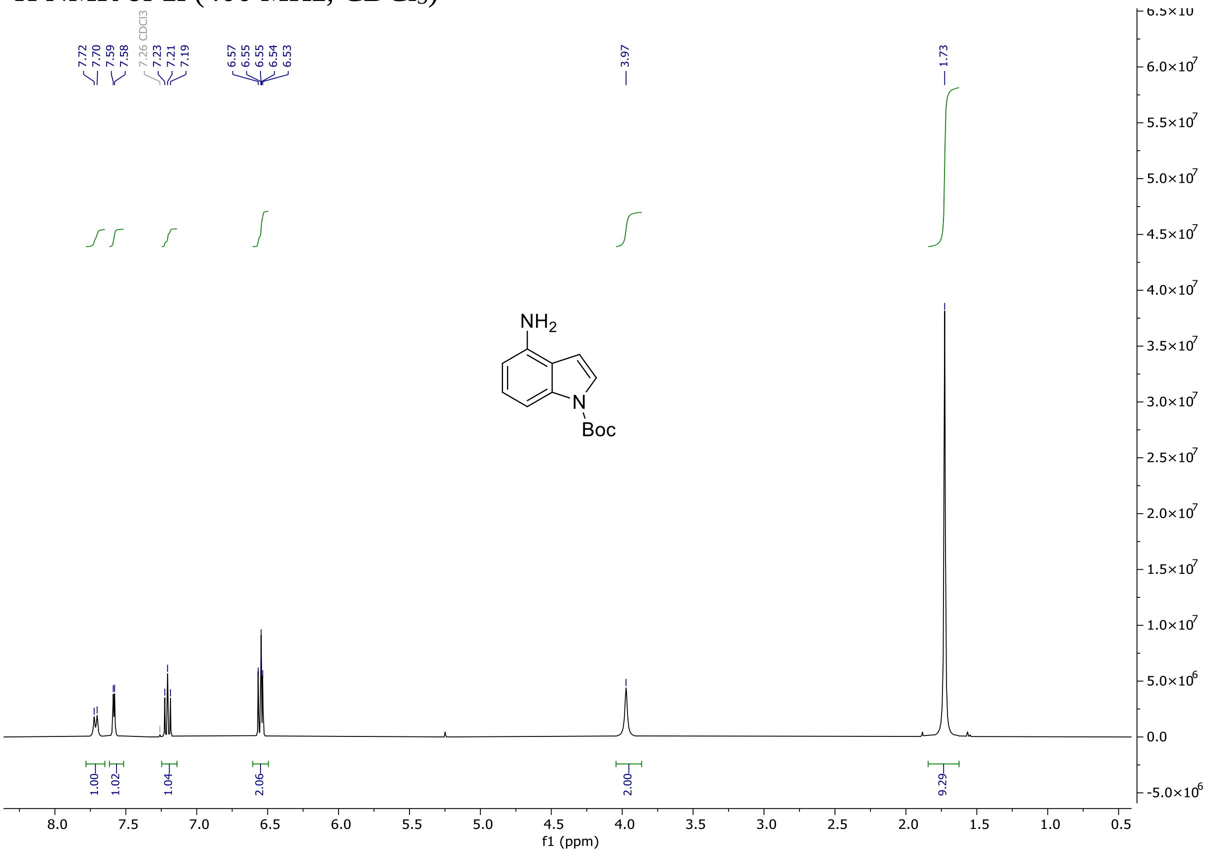

$^{13}C\{^{1}H\}$ NMR of **1i** (101 MHz, $CDCl_3$)

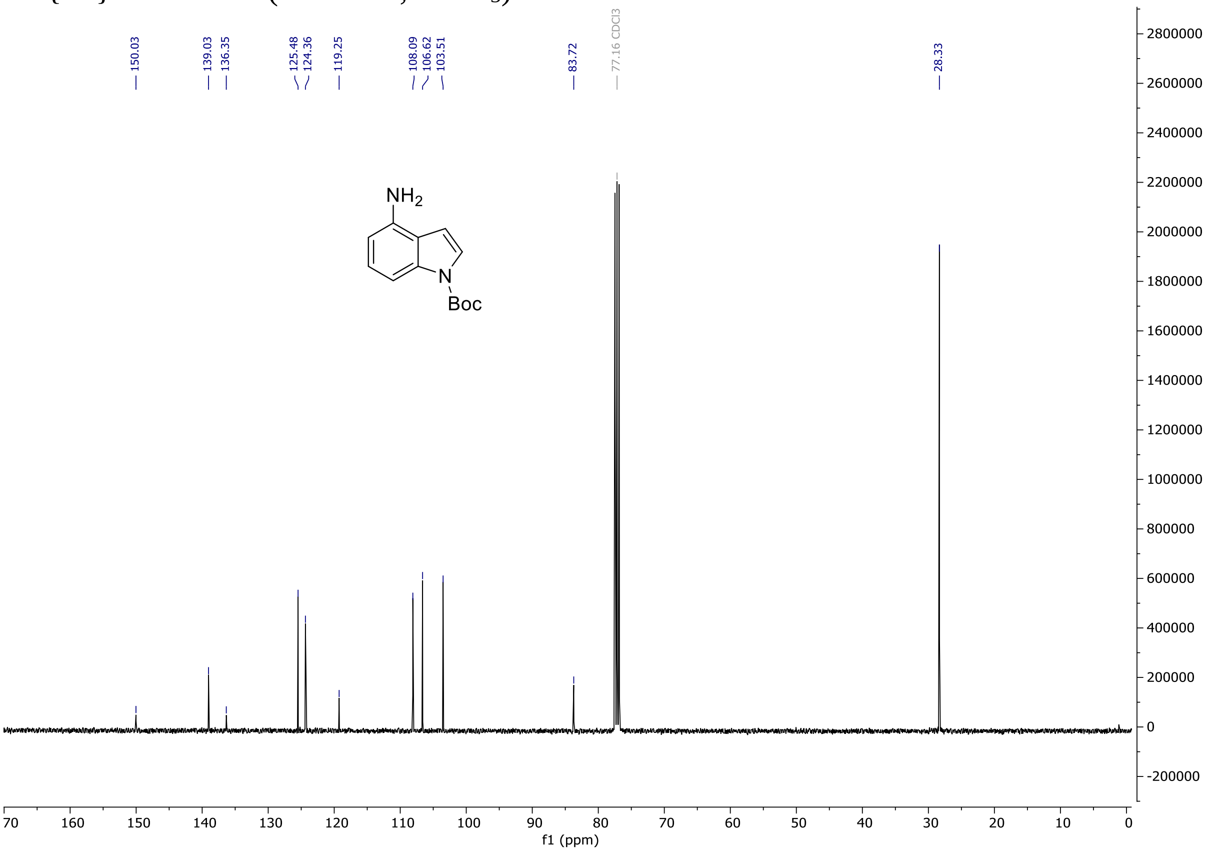

1-Tosyl-1*H*-indol-4-amine (1j)

^{1}H NMR of **1j** (400 MHz, DMSO-d_6)

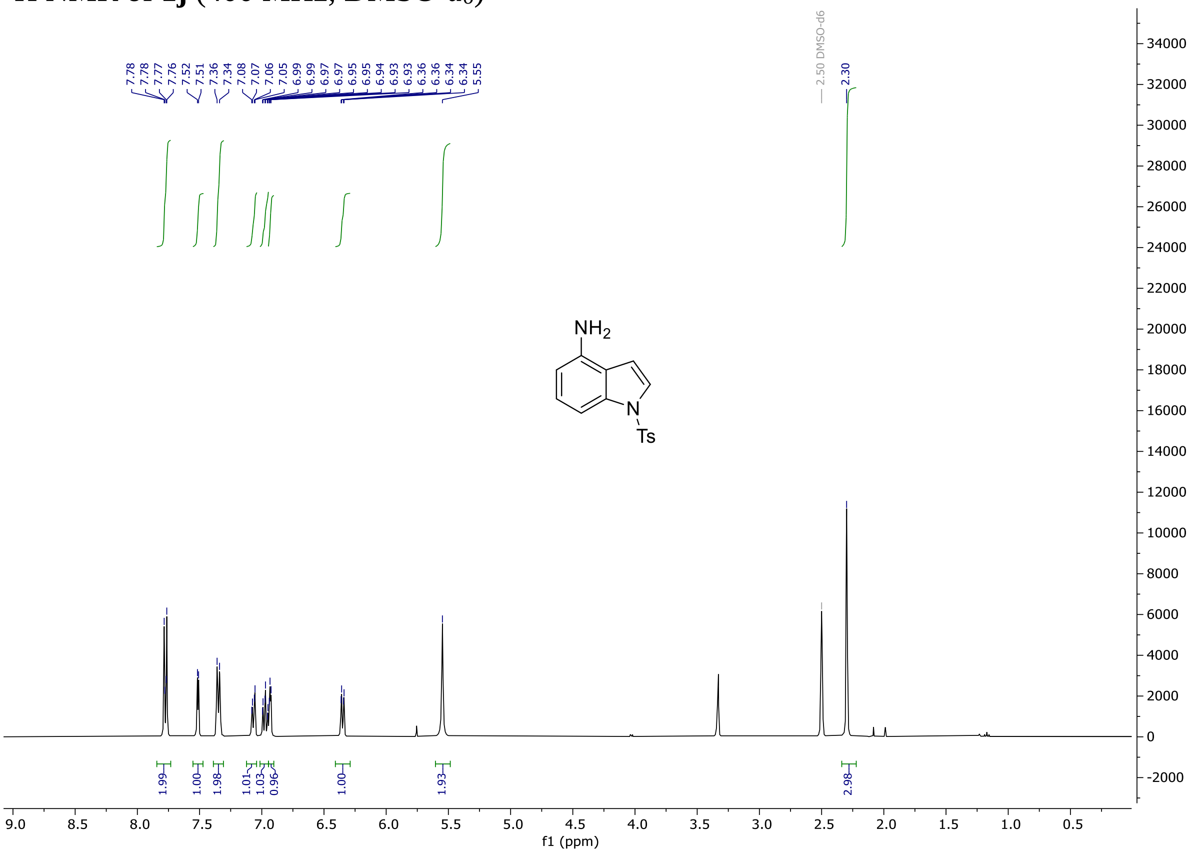

^{13}C{^{1}H} NMR of **1j** (101 MHz, DMSO-d_6)

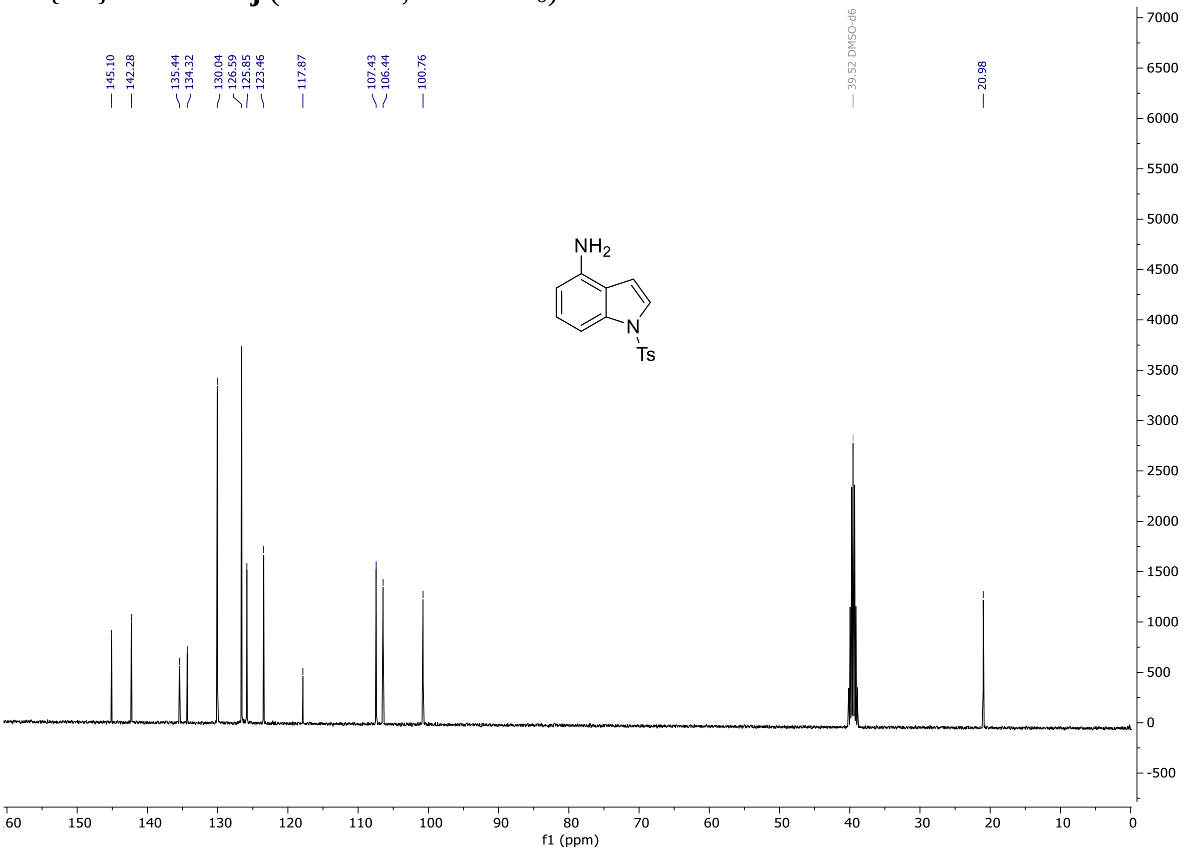

1-Methyl-1*H*-indol-5-amine (1k)

^{1}H NMR of **1k** (400 MHz, $CDCl_3$)

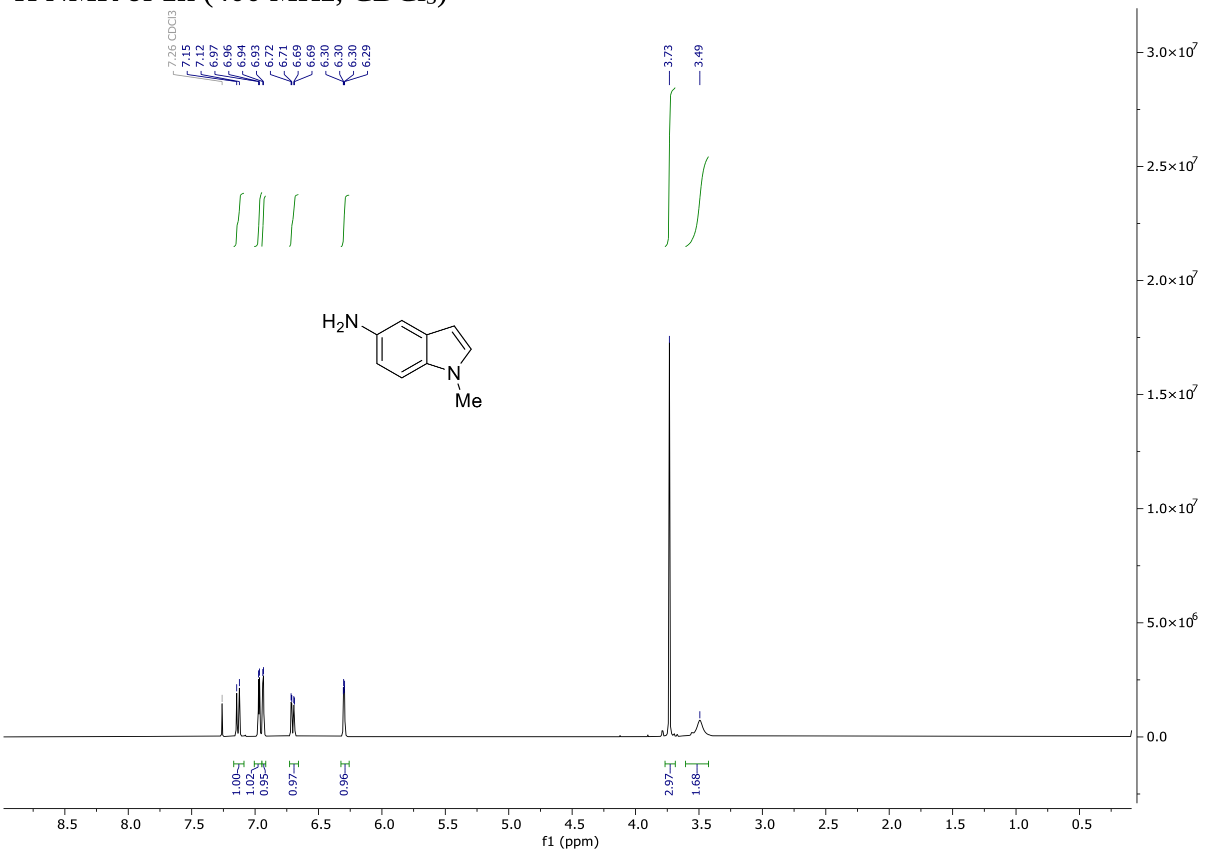

^{13}C{^{1}H} NMR of **1k** (101 MHz, $CDCl_3$)

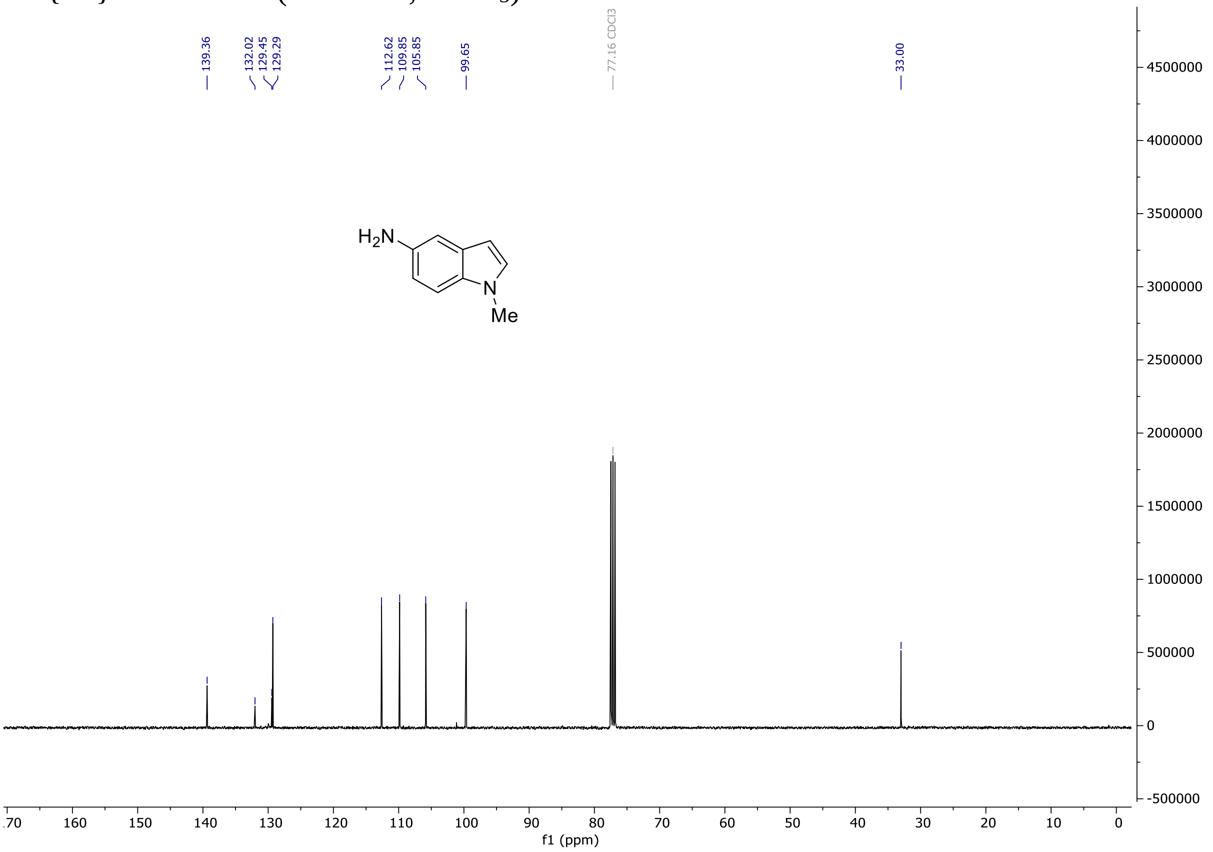

1-Methyl-1*H*-indol-6-amine (1l)

^{1}H NMR of **1l** (400 MHz, $CDCl_3$)

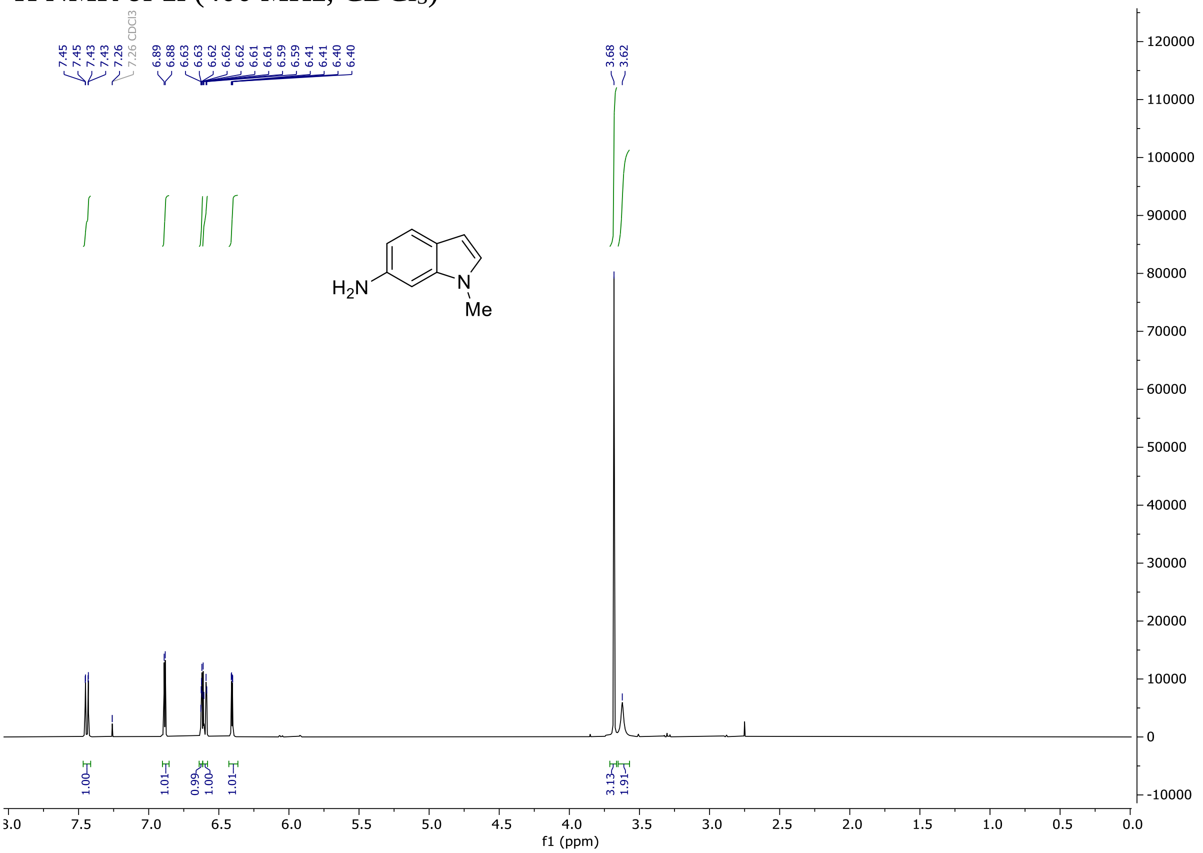

$^{13}C\{^{1}H\}$ NMR of **1l** (101 MHz, $CDCl_3$)

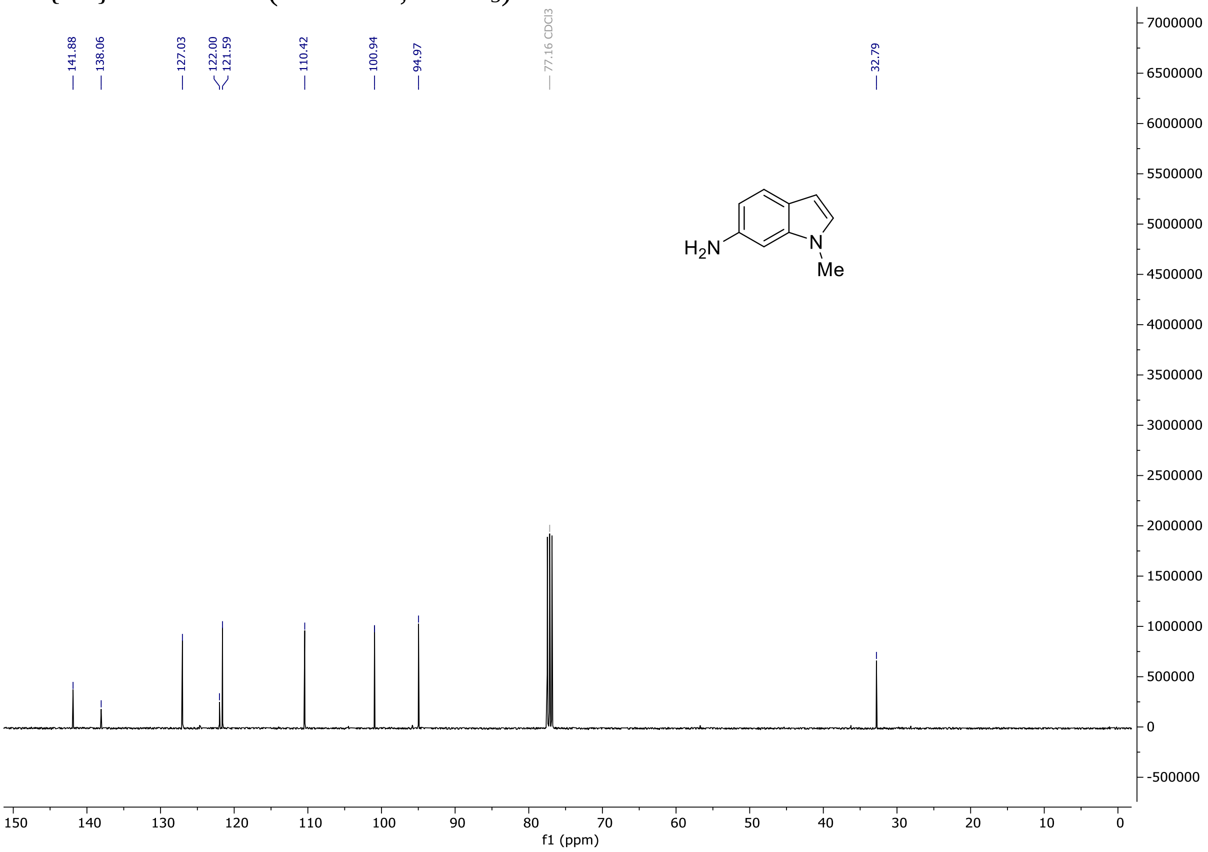

1-Methyl-1*H*-indol-7-amine (1m)

^{1}H NMR of **1m** (400 MHz, $CDCl_3$)

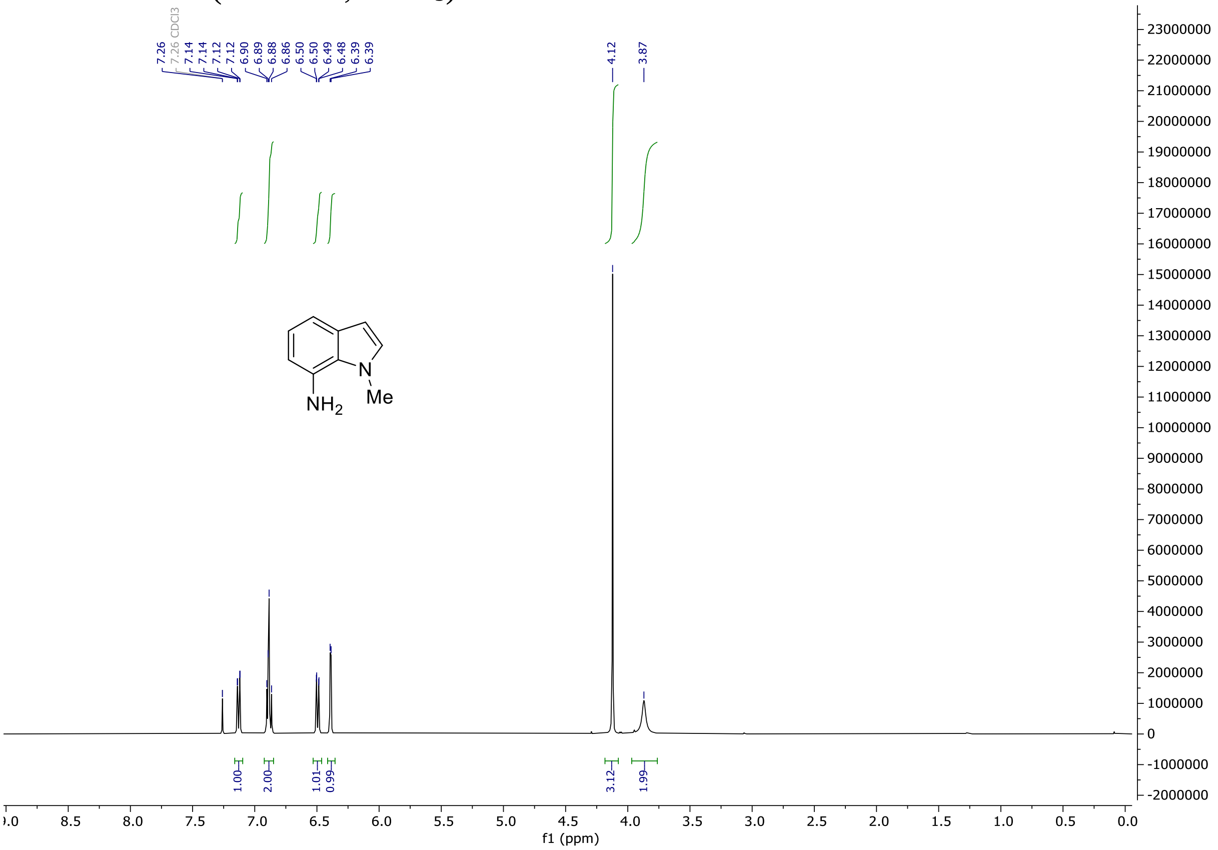

^{13}C{^{1}H} NMR of **1m** (101 MHz, $CDCl_3$)

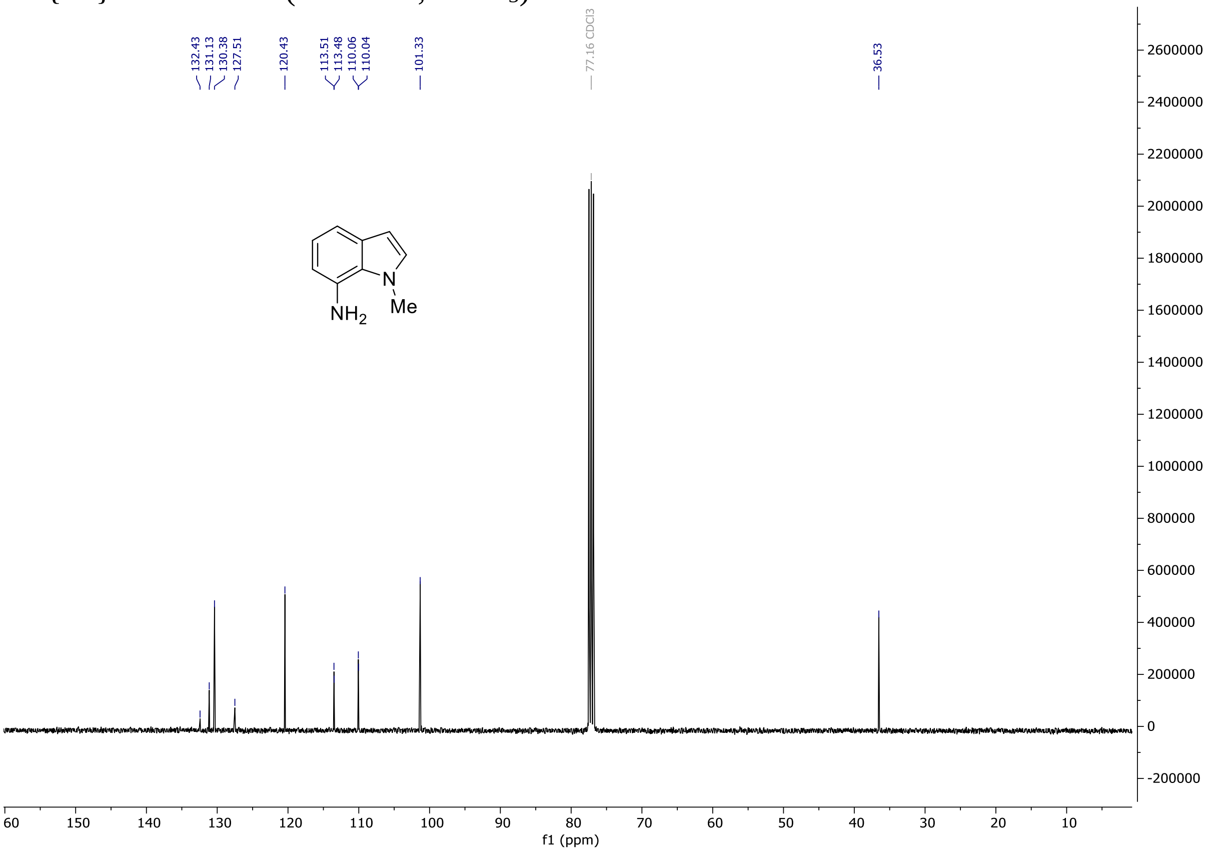

Benzofuran-4-amine (1n)

^{1}H NMR of **1n** (400 MHz, $CDCl_3$)

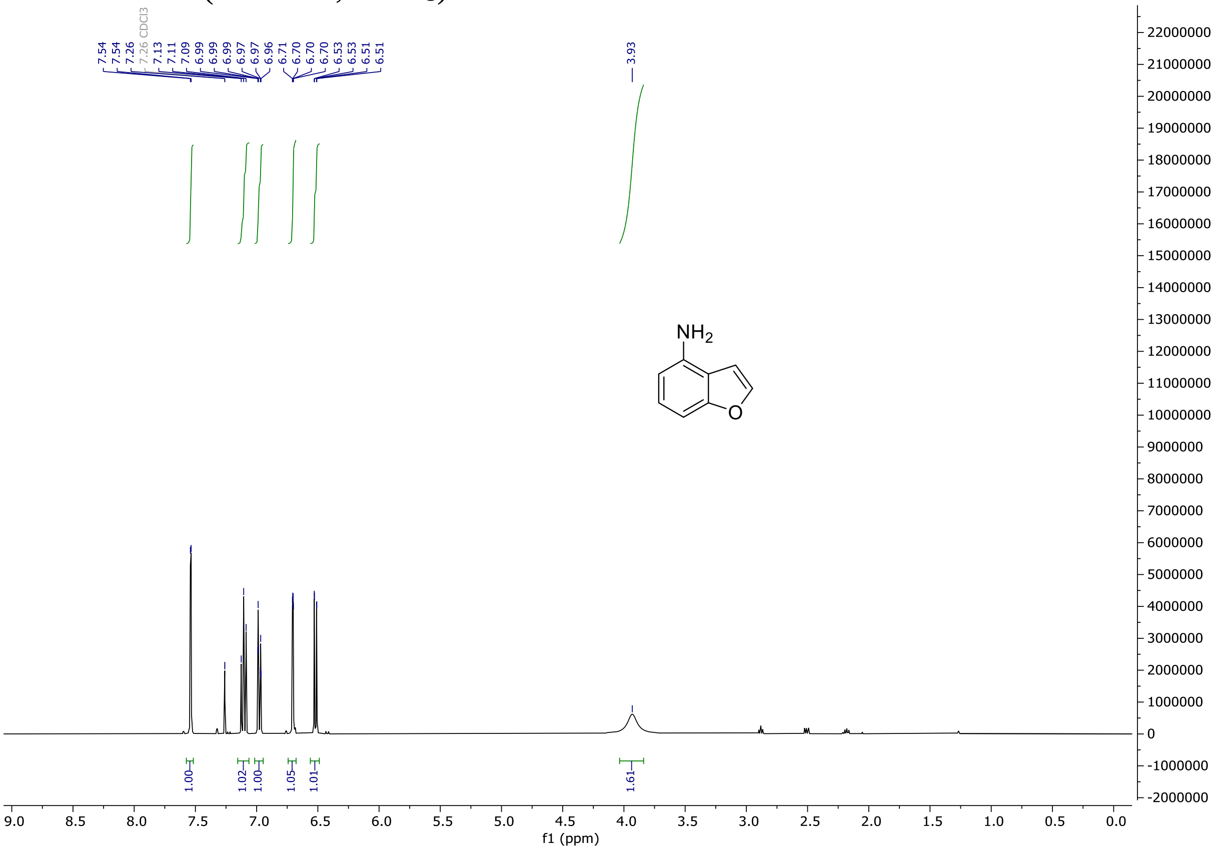

^{13}C{^{1}H} NMR of **1n** (101 MHz, $CDCl_3$)

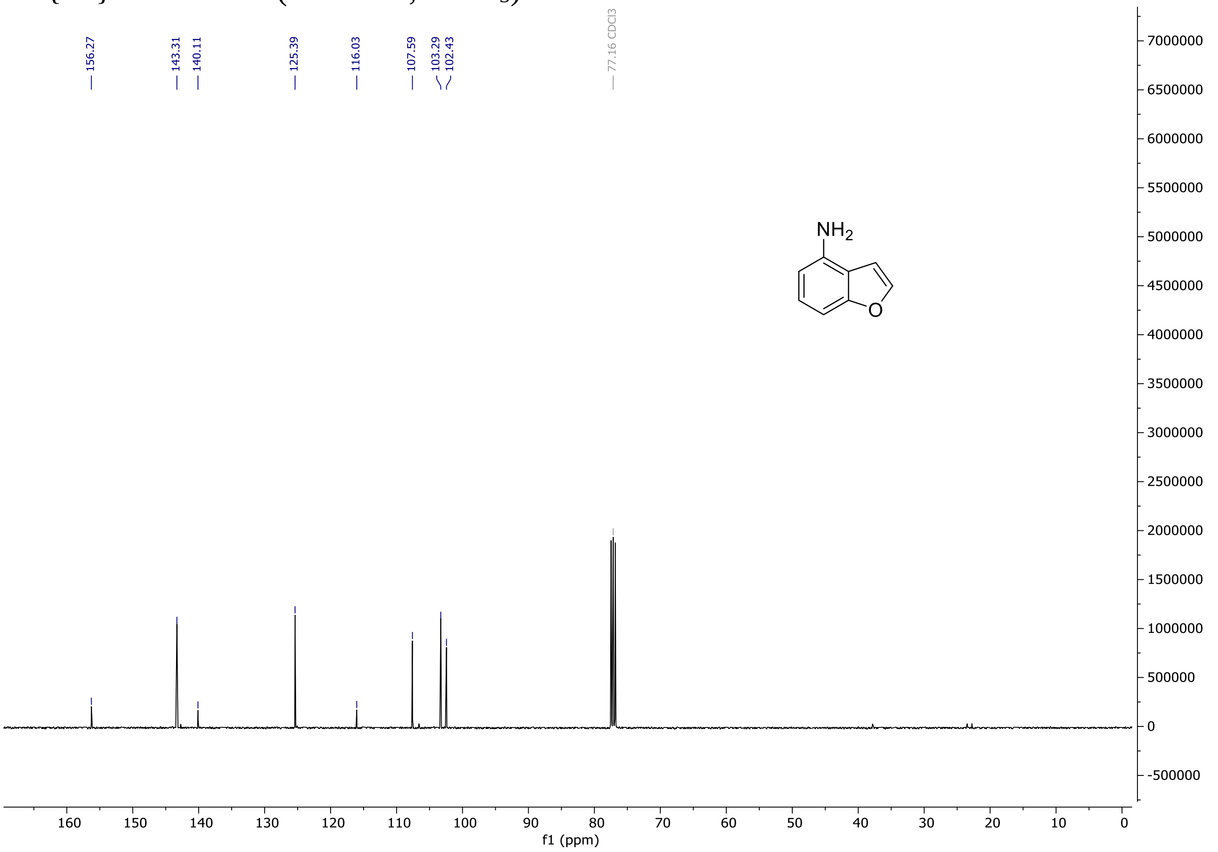

Benzo[*b*]thiophen-4-amine (1o)

^{1}H NMR of **1o** (400 MHz, $CDCl_3$)

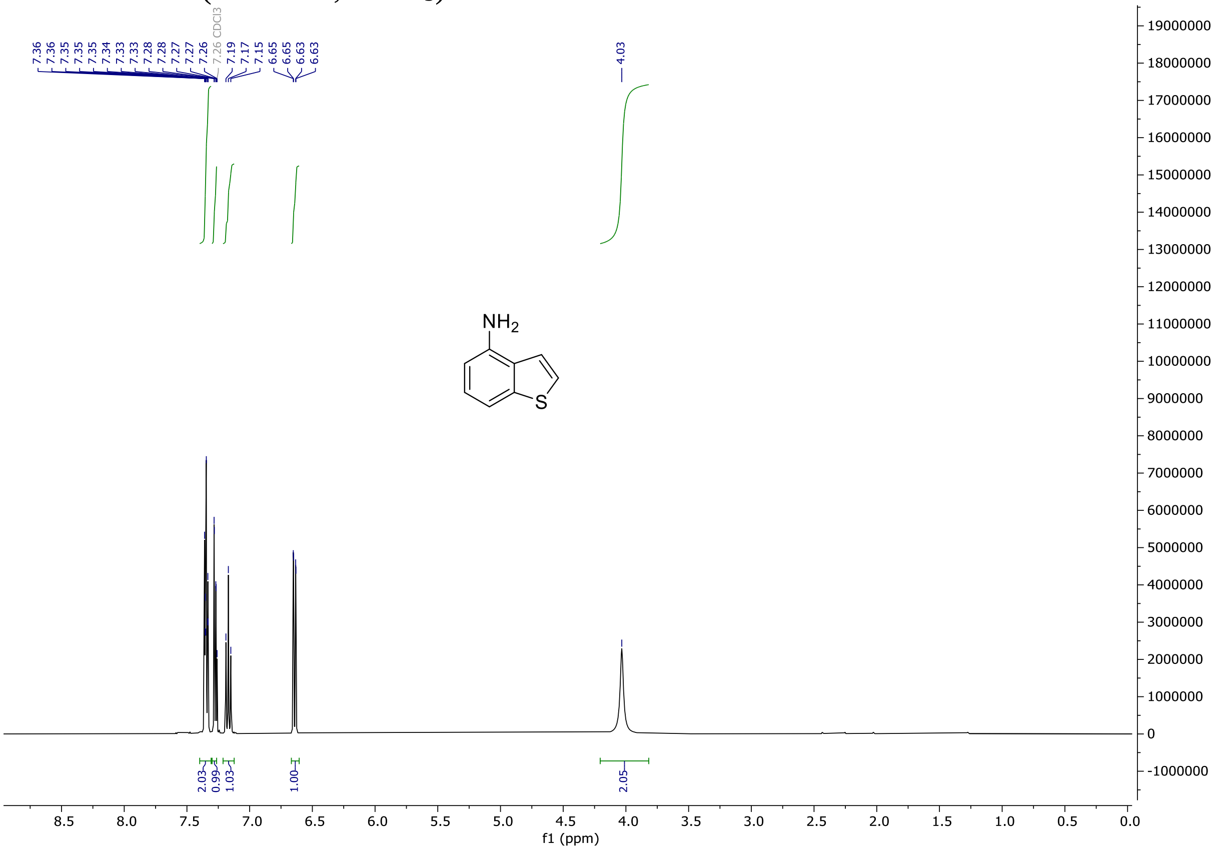

$^{13}C\{^{1}H\}$ NMR of **1o** (101 MHz, $CDCl_3$)

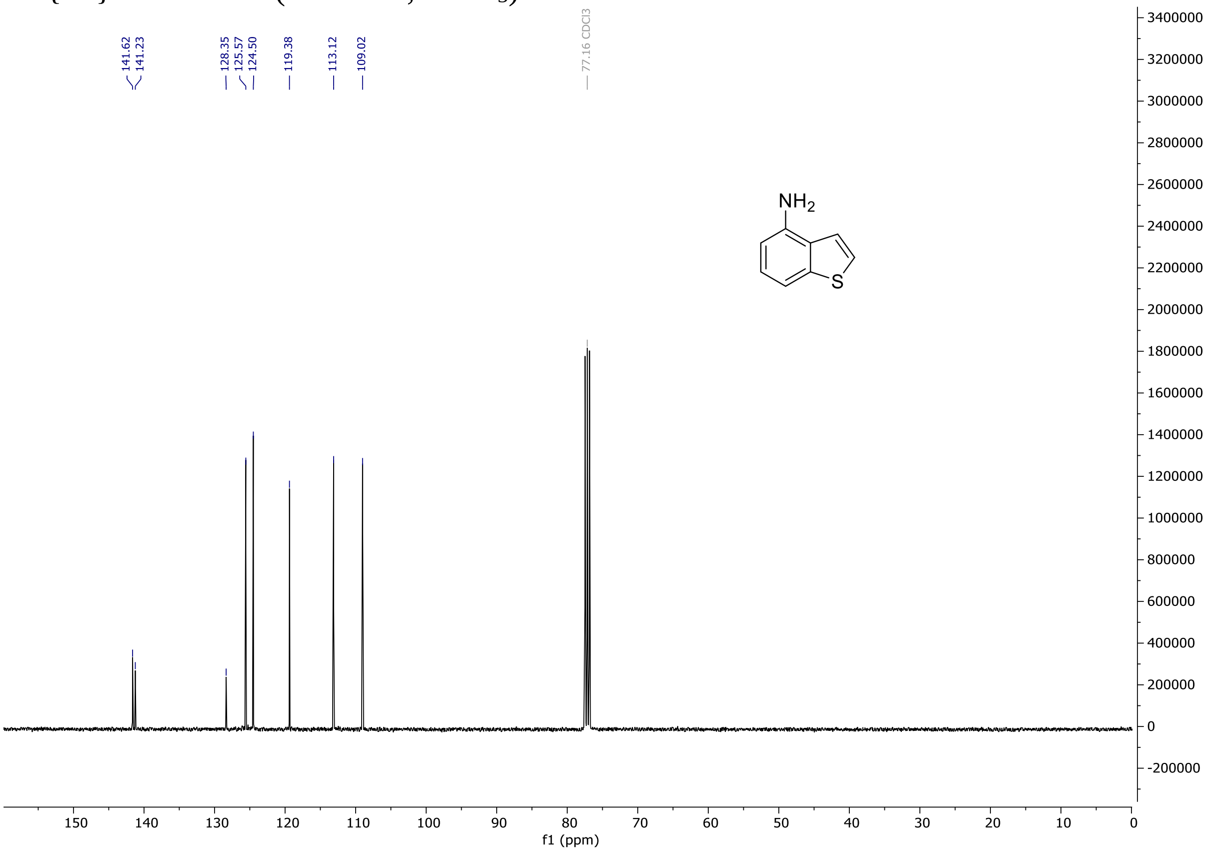

(*S*)-4-Phenyl-1,3,4,7-tetrahydro-2*H*-pyrrolo[2,3-*h*]quinolin-2-one (3a), purity ~ 90%

^{1}H NMR of **3a** (400 MHz, $CDCl_3$)

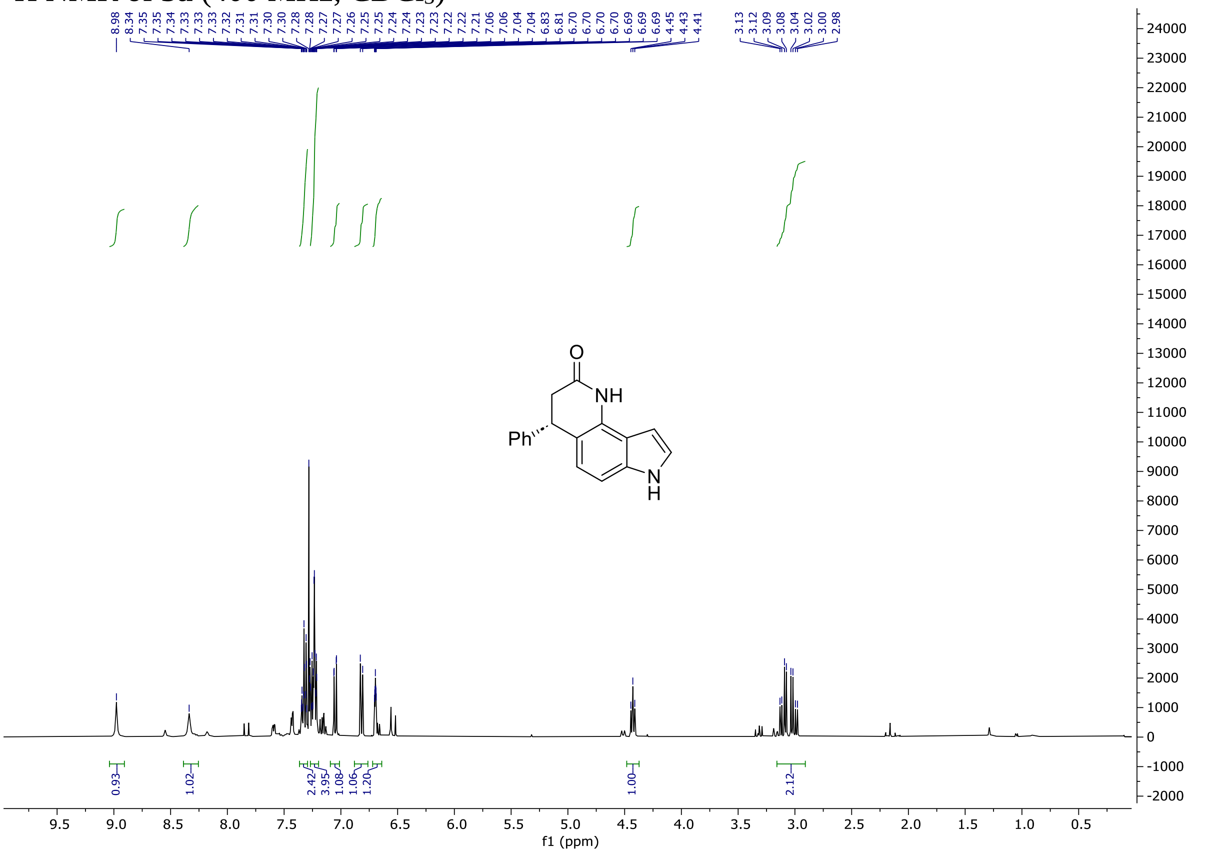

^{13}C{^{1}H} NMR of **3a** (101 MHz, $CDCl_3$)

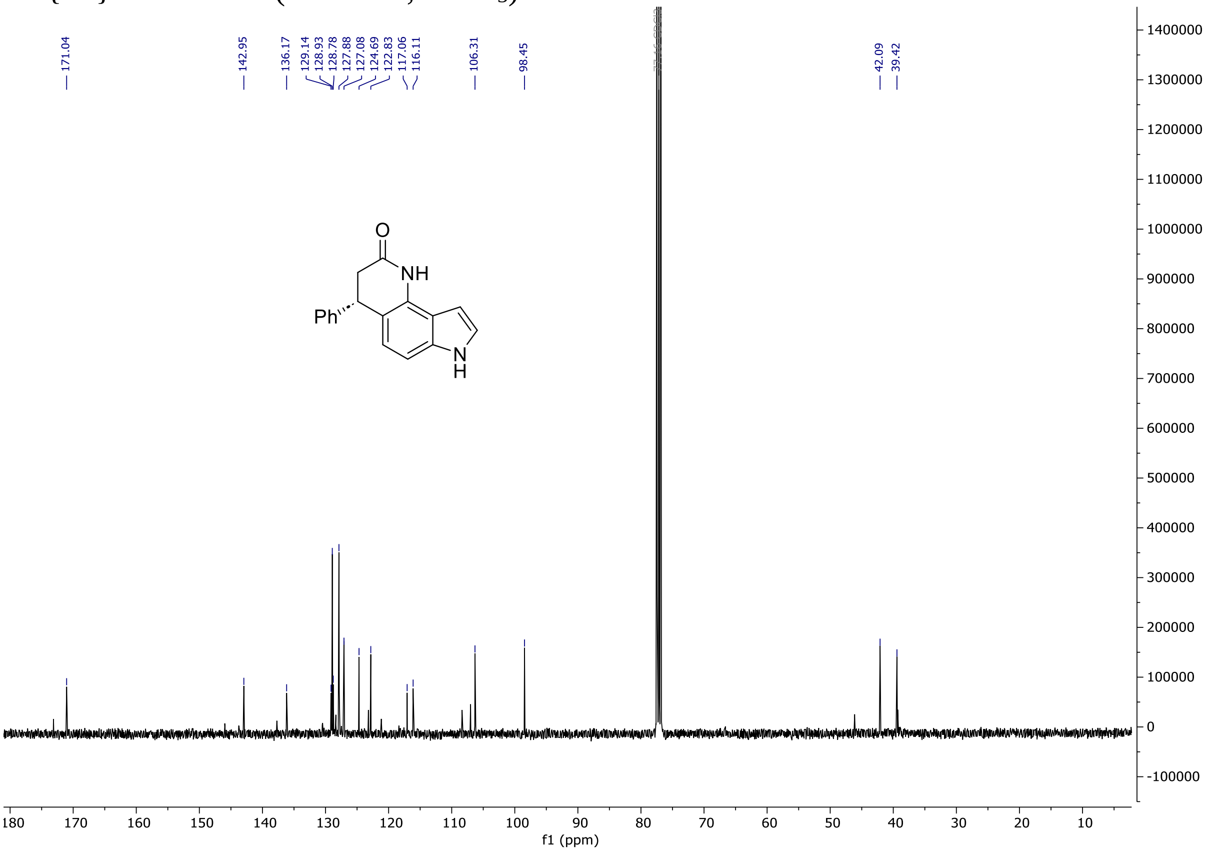

(*S*)-7-Methyl-4-phenyl-1,3,4,7-tetrahydro-2*H*-pyrrolo[2,3-h]quinolin-2-one (3b)

^{1}H NMR of **3b** (400 MHz, $CDCl_3$)

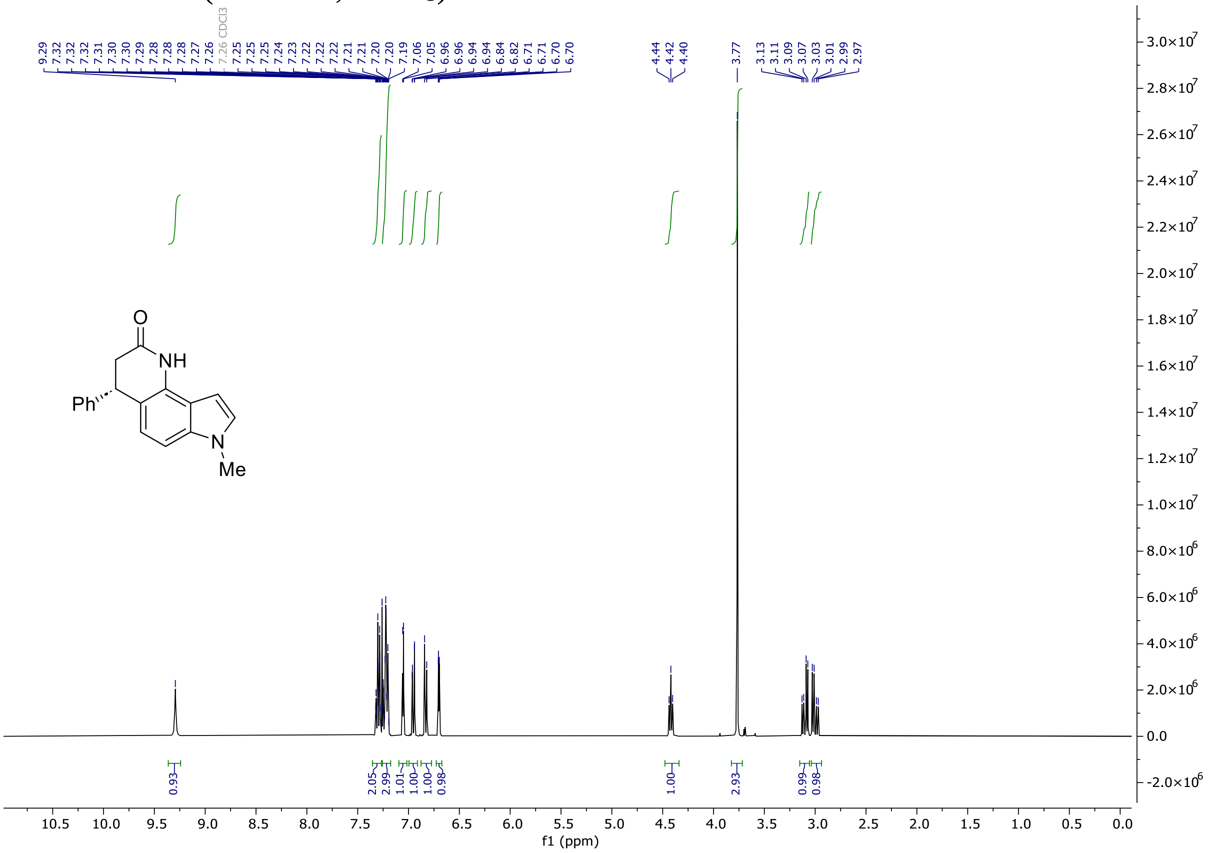

^{13}C{^{1}H} NMR of **3b** (101 MHz, $CDCl_3$)

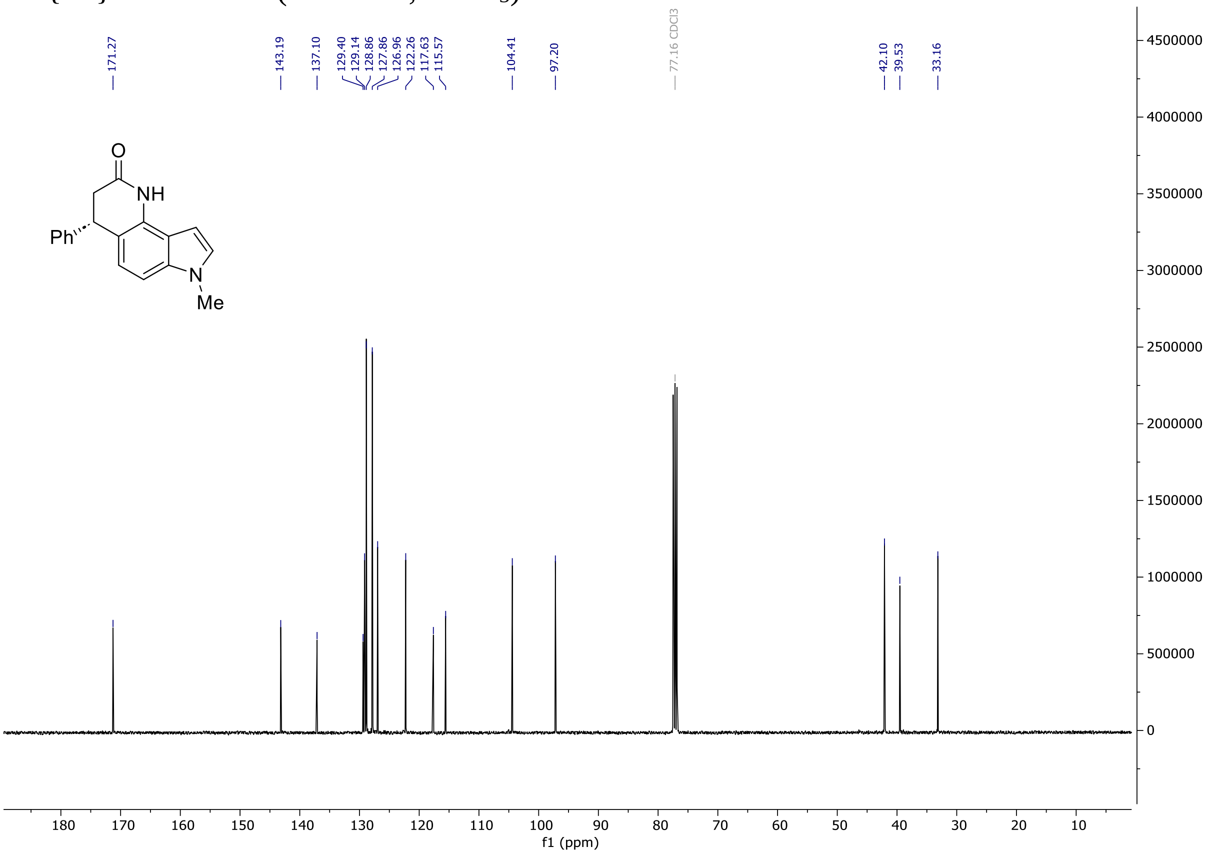

(*S*)-4-Phenyl-7-propyl-1,3,4,7-tetrahydro-2*H*-pyrrolo[2,3-*h*]quinolin-2-oneone (3c)

^{1}H NMR of **3c** (400 MHz, $CDCl_3$)

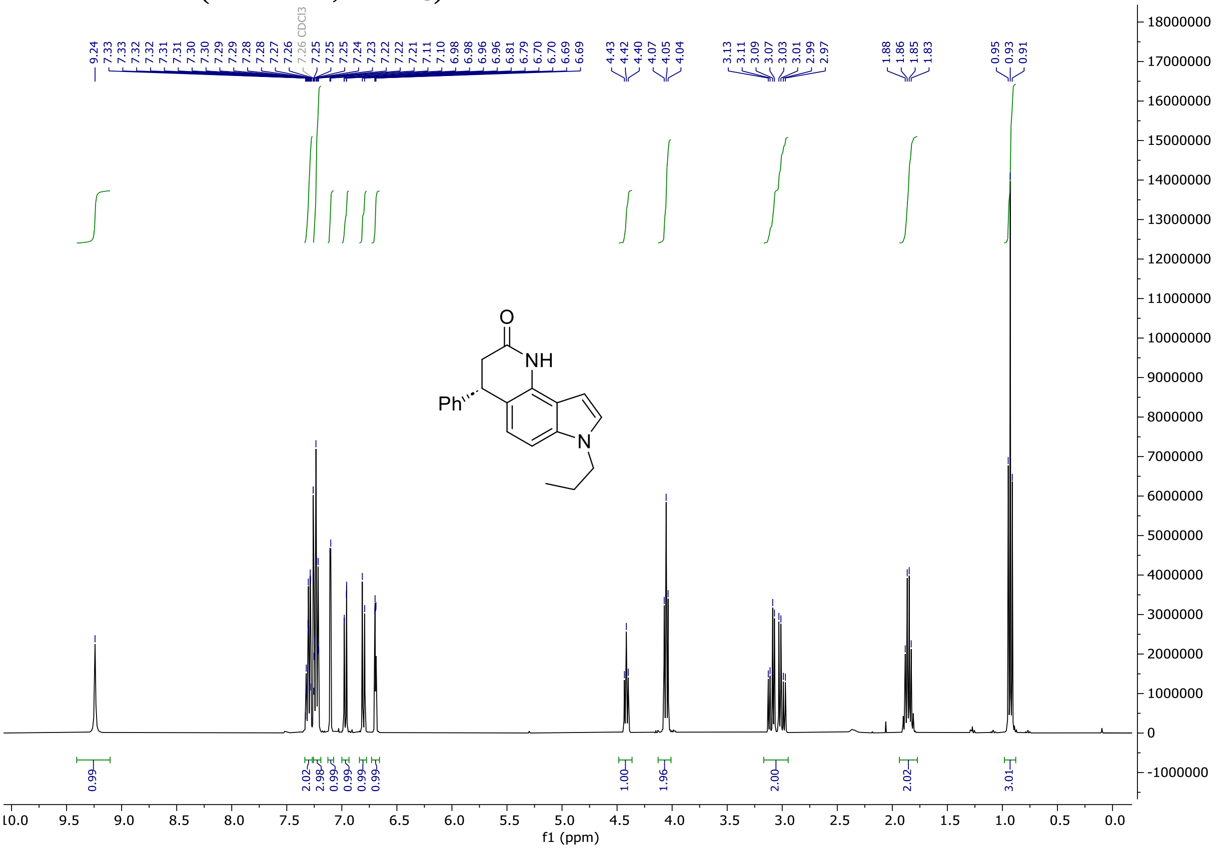

^{13}C{^{1}H} NMR of **3c** (101 MHz, $CDCl_3$)

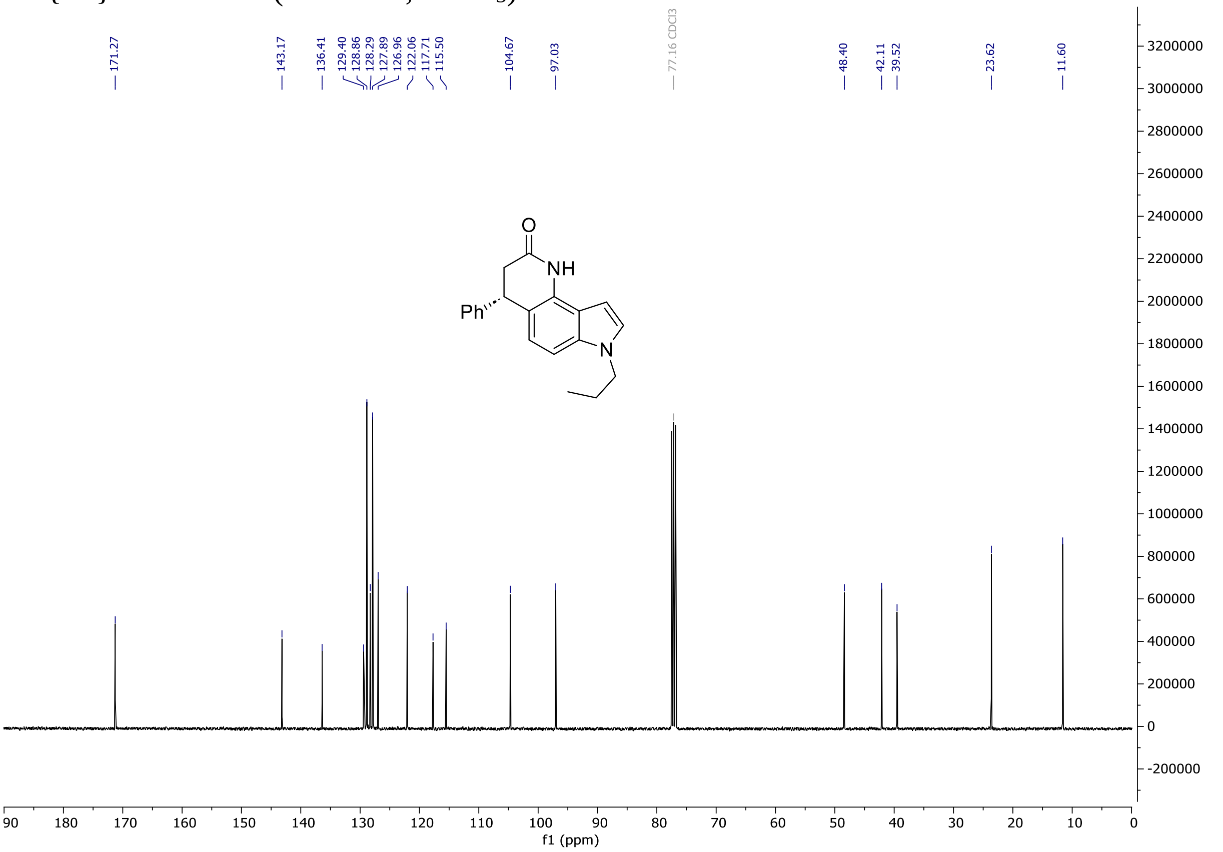

(*S*)-7-Allyl-4-phenyl-1,3,4,7-tetrahydro-2*H*-pyrrolo[2,3-*h*]quinolin-2-one (3d)

^{1}H NMR of **3d** (400 MHz, $CDCl_3$)

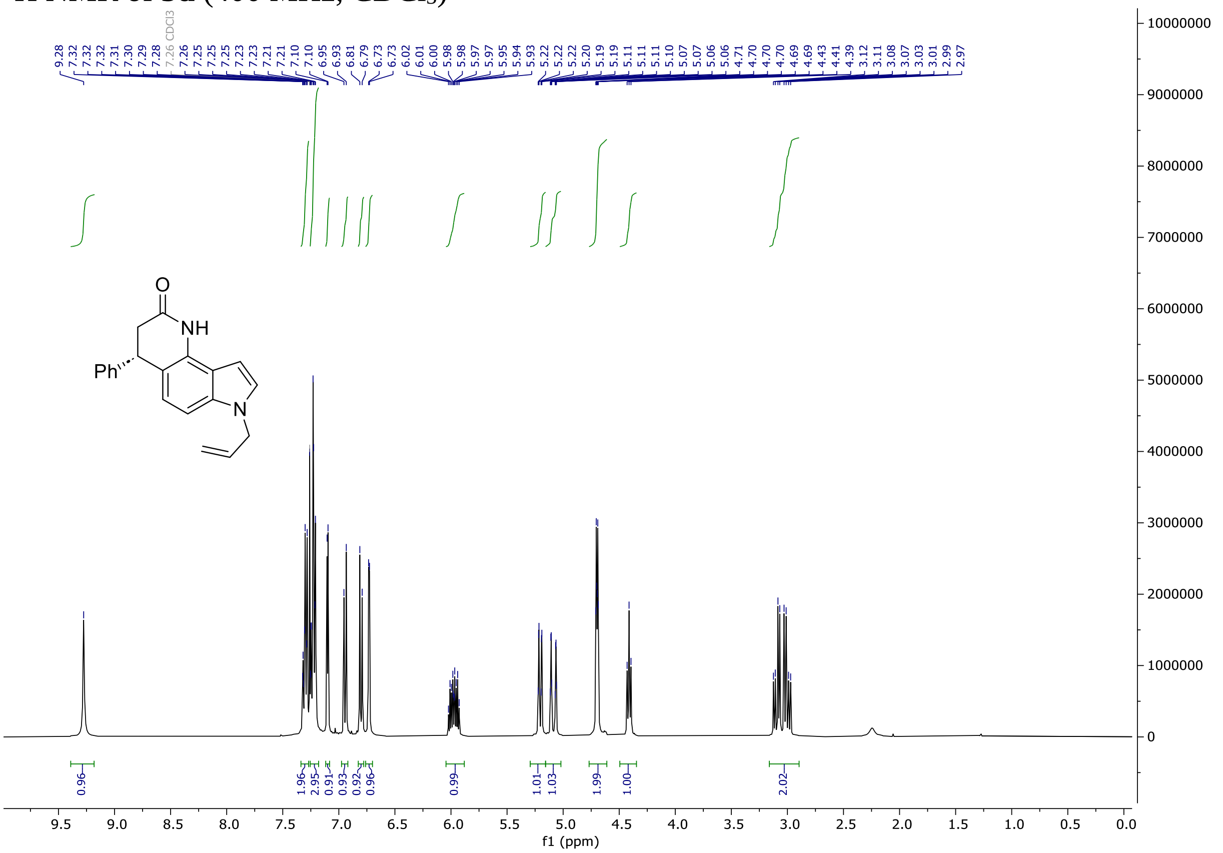

^{13}C{^{1}H} NMR of **3d** (101 MHz, $CDCl_3$)

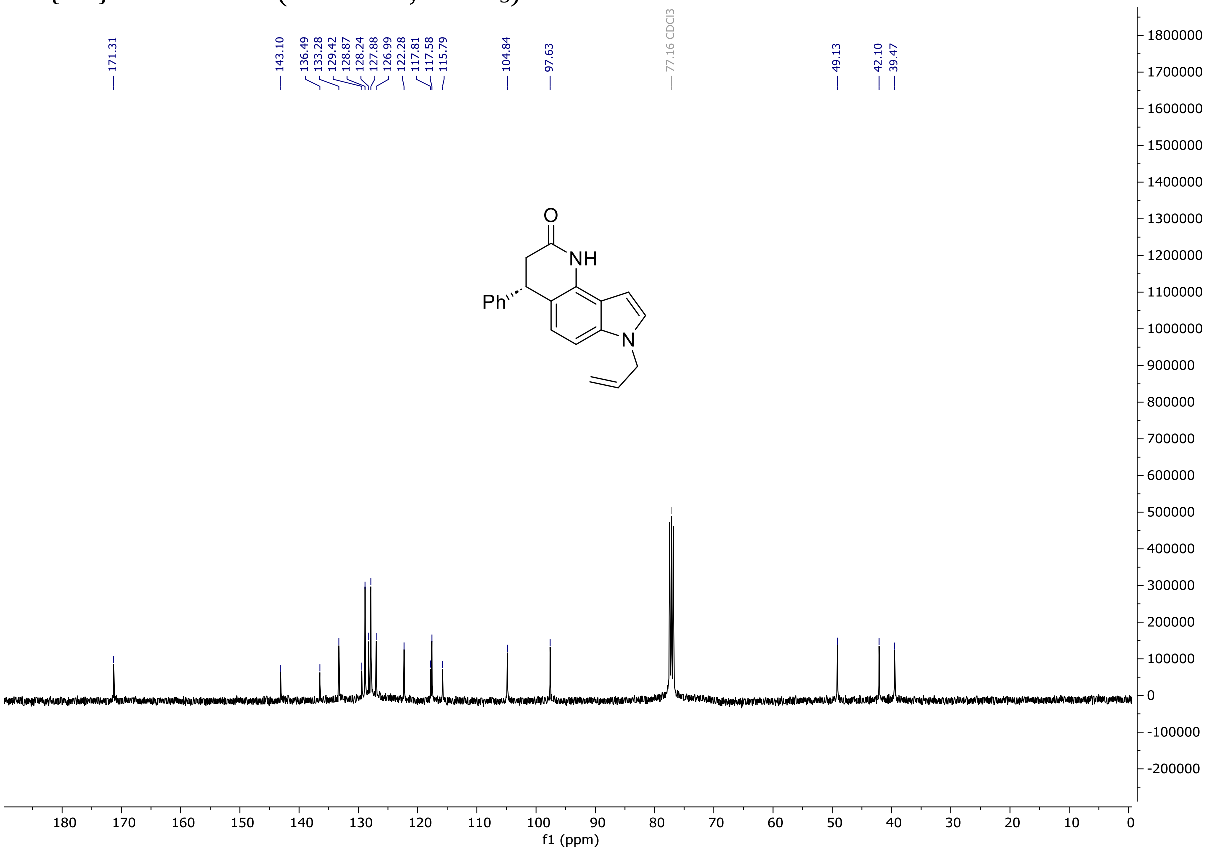

(*S*)-7-Benzyl-4-phenyl-1,3,4,7-tetrahydro-2*H*-pyrrolo[2,3-*h*]quinolin-2-one (3e)

^{1}H NMR of **3e** (400 MHz, $CDCl_3$)

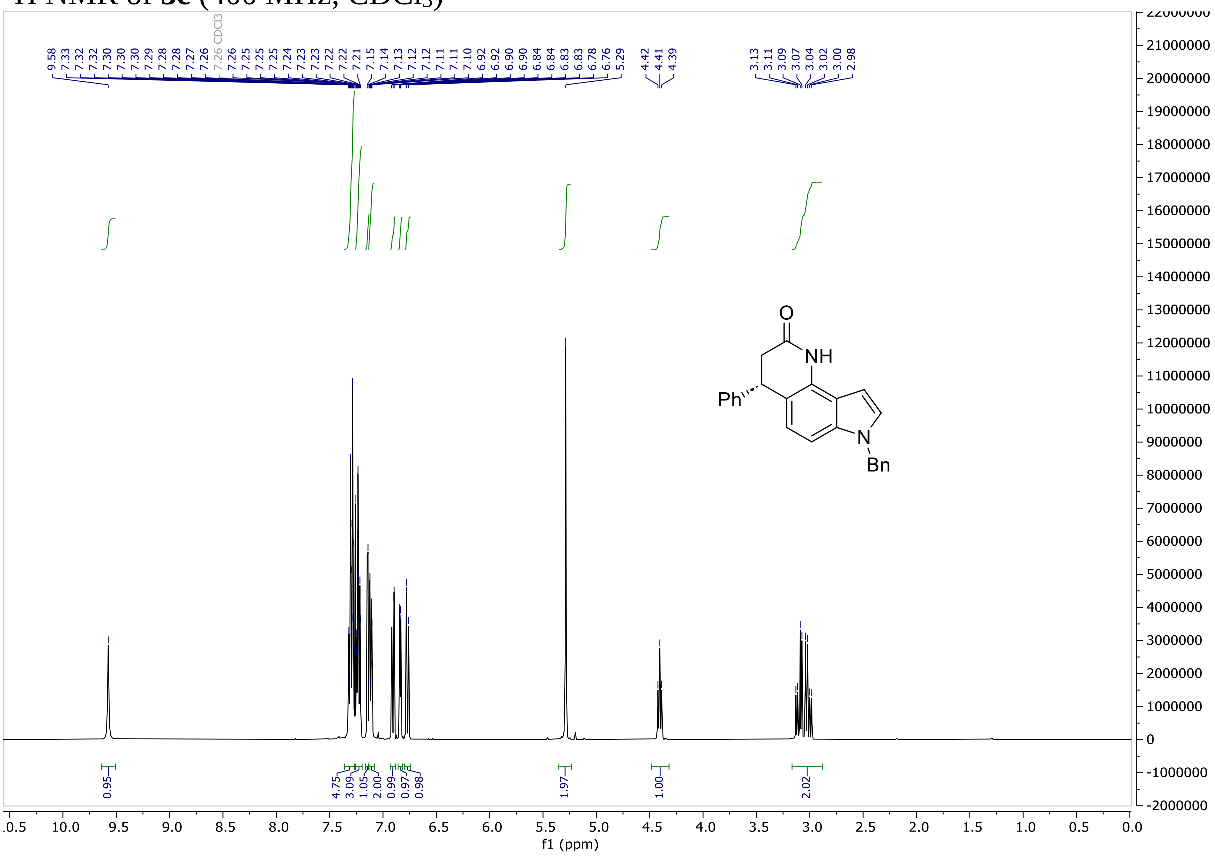

^{13}C{^{1}H} NMR of **3e** (101 MHz, $CDCl_3$)

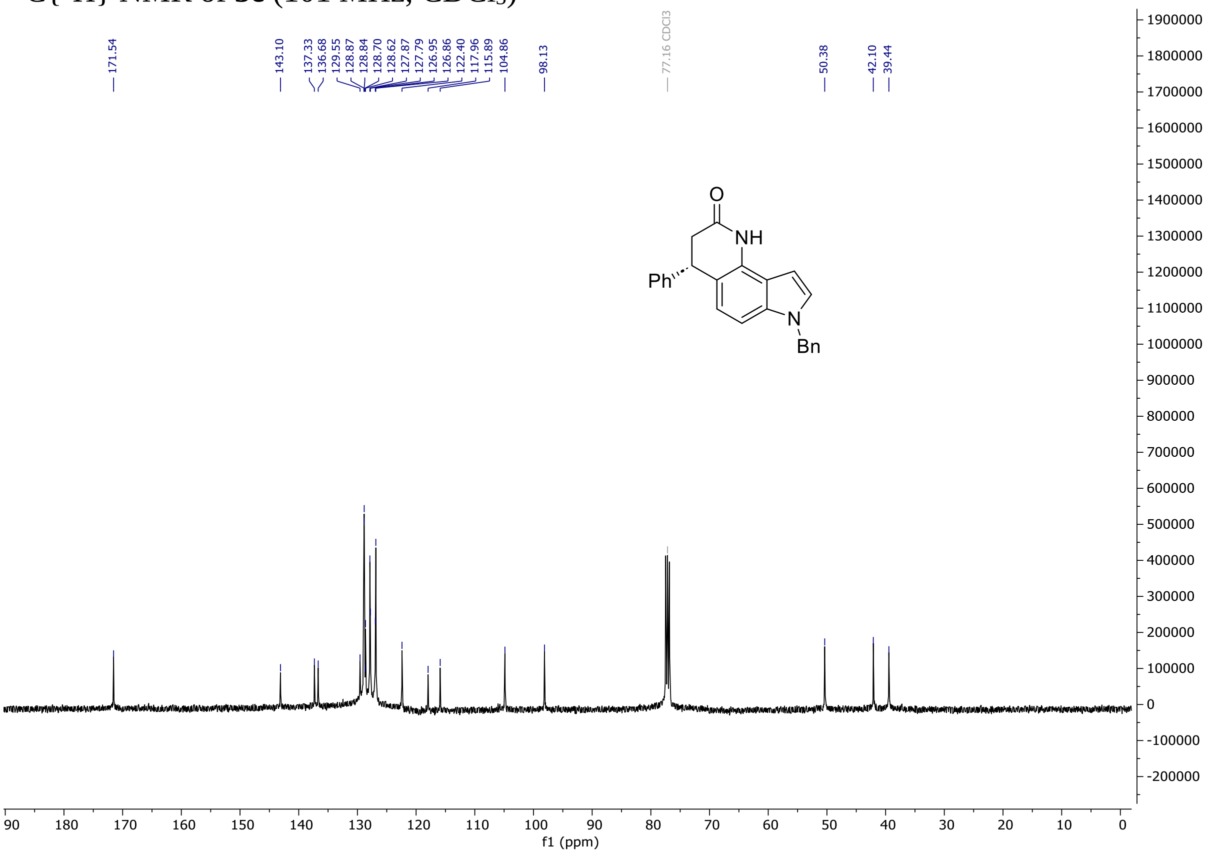

(*S*)-7,9-Dimethyl-4-phenyl-1,3,4,7-tetrahydro-2*H*-pyrrolo[2,3-*h*]quinolin-2-one (3f)

^{1}H NMR of **3f** (400 MHz, $CDCl_3$)

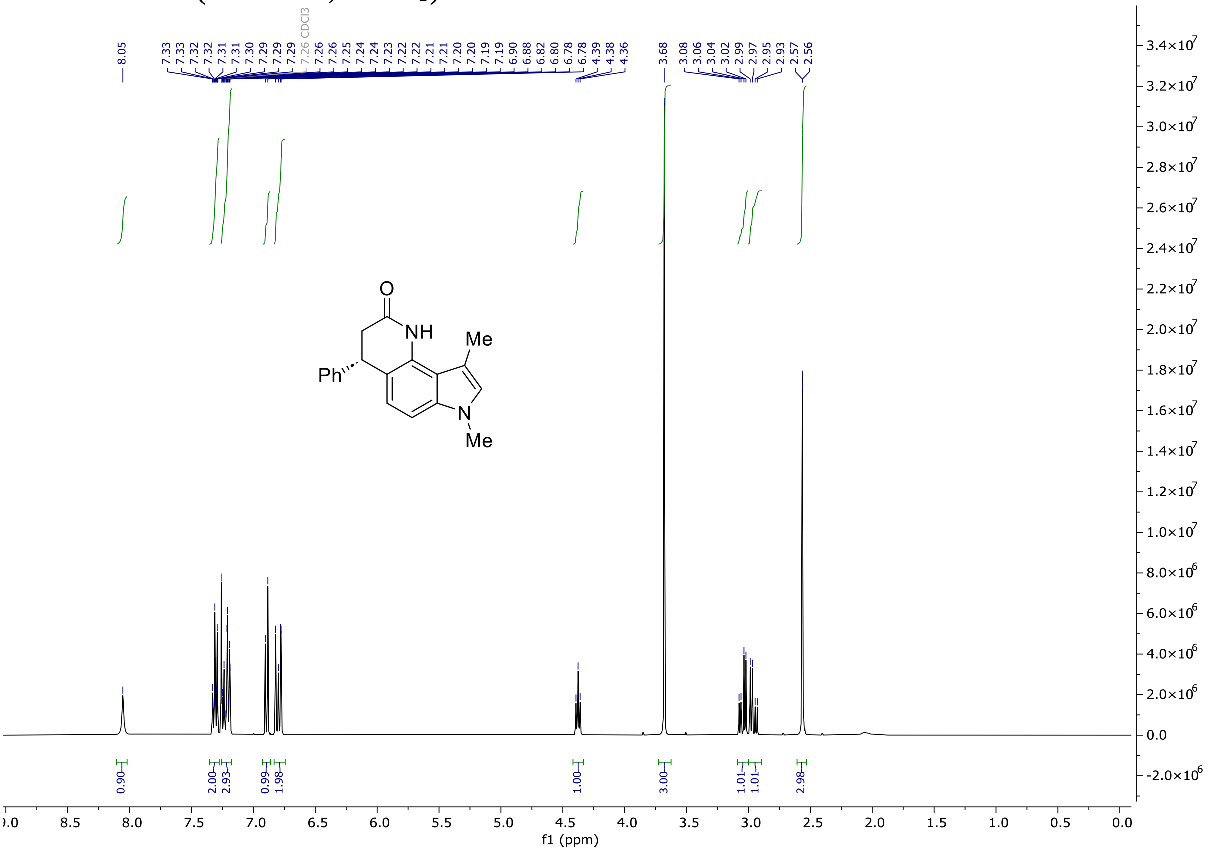

^{13}C{^{1}H} NMR of **3f** (101 MHz, $CDCl_3$)

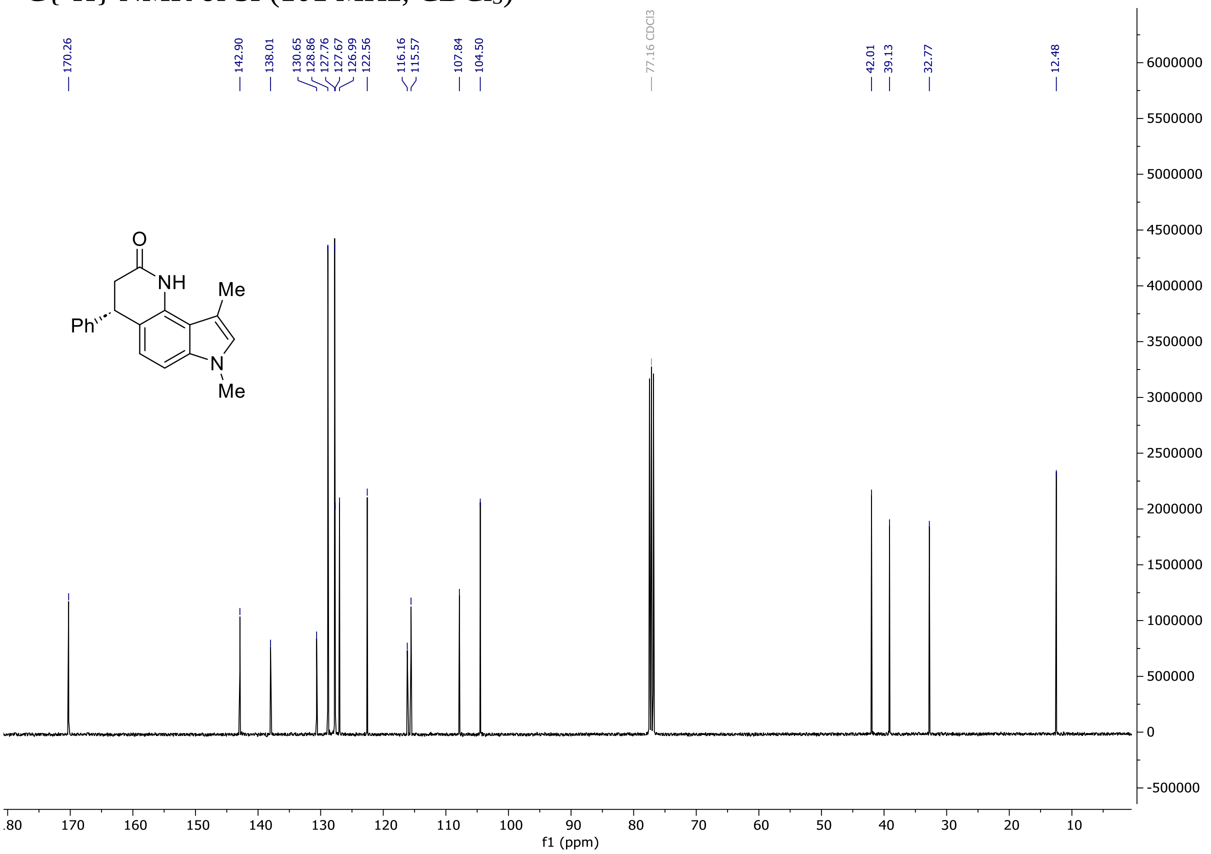

(*S*)-7-Methyl-4-(naphthalen-2-yl)-1,3,4,7-tetrahydro-2*H*-pyrrolo[2,3-*h*]quinolin-2-one (3g)

^{1}H NMR of **3g** (400 MHz, $CDCl_3$)

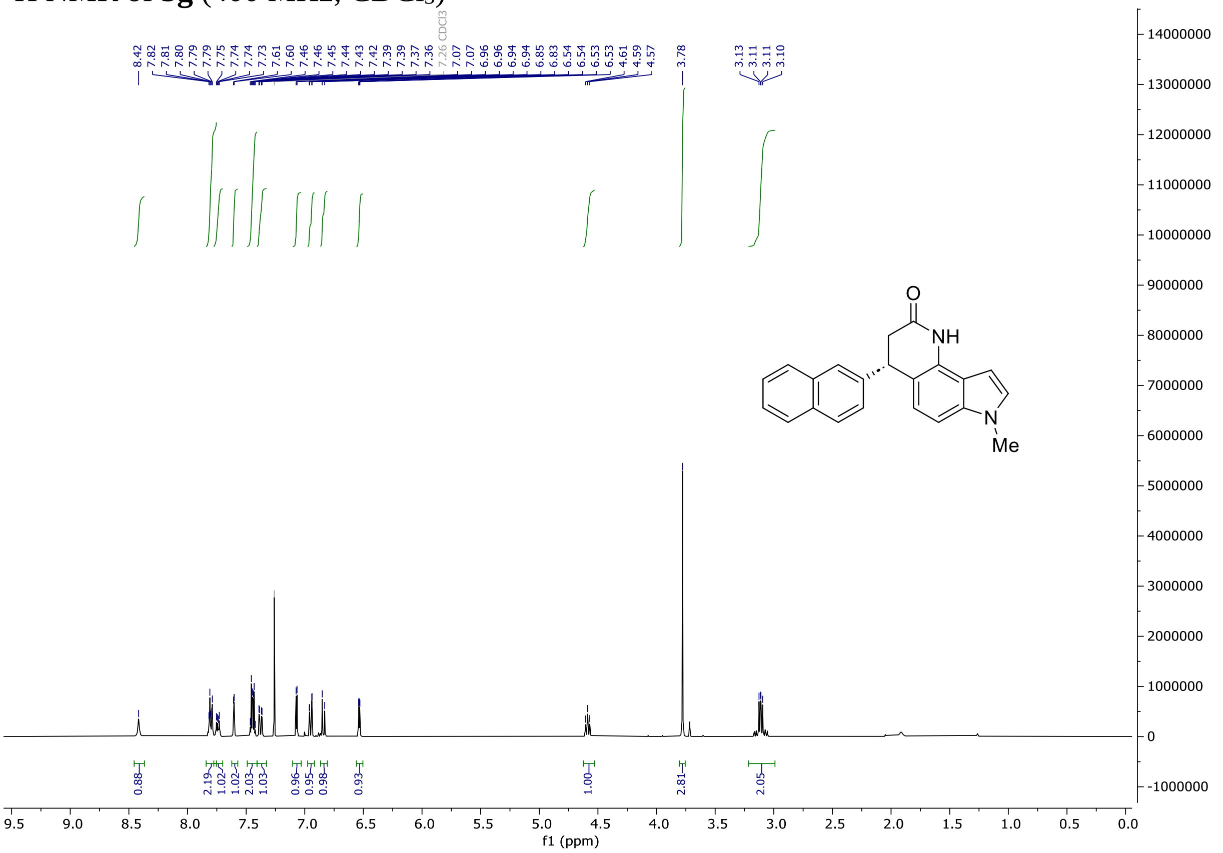

^{13}C{^{1}H} NMR of **3g** (101 MHz, $CDCl_3$)

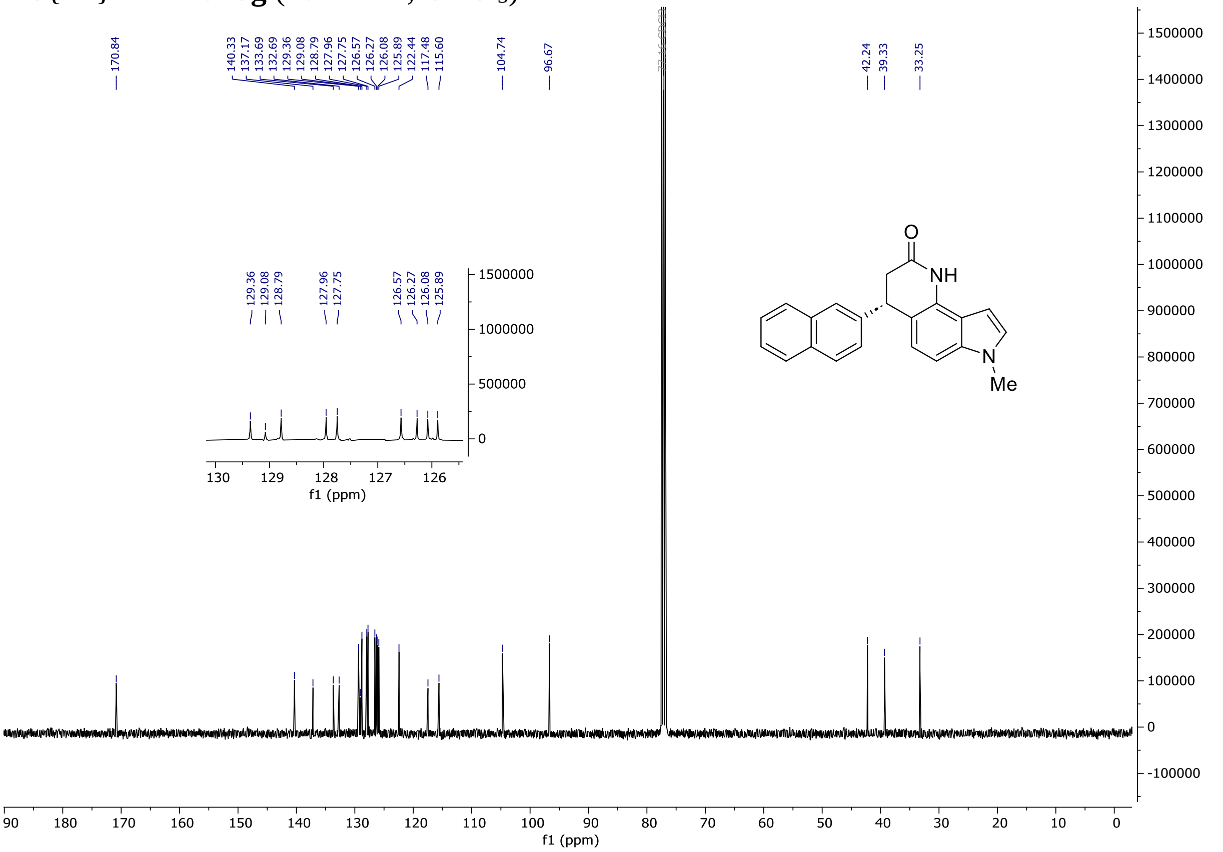

(*S*)-7-Methyl-4-(*p*-tolyl)-1,3,4,7-tetrahydro-2*H*-pyrrolo[2,3-*h*]quinolin-2-one (3h)

^{1}H NMR of **3h** (400 MHz, $CDCl_3$)

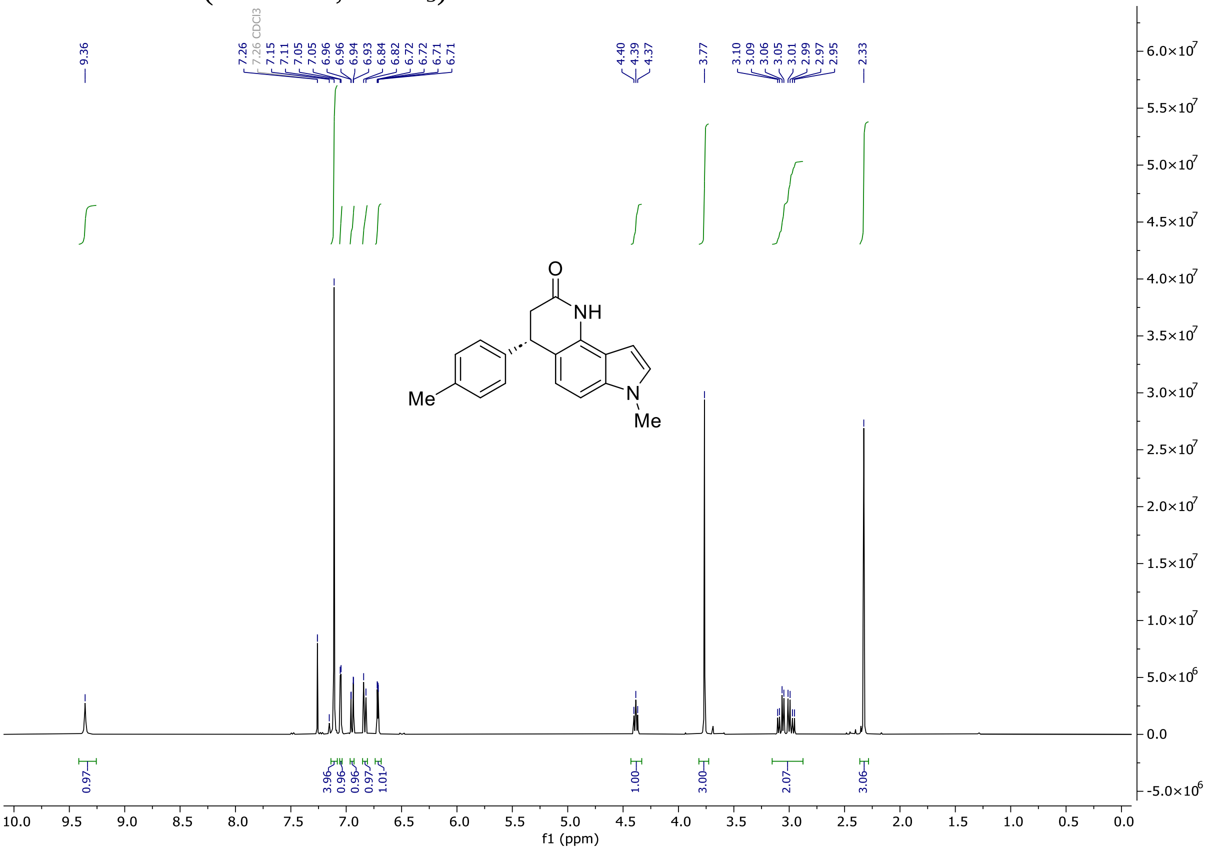

^{13}C{^{1}H} NMR of **3h** (101 MHz, $CDCl_3$)

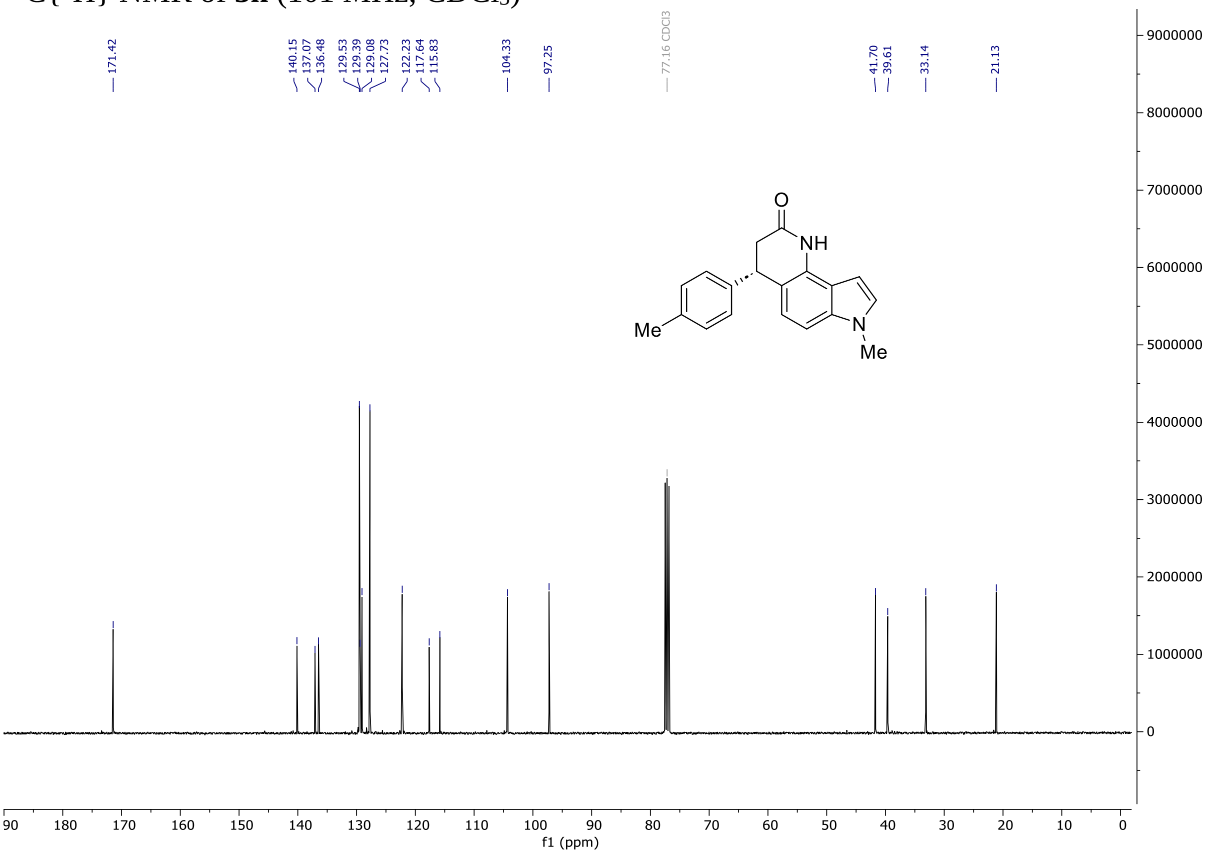

(*S*)-4-(4-Methoxyphenyl)-7-methyl-1,3,4,7-tetrahydro-2*H*-pyrrolo[2,3-*h*]quinolin-2-one (3i)

^{1}H NMR of **3h** (400 MHz, $CDCl_3$)

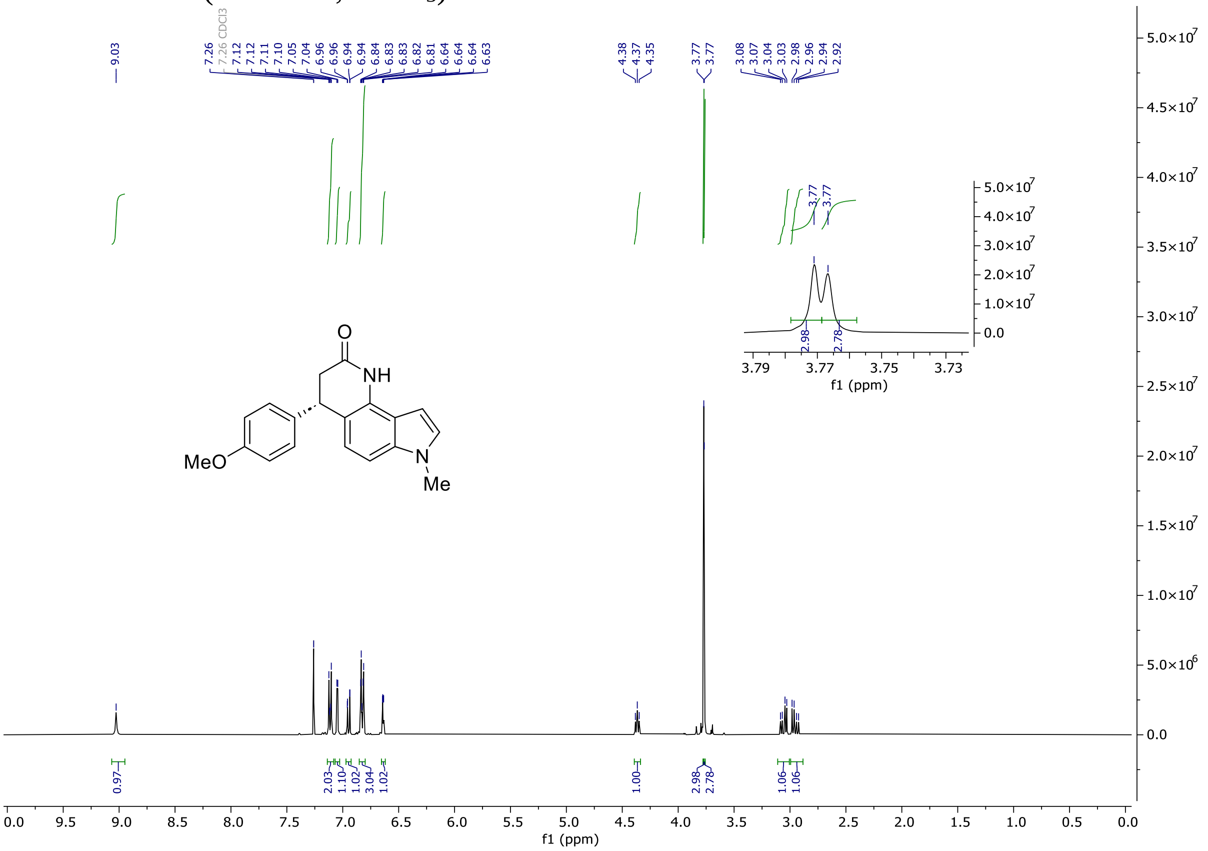

^{13}C{^{1}H} NMR of **3i** (101 MHz, $CDCl_3$)

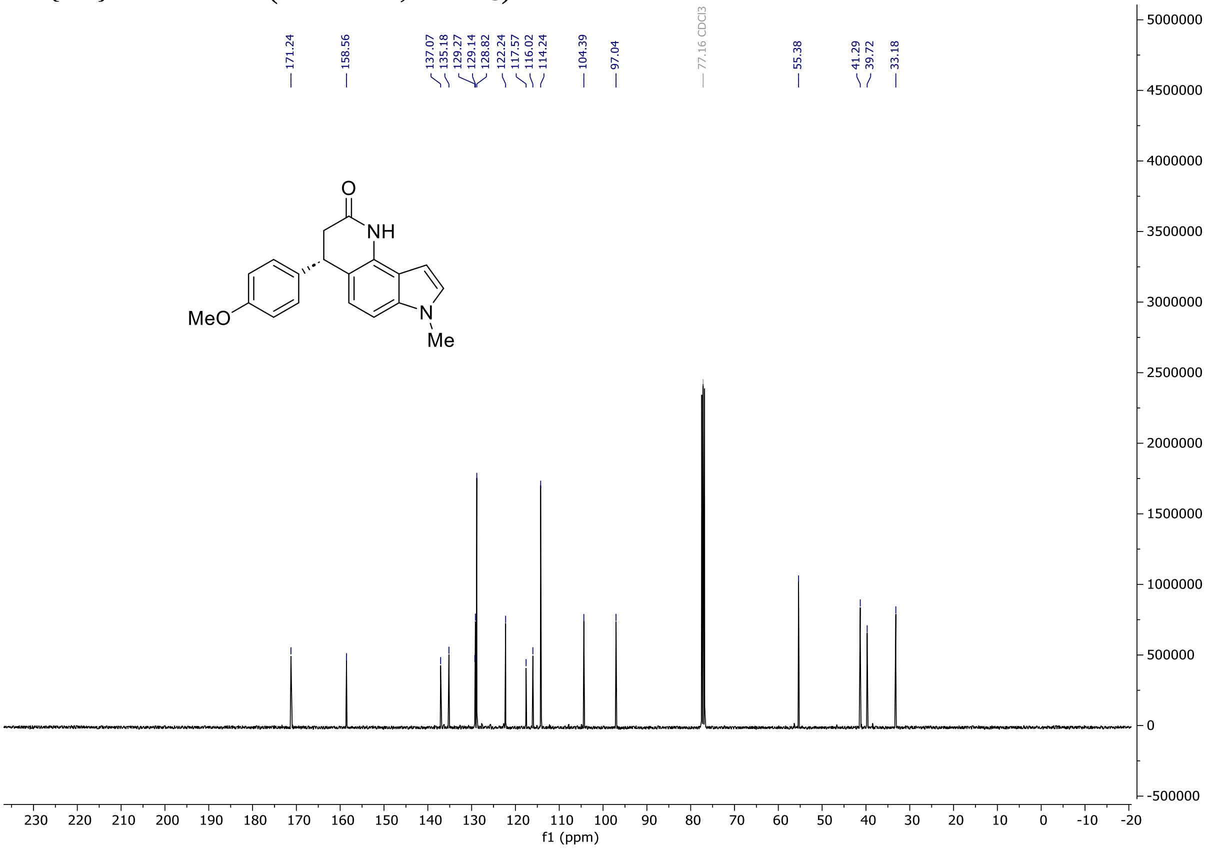

(*S*)-7-Methyl-4-(4-nitrophenyl)-1,3,4,7-tetrahydro-2*H*-pyrrolo[2,3-*h*]quinolin-2-one (3j)

^{1}H NMR of **3j** (400 MHz, $CDCl_3$)

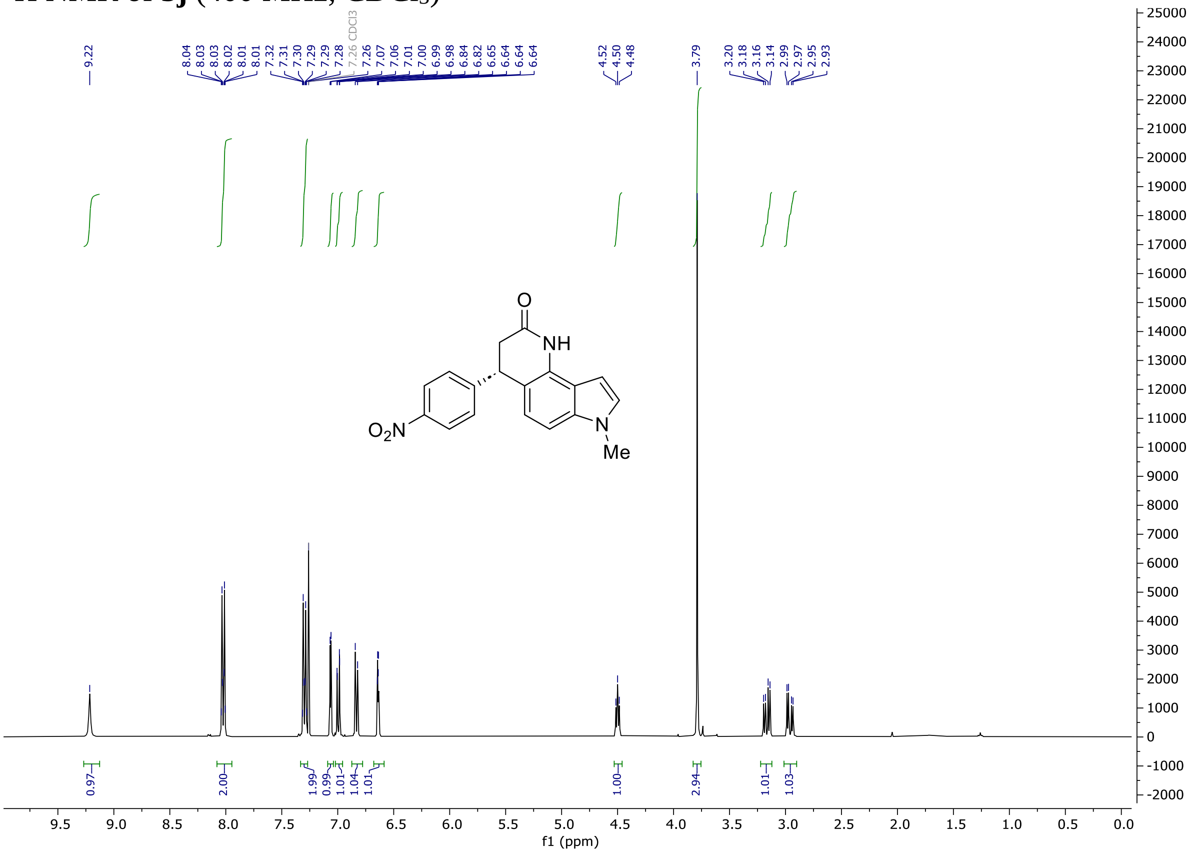

^{13}C{^{1}H} NMR of **3j** (101 MHz, $CDCl_3$)

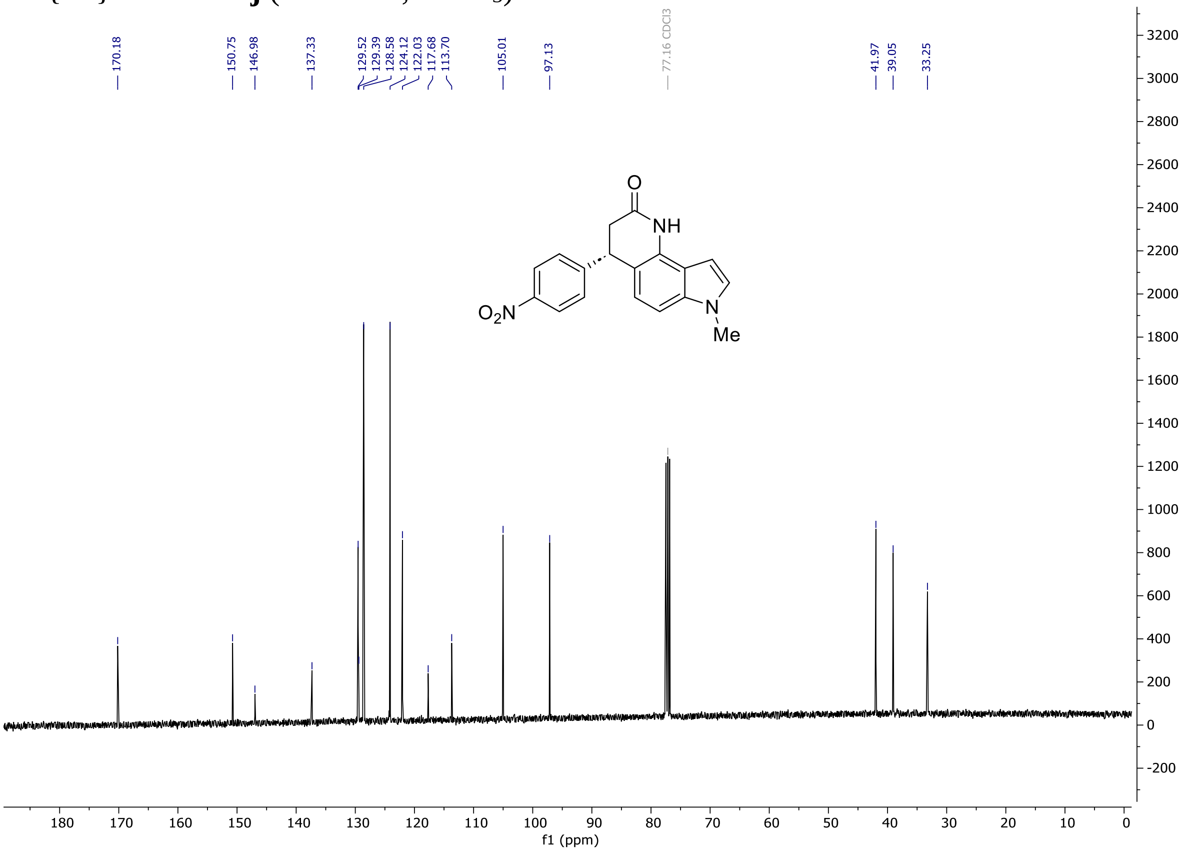

(*S*)-4-(7-Methyl-2-oxo-2,3,4,7-tetrahydro-1*H*-pyrrolo[2,3-*h*]quinolin-4-yl)benzonitrile (3k)

^{1}H NMR of **3k** (400 MHz, $CDCl_3$)

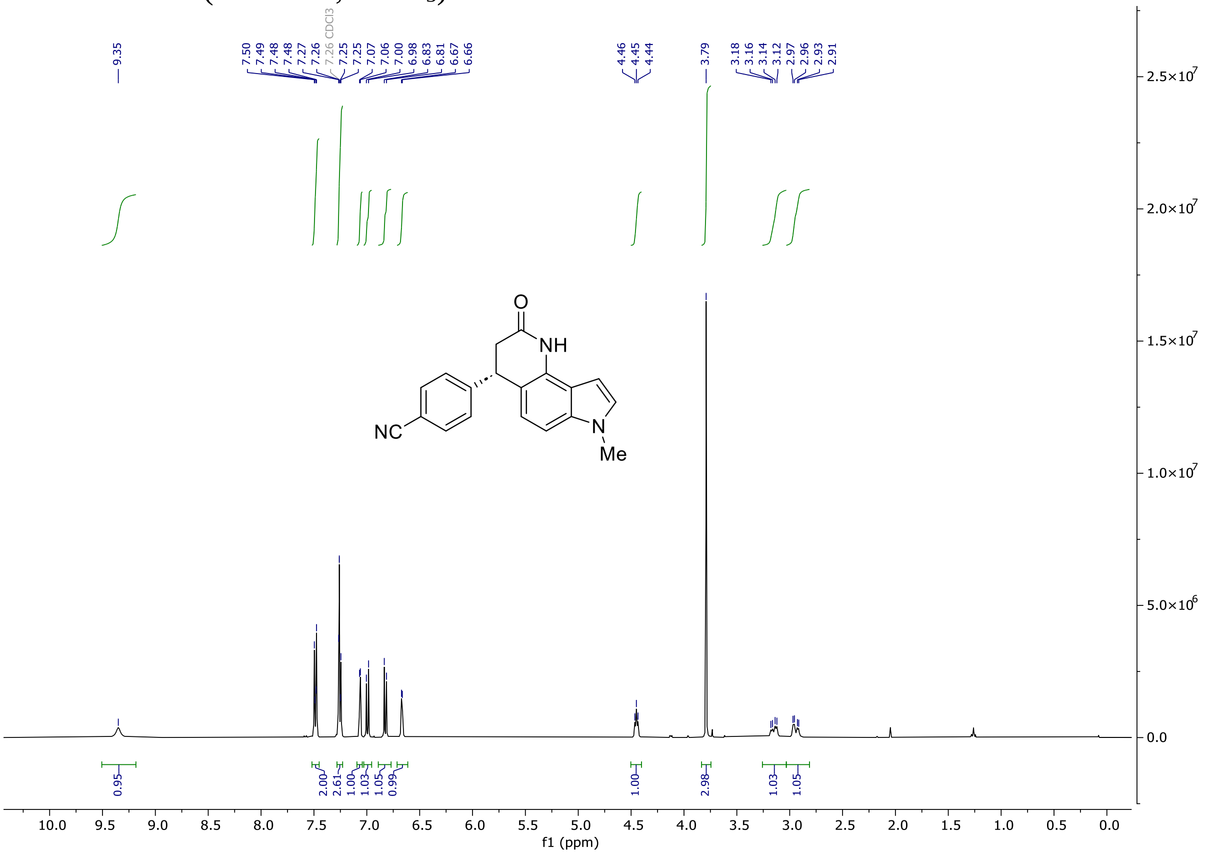

^{13}C{^{1}H} NMR of **3k** (101 MHz, $CDCl_3$)

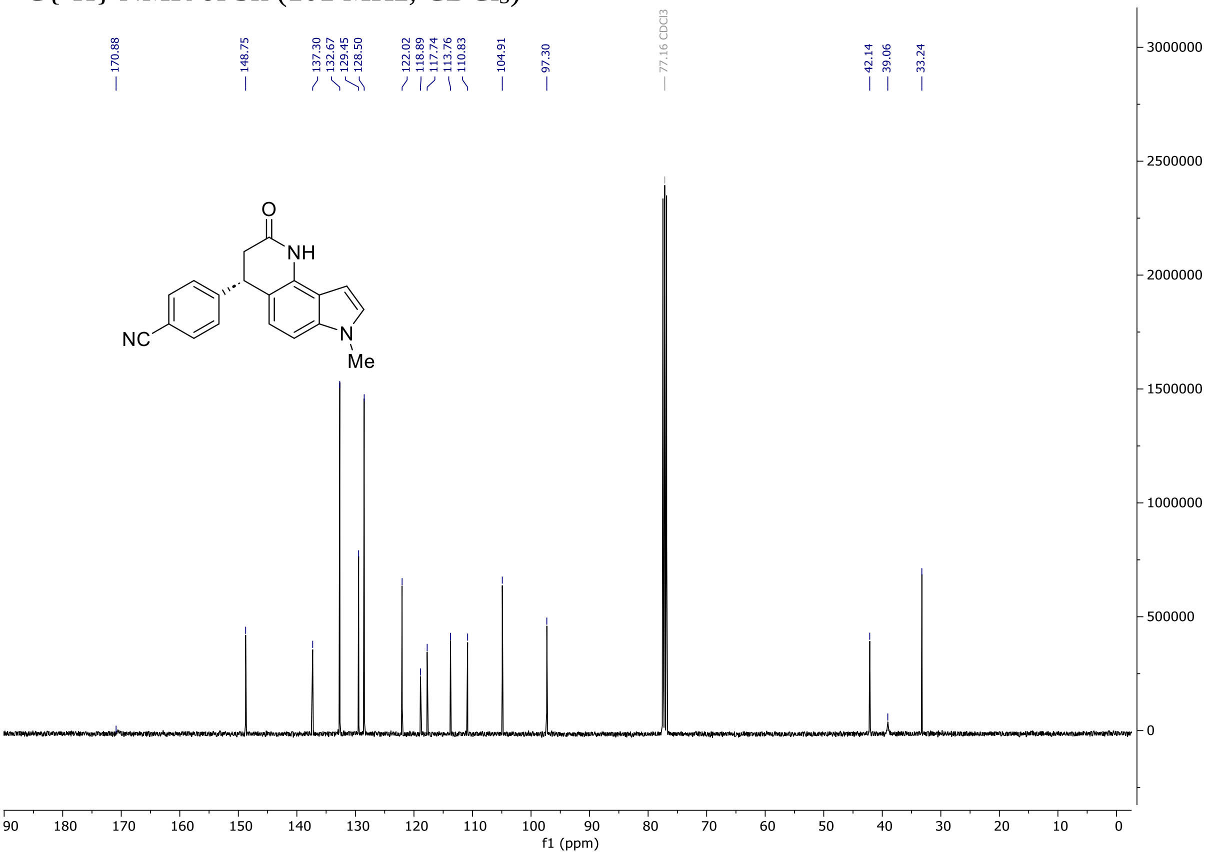

(*S*)-7-Methyl-4-(4-(trifluoromethyl)phenyl)-1,3,4,7-tetrahydro-2*H*-pyrrolo[2,3-*h*]quinolin-2-one (3l)

^{1}H NMR of **3l** (400 MHz, $CDCl_3$)

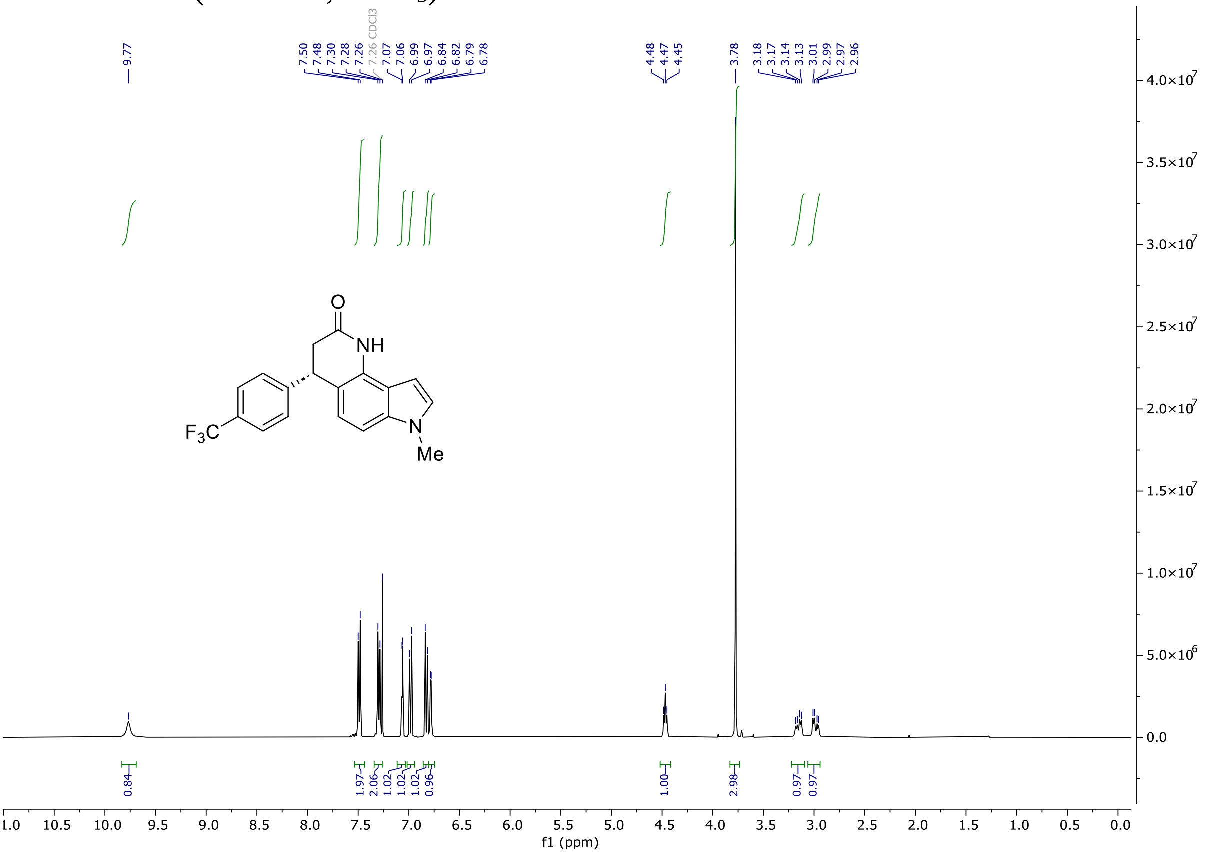

^{13}C{^{1}H} NMR of **3l** (101 MHz, $CDCl_3$)

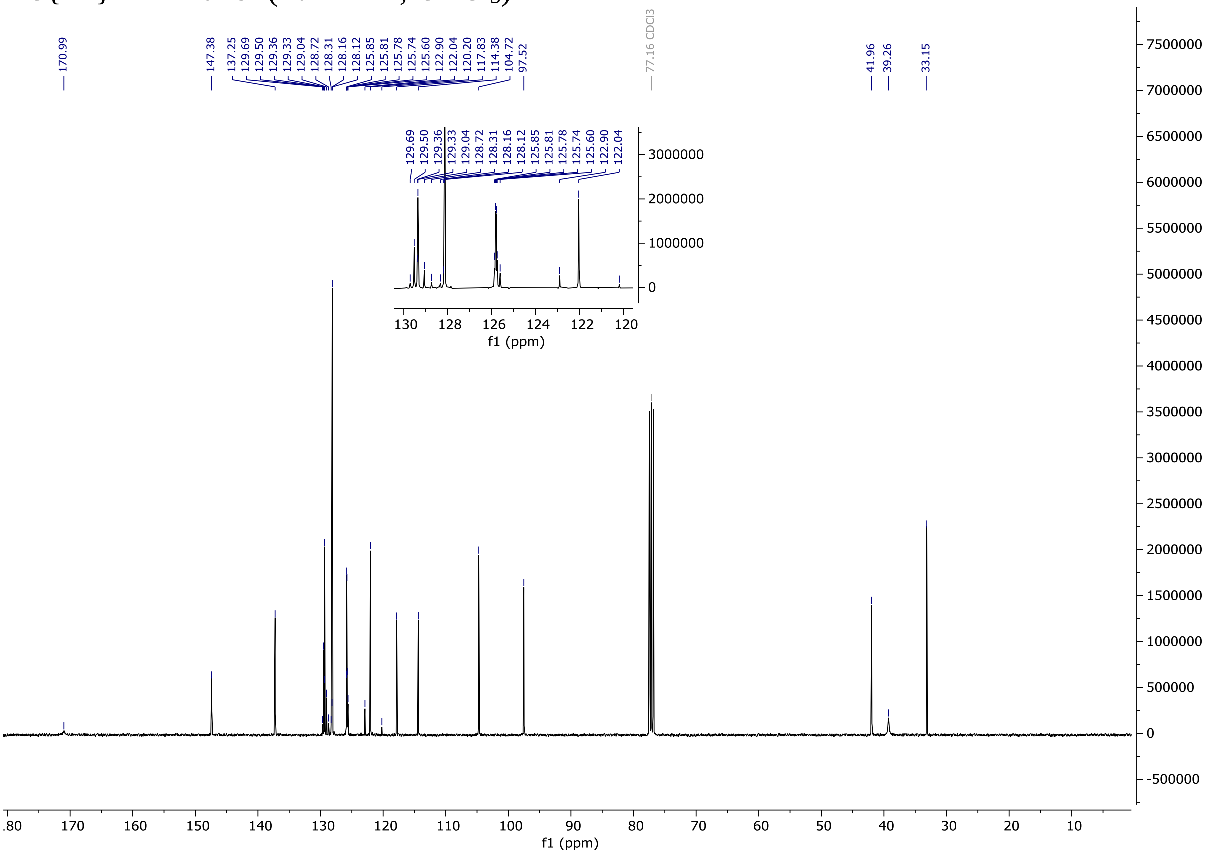

^{19}F NMR of **3l** (376 MHz, $CDCl_3$)

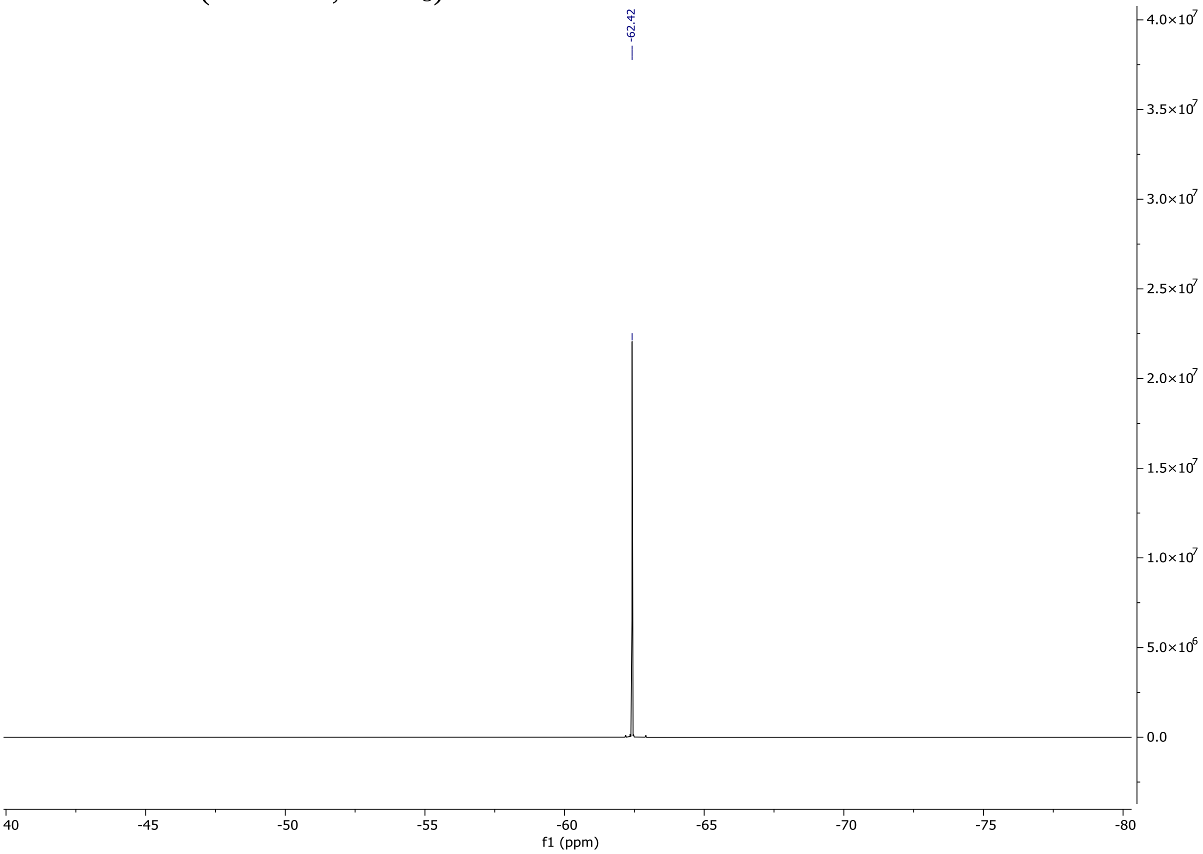

(*S*)-4-(4-Fluorophenyl)-7-methyl-1,3,4,7-tetrahydro-2*H*-pyrrolo[2,3-*h*]quinolin-2-one (3l)

^{1}H NMR of **3m** (400 MHz, $CDCl_3$)

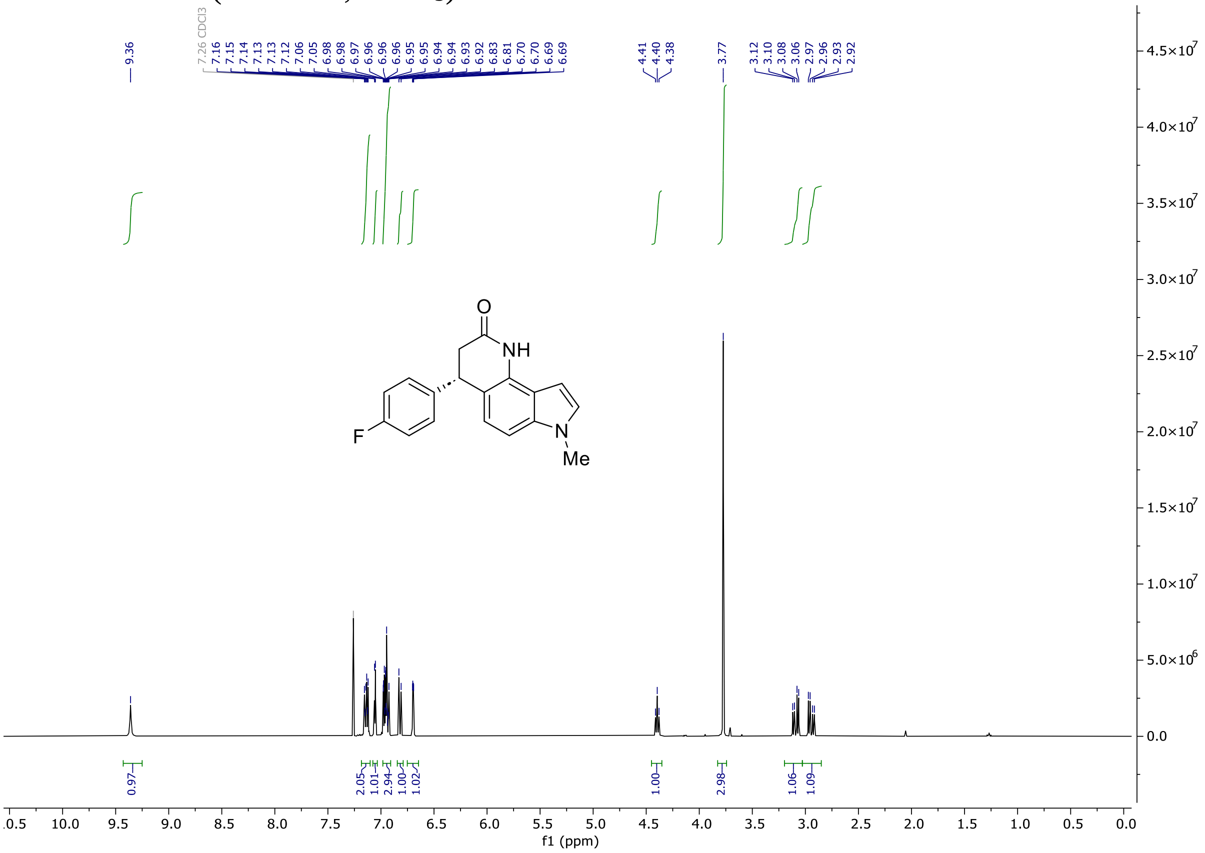

^{13}C{^{1}H} NMR of **3m** (101 MHz, $CDCl_3$)

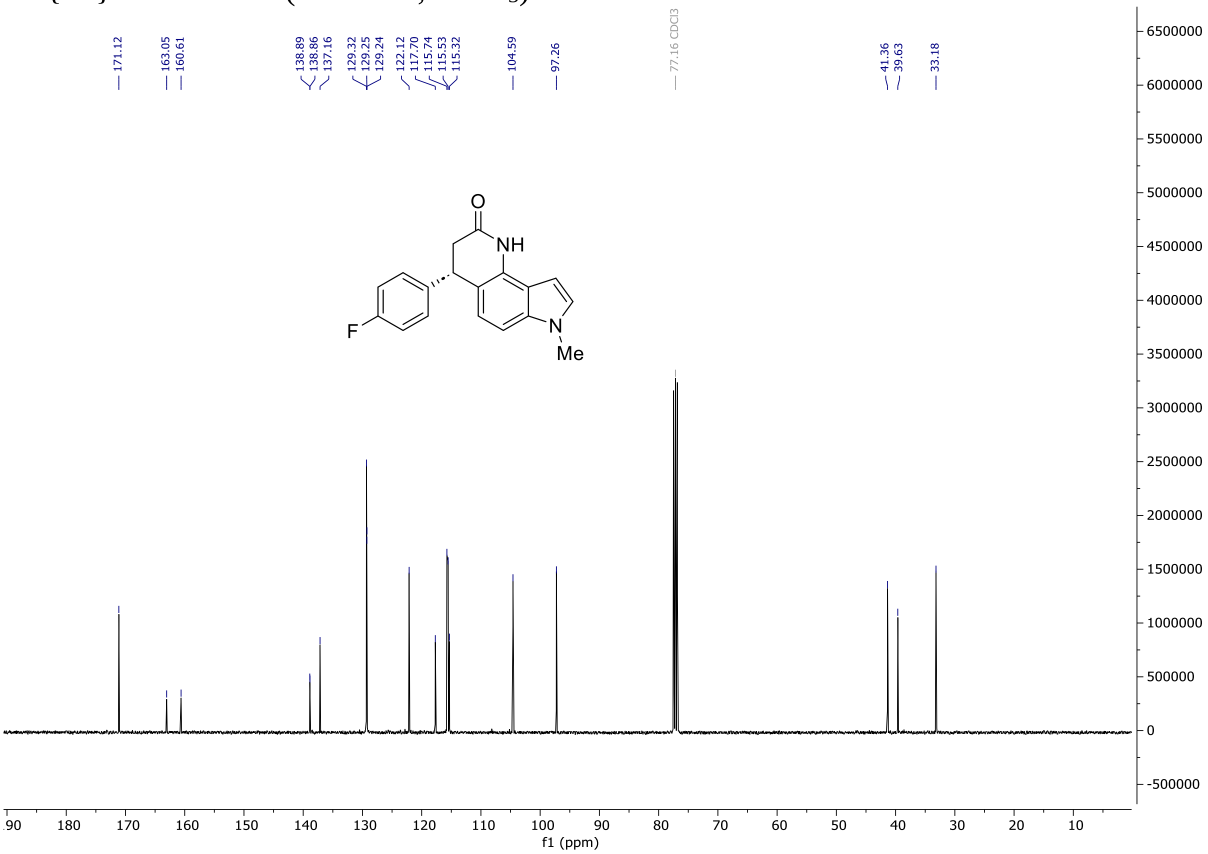

^{19}F NMR of **3m** (376 MHz, $CDCl_3$)

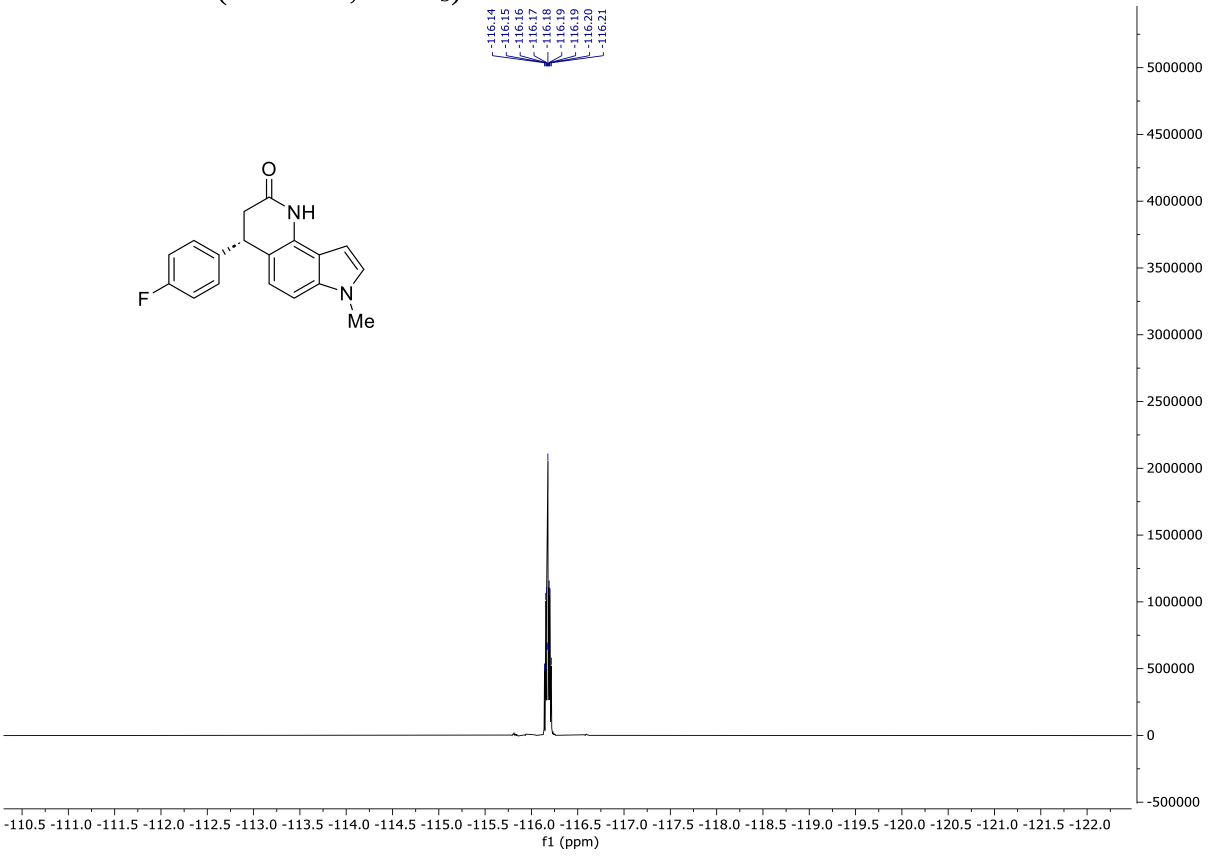

(*S*)-4-(4-Chlorophenyl)-7-methyl-1,3,4,7-tetrahydro-2*H*-pyrrolo[2,3-*h*]quinolin-2-one (3n)

^{1}H NMR of **3n** (400 MHz, $CDCl_3$)

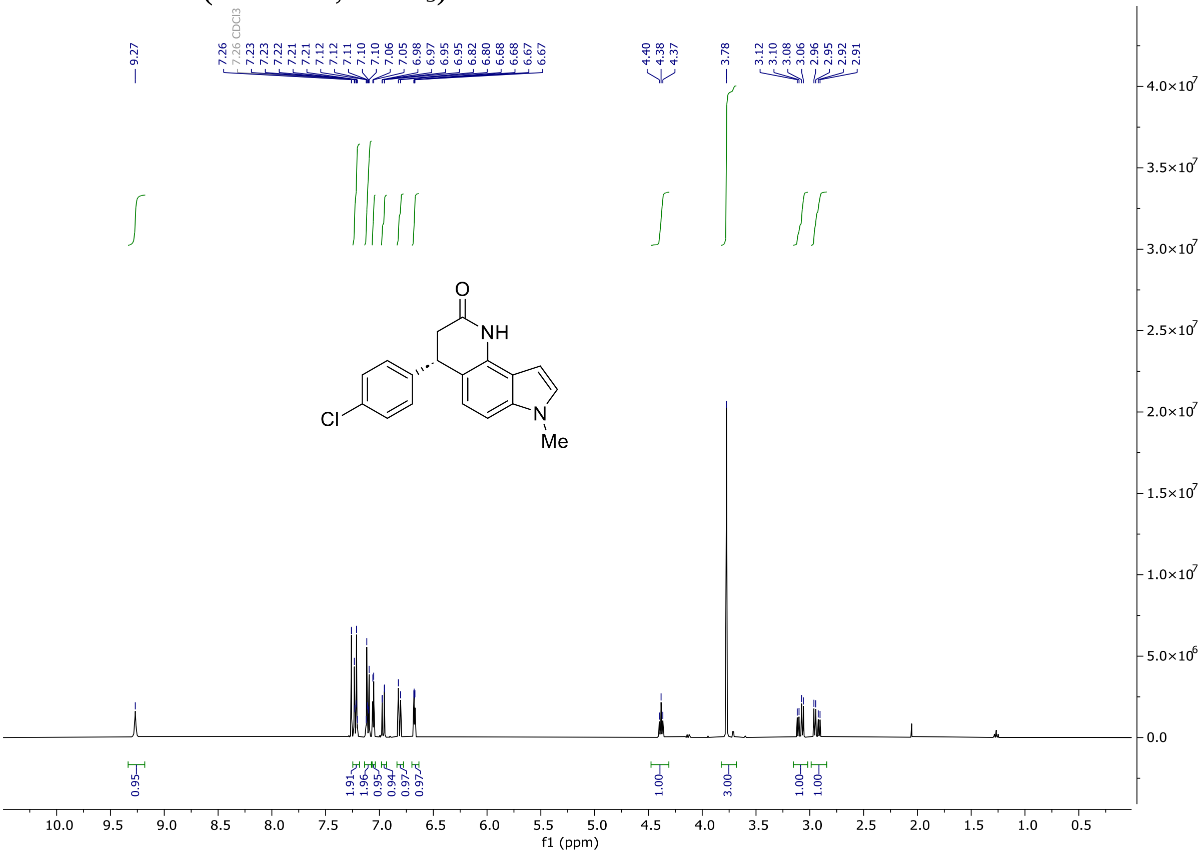

^{13}C{^{1}H} NMR of **3n** (101 MHz, $CDCl_3$)

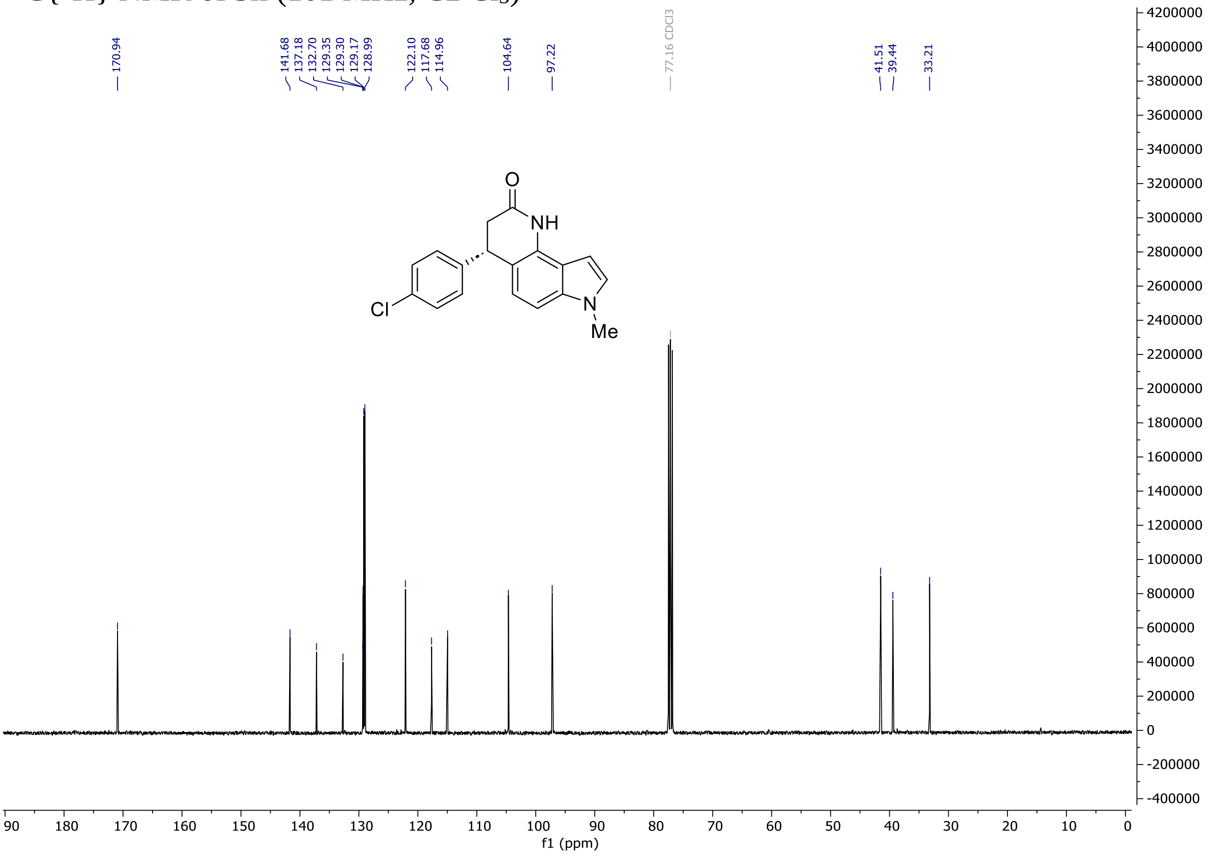

(*S*)-4-(4-Bromophenyl)-7-methyl-1,3,4,7-tetrahydro-2*H*-pyrrolo[2,3-*h*]quinolin-2-one (3o)

^{1}H NMR of **3o** (400 MHz, $CDCl_3$)

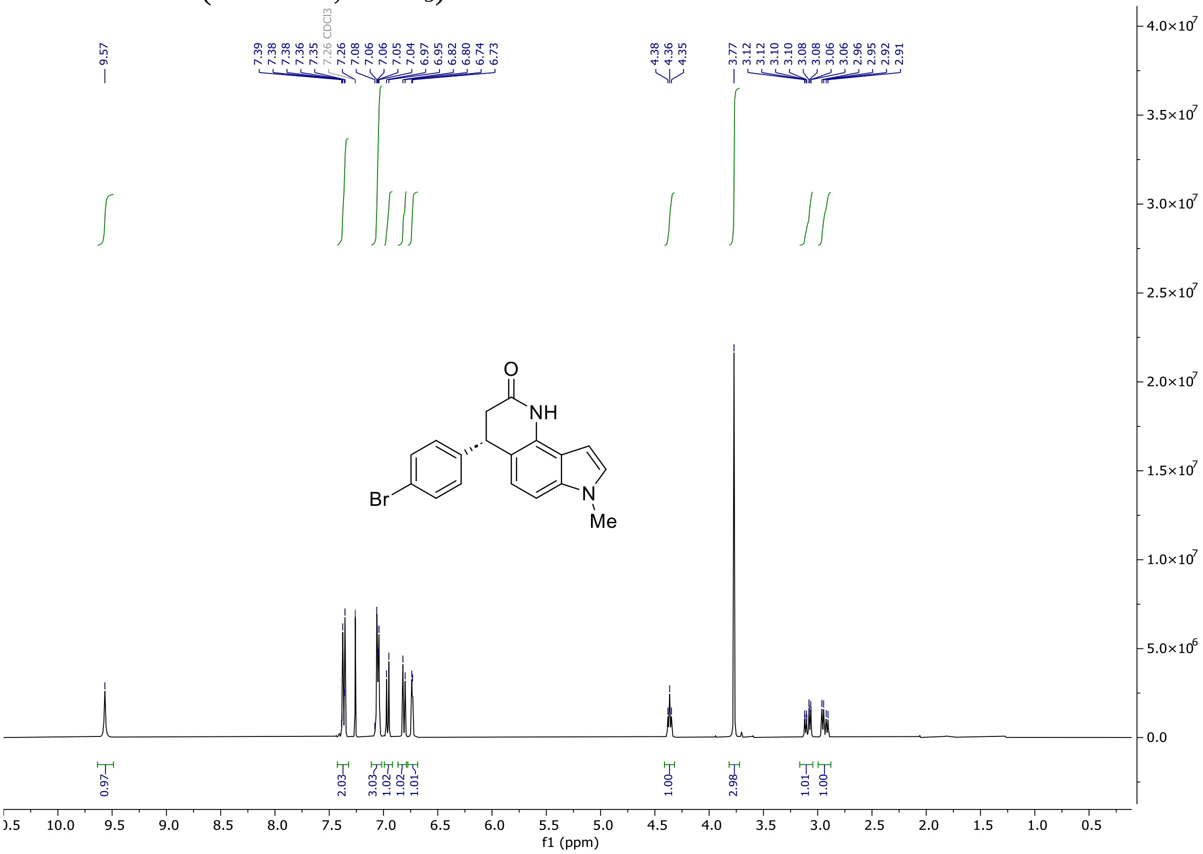

^{13}C{^{1}H} NMR of **3o** (101 MHz, $CDCl_3$)

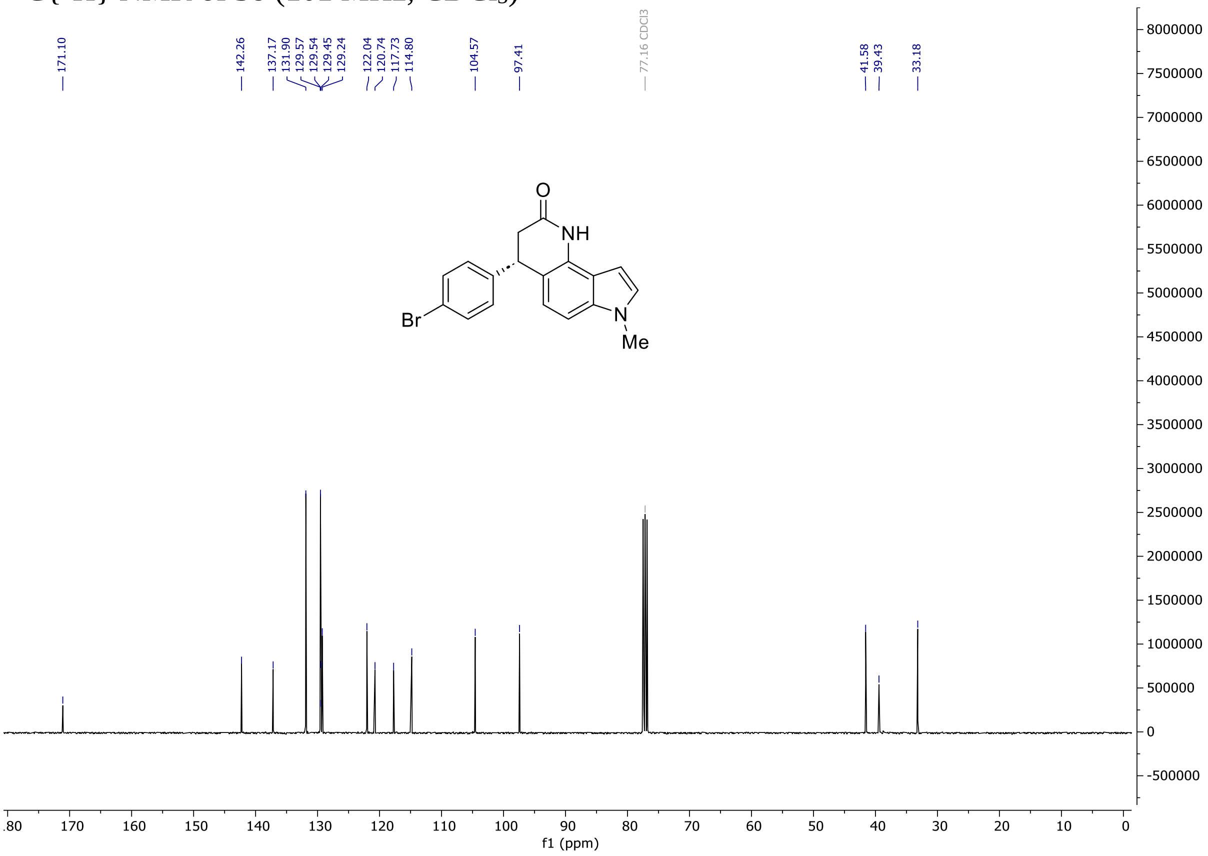

(*S*)-4-(3-Chlorophenyl)-7-methyl-1,3,4,7-tetrahydro-2*H*-pyrrolo[2,3-*h*]quinolin-2-one (3p)

^{1}H NMR of **3p** (400 MHz, $CDCl_3$)

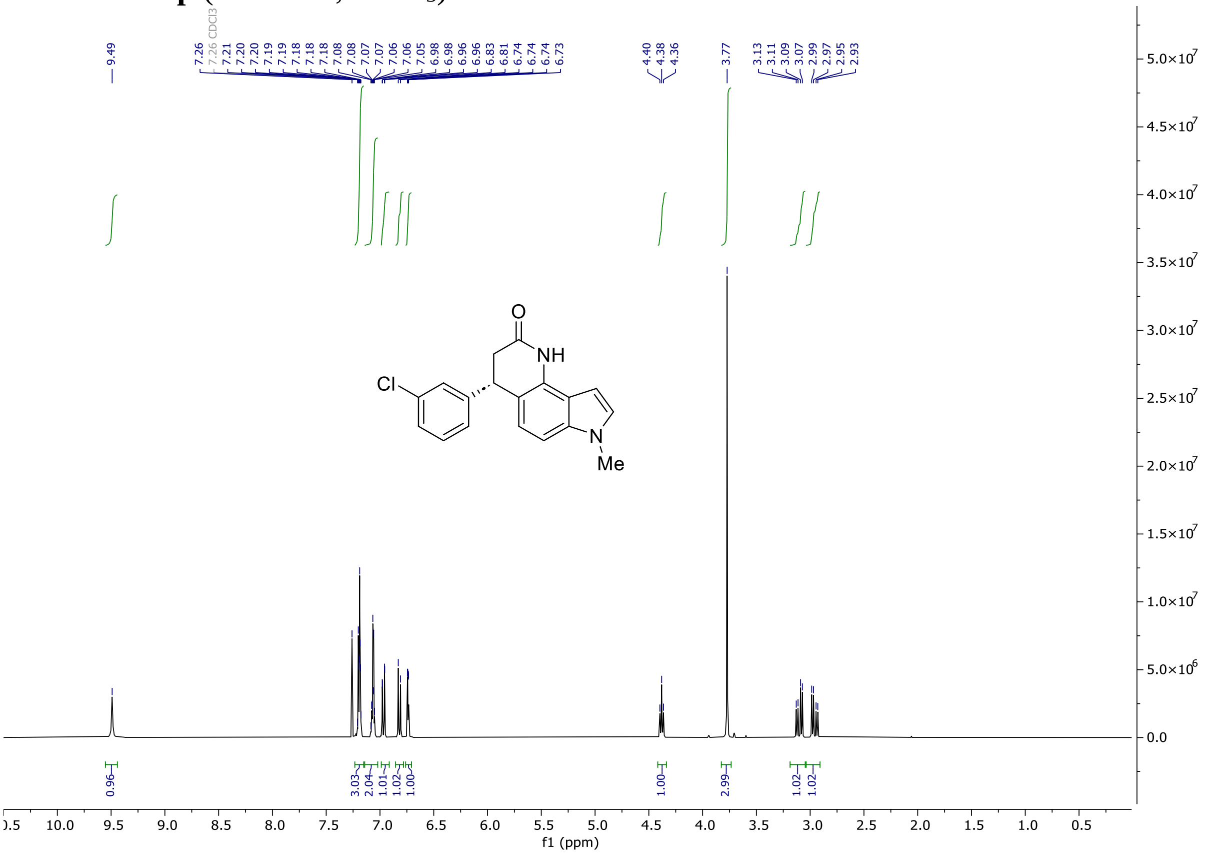

^{13}C{^{1}H} NMR of **3p** (101 MHz, $CDCl_3$)

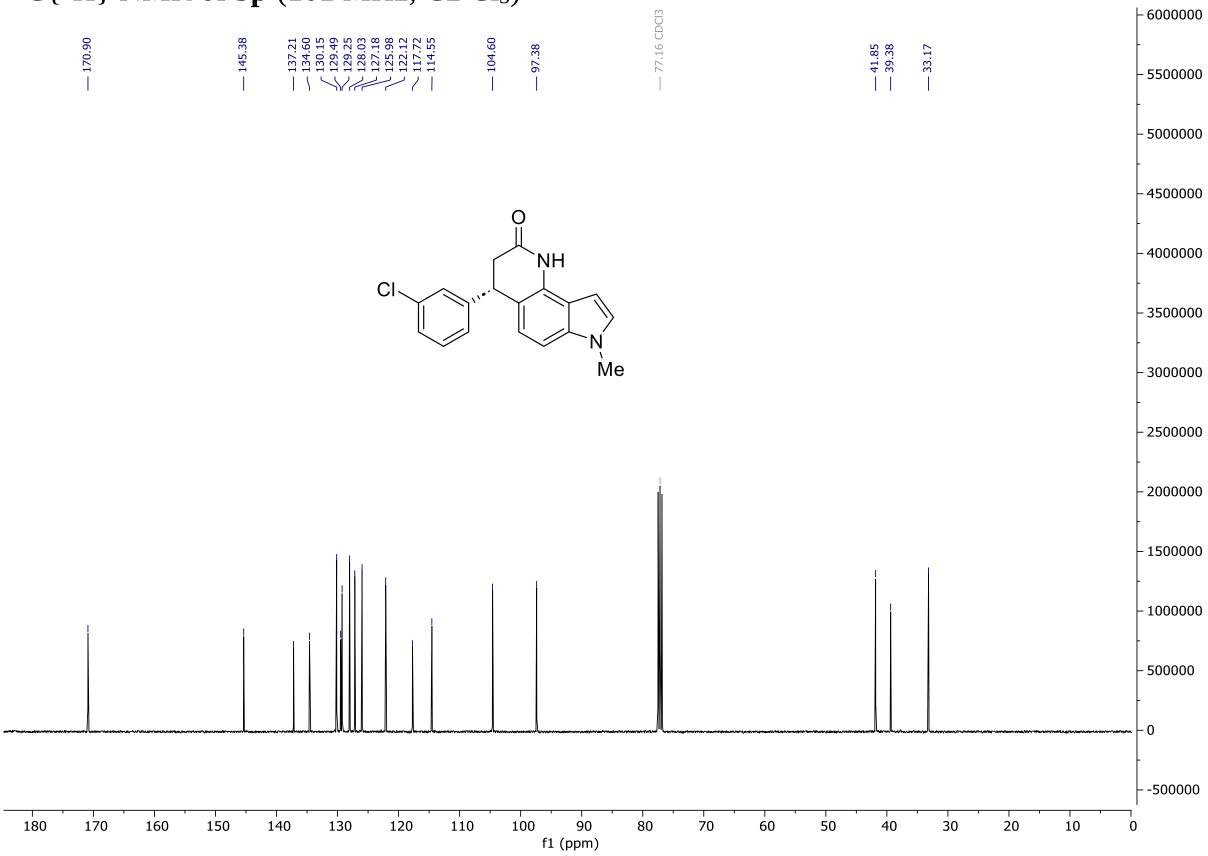

(*R*)-4-(Furan-2-yl)-7-methyl-1,3,4,7-tetrahydro-2*H*-pyrrolo[2,3-*h*]quinolin-2-one (3p)

^{1}H NMR of **3q** (400 MHz, $CDCl_3$)

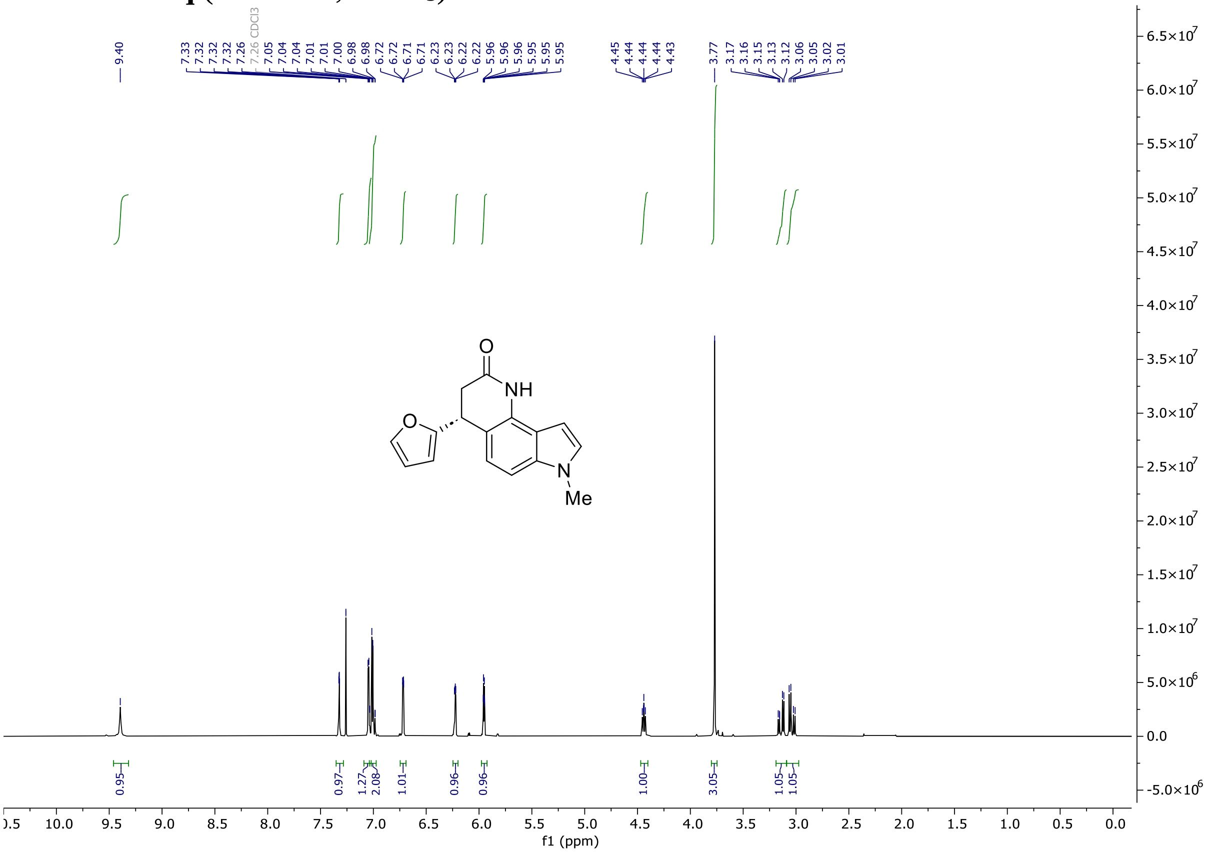

^{13}C{^{1}H} NMR of **3q** (101 MHz, $CDCl_3$)

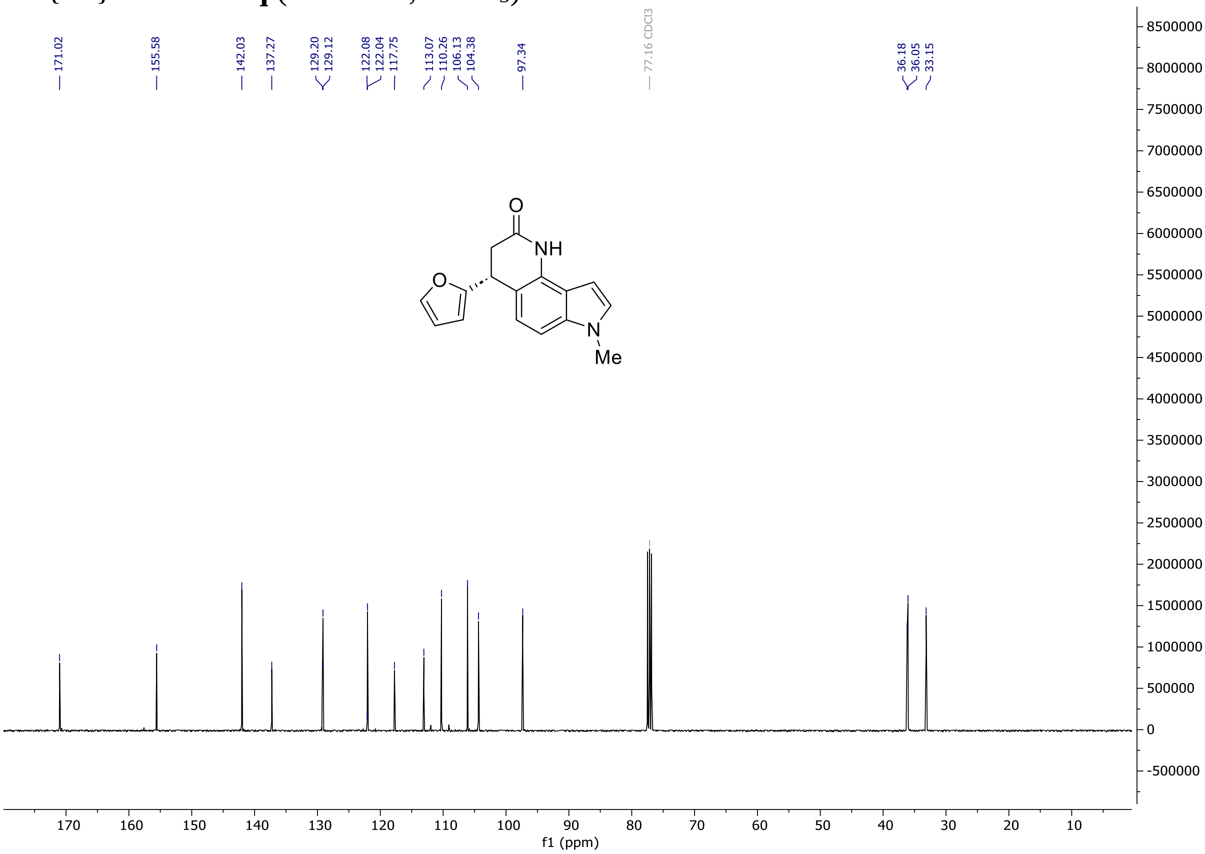

(*R*)-7-Methyl-4-(thiophen-2-yl)-1,3,4,7-tetrahydro-2H-pyrrolo[2,3-h]quinolin-2-one (3q)

^{1}H NMR of **3r** (400 MHz, $CDCl_3$)

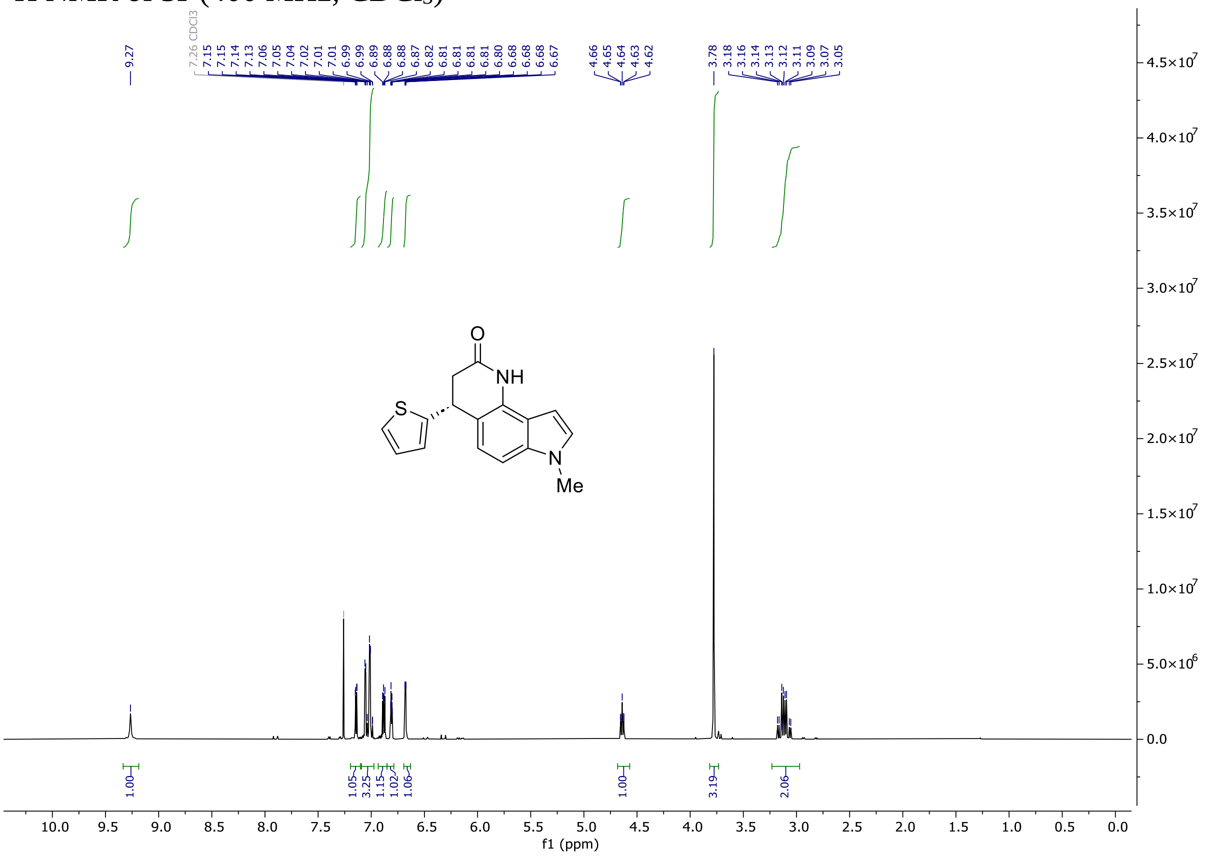

^{13}C{^{1}H} NMR of **3r** (101 MHz, $CDCl_3$)

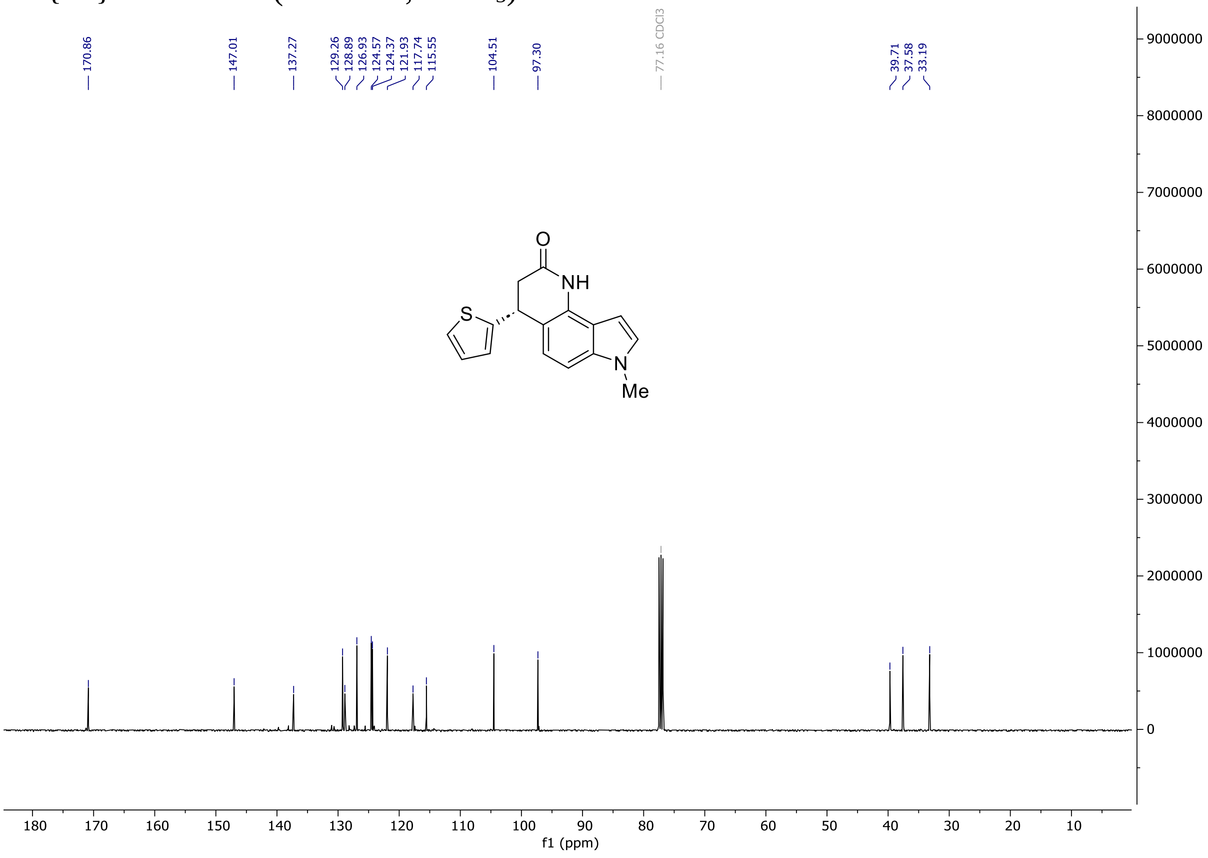

Ethyl (*R*)-7-methyl-2-oxo-2,3,4,7-tetrahydro-1*H*-pyrrolo[2,3-*h*]quinoline-4-carboxylate (3s)

^{1}H NMR of **3s** (400 MHz, $CDCl_3$)

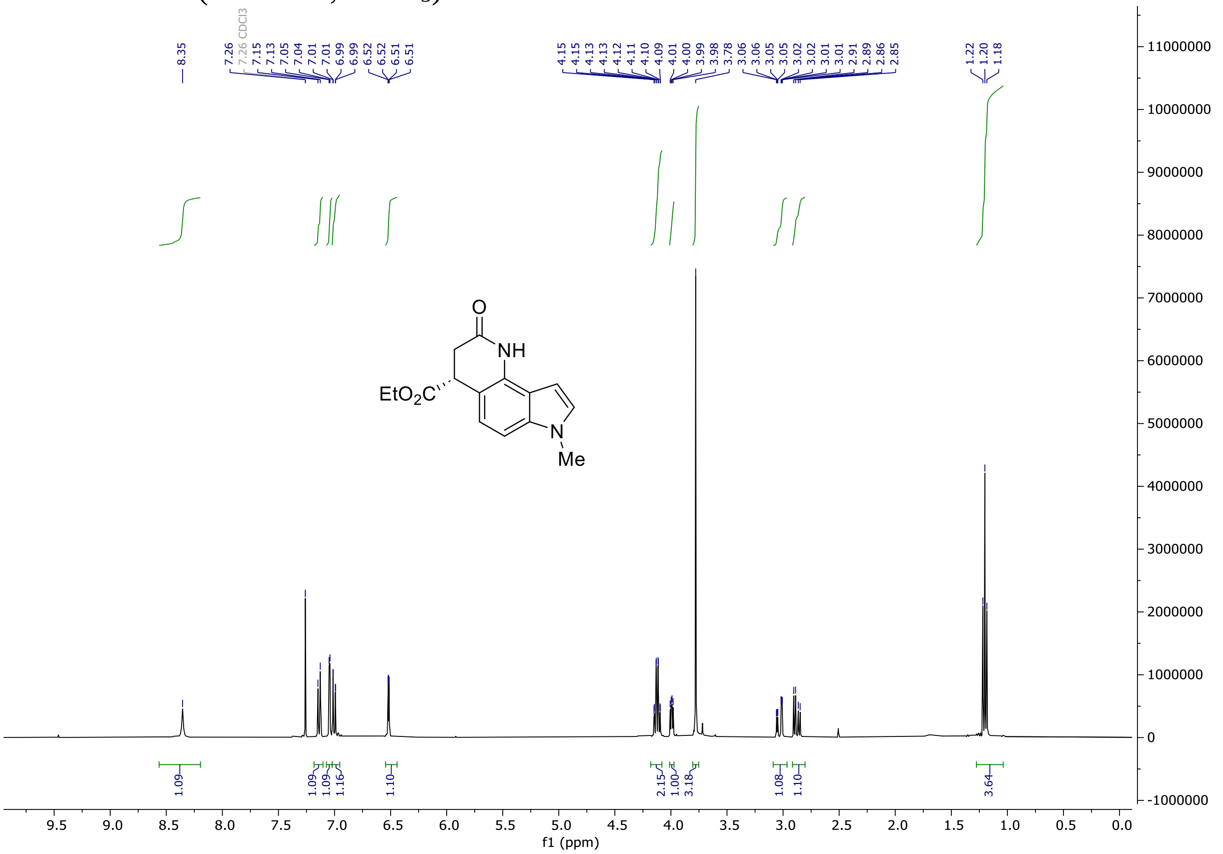

^{13}C{^{1}H} NMR of **3s** (101 MHz, $CDCl_3$)

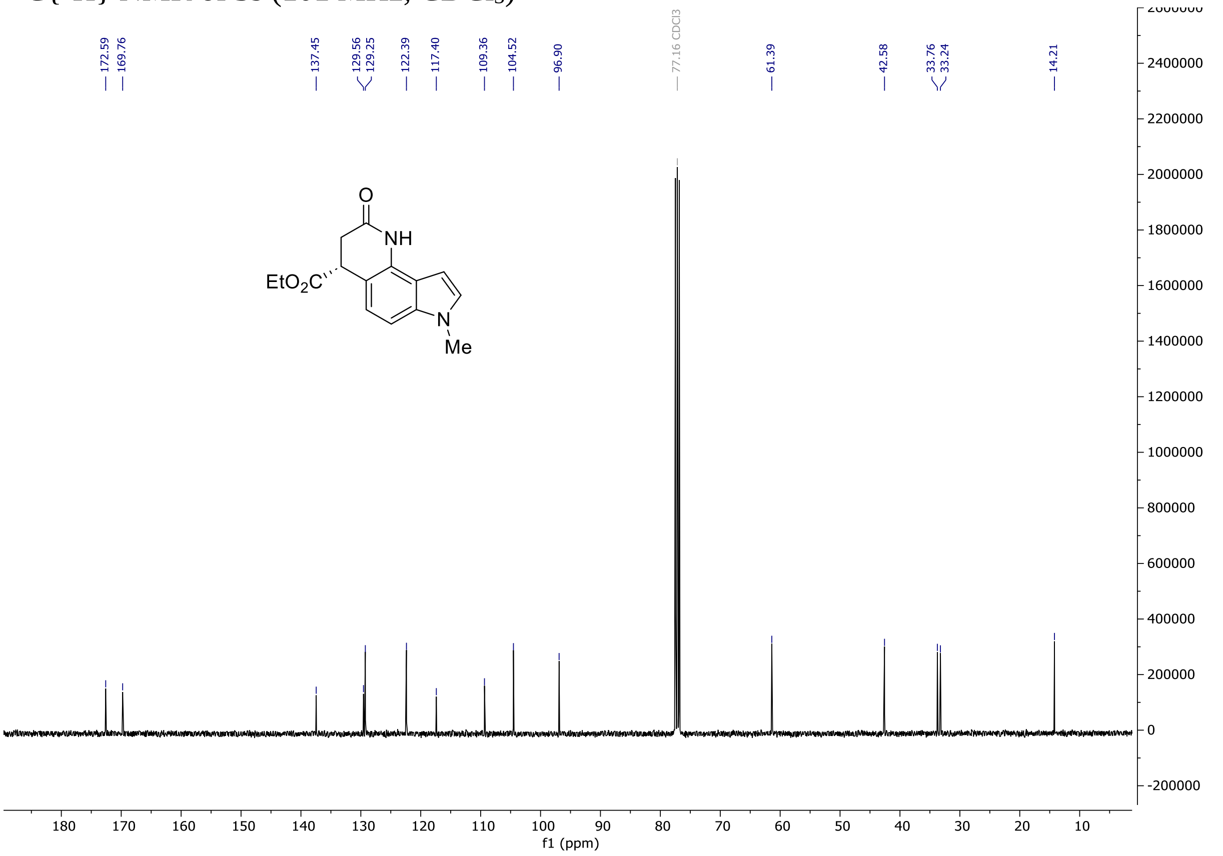

(*R*)-4-Butyl-7-methyl-1,3,4,7-tetrahydro-2*H*-pyrrolo[2,3-*h*]quinolin-2-one (3s)

^{1}H NMR of **3t** (400 MHz, $CDCl_3$)

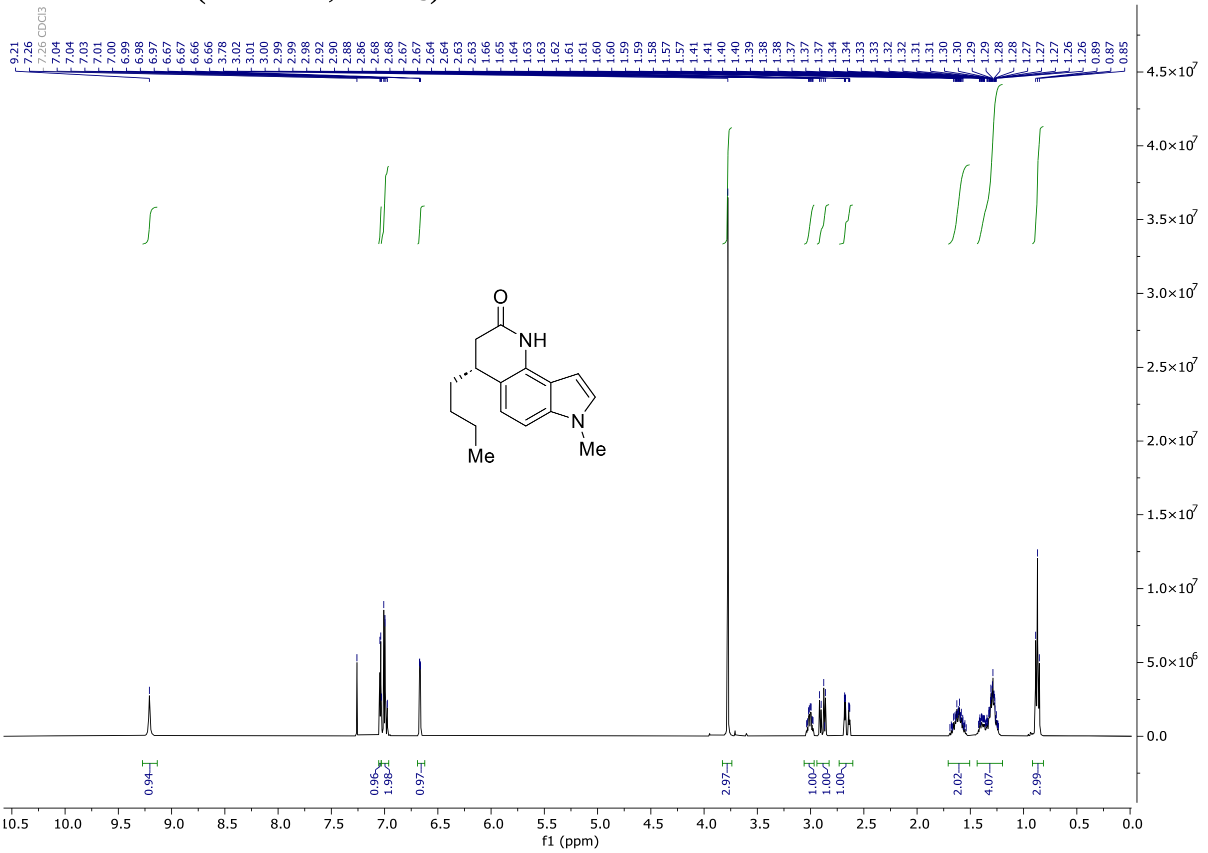

^{13}C{^{1}H} NMR of **3t** (101 MHz, $CDCl_3$)

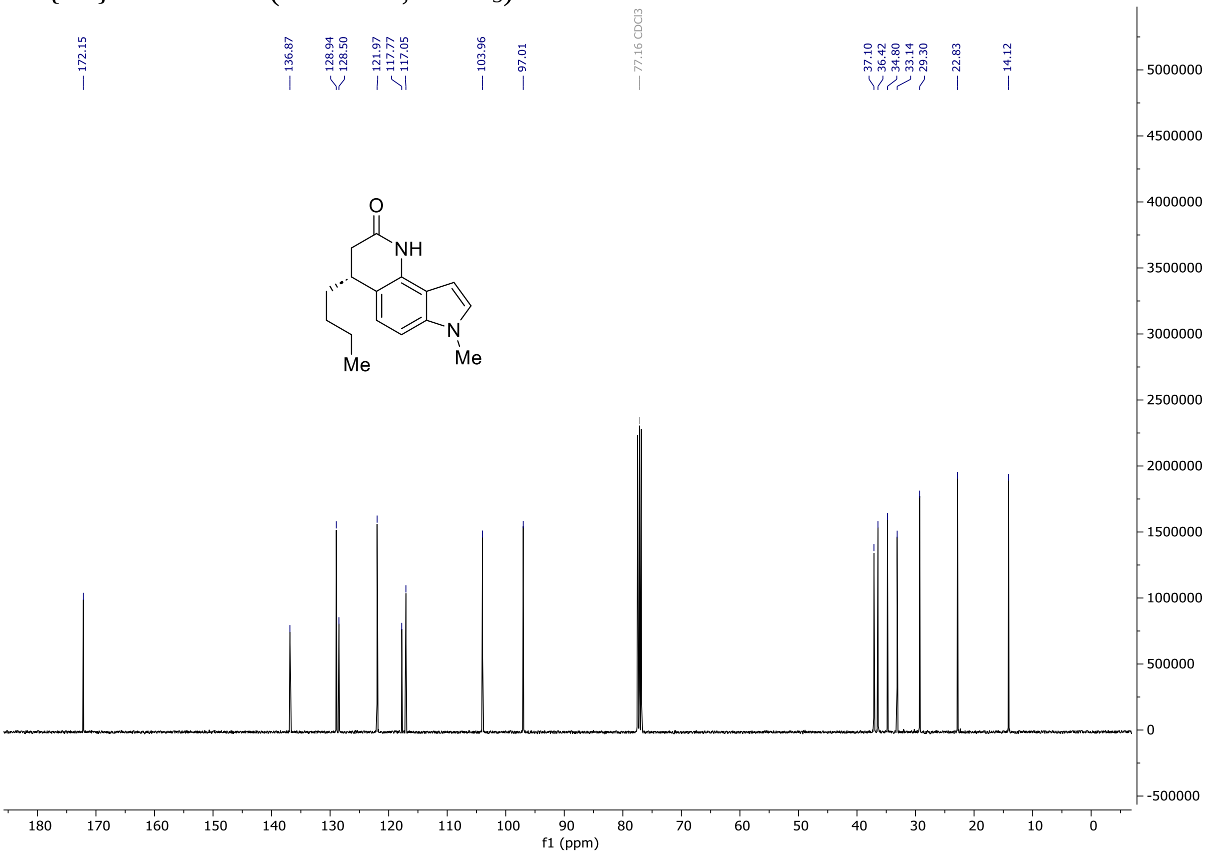

3-Methyl-9-phenyl-3,6,8,9-tetrahydro-7*H*-9λ^3-pyrrolo[3,2-*f*]quinolin-7-one (3t)

^{1}H NMR of **3u** (400 MHz, $CDCl_3$)

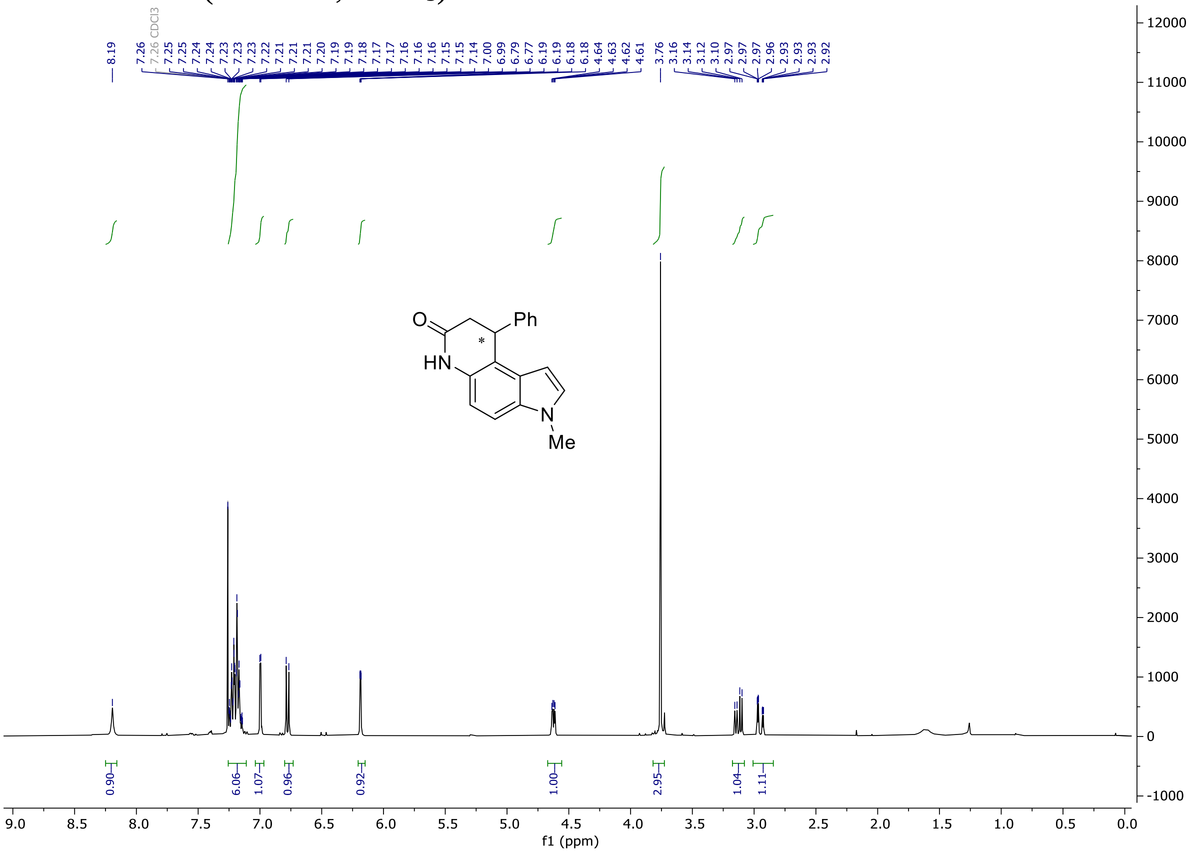

^{13}C{^{1}H} NMR of **3u** (101 MHz, $CDCl_3$)

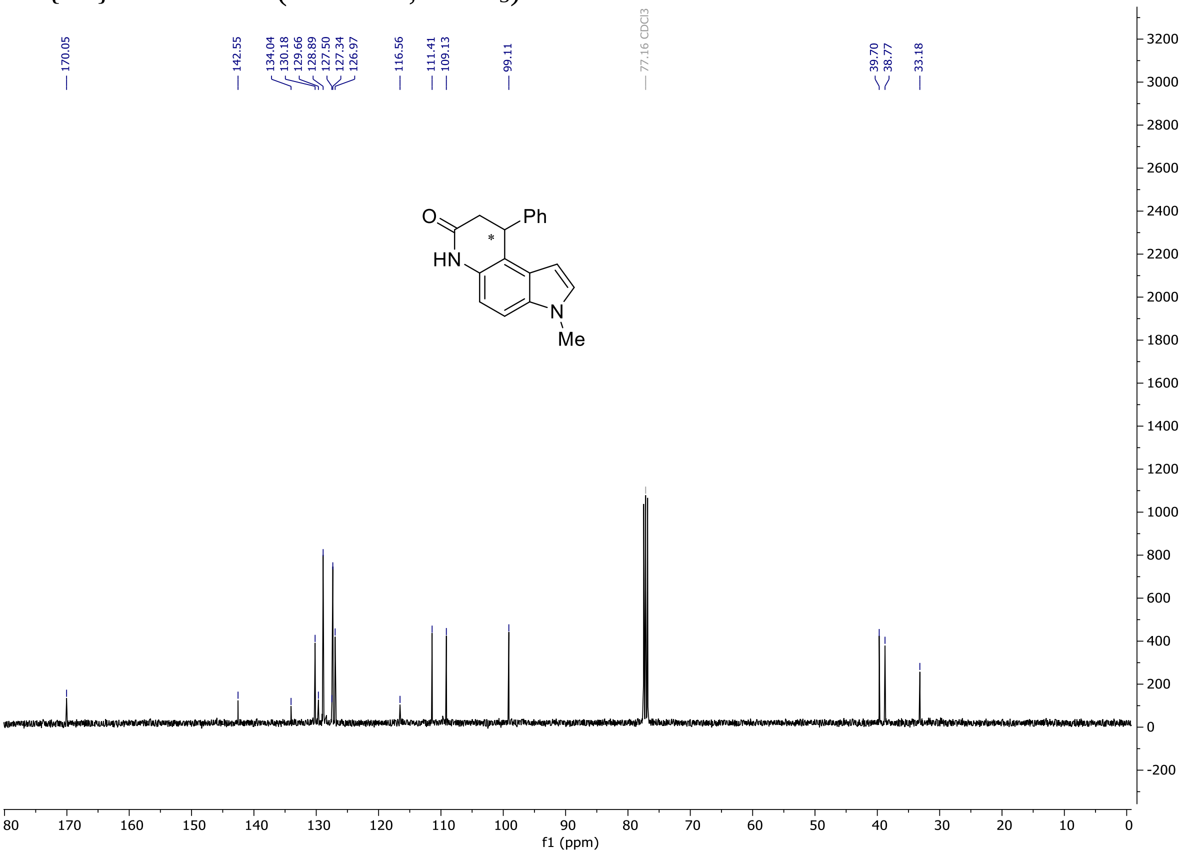

1-Methyl-6-phenyl-1,6,7,9-tetrahydro-8*H*-6λ³-pyrrolo[3,2-*h*]quinolin-8-one (3v)

^{1}H NMR of **3v** (400 MHz, $CDCl_3$)

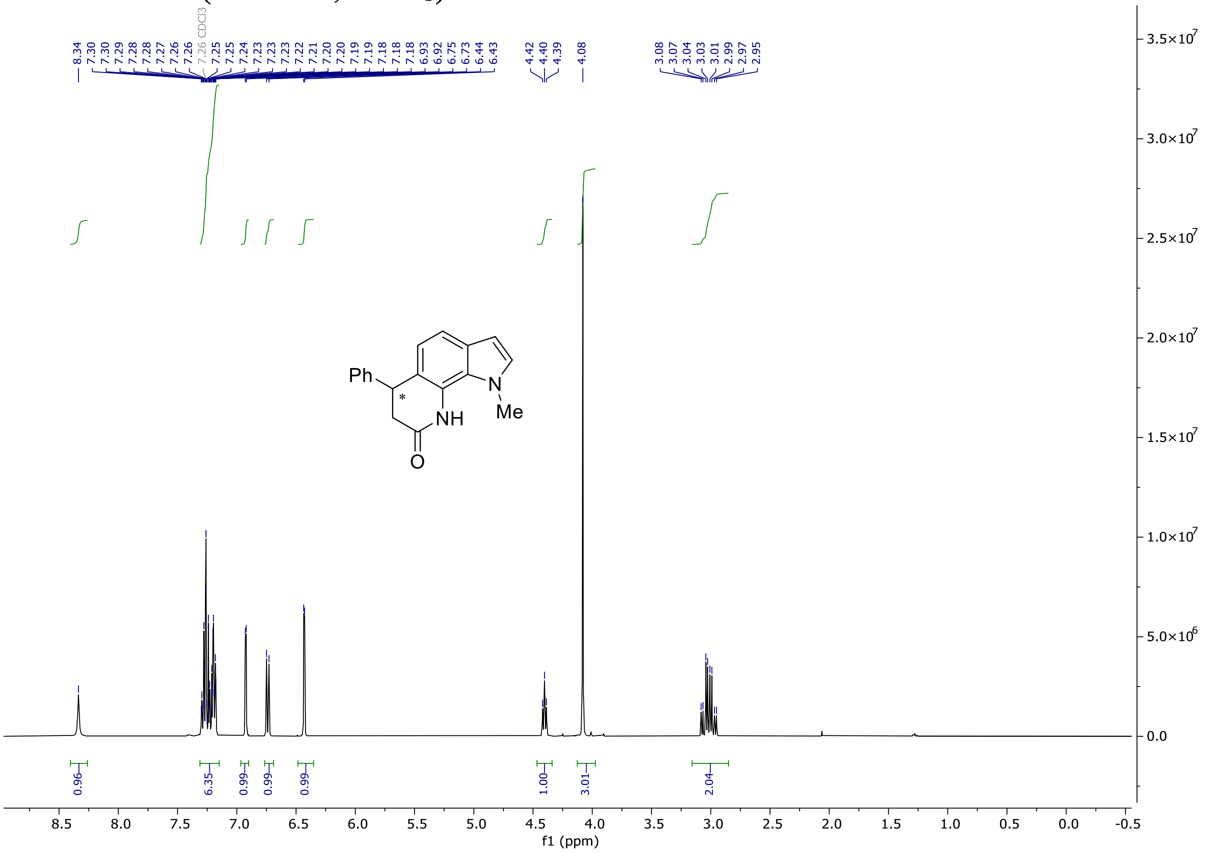

^{13}C{^{1}H} NMR of **3v** (101 MHz, $CDCl_3$)

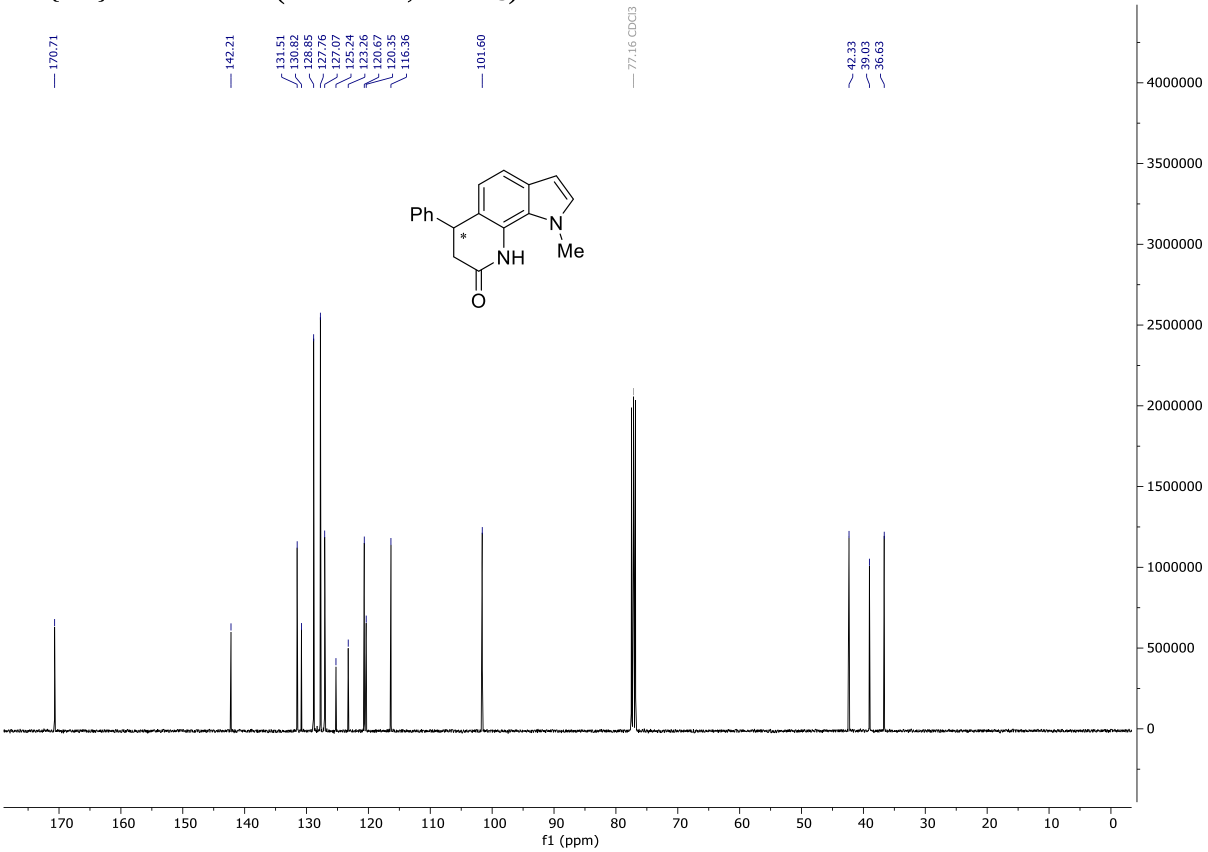

(*S*)-1,7-Dimethyl-4-phenyl-1,3,4,7-tetrahydro-2*H*-pyrrolo[2,3-*h*]quinolin-2-one (5)

^{1}H NMR of **5** (400 MHz, $CDCl_3$)

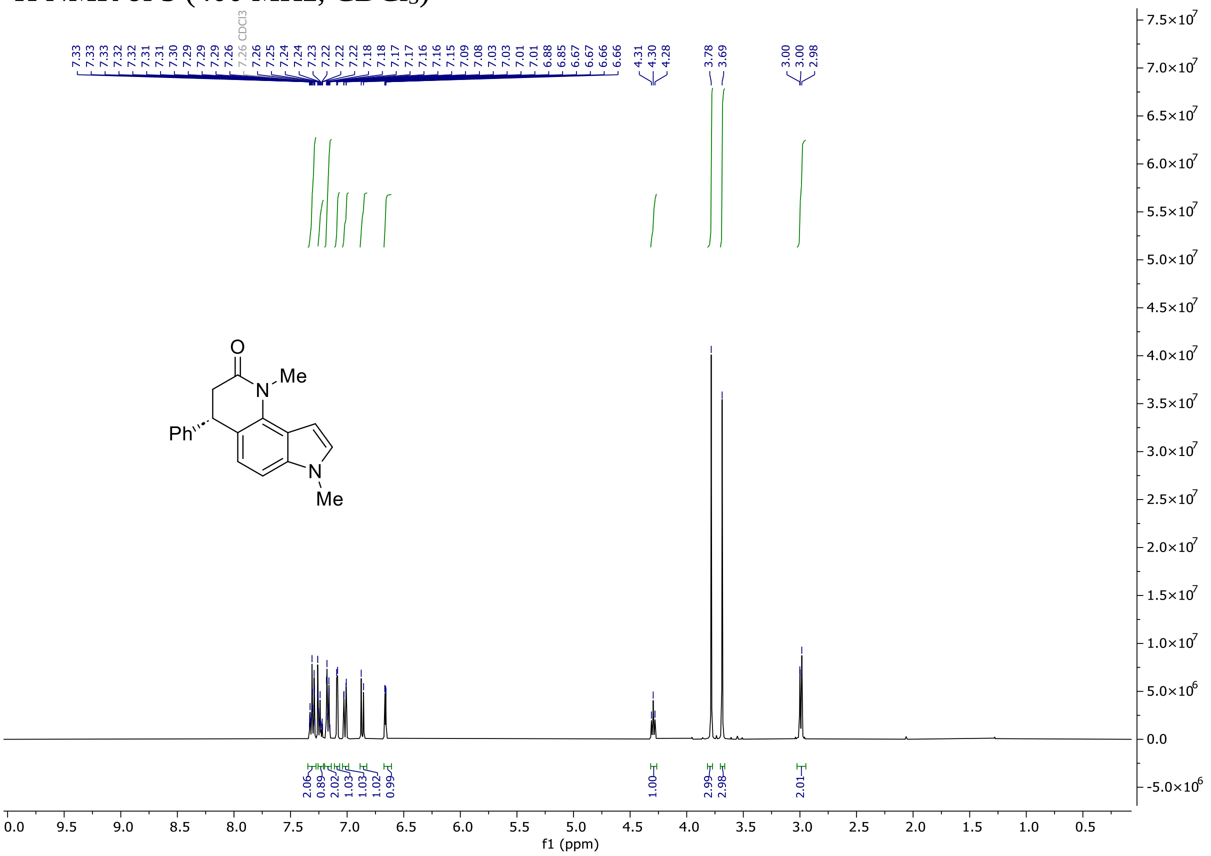

^{13}C{^{1}H} NMR of **5** (101 MHz, $CDCl_3$)

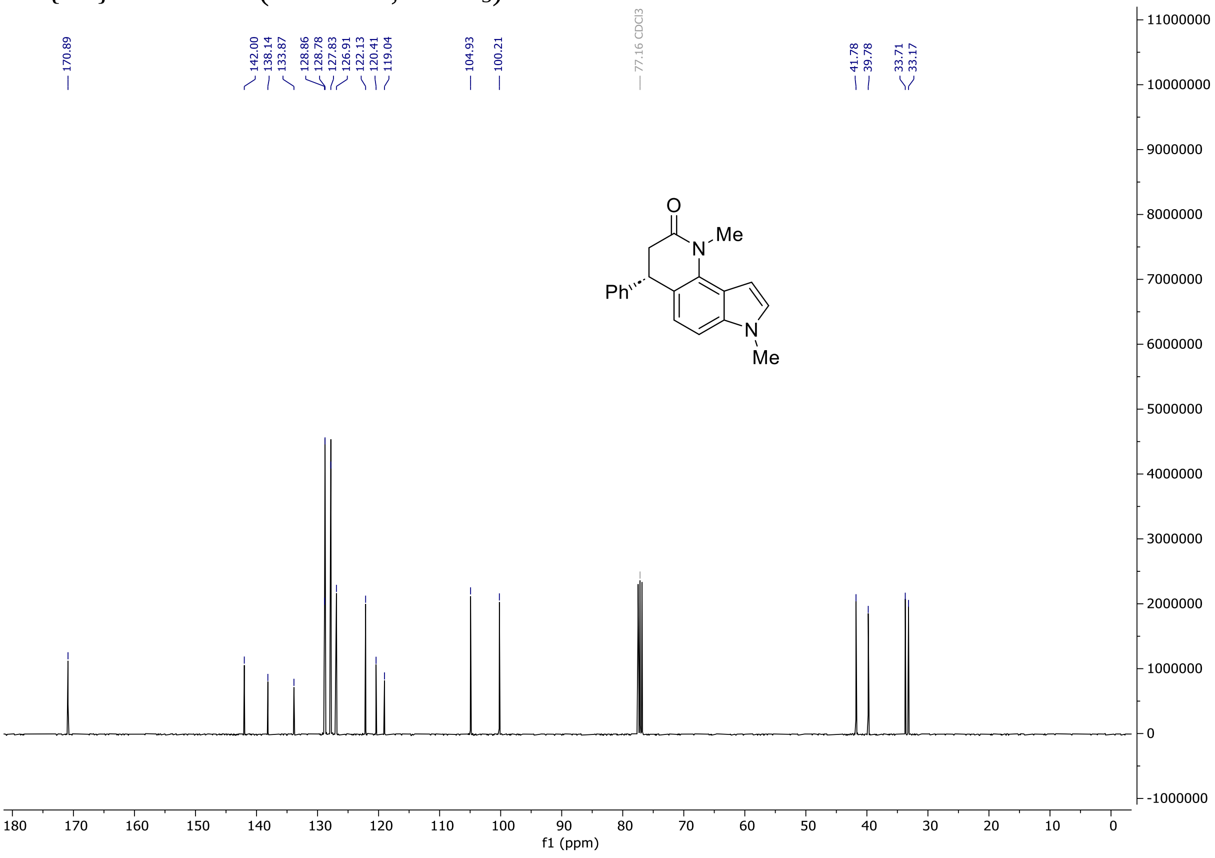

(*S*)-7-Methyl-4-phenyl-2,3,4,7-tetrahydro-1*H*-pyrrolo[2,3-*h*]quinoline (6)

^{1}H NMR of **6** (400 MHz, $CDCl_3$)

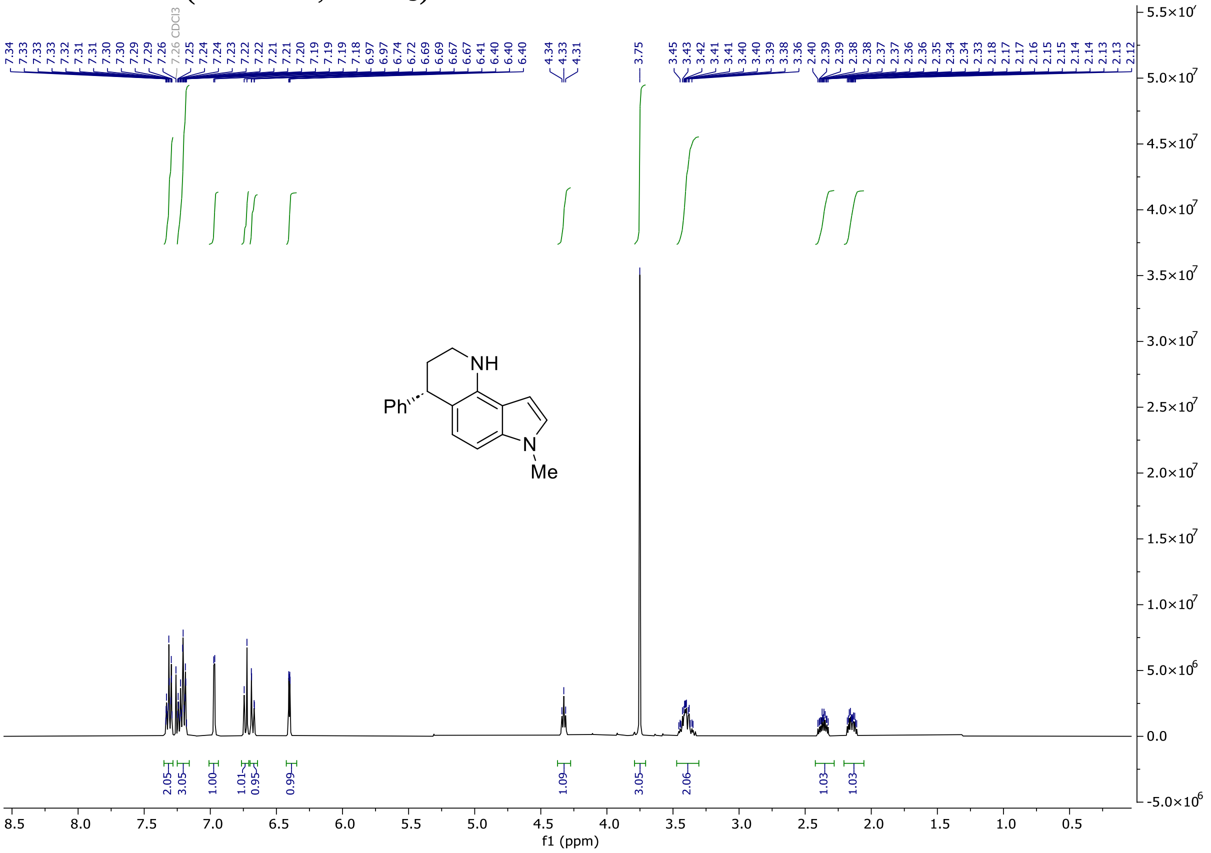

^{13}C{^{1}H} NMR of **6** (101 MHz, $CDCl_3$)

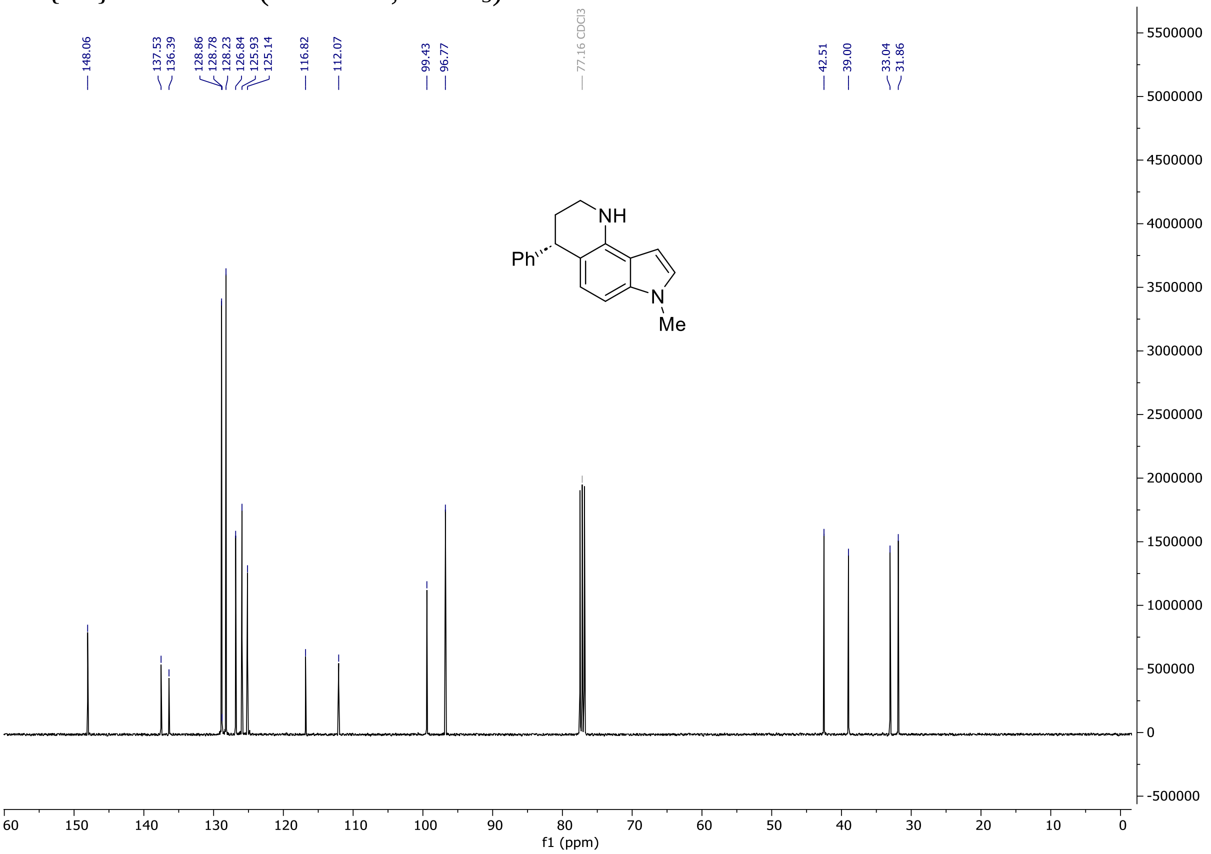

(*S*)-7-Methyl-9-(3-oxobutyl)-4-phenyl-1,3,4,7-tetrahydro-2*H*-pyrrolo[2,3-*h*]quinolin-2-one (7)

^{1}H NMR of **7** (400 MHz, $CDCl_3$)

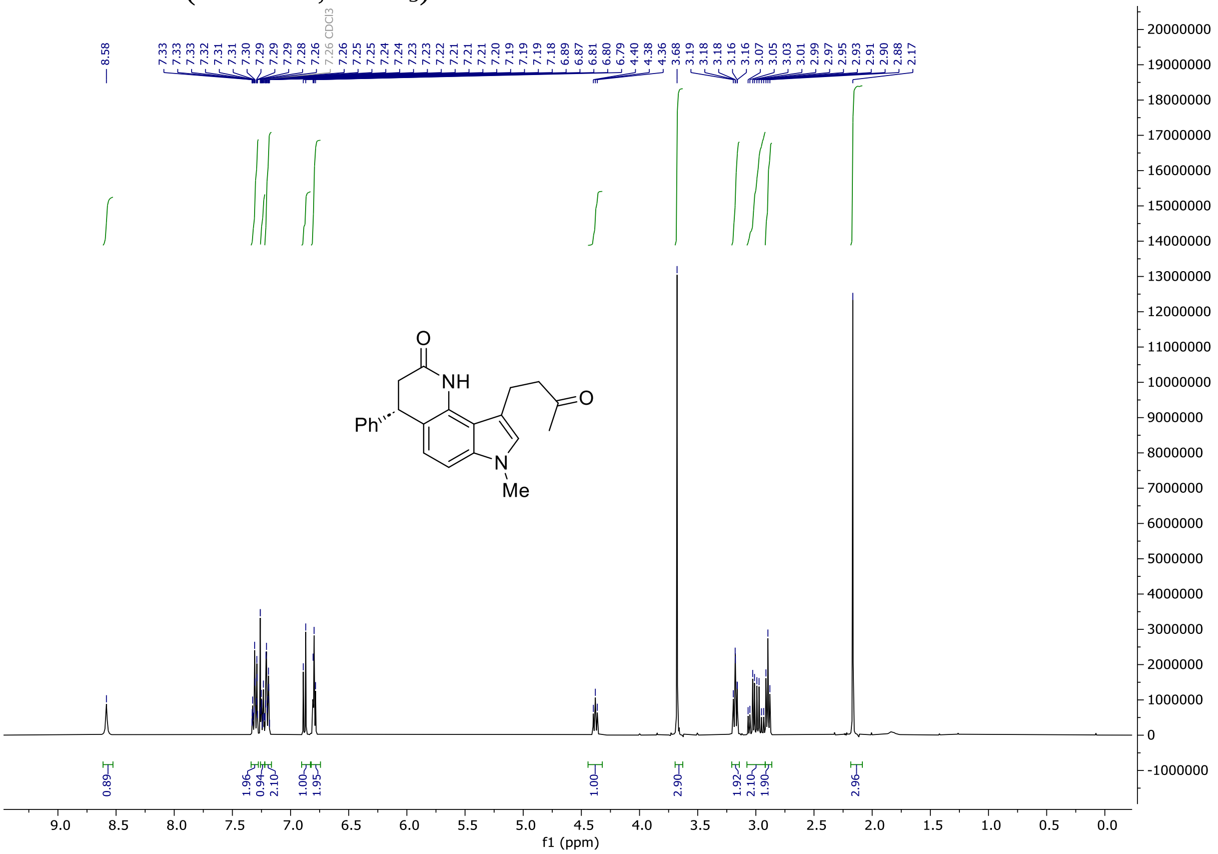

$^{13}C\{^{1}H\}$ NMR of **7** (101 MHz, $CDCl_3$)

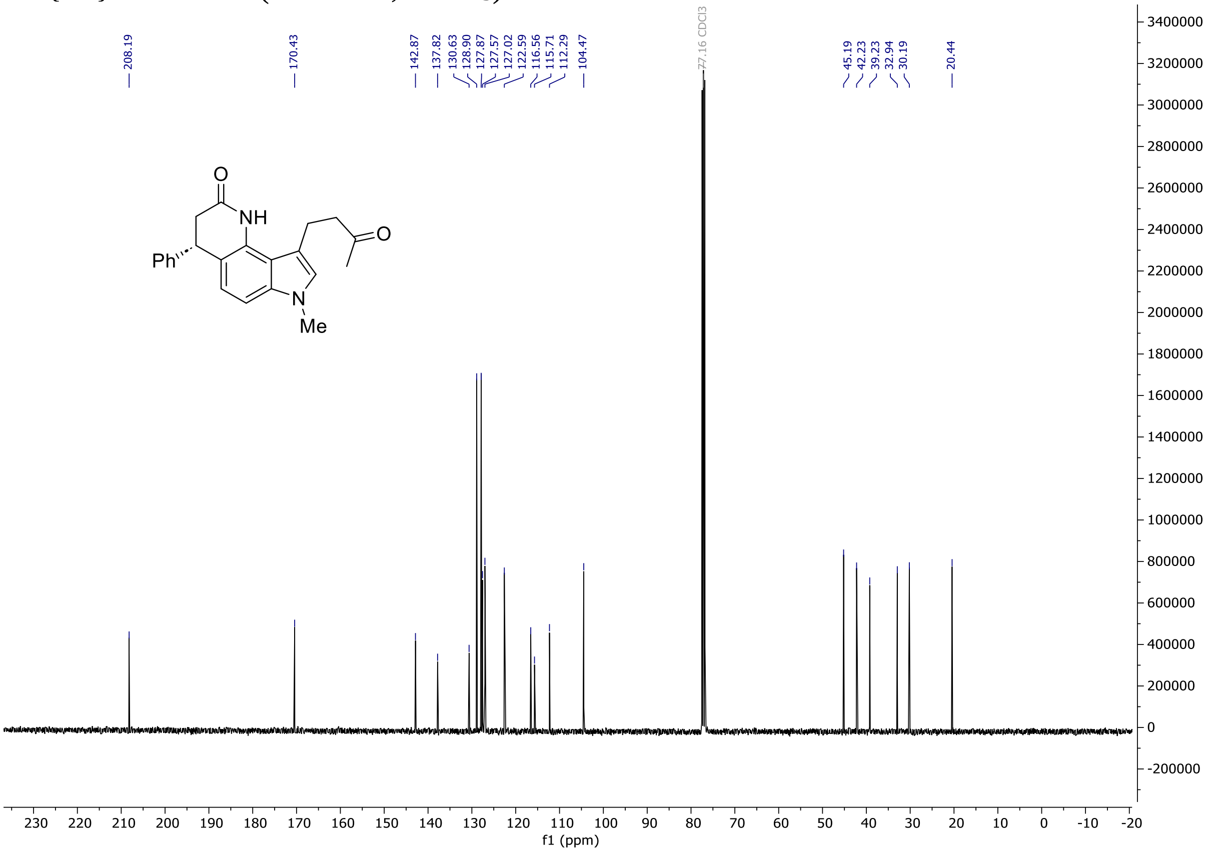

(*S*)-7-Methyl-4-phenyl-1,3,4,7,8,9-hexahydro-2*H*-pyrrolo[2,3-*h*]quinolin-2-one (8)

^{1}H NMR of **8** (400 MHz, $CDCl_3$)

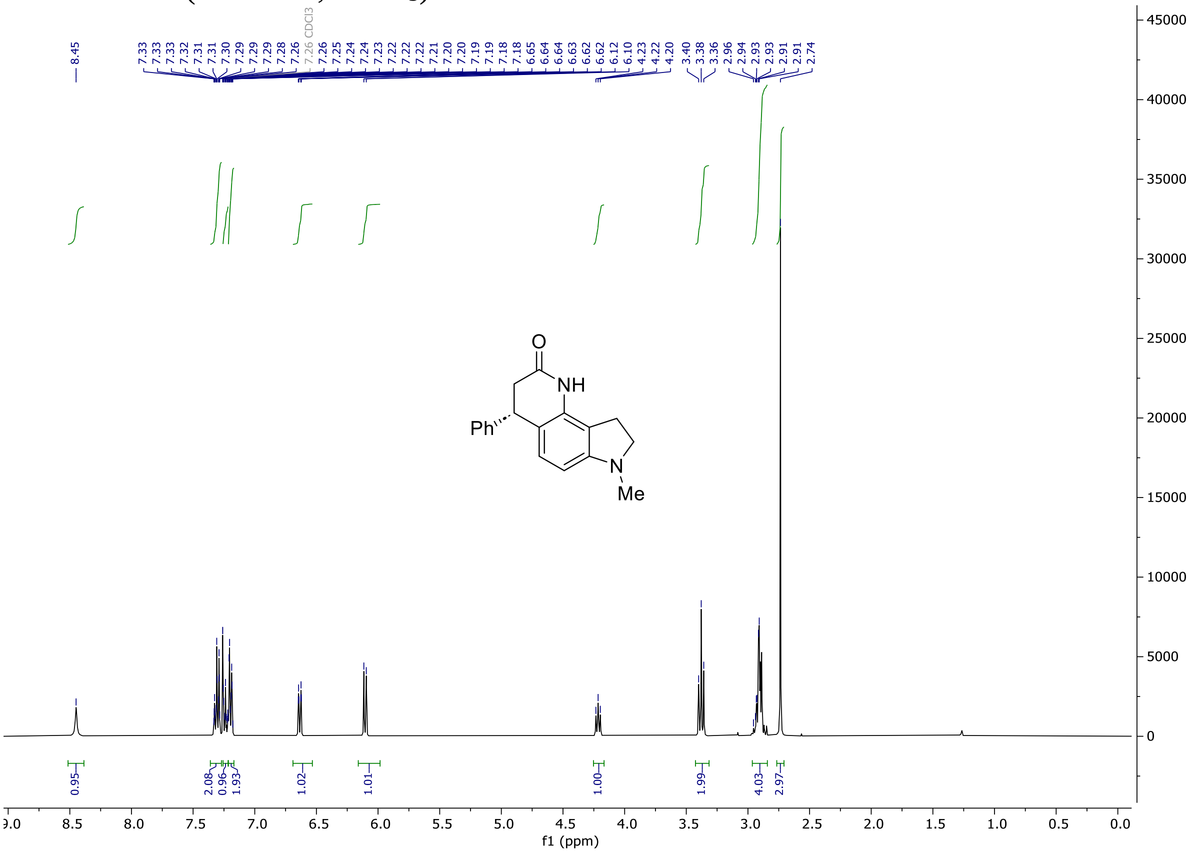

^{13}C{^{1}H} NMR of **8** (101 MHz, $CDCl_3$)

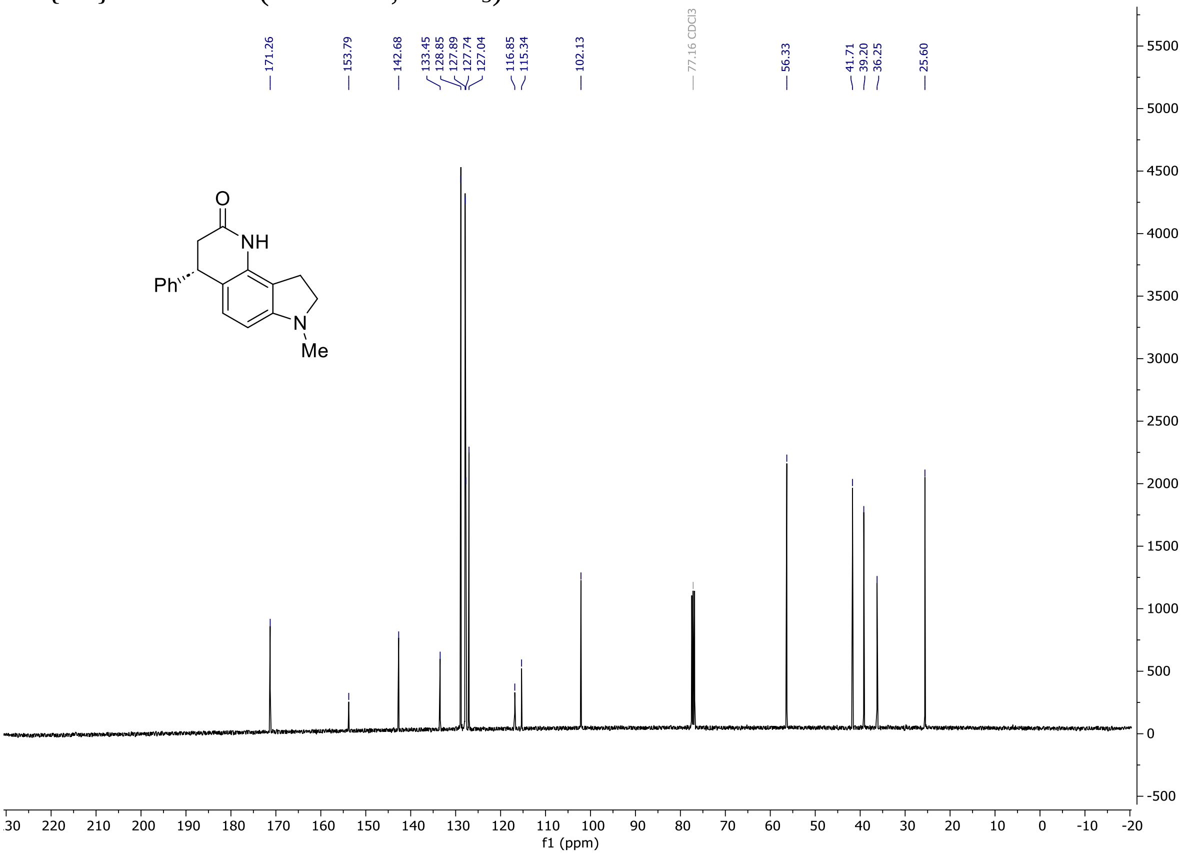

Chiral HPLC

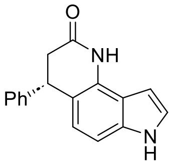

Conditions: IB column
mobile phase: *n*-heptane/*i*-PrOH – 80:20
λ = 252 nm, V = 1.0 ml/min, t = 25 °C
for **3a**: t_R = 13.0 min (minor), t_R = 15.6 min (major)

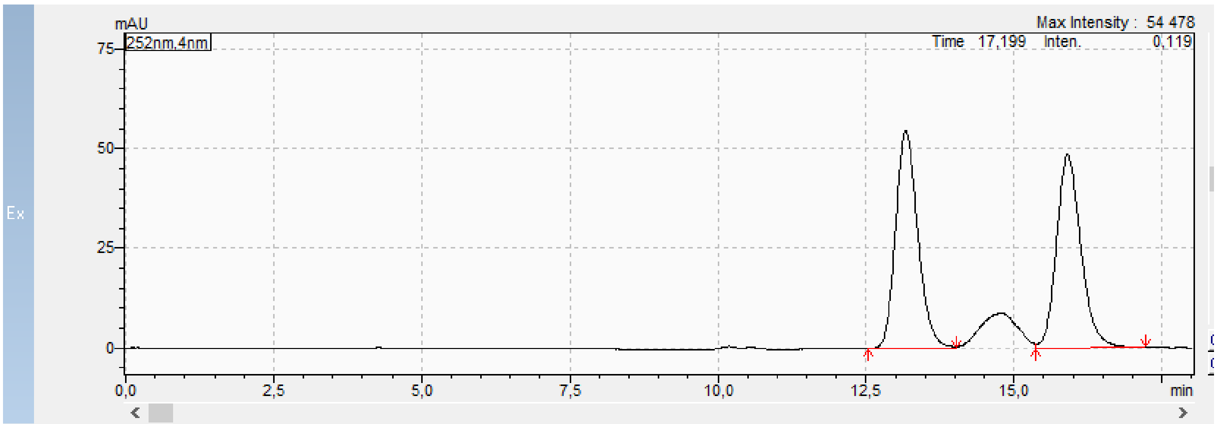

Results View - Peak Table

Peak Table | Compound | Group | Calibration Curve

Peak#	Ret. Time	Area	Height	Peak Start	Peak End	Mark	Conc.	Unit	Area%
1	13,173	1433922	54665	12,533	14,016		50,767		50,767
2	15,910	1390592	48620	15,371	17,216	S	49,233		49,233
Total		2824514	103285				100,000		100,000

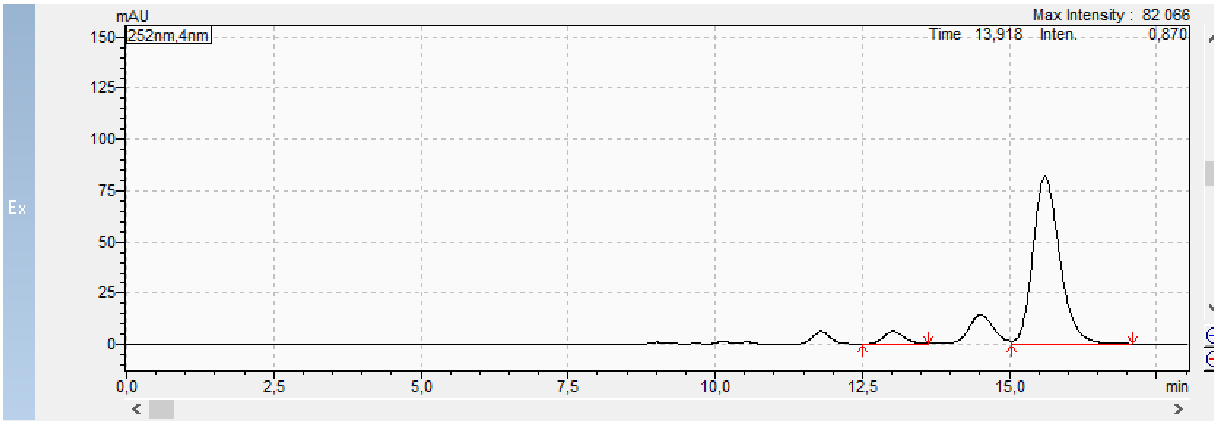

Results View - Peak Table

Peak Table | Compound | Group | Calibration Curve

Peak#	Ret. Time	Area	Height	Peak Start	Peak End	Mark	Conc.	Unit	Area%
1	13,024	152026	6097	12,501	13,621		5,566		5,566
2	15,604	2579219	81676	15,029	17,099		94,434		94,434
Total		2731245	87773				100,000		100,000

for **3a**: *er* = 94:6 (*ee* = 89%)

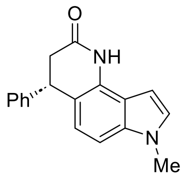

Conditions: IA column
mobile phase: *n*-heptane/*i*-PrOH – 80:20
λ = 310 nm, V = 1.0 ml/min, t = 25 °C
for **3b**: t_R = 12.5 min (minor), t_R = 20.0 min (major)

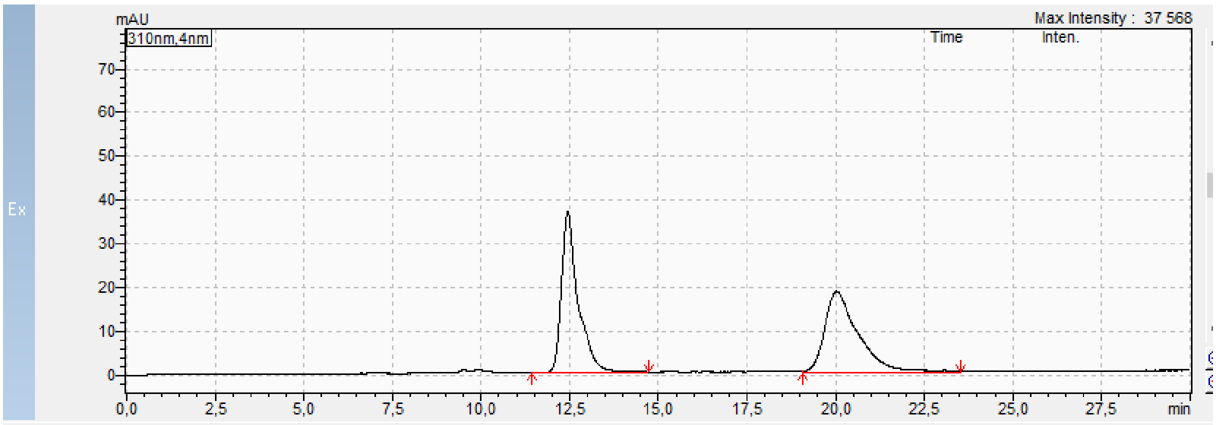

Results View - Peak Table

Peak Table | Compound | Group | Calibration Curve

Peak#	Ret. Time	Area	Height	Peak Start	Peak End	Mark	Conc.	Unit	Area%
1	12,445	1200812	36901	11,435	14,731	M	49,249		49,249
2	20,030	1237419	18584	19,072	23,531	M	50,751		50,751
Total		2438230	55485				100,000		100,000

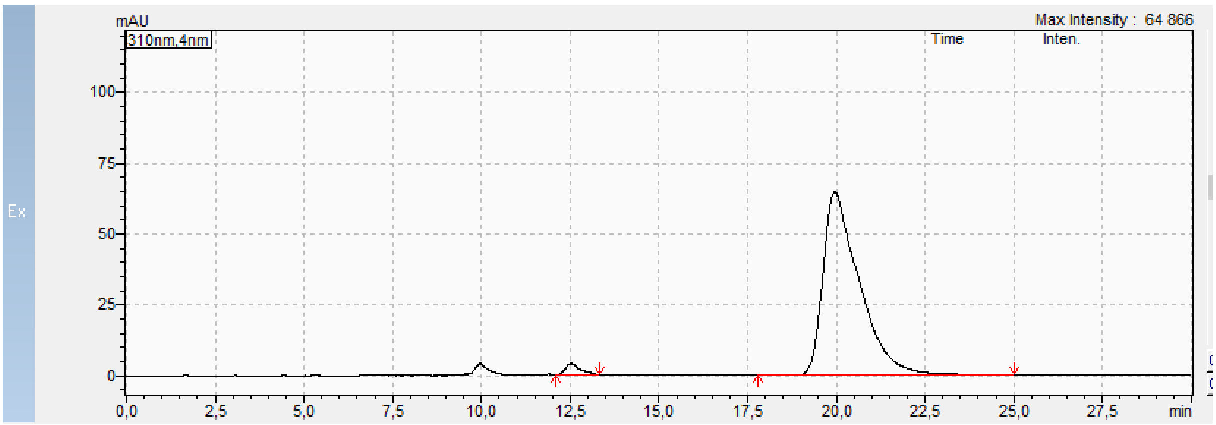

Results View - Peak Table

Peak Table | Compound | Group | Calibration Curve

Peak#	Ret. Time	Area	Height	Peak Start	Peak End	Mark	Conc.	Unit	Area%
1	12,523	131823	4113	12,075	13,323		2,792		2,792
2	19,959	4589910	64591	17,781	25,024	M	97,208		97,208
Total		4721733	68704				100,000		100,000

for **3b**: *er* = 97:3 (*ee* = 94%)

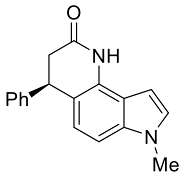

Conditions: IA column
mobile phase: *n*-heptane/*i*-PrOH – 80:20
λ = 310 nm, V = 1.0 ml/min, t = 25 °C
for *ent*-**3b**: t_R = 12.5 min (major), t_R = 20.0 min (minor)

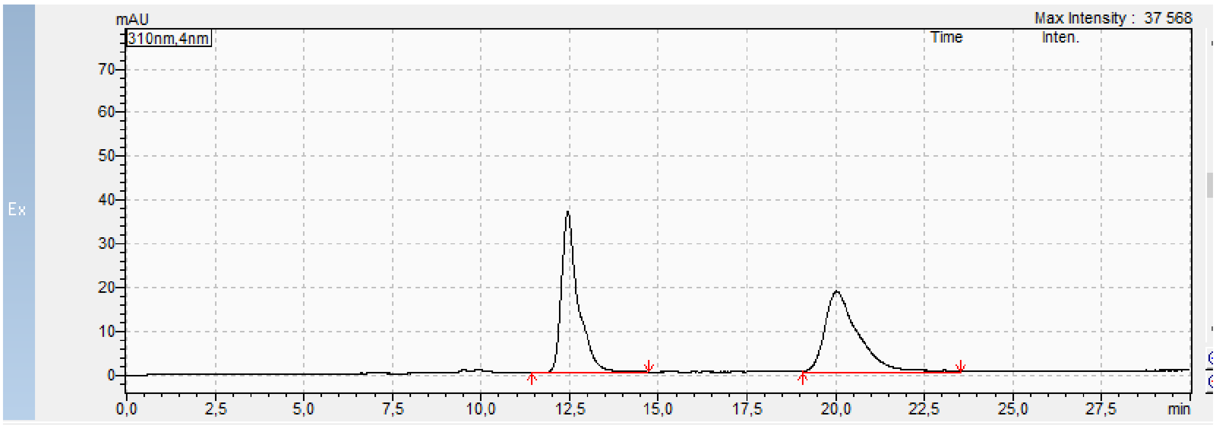

Results View - Peak Table

Peak Table | Compound | Group | Calibration Curve

Peak#	Ret. Time	Area	Height	Peak Start	Peak End	Mark	Conc.	Unit	Area%
1	12,445	1200812	36901	11,435	14,731	M	49,249		49,249
2	20,030	1237419	18584	19,072	23,531	M	50,751		50,751
Total		2438230	55485				100,000		100,000

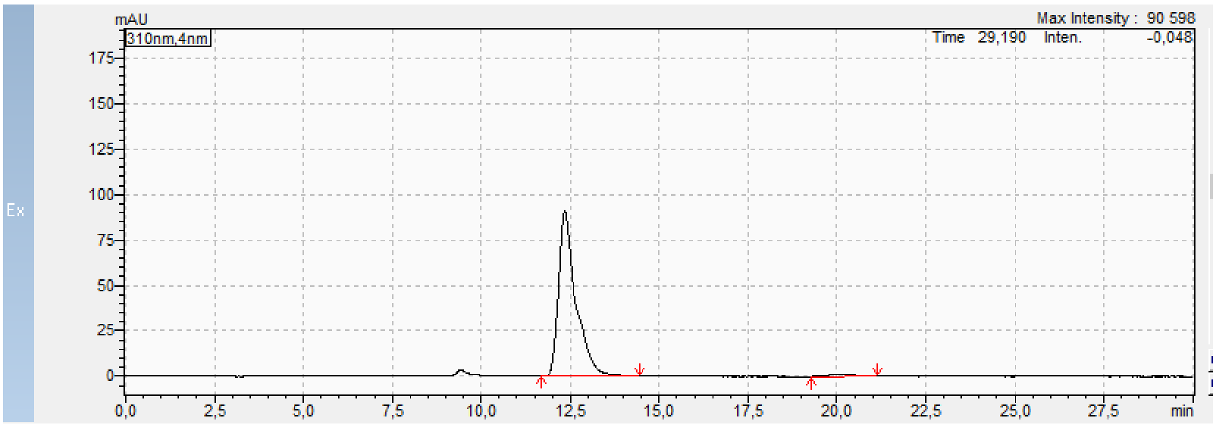

Results View - Peak Table

Peak Table | Compound | Group | Calibration Curve

Peak#	Ret. Time	Area	Height	Peak Start	Peak End	Mark	Conc.	Unit	Area%
1	12,354	3047031	90506	11,680	14,443		97,724		97,724
2	20,150	70958	1314	19,296	21,131		2,276		2,276
Total		3117990	91820				100,000		100,000

for *ent*-**3b**: *er* = 98:2 (*ee* = 96%)

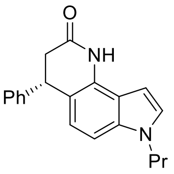

Conditions: IA column
mobile phase: *n*-heptane/*i*-PrOH – 60:40
λ = 310 nm, V = 1.0 ml/min, t = 25 °C
for **3c**: t_R = 8.3 min (minor), t_R = 11.9 min (major)

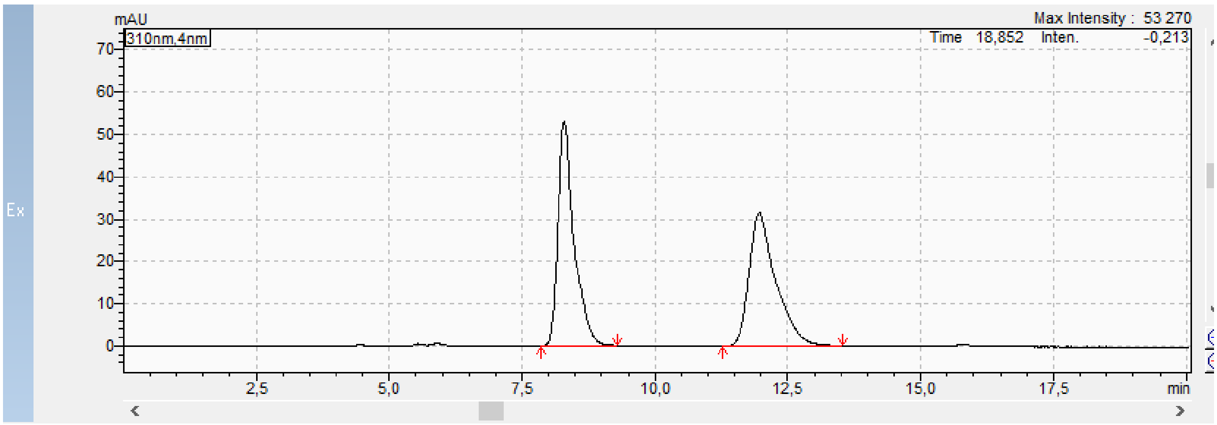

Results View - Peak Table

Peak Table | Compound | Group | Calibration Curve

Peak#	Ret. Time	Area	Height	Peak Start	Peak End	Mark	Conc.	Unit	Area%
1	8,293	1122935	53301	7,861	9,291		50,210		50,210
2	11,964	1113556	31597	11,264	13,547	S	49,790		49,790
Total		2236491	84897				100,000		100,000

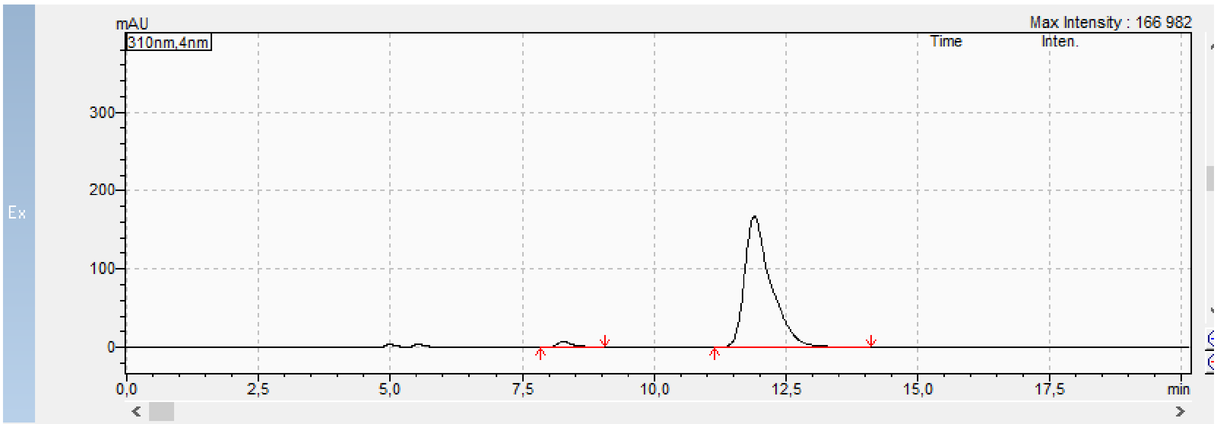

Results View - Peak Table

Peak Table | Compound | Group | Calibration Curve

Peak#	Ret. Time	Area	Height	Peak Start	Peak End	Mark	Conc.	Unit	Area%
1	8,281	165898	7825	7,851	9,077		2,760		2,760
2	11,901	5844071	167150	11,147	14,123		97,240		97,240
Total		6009969	174976				100,000		100,000

for **3c**: *er* = 97:3 (*ee* = 95%)

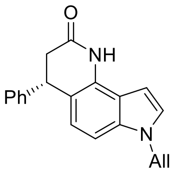

Conditions: IA column
mobile phase: *n*-heptane/*i*-PrOH – 60:40
λ = 222 nm, V = 1.0 ml/min, t = 25 °C
for **3d**: t_R = 8.7 min (minor), t_R = 12.3 min (major)

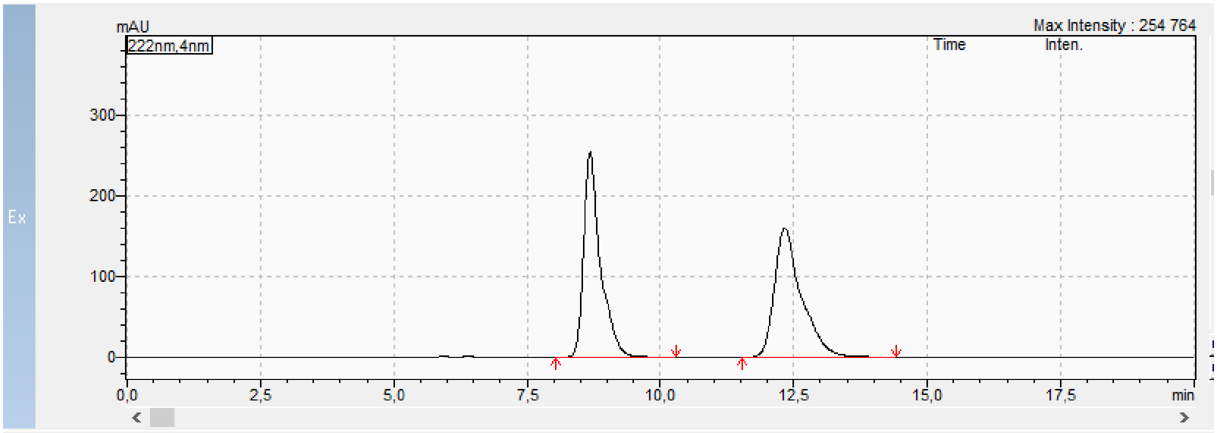

Results View - Peak Table

Peak Table | Compound | Group | Calibration Curve

Peak#	Ret. Time	Area	Height	Peak Start	Peak End	Mark	Conc.	Unit	Area%
1	8,684	5560004	254520	8,021	10,283		49,817		49,817
2	12,330	5600843	159948	11,531	14,421		50,183		50,183
Total		11160847	414468				100,000		100,000

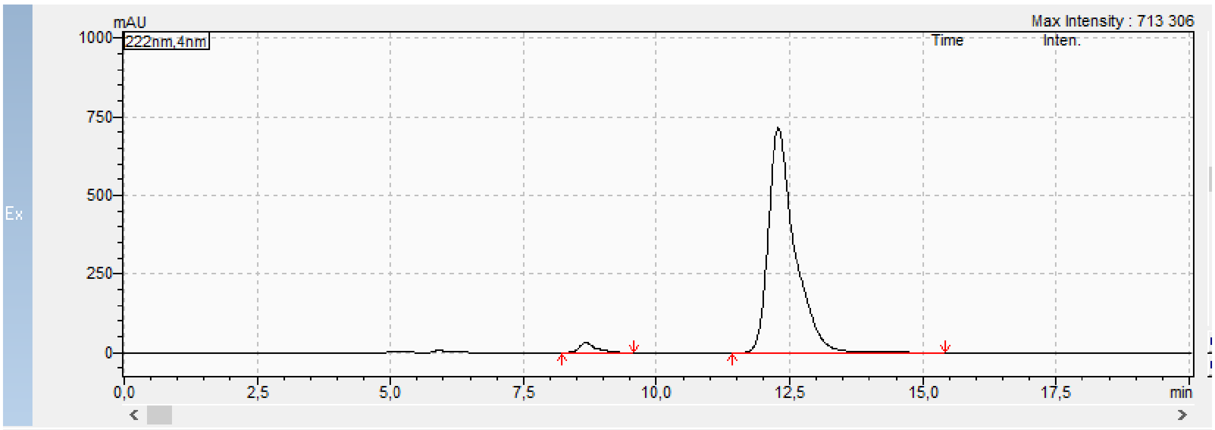

Results View - Peak Table

Peak Table | Compound | Group | Calibration Curve

Peak#	Ret. Time	Area	Height	Peak Start	Peak End	Mark	Conc.	Unit	Area%
1	8,671	675534	31194	8,235	9,568		2,692		2,692
2	12,289	24415323	712940	11,435	15,413		97,308		97,308
Total		25090857	744134				100,000		100,000

for **3d**: *er* = 97:3 (*ee* = 95%)

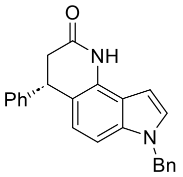

Conditions: IA column
mobile phase: *n*-heptane/*i*-PrOH – 60:40
λ = 300 nm, V = 1.0 ml/min, t = 25 °C
for **3e**: t_R = 11.1 min (minor), t_R = 15.3 min (major)

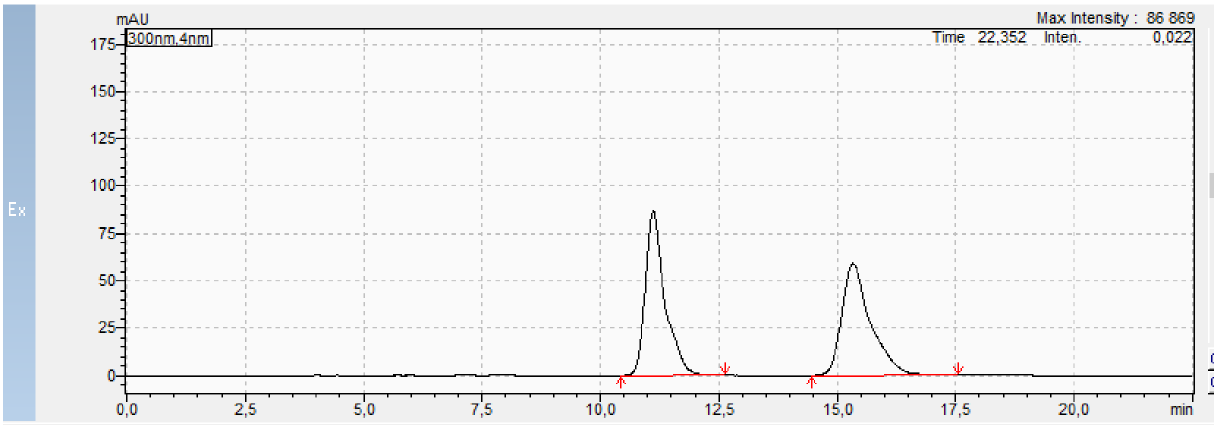

Results View - Peak Table

Peak Table | Compound | Group | Calibration Curve

Peak#	Ret. Time	Area	Height	Peak Start	Peak End	Mark	Conc.	Unit	Area%
1	11,116	2486020	86746	10,443	12,619		49,936		49,936
2	15,337	2492418	58859	14,453	17,557		50,064		50,064
Total		4978437	145604				100,000		100,000

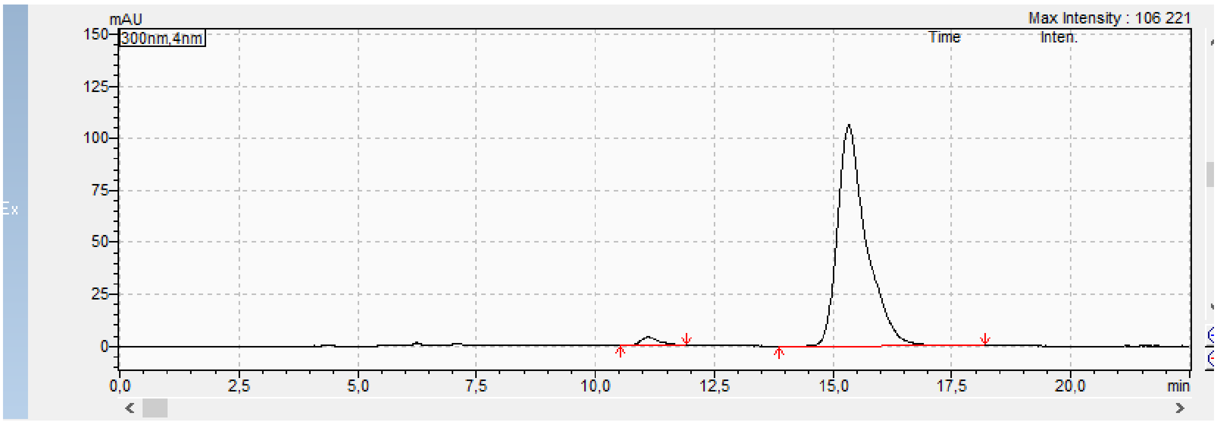

Results View - Peak Table

Peak Table | Compound | Group | Calibration Curve

Peak#	Ret. Time	Area	Height	Peak Start	Peak End	Mark	Conc.	Unit	Area%
1	11,118	115158	4098	10,539	11,915	M	2,505		2,505
2	15,330	4482717	106101	13,867	18,197	M	97,495		97,495
Total		4597874	110199				100,000		100,000

for **3e**: *er* = 97:3 (*ee* = 95%)

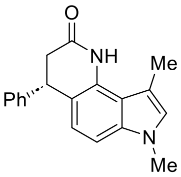

Conditions: Lux Amylose-1 column
mobile phase: *n*-heptane/*i*-PrOH – 80:20
λ= 227 nm, V = 1.0 ml/min, t = 25 °C
for **3f**: t_R = 11.9 min (major), t_R = 13.1 min (minor)

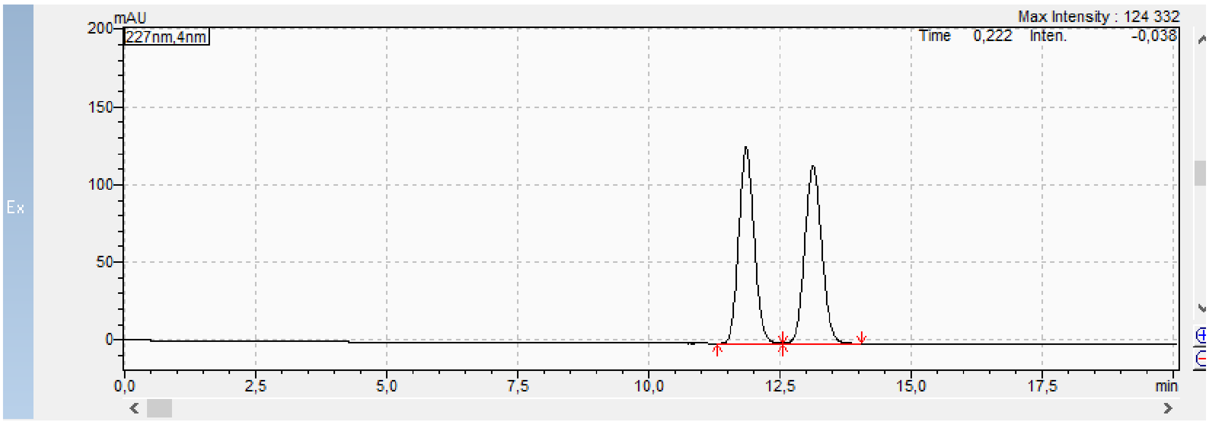

Results View - Peak Table

Peak Table | Compound | Group | Calibration Curve

Peak#	Ret. Time	Area	Height	Peak Start	Peak End	Mark	Conc.	Unit	Area%
1	11,862	2585145	126405	11,317	12,565		49,967		49,967
2	13,138	2588523	114679	12,565	14,059	V	50,033		50,033
Total		5173668	241084				100,000		100,000

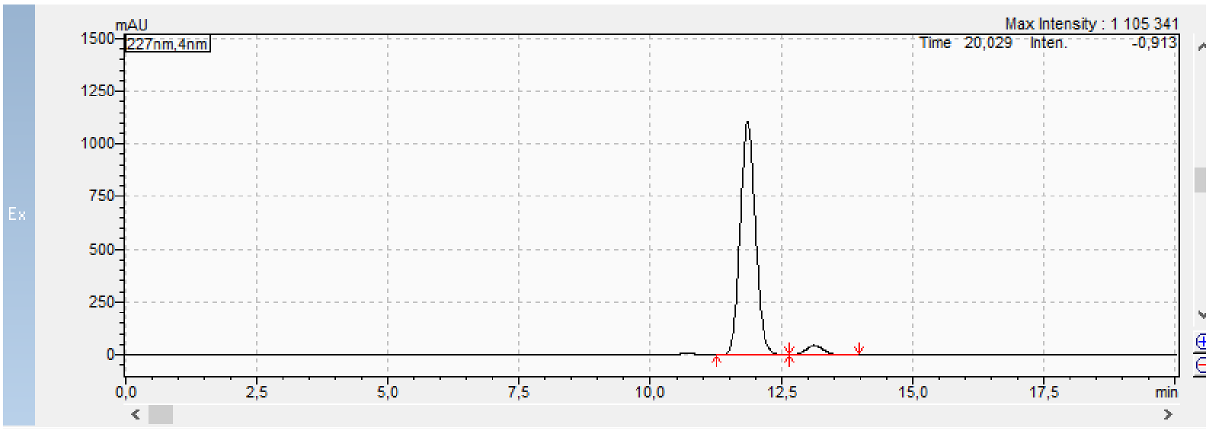

Results View - Peak Table

Peak Table | Compound | Group | Calibration Curve

Peak#	Ret. Time	Area	Height	Peak Start	Peak End	Mark	Conc.	Unit	Area%
1	11,857	22482592	1105835	11,264	12,661		95,523		95,523
2	13,130	1053688	46086	12,661	13,973	V	4,477		4,477
Total		23536280	1151920				100,000		100,000

for **3f**: *er* = 96:4 (*ee* = 91%)

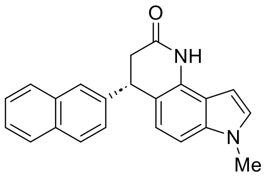

Conditions: IA column
mobile phase: *n*-heptane/*i*-PrOH – 60:40
λ = 225 nm, V = 1.0 ml/min, t = 25 °C
for **3g**: t_R = 9.9 min (minor), t_R = 20.5 min (major)

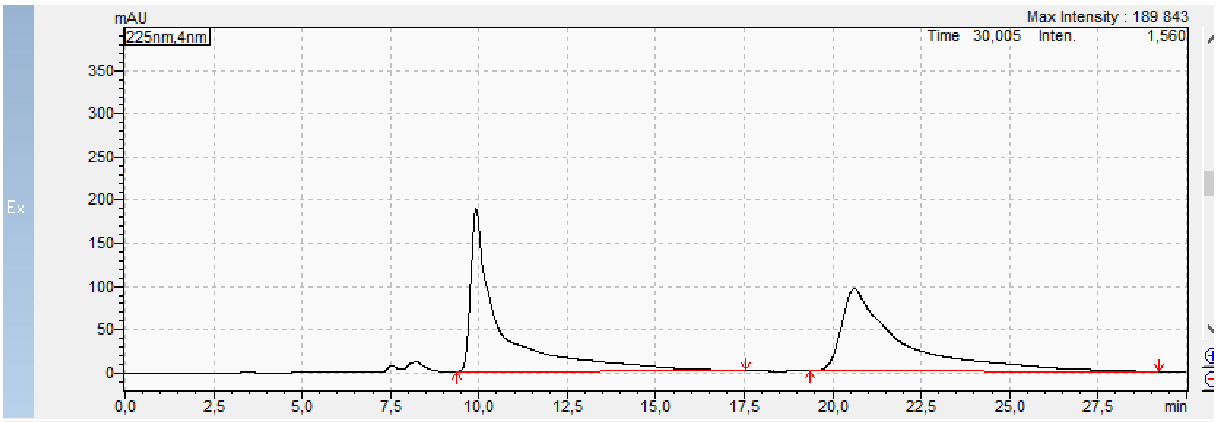

Results View - Peak Table

Peak Table | Compound | Group | Calibration Curve

Peak#	Ret. Time	Area	Height	Peak Start	Peak End	Mark	Conc.	Unit	Area%
1	9,921	10674623	188042	9,355	17,525	M	49,471		49,471
2	20,611	10902733	95213	19,339	29,205		50,529		50,529
Total		21577356	283256				100,000		100,000

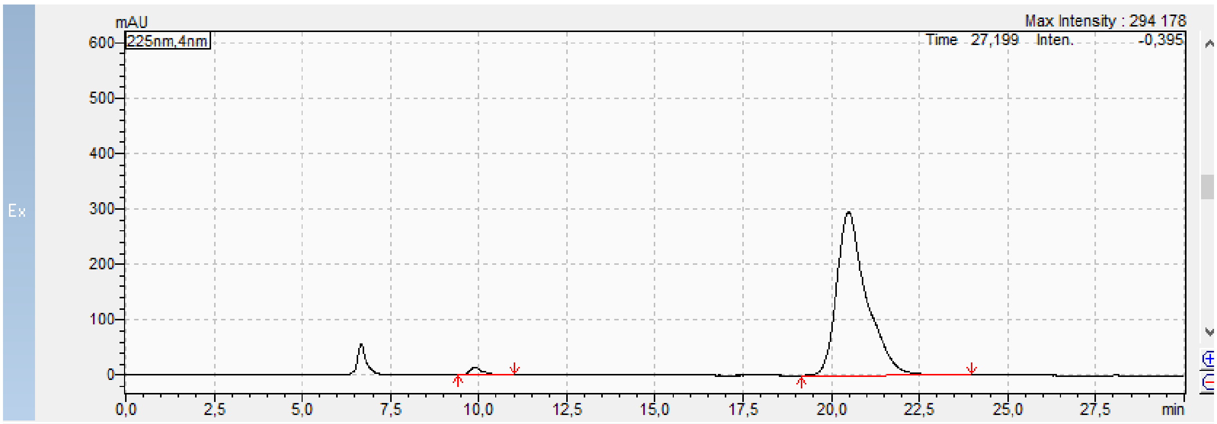

Results View - Peak Table

Peak Table | Compound | Group | Calibration Curve

Peak#	Ret. Time	Area	Height	Peak Start	Peak End	Mark	Conc.	Unit	Area%
1	9,887	393580	14796	9,397	11,008		2,211		2,211
2	20,490	17407155	294471	19,157	24,000		97,789		97,789
Total		17800735	309267				100,000		100,000

for **3g**: *er* = 98:2 (*ee* = 96%)

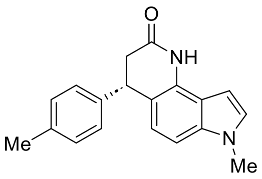

Conditions: IA column
mobile phase: *n*-heptane/*i*-PrOH – 60:40
λ = 223 nm, V = 1.0 ml/min, t = 25 °C
for **3h**: t_R = 7.6 min (minor), t_R = 15.6 min (major)

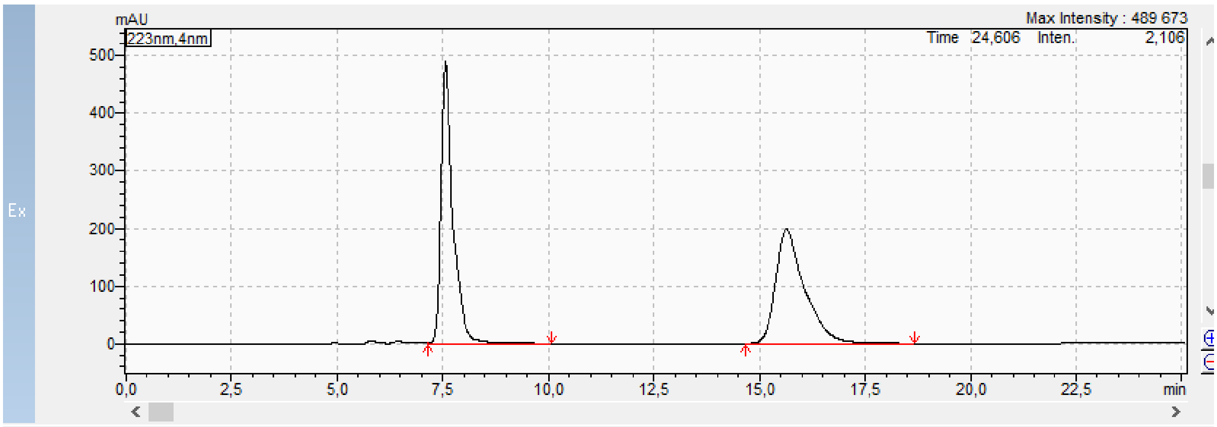

Results View - Peak Table

Peak Table | Compound | Group | Calibration Curve

Peak#	Ret. Time	Area	Height	Peak Start	Peak End	Mark	Conc.	Unit	Area%
1	7,563	9667719	489355	7,136	10,080		50,707		50,707
2	15,643	9397950	197943	14,667	18,667		49,293		49,293
Total		19065669	687298				100,000		100,000

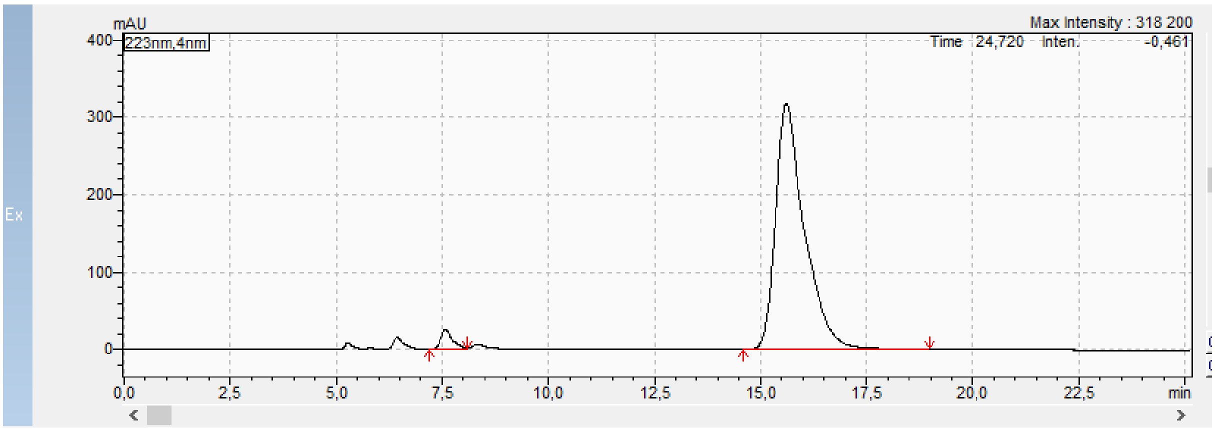

Results View - Peak Table

Peak Table | Compound | Group | Calibration Curve

Peak#	Ret. Time	Area	Height	Peak Start	Peak End	Mark	Conc.	Unit	Area%
1	7,572	501037	25598	7,200	8,096		3,214		3,214
2	15,609	15086734	318240	14,592	18,997		96,786		96,786
Total		15587772	343838				100,000		100,000

for **3h**: *er* = 97:3 (*ee* = 94%)

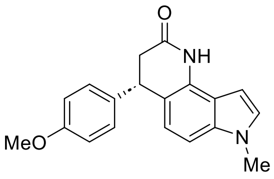

Conditions: IA column
mobile phase: *n*-heptane/*i*-PrOH – 80:20
λ = 223 nm, V = 1.0 ml/min, t = 25 °C
for **3i**: t_R = 16.5 min (minor), t_R = 36.3 min (major)

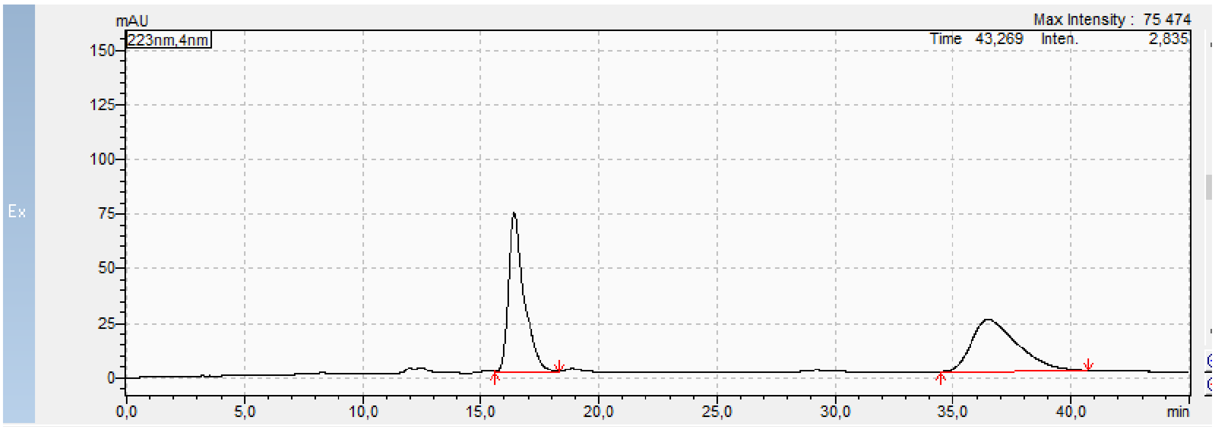

Results View - Peak Table

Peak Table | Compound | Group | Calibration Curve

Peak#	Ret. Time	Area	Height	Peak Start	Peak End	Mark	Conc.	Unit	Area%
1	16,426	3410172	73185	15,595	18,304		51,541		51,541
2	36,492	3206198	23902	34,475	40,747	S	48,459		48,459
Total		6616371	97087				100,000		100,000

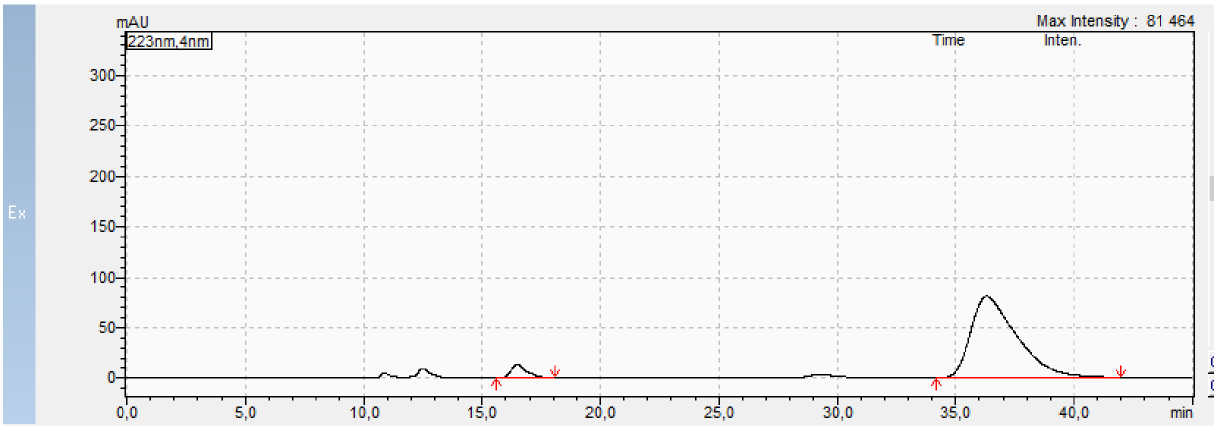

Results View - Peak Table

Peak Table | Compound | Group | Calibration Curve

Peak#	Ret. Time	Area	Height	Peak Start	Peak End	Mark	Conc.	Unit	Area%
1	16,481	634039	13688	15,616	18,101		5,466		5,466
2	36,317	10965702	81335	34,187	41,952		94,534		94,534
Total		11599742	95024				100,000		100,000

for **3i**: *er* = 95:5 (*ee* = 89%)

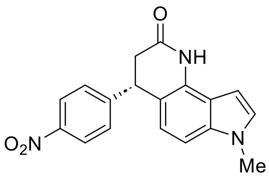

Conditions: IA column
mobile phase: *n*-heptane/*i*-PrOH – 50:50
λ = 289 nm, V = 1.0 ml/min, t = 25 °C
for **3j**: t_R = 12.5 min (minor), t_R = 25.2 min (major)

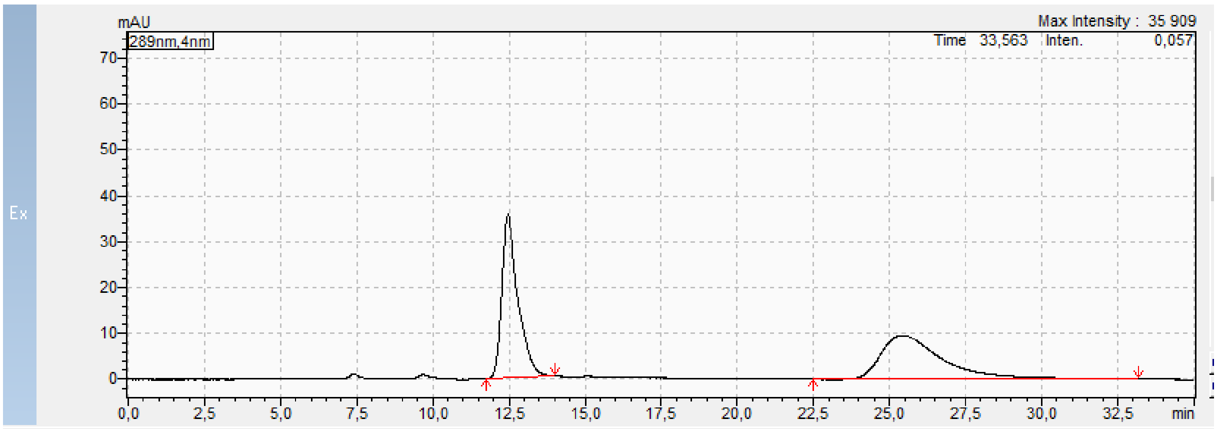

Results View - Peak Table

Peak Table | Compound | Group | Calibration Curve

Peak#	Ret. Time	Area	Height	Peak Start	Peak End	Mark	Conc.	Unit	Area%
1	12,468	1313449	35663	11,765	14,005		49,736		49,736
2	25,399	1327400	9564	22,485	33,163	M	50,264		50,264
Total		2640849	45227				100,000		100,000

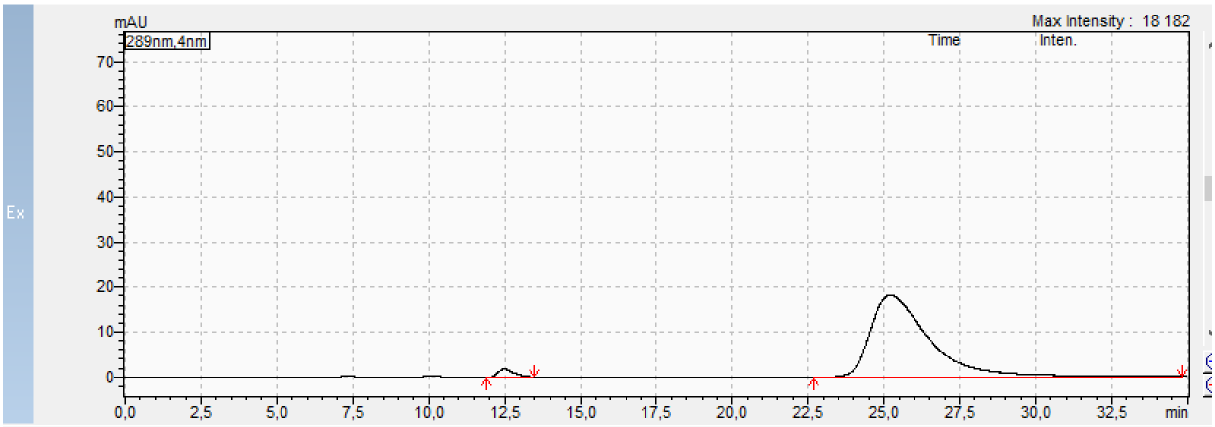

Results View - Peak Table

Peak Table | Compound | Group | Calibration Curve

Peak#	Ret. Time	Area	Height	Peak Start	Peak End	Mark	Conc.	Unit	Area%
1	12,497	67677	1893	11,915	13,472		2,640		2,640
2	25,218	2495435	18214	22,677	34,816	M	97,360		97,360
Total		2563112	20107				100,000		100,000

for **3j**: *er* = 97:3 (*ee* = 95%)

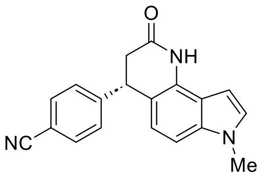

Conditions: IA column
mobile phase: *n*-heptane/*i*-PrOH – 50:50
λ = 227 nm, V = 1.0 ml/min, t = 25 °C
for **3k**: t_R = 9.1 min (minor), t_R = 15.9 min (major)

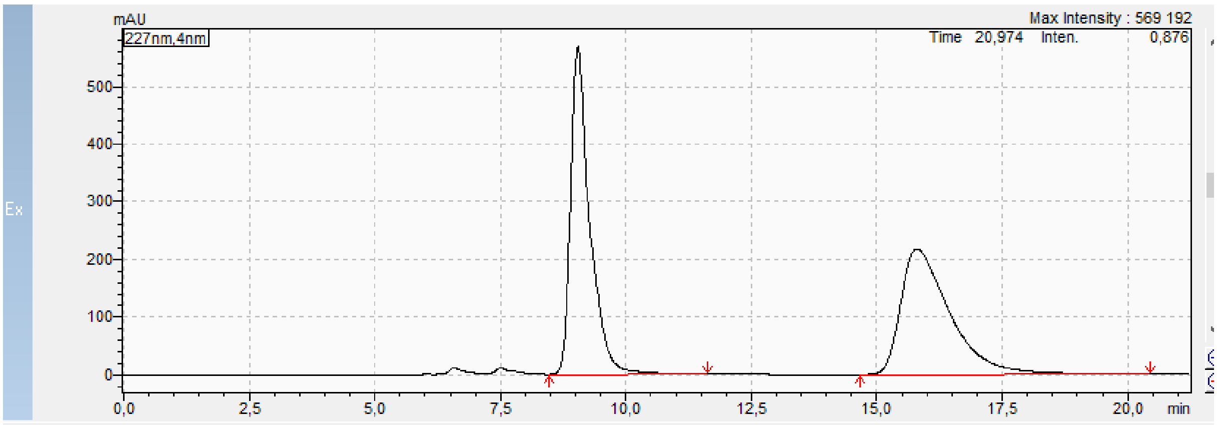

Results View - Peak Table

Peak Table | Compound | Group | Calibration Curve

Peak#	Ret. Time	Area	Height	Peak Start	Peak End	Mark	Conc.	Unit	Area%
1	9,045	15075935	568509	8,459	11,637		50,495		50,495
2	15,802	14780177	217568	14,677	20,437		49,505		49,505
Total		29856112	786077				100,000		100,000

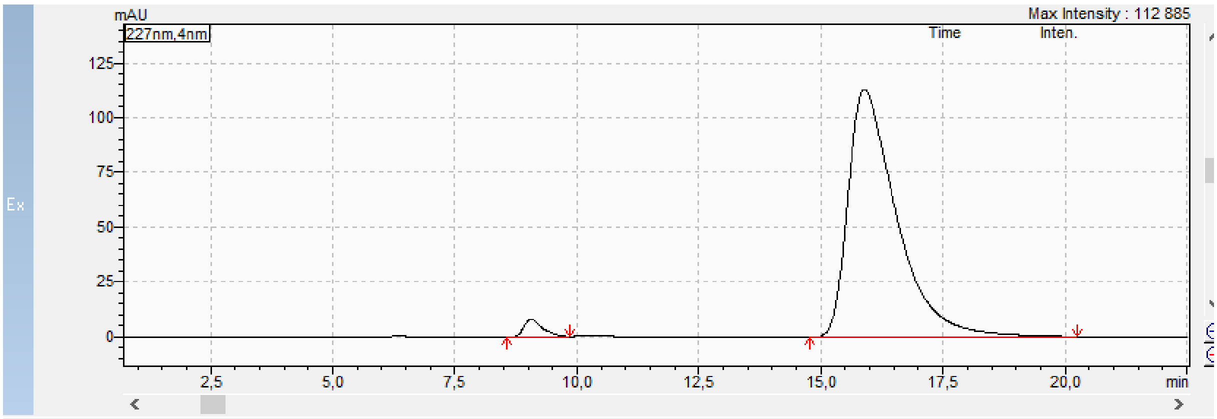

Results View - Peak Table

Peak Table | Compound | Group | Calibration Curve

Peak#	Ret. Time	Area	Height	Peak Start	Peak End	Mark	Conc.	Unit	Area%
1	9,063	228905	8315	8,565	9,845		2,832		2,832
2	15,893	7852658	113164	14,773	20,256		97,168		97,168
Total		8081563	121479				100,000		100,000

for **3k**: *er* = 97:3 (*ee* = 94%)

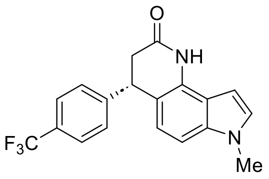

Conditions: IA column
mobile phase: *n*-heptane/*i*-PrOH – 60:40
λ = 221 nm, V = 1.0 ml/min, t = 25 °C
for **3l**: t_R = 7.0 min (minor), t_R = 10.8 min (major)

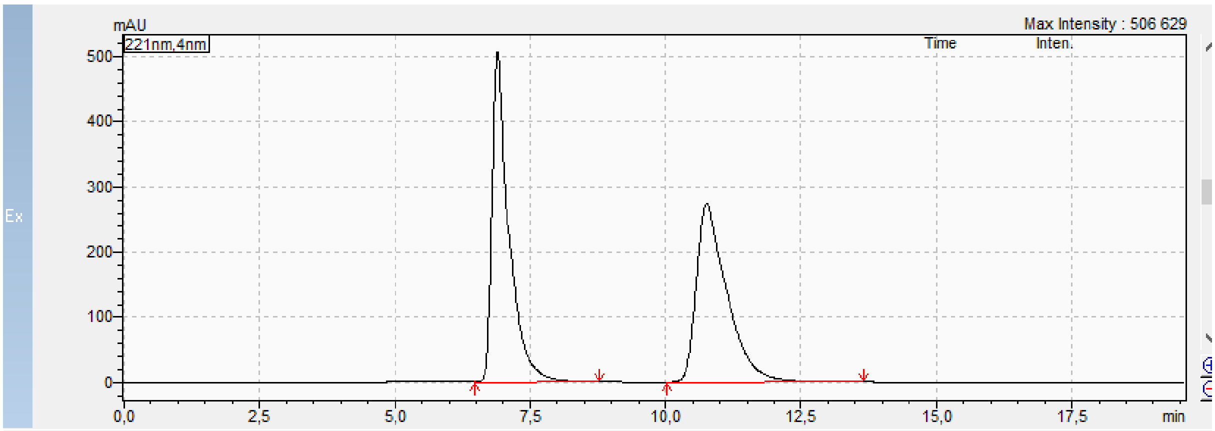

Results View - Peak Table

Peak Table | Compound | Group | Calibration Curve

Peak#	Ret. Time	Area	Height	Peak Start	Peak End	Mark	Conc.	Unit	Area%
1	6,895	10855648	506095	6,485	8,768		50,110		50,110
2	10,757	10808112	275128	10,016	13,643		49,890		49,890
Total		21663759	781222				100,000		100,000

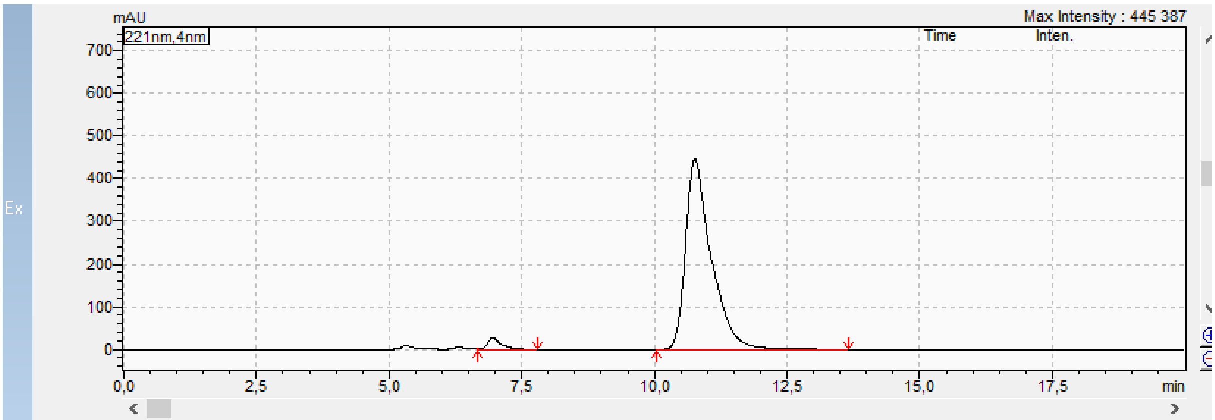

Results View - Peak Table

Peak Table | Compound | Group | Calibration Curve

Peak#	Ret. Time	Area	Height	Peak Start	Peak End	Mark	Conc.	Unit	Area%
1	6,956	468894	26455	6,677	7,808		3,019		3,019
2	10,765	15060548	445854	10,048	13,664		96,981		96,981
Total		15529443	472309				100,000		100,000

for **3l**: *er* = 97:3 (*ee* = 94%)

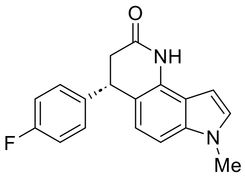

Conditions: IA column
mobile phase: *n*-heptane/*i*-PrOH – 60:40
λ = 223 nm, V = 1.0 ml/min, t = 25 °C
for **3m**: t_R = 8.6 min (minor), t_R = 12.7 min (major)

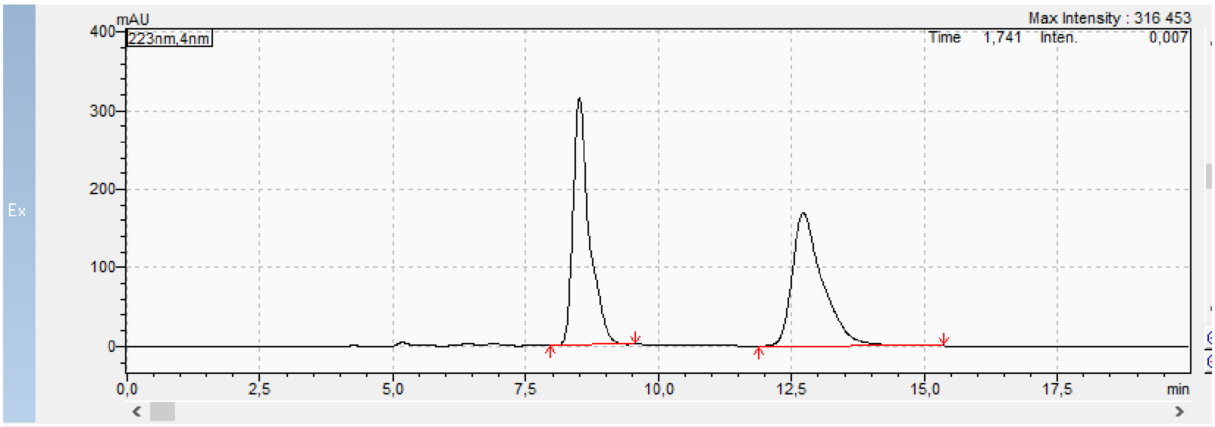

Results View - Peak Table

Peak Table | Compound | Group | Calibration Curve

Peak#	Ret. Time	Area	Height	Peak Start	Peak End	Mark	Conc.	Unit	Area%
1	8,508	6677936	315005	7,947	9,568	M	49,817		49,817
2	12,722	6726875	169353	11,893	15,360		50,183		50,183
Total		13404811	484358				100,000		100,000

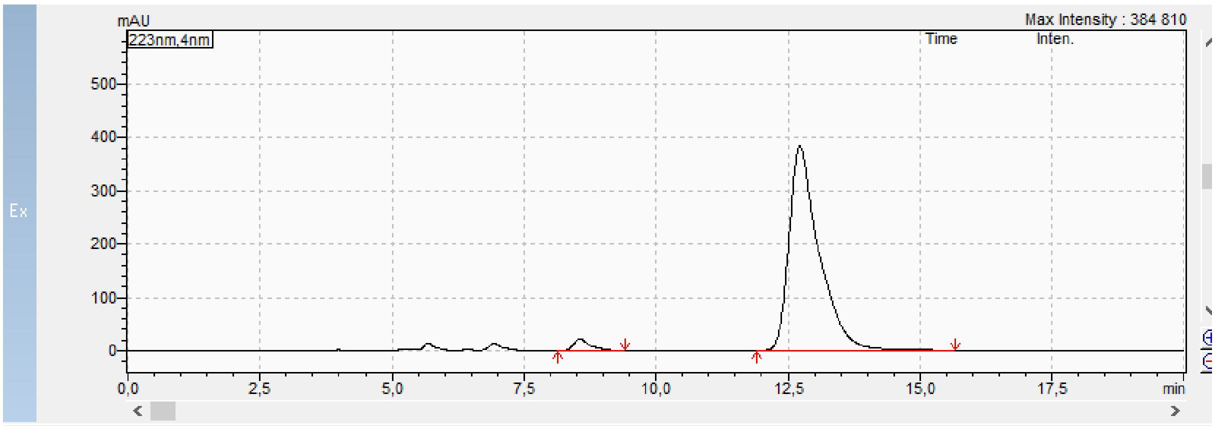

Results View - Peak Table

Peak Table | Compound | Group | Calibration Curve

Peak#	Ret. Time	Area	Height	Peak Start	Peak End	Mark	Conc.	Unit	Area%
1	8,561	477814	22131	8,128	9,419		3,059		3,059
2	12,724	15143042	384477	11,893	15,669		96,941		96,941
Total		15620856	406608				100,000		100,000

for **3m**: *er* = 97:3 (*ee* = 94%)

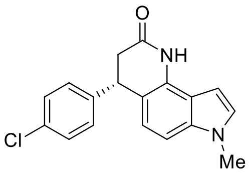

Conditions: IA column
mobile phase: *n*-heptane/*i*-PrOH – 80:20
λ = 233 nm, V = 1.0 ml/min, t = 25 °C
for **3n**: t_R = 13.5 min (minor), t_R = 30.2 min (major)

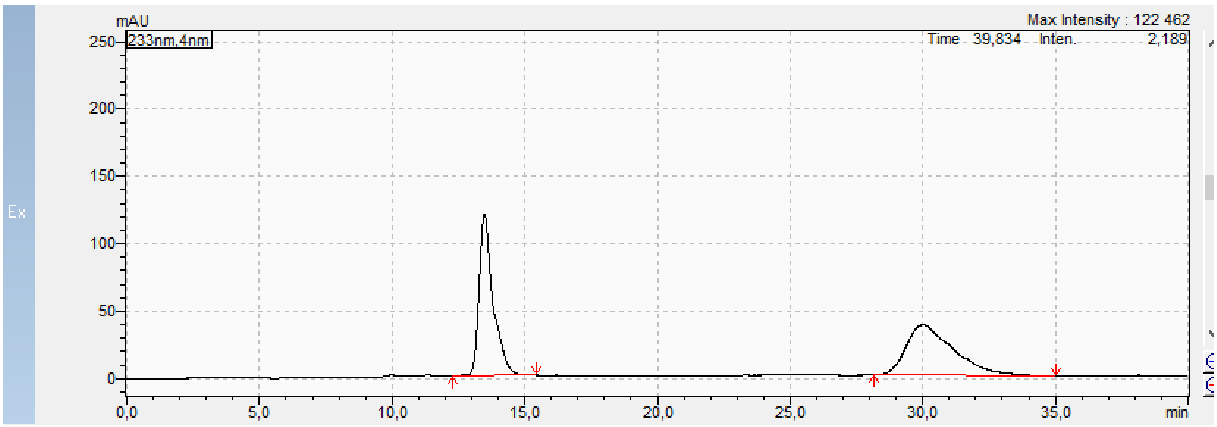

Results View - Peak Table

Peak Table | Compound | Group | Calibration Curve

Peak#	Ret. Time	Area	Height	Peak Start	Peak End	Mark	Conc.	Unit	Area%
1	13,484	4396525	120422	12,288	15,445		48,806		48,806
2	30,003	4611716	37333	28,181	35,029	M	51,194		51,194
Total		9008241	157755				100,000		100,000

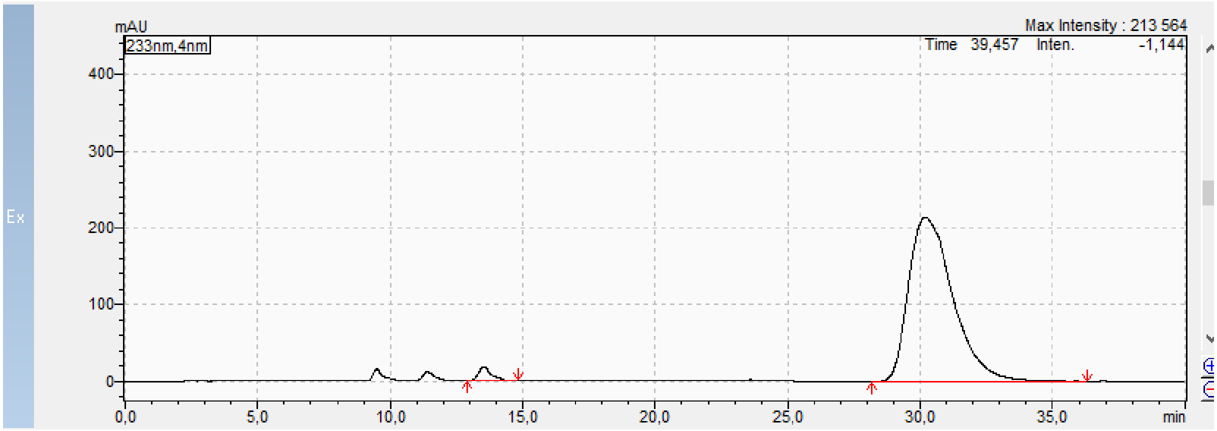

Results View - Peak Table

Peak Table | Compound | Group | Calibration Curve

Peak#	Ret. Time	Area	Height	Peak Start	Peak End	Mark	Conc.	Unit	Area%
1	13,517	653814	18628	12,907	14,848		2,546		2,546
2	30,195	25022716	214152	28,192	36,320	S	97,454		97,454
Total		25676530	232781				100,000		100,000

for **3n**: *er* = 97:3 (*ee* = 95%)

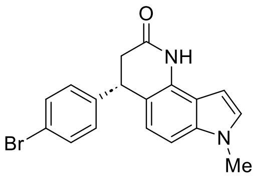

Conditions: IA column
mobile phase: *n*-heptane/*i*-PrOH – 60:40
λ = 233 nm, V = 1.0 ml/min, t = 25 °C
for **3o**: t_R = 8.6 min (minor), t_R = 17.4 min (major)

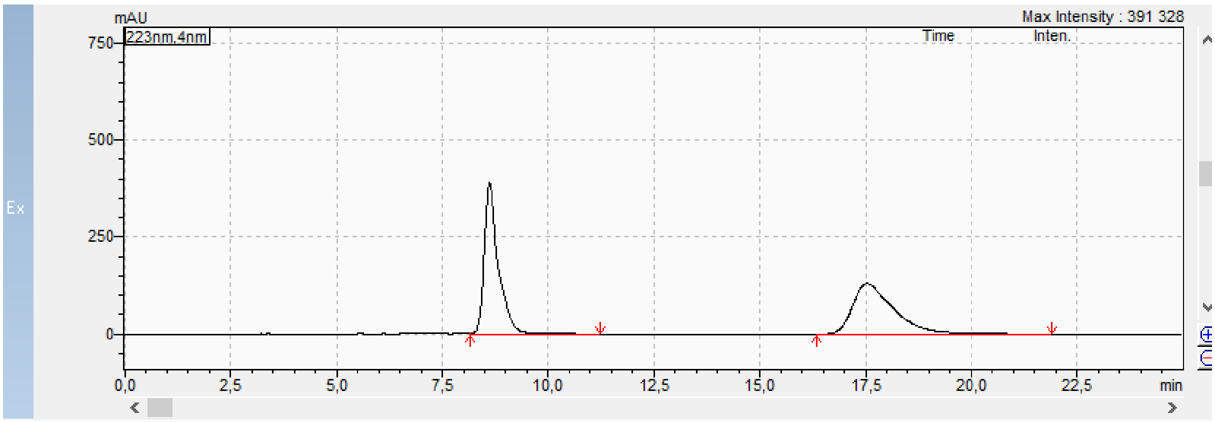

Results View - Peak Table

Peak Table | Compound | Group | Calibration Curve

Peak#	Ret. Time	Area	Height	Peak Start	Peak End	Mark	Conc.	Unit	Area%
1	8,614	9041433	391512	8,171	11,232	S	50,638		50,638
2	17,532	8813557	129817	16,341	21,899		49,362		49,362
Total		17854990	521329				100,000		100,000

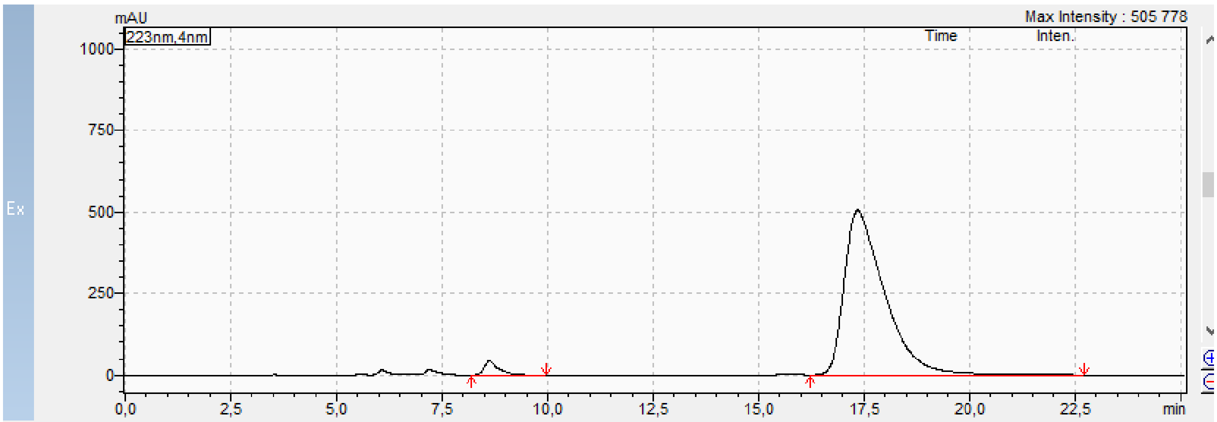

Results View - Peak Table

Peak Table | Compound | Group | Calibration Curve

Peak#	Ret. Time	Area	Height	Peak Start	Peak End	Mark	Conc.	Unit	Area%
1	8,626	1020758	43533	8,192	9,984		2,964		2,964
2	17,346	33416604	505726	16,224	22,709	S	97,036		97,036
Total		34437362	549259				100,000		100,000

for **3o**: *er* = 97:3 (*ee* = 94%)

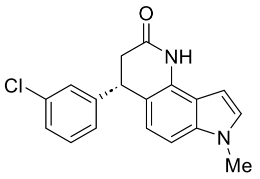

Conditions: IA column
mobile phase: *n*-heptane/*i*-PrOH – 60:40
λ = 222 nm, V = 1.0 ml/min, t = 25 °C
for **3p**: t_R = 8.2 min (minor), t_R = 9.7 min (major)

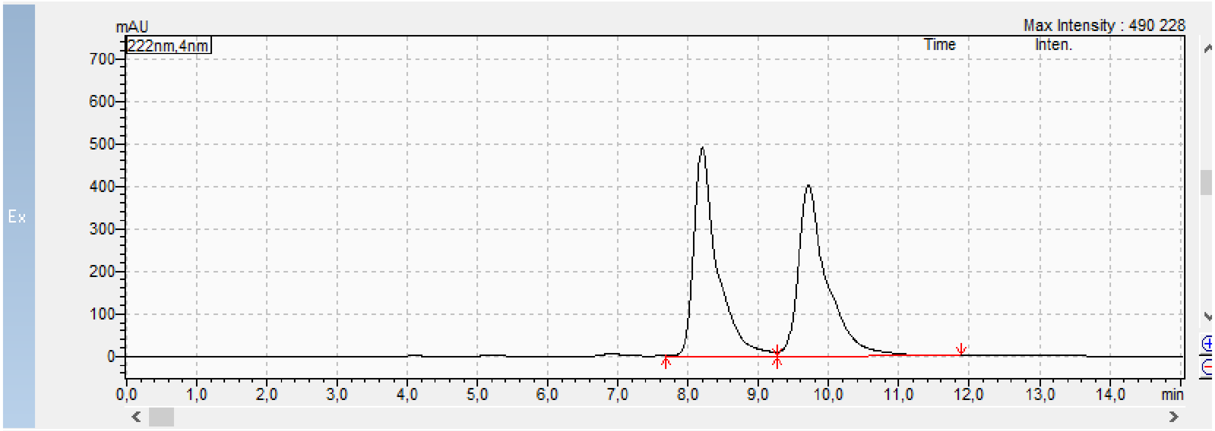

Results View - Peak Table

Peak Table | Compound | Group | Calibration Curve

Peak#	Ret. Time	Area	Height	Peak Start	Peak End	Mark	Conc.	Unit	Area%
1	8,198	11257258	489566	7,680	9,259		49,676		49,676
2	9,713	11404117	401297	9,259	11,883	V	50,324		50,324
Total		22661374	890863				100,000		100,000

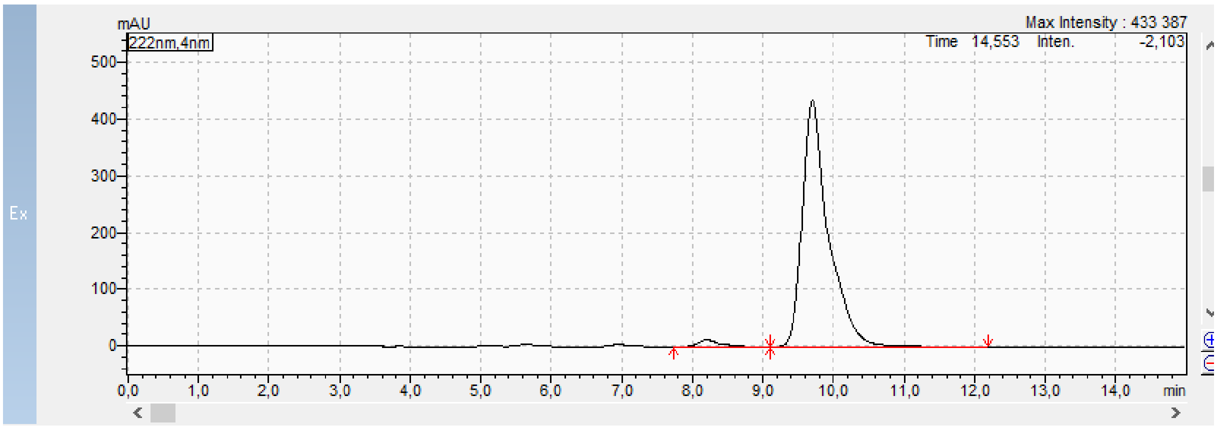

Results View - Peak Table

Peak Table | Compound | Group | Calibration Curve

Peak#	Ret. Time	Area	Height	Peak Start	Peak End	Mark	Conc.	Unit	Area%
1	8,200	261919	12201	7,723	9,099		2,276		2,276
2	9,700	11246021	434964	9,099	12,192		97,724		97,724
Total		11507940	447164				100,000		100,000

for **3p**: *er* = 98:2 (*ee* = 96%)

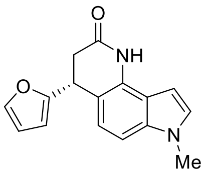

Conditions: IA column
mobile phase: *n*-heptane/*i*-PrOH – 60:40
λ = 296 nm, V = 1.0 ml/min, t = 25 °C
for **3q**: t_R = 7.9 min (minor), t_R = 12.0 min (major)

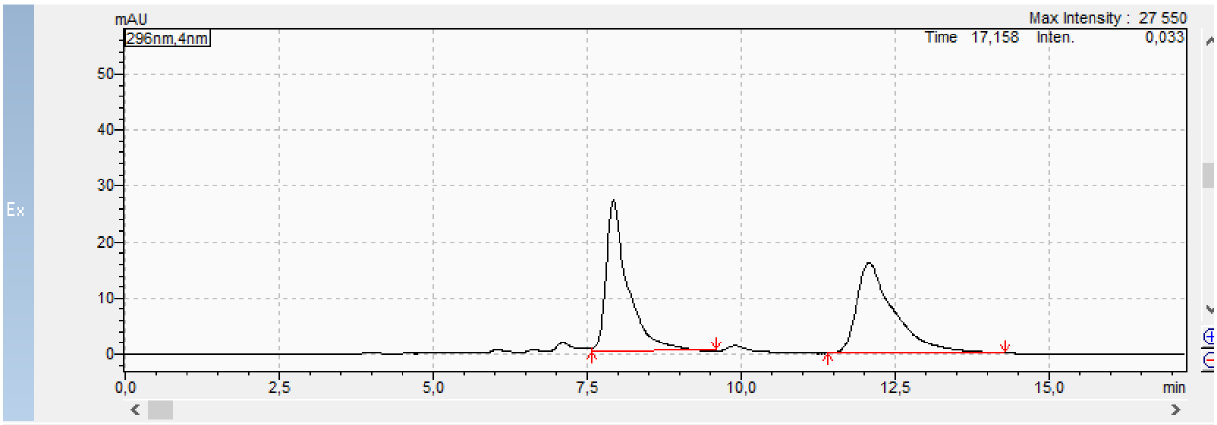

Results View - Peak Table

Peak Table | Compound | Group | Calibration Curve

Peak#	Ret. Time	Area	Height	Peak Start	Peak End	Mark	Conc.	Unit	Area%
1	7,921	663738	27115	7,563	9,589	M	49,406		49,406
2	12,070	679687	16126	11,392	14,272		50,594		50,594
Total		1343425	43241				100,000		100,000

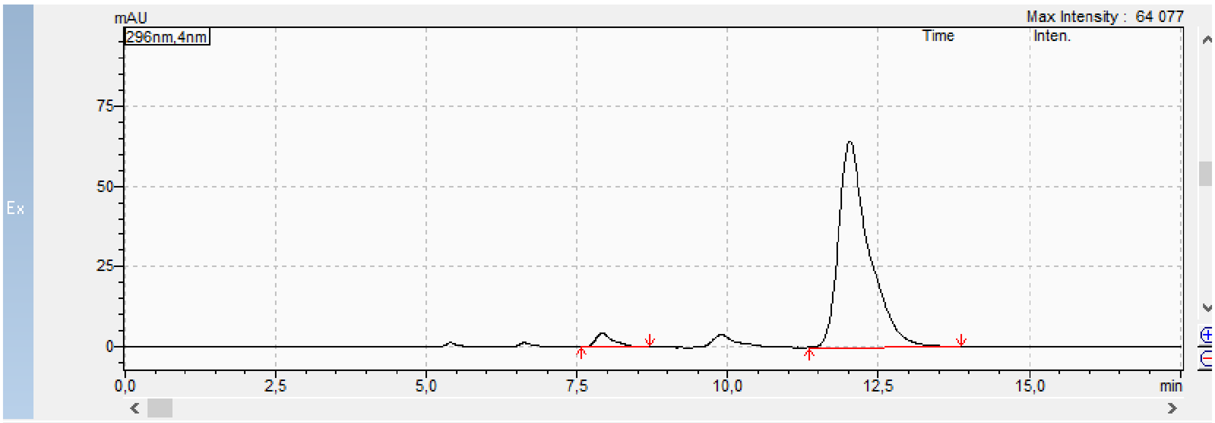

Results View - Peak Table

Peak Table | Compound | Group | Calibration Curve

Peak#	Ret. Time	Area	Height	Peak Start	Peak End	Mark	Conc.	Unit	Area%
1	7,915	91020	4276	7,563	8,715		3,901		3,901
2	12,022	2241954	64180	11,349	13,877		96,099		96,099
Total		2332974	68456				100,000		100,000

for **3q**: *er* = 96:4 (*ee* = 92%)

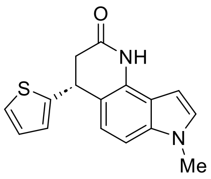

Conditions: IA column
mobile phase: *n*-heptane/*i*-PrOH – 60:40
λ= 309 nm, V = 1.0 ml/min, t = 25 °C
for **3r**: t_R = 8.2 min (minor), t_R = 16.1 min (major)

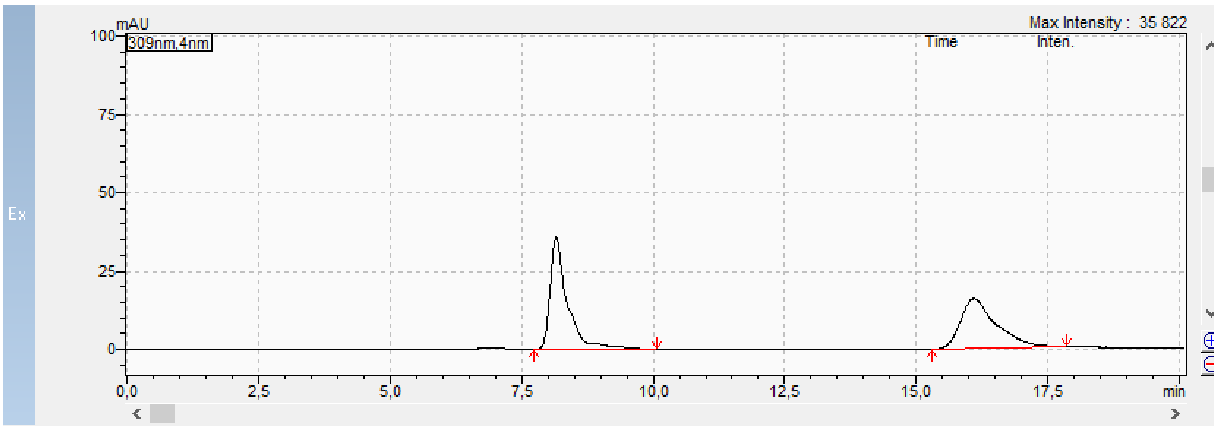

Results View - Peak Table

Peak Table | Compound | Group | Calibration Curve

Peak#	Ret. Time	Area	Height	Peak Start	Peak End	Mark	Conc.	Unit	Area%
1	8,158	827619	35865	7,723	10,080		52,237		52,237
2	16,090	756746	15951	15,285	17,856		47,763		47,763
Total		1584365	51817				100,000		100,000

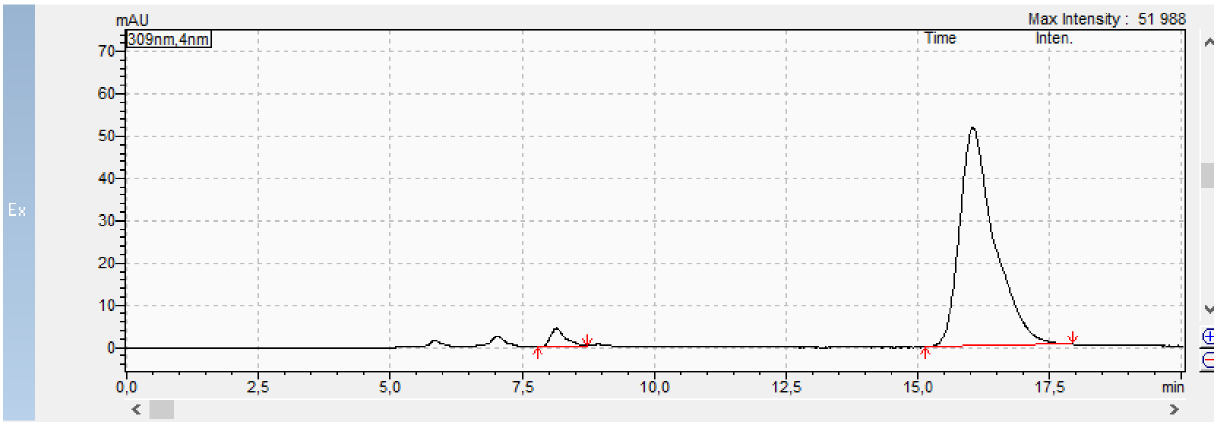

Results View - Peak Table

Peak Table | Compound | Group | Calibration Curve

Peak#	Ret. Time	Area	Height	Peak Start	Peak End	Mark	Conc.	Unit	Area%
1	8,150	90006	4410	7,787	8,725		3,642		3,642
2	16,046	2381619	51694	15,125	17,931		96,358		96,358
Total		2471625	56104				100,000		100,000

for **3r**: *er* = 96:4 (*ee* = 93%)

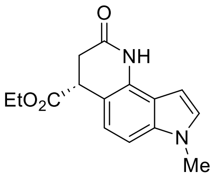

Conditions: IA column
mobile phase: *n*-heptane/*i*-PrOH – 60:40
λ= 217 nm, V = 1.0 ml/min, t = 25 °C
for **3s**: t_R = 7.9 min (minor), t_R = 17.2 min (major)

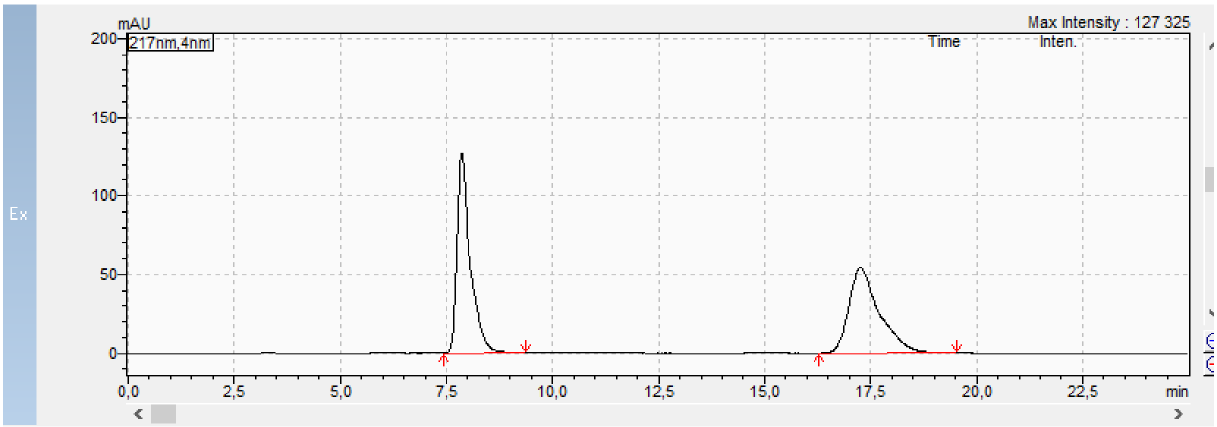

Results View - Peak Table

Peak Table | Compound | Group | Calibration Curve

Peak#	Ret. Time	Area	Height	Peak Start	Peak End	Mark	Conc.	Unit	Area%
1	7,865	2728261	127131	7,435	9,365		50,219		50,219
2	17,263	2704434	54298	16,288	19,520	S	49,781		49,781
Total		5432695	181428				100,000		100,000

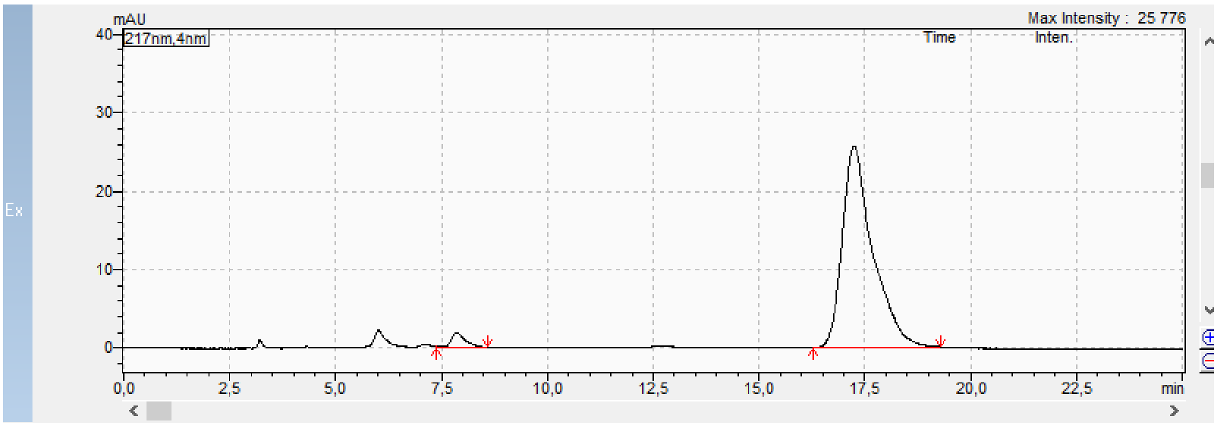

Results View - Peak Table

Peak Table | Compound | Group | Calibration Curve

Peak#	Ret. Time	Area	Height	Peak Start	Peak End	Mark	Conc.	Unit	Area%
1	7,863	41263	1900	7,381	8,597		3,109		3,109
2	17,243	1285968	25722	16,267	19,296	S	96,891		96,891
Total		1327231	27622				100,000		100,000

for **3s**: *er* = 97:3 (*ee* = 94%)

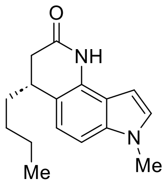

Conditions: IA column
mobile phase: *n*-heptane/*i*-PrOH – 60:40
λ= 310 nm, V = 1.0 ml/min, t = 25 °C
for **3t**: t_R = 5.6 min (minor), t_R = 6.9 min (major)

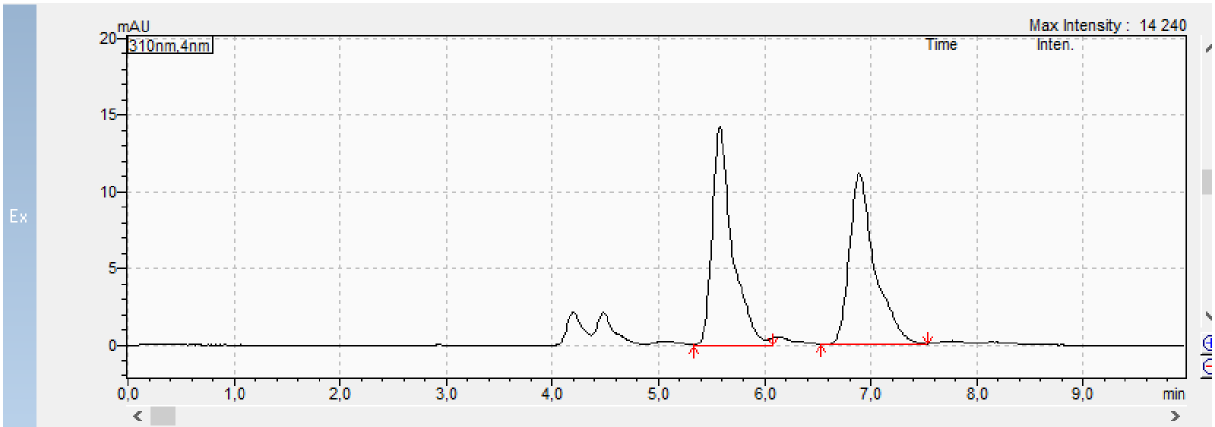

Results View - Peak Table

Peak Table | Compound | Group | Calibration Curve

Peak#	Ret. Time	Area	Height	Peak Start	Peak End	Mark	Conc.	Unit	Area%
1	5,578	189749	14243	5,333	6,069	M	50,620		50,620
2	6,892	185103	11168	6,528	7,531		49,380		49,380
Total		374851	25411				100,000		100,000

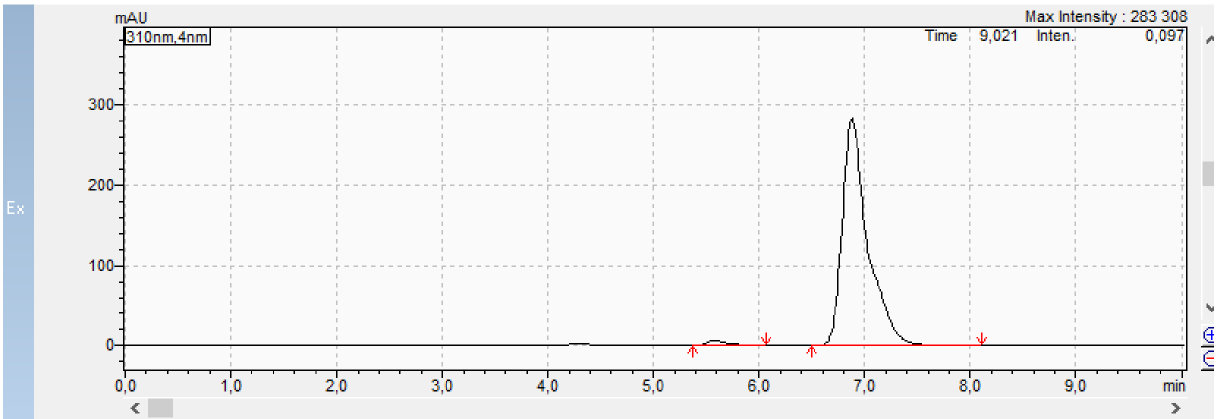

Results View - Peak Table

Peak Table | Compound | Group | Calibration Curve

Peak#	Ret. Time	Area	Height	Peak Start	Peak End	Mark	Conc.	Unit	Area%
1	5,577	83289	6359	5,365	6,059		1,731		1,731
2	6,880	4728145	283032	6,496	8,107		98,269		98,269
Total		4811435	289391				100,000		100,000

for **3t**: *er* = 98:2 (*ee* = 97%)

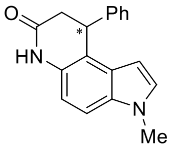

Conditions: IA column
mobile phase: *n*-heptane/*i*-PrOH – 80:20
λ= 256 nm, V = 1.0 ml/min, t = 25 °C
for **3u**: t_R = 9.4 min (minor), t_R = 10.6 min (major)

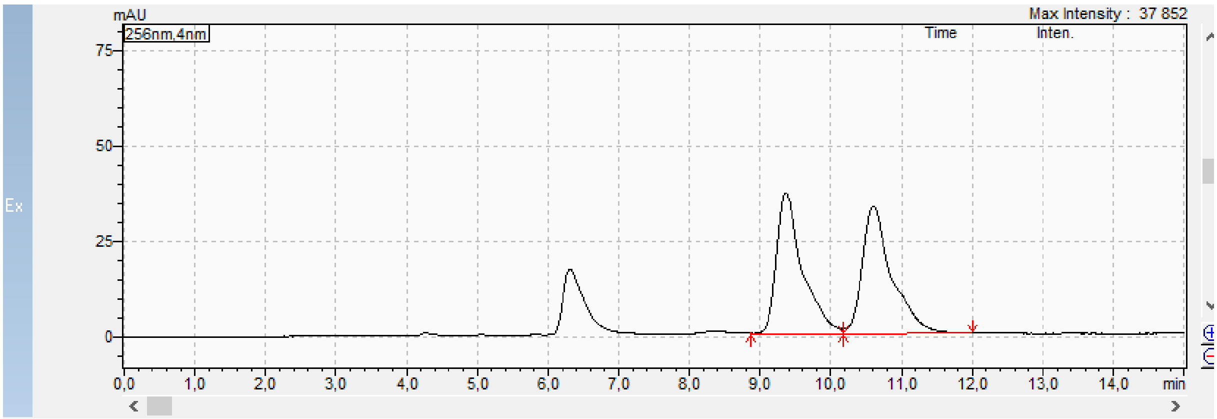

Results View - Peak Table

Peak Table | Compound | Group | Calibration Curve

Peak#	Ret. Time	Area	Height	Peak Start	Peak End	Mark	Conc.	Unit	Area%
1	9,364	936922	36870	8,875	10,176		49,476		49,476
2	10,602	956772	33179	10,176	12,000	V	50,524		50,524
Total		1893694	70049				100,000		100,000

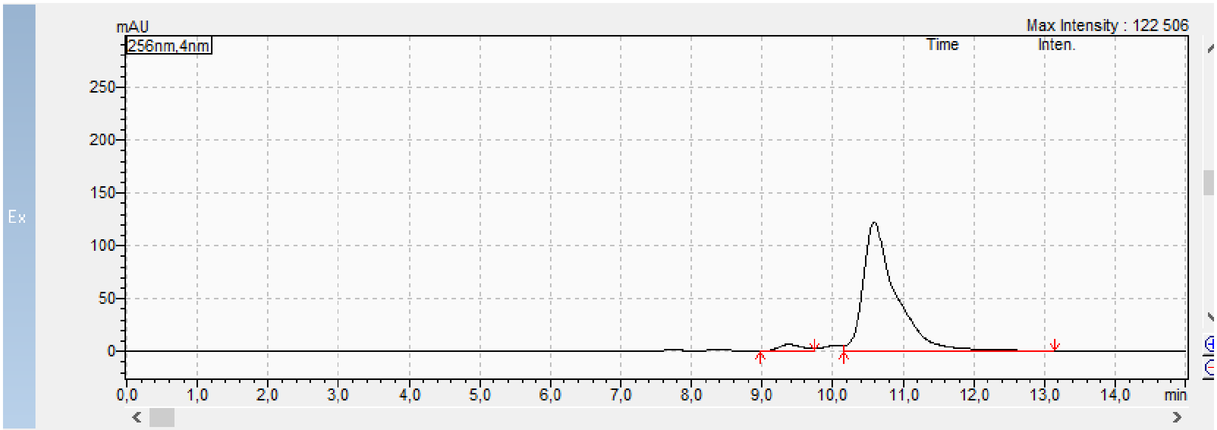

Results View - Peak Table

Peak Table | Compound | Group | Calibration Curve

Peak#	Ret. Time	Area	Height	Peak Start	Peak End	Mark	Conc.	Unit	Area%
1	9,377	154257	6552	8,971	9,749		3,804		3,804
2	10,592	3901288	122054	10,155	13,152		96,196		96,196
Total		4055545	128606				100,000		100,000

for **3u**: *er* = 96:4 (*ee* = 92%)

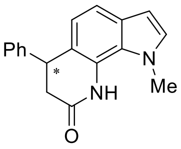

Conditions: IB column
mobile phase: *n*-heptane/*i*-PrOH – 80:20
λ= 234 nm, V = 1.0 ml/min, t = 25 °C
for **3v**: t_R = 11.0 min (major), t_R = 14.0 min (minor)

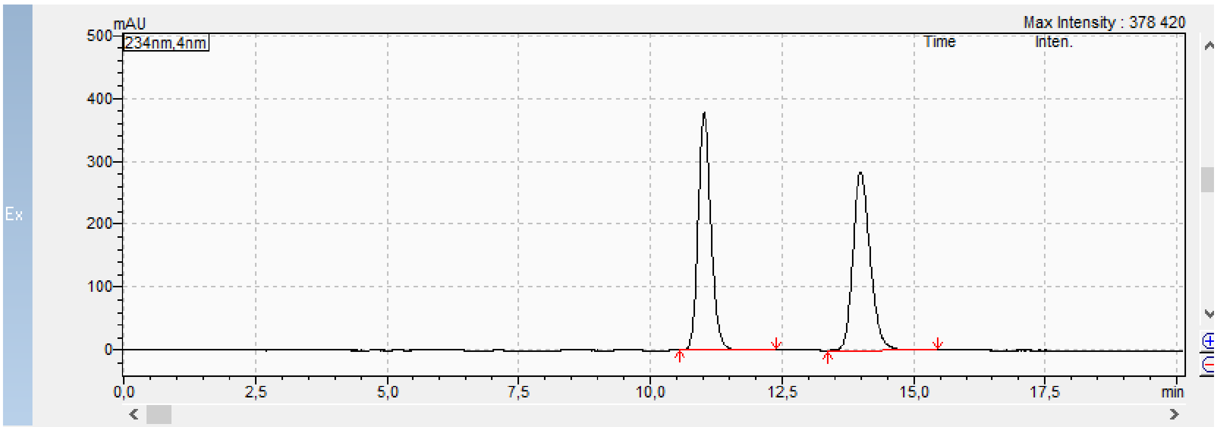

Results View - Peak Table

Peak Table | Compound | Group | Calibration Curve

Peak#	Ret. Time	Area	Height	Peak Start	Peak End	Mark	Conc.	Unit	Area%
1	11,020	6470330	378455	10,560	12,395	S	50,027		50,027
2	13,996	6463220	283470	13,376	15,456		49,973		49,973
Total		12933550	661925				100,000		100,000

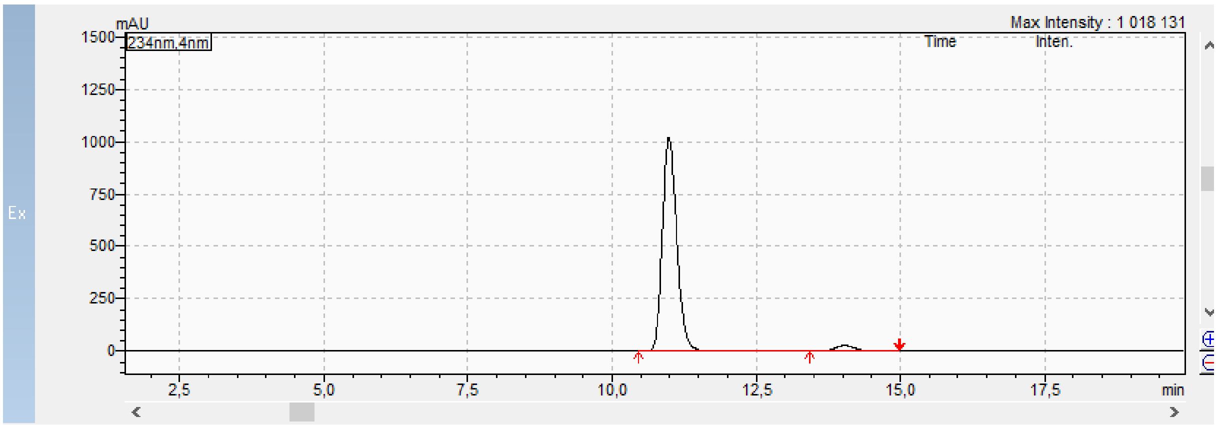

Results View - Peak Table

Peak Table | Compound | Group | Calibration Curve

Peak#	Ret. Time	Area	Height	Peak Start	Peak End	Mark	Conc.	Unit	Area%
1	10,984	17765630	1018227	10,453	15,008	S	96,485		96,485
2	14,034	647130	28048	13,419	14,965	T	3,515		3,515
Total		18412760	1046275				100,000		100,000

for **3v**: *er* = 96:4 (*ee* = 93%)

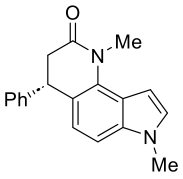

Conditions: IA column
mobile phase: *n*-heptane/*i*-PrOH – 80:20
λ= 227 nm, V = 1.0 ml/min, t = 25 °C
for **5**: t_R = 9.0 min (minor), t_R = 13.0 min (major)

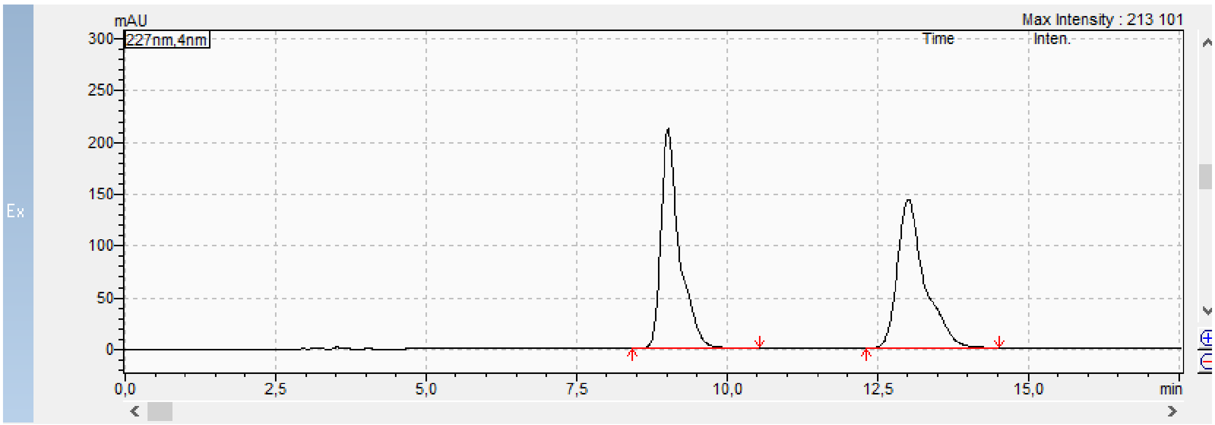

Results View - Peak Table

Peak Table | Compound | Group | Calibration Curve

Peak#	Ret. Time	Area	Height	Peak Start	Peak End	Mark	Conc.	Unit	Area%
1	9,021	4530952	211986	8,416	10,528		50,027		50,027
2	13,012	4526014	143412	12,309	14,517		49,973		49,973
Total		9056966	355398				100,000		100,000

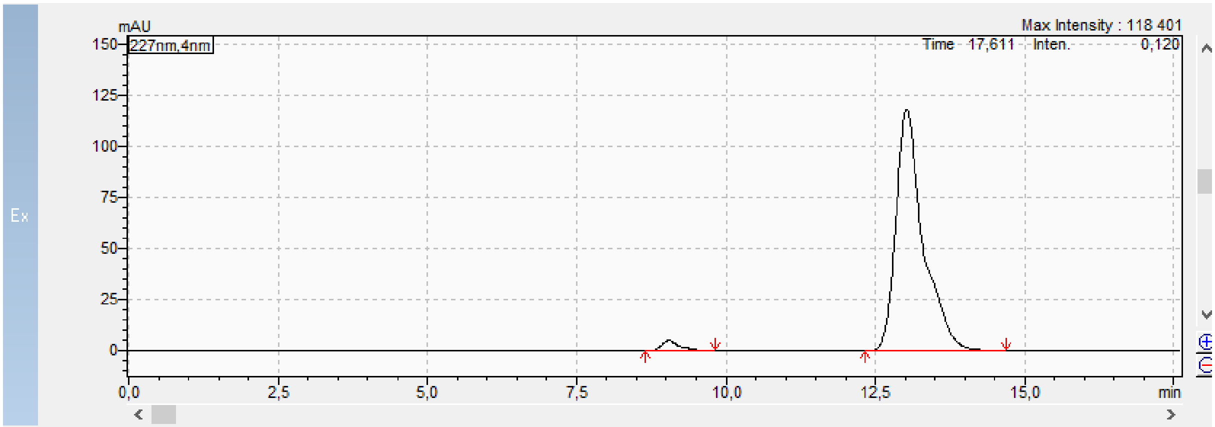

Results View - Peak Table

Peak Table | Compound | Group | Calibration Curve

Peak#	Ret. Time	Area	Height	Peak Start	Peak End	Mark	Conc.	Unit	Area%
1	9,043	106205	4966	8,651	9,824		2,764		2,764
2	13,026	3735796	118211	12,320	14,688		97,236		97,236
Total		3842001	123177				100,000		100,000

for **5**: *er* = 97:3 (*ee* = 95%)

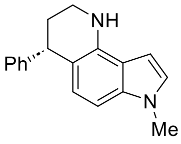

Conditions: IB column
mobile phase: *n*-heptane/*i*-PrOH – 80:20
λ= 313 nm, V = 1.0 ml/min, t = 25 °C
for **6**: t_R = 14.4 min (minor), t_R = 23.9 min (major)

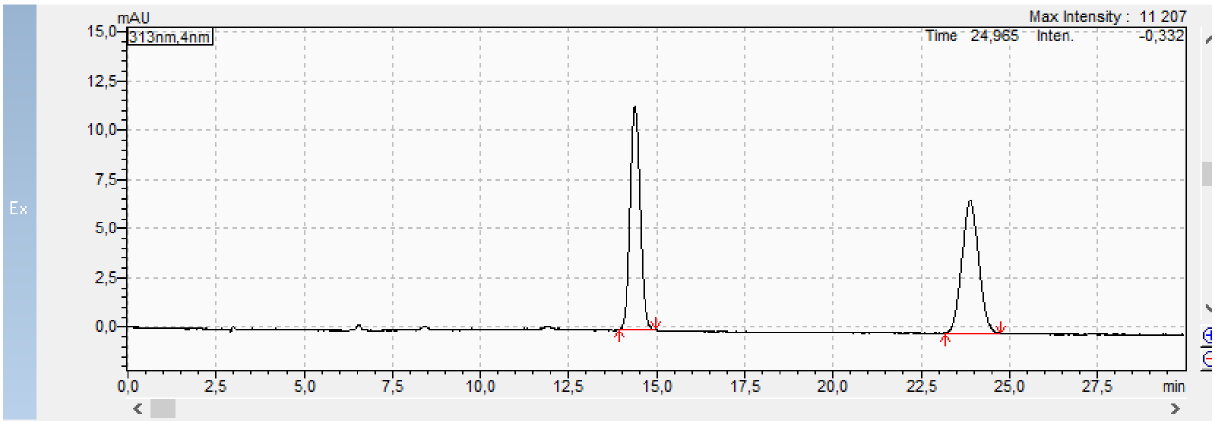

Results View - Peak Table

Peak Table | Compound | Group | Calibration Curve

Peak#	Ret. Time	Area	Height	Peak Start	Peak End	Mark	Conc.	Unit	Area%
1	14,383	223420	11347	13,920	14,955		49,882		49,882
2	23,894	224479	6756	23,168	24,768		50,118		50,118
Total		447898	18103				100,000		100,000

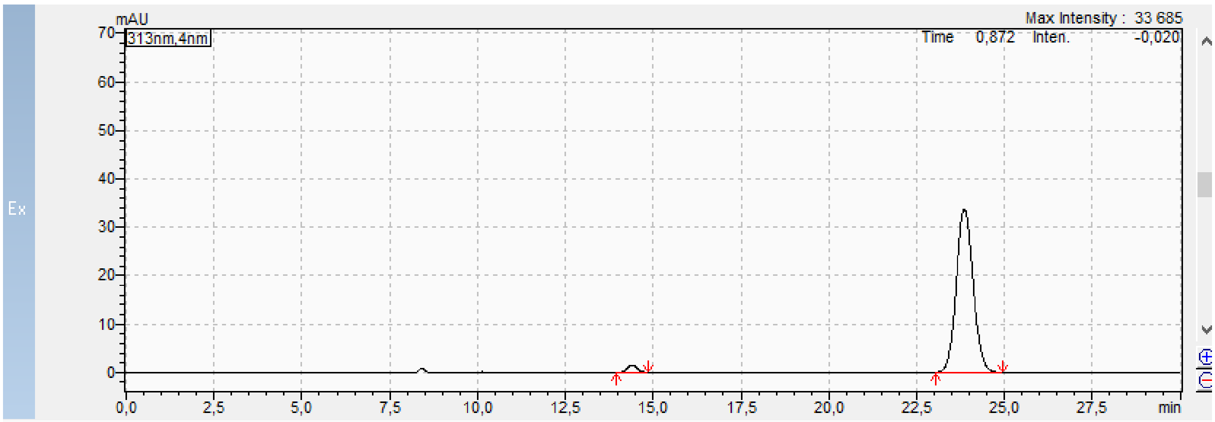

Results View - Peak Table

Peak Table | Compound | Group | Calibration Curve

Peak#	Ret. Time	Area	Height	Peak Start	Peak End	Mark	Conc.	Unit	Area%
1	14,408	31043	1563	13,952	14,869		2,669		2,669
2	23,858	1132056	33694	23,040	24,971		97,331		97,331
Total		1163099	35257				100,000		100,000

for **6**: *er* = 97:3 (*ee* = 95%)

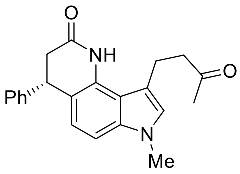

Conditions: IB column
mobile phase: *n*-heptane/*i*-PrOH – 80:20
λ= 227 nm, V = 1.0 ml/min, t = 25 °C
for **7**: t_R = 22.5 min (major), t_R = 24.7 min (minor)

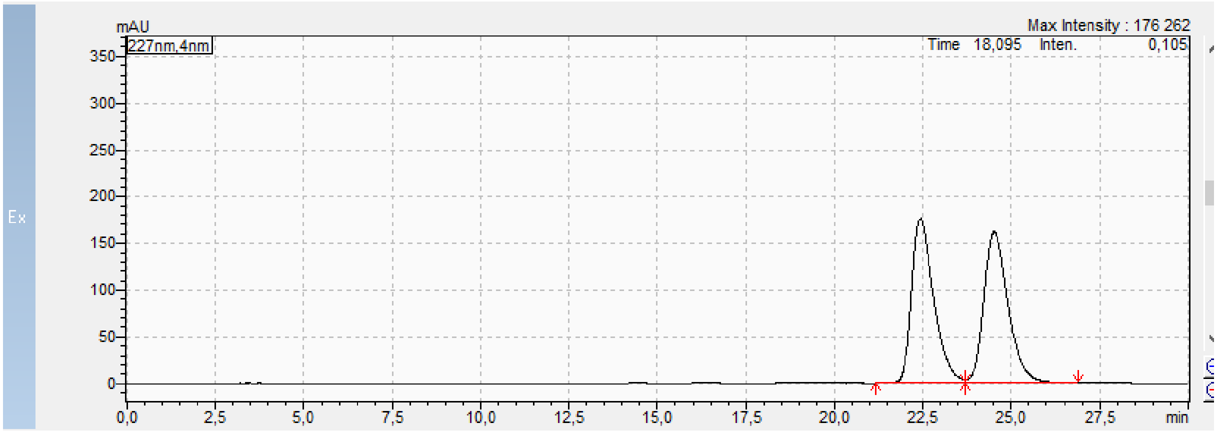

Results View - Peak Table

Peak Table | Compound | Group | Calibration Curve

Peak#	Ret. Time	Area	Height	Peak Start	Peak End	Mark	Conc.	Unit	Area%
1	22,429	7691184	175883	21,173	23,701		49,826		49,826
2	24,518	7744826	162187	23,701	26,891	V	50,174		50,174
Total		15436009	338070				100,000		100,000

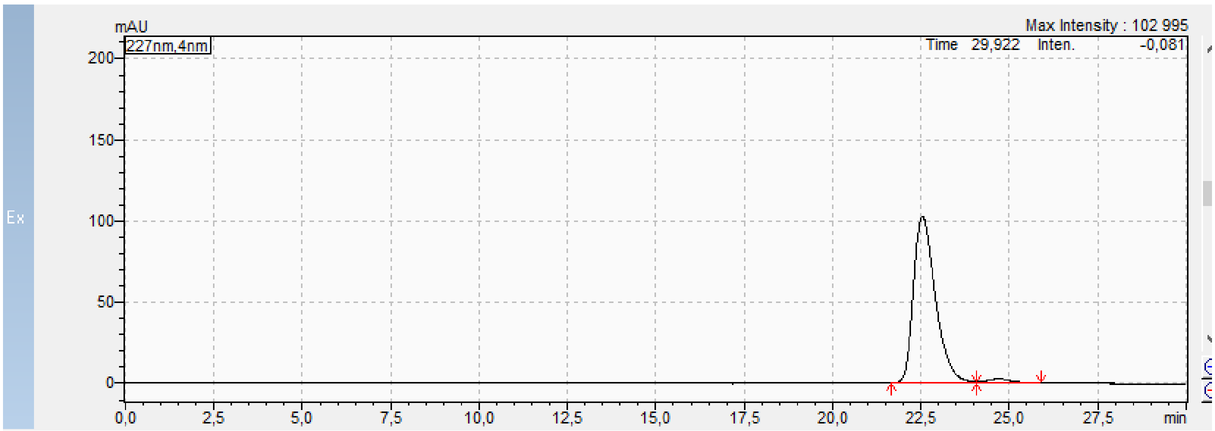

Results View - Peak Table

Peak Table | Compound | Group | Calibration Curve

Peak#	Ret. Time	Area	Height	Peak Start	Peak End	Mark	Conc.	Unit	Area%
1	22,541	4474187	102949	21,664	24,085		96,933		96,933
2	24,693	141559	2833	24,085	25,888	V	3,067		3,067
Total		4615746	105782				100,000		100,000

for **7**: *er* = 97:3 (*ee* = 94%)

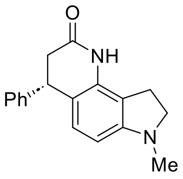

Conditions: IB column
mobile phase: *n*-heptane/*i*-PrOH – 80:20
λ= 237 nm, V = 1.0 ml/min, t = 25 °C
for **8**: t_R = 9.7 min (major), t_R = 13.0 min (minor)

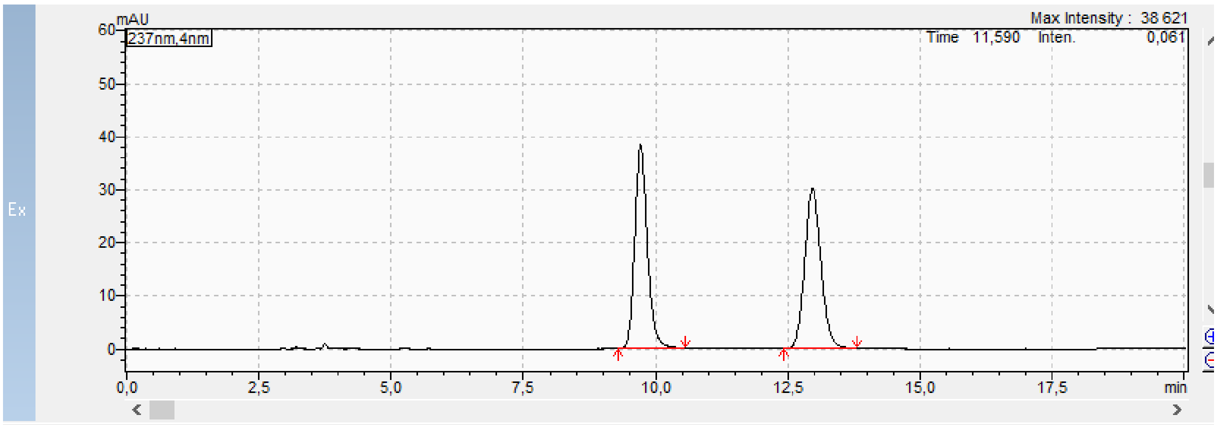

Results View - Peak Table

Peak Table | Compound | Group | Calibration Curve

Peak#	Ret. Time	Area	Height	Peak Start	Peak End	Mark	Conc.	Unit	Area%
1	9,716	643585	38537	9,291	10,560		49,802		49,802
2	12,968	648697	30178	12,427	13,813		50,198		50,198
Total		1292282	68715				100,000		100,000

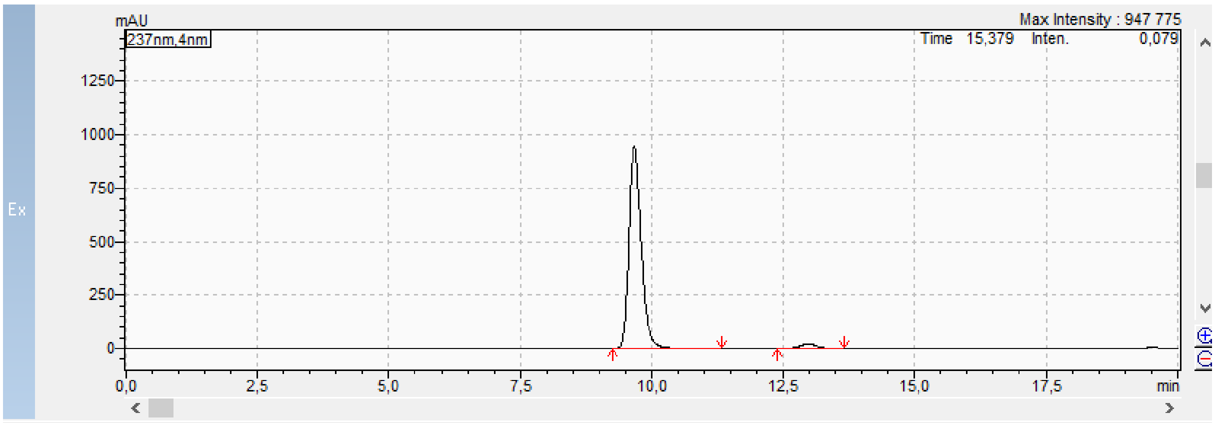

Results View - Peak Table

Peak Table | Compound | Group | Calibration Curve

Peak#	Ret. Time	Area	Height	Peak Start	Peak End	Mark	Conc.	Unit	Area%
1	9,668	15366942	947443	9,248	11,339		97,000		97,000
2	12,971	475256	21163	12,384	13,653		3,000		3,000
Total		15842198	968606				100,000		100,000

for **8**: *er* = 97:3 (*ee* = 94%)

References

(1) Iqbal, S. A.; Cid, J.; Procter, R. J.; Uzelac, M.; Yuan, K.; Ingleson, M. J. Acyl-Directed *ortho*-Borylation of Anilines and C7 Borylation of Indoles using just BBr_3. *Angew. Chem. Int. Ed.* **2019**, *58*, 15381–15385.

(2) Ding, Z.-C.; Li, C.-Y.; Chen, J.-J.; Zeng, J.-H.; Tang, H.-T.; Ding, Y.-J.; Zhan, Z.-P. Palladium/Phosphorus-Doped Porous Organic Polymer as Recyclable Chemoselective and Efficient Hydrogenation Catalyst under Ambient Conditions. *Adv. Synth. Catal.* **2017**, *359*, 2280–2287.

(3) Plé, P. A.; Green, T. P.; Hennequin, L. F.; Curwen, J.; Fennell, M.; Allen, J.; Lambert-van der Brempt, C.; Costello, G. Discovery of a New Class of Anilinoquinazoline Inhibitors with High Affinity and Specificity for the Tyrosine Kinase Domain of c-Src. *J. Med. Chem.* **2004**, *47*, 871–887.

(4) Katritzky, A. R.; Rachwal, S.; Bayyuk, S. An Improved Synthesis of Nitroindoles. 1,3-Dimethyl-4-, 5- and 6-Nitroindoles. *Org. Prep. Proced. Int.* **1991**, *23* (3), 357–363.

(5) Cai, L.; Zhao, Y.; Huang, T.; Meng, S.; Jia, X.; Chan, A. S. C.; Zhao, J. Chiral Phosphoric-Acid-Catalyzed Regioselective and Enantioselective C7-Friedel–Crafts Alkylation of 4-Aminoindoles with Trifluoromethyl Ketones. *Org. Lett.* **2019**, *21* (10), 3538–3542.

(6) Passow, K. T.; Daniel A. Harki, D. A.4-Isocyanoindole-2′-deoxyribonucleoside (4ICIN): An isomorphic indole nucleoside suitable for inverse electron demand Diels-Alder reactions. *Tetrahedron Lett.* **2023**, *132*, 154807.

(7) Gerwe, H.; He, F.; Pottie, E.; Stove, C.; Decker, M. Enlightening the "Spirit Molecule": Photomodulation of the 5-HT_{2A} Receptor by a Light-Controllable *N,N*-Dimethyltryptamine Derivative. *Angew. Chem. Int. Ed.* **2022**, *61*, e202203034.

(8) Xue, B.; Shen, J.; Manna, S.; Doppiu, A.; Gooßen, L. J. Selective Monoarylation of Ammonium Triflate with Aryl Chlorides Catalyzed by $[Pd(\beta\text{-MeNAP})Br]_2$ and AdBrettPhos. *Adv. Synth. Catal.* **2023**, *365*, 3473–3477.

(9) Smith, D.; Marjamaki, A.; Ojala, M.; Pihlavisto, M.; Heino, J.; Kapyla, J.; Pantikainen, O.; Nyronen, T.; Johnson, M.; Huhtala, M. Sulphonamide Derivatives. *US2008255169A1* **2008**.

(10) Gao, H.; Zhou, Z.; Kurti, L. Amination and hydroxylation of arylmetal compounds. *US2018057444A1* **2018**.

(11) Bonini, C.; Cristiani, G.; Funicello, M.; Viggiani, L. Facile Entry to 4- and 5-Hydroxybenzofurane and to Their Amino Derivatives. *Synth. Commun.* **2006**, *36*, 1983–1990.

(12) Wu, Q.; Wu, S.; Zou, J.; Wang, Q.; Mou, C.; Zheng, P.; Chi, Y. R. Carbene-Catalyzed Access to Thiochromene Derivatives: Control of Reaction Pathways via Slow Release of Thiols from Disulfides. *Org. Lett.* **2023**, *25*, 3967–3971.

(13) Sheldrick, G. M. SHELXT - Integrated Space-Group and Crystal-Structure Determination. *Acta Crystallogr. Sect. A Found. Crystallogr.* **2015**, *A71*, 3–8.

(14) Sheldrick, G. M. Crystal Structure Refinement with SHELXL. *Acta Crystallogr. Sect. C Struct. Chem.* **2015**, *C71*, 3–8.

(15) Parsons, S.; Flack, H. D.; Wagner, T. Use of Intensity Quotients and Differences in Absolute Structure Refinement. *Acta Crystallogr. Sect. B Struct. Sci. Cryst. Eng. Mater.* **2013**, *B69*, 249–259.

(16) Neese, F. Software Update: The ORCA Program System—Version 5.0. *WIREs Comput. Mol. Sci.* **2022**, *12*, e1606.

(17) Pracht, P.; Bohle, F.; Grimme, S. Automated Exploration of the Low-Energy Chemical Space with Fast Quantum Chemical Methods. *Phys. Chem. Chem. Phys.* **2020**, *22*, 7169–7192.

(18) Bannwarth, C.; Caldeweyher, E.; Ehlert, S.; Hansen, A.; Pracht, P.; Seibert, J.; Spicher, S.; Grimme, S. Extended tight-binding quantum chemistry methods. *WIREs Comput. Mol. Sci.* **2021**, *11*, e1493.

(19) Wheeler, S. E.; Houk, K. N. Integration Grid Errors for Meta-GGA-Predicted Reaction Energies: Origin of Grid Errors for the M06 Suite of Functionals. *J. Chem. Theory Comput.* **2010**, *6*, 395–404.
(20) Ayers, P. W.; Yang, W.; Bartolotti, L. J. Fukui Function, in Chemical Reactivity Theory: A Density Functional View, ed. P. K. Chattaraj, CRC Press, **2009**, 255–267.
(21) De Proft, F.; Van Alsenoy, C.; Peeters, A.; Langenaeker, W.; Geerlings, P. Atomic Charges, Dipole Moments, and Fukui Functions Using the Hirshfeld Partitioning of the Electron Density. *J. Comput. Chem.* **2002**, *23*,1198–1209.
