# Supplementary figures and images for: Regio- and Enantioselective N-Heterocyclic Carbene-Catalyzed Annulation of Aminoindoles Initiated by Friedel–Crafts Alkylation
Source: Org Lett. 2024 Aug 8;26(33):6993–8. doi: 10.1021/acs.orglett.4c02434 (PMC11348421; doi:10.1021/acs.orglett.4c02434)

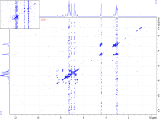

Supplement: Supplementary file 2 — ol4c02434_si_002.zip [file ol4c02434_si_002.zip › Annulation_products/3a_(R1,2=H;PG=H;R3=Ph)/COSY/pdata/1/thumb.png]
